# Supplementary material for: Photocatalyzed cycloaromatization of vinylsilanes with arylsulfonylazides
Source: Nat Commun. 2021 Jun 3;12:3304. doi: 10.1038/s41467-021-23326-2 (PMC8175346; doi:10.1038/s41467-021-23326-2)
Supplement: Supplementary file 1 — Supplementary Information [file 41467_2021_23326_MOESM1_ESM.pdf]

Supplementary Information

Photocatalyzed Cycloaromatization of Vinylsilanes with Arylsulfonylazides

Fengjuan Chen[1, §], Youxiang Shao[2, §], Mengke Li[3, §], Can Yang[1], Shi-Jian Su[3]*, Huanfeng Jiang[1], Zhuofeng Ke[2]*, and Wei Zeng[1]*

[1]*Key Laboratory of Functional Molecular Engineering of Guangdong Province, School of Chemistry and Chemical Engineering, South China University of Technology, Guangzhou 510641, China*

[2]*School of Materials Science and Engineering, PFCM Lab, Sun Yat-sen University, Guangzhou 510275, China*

[3]*State Key Laboratory of Luminescent Materials and Devices, Institute of Polymer Optoelectronic Materials and Devices, South China University of Technology, Guangzhou 510641, China*

Table of Contents

I. General Experimental Information. 4

II. Material and Methods. 4

1. General procedure for the preparation of sulfonylazides **2a-2t**.… 4

2. The procedure for the preparation of sila-enyne **1a-1l** ,**1n-1q**, **1m** and **1s**.… 4

3. Spectroscopic data of vinylsilanes.… 5

4. The procedure for the preparation of sila-enyne **1r**.… 10

5. The procedure for the preparation of vinylether **1u**.… 11

6. The procedure for the preparation of sulfonylhydrazine **10**.… 12

III. Optimization of the reaction conditions. 12

Supplementary Table 1. The effect of photocatalysts on the cycloaromatization of sila-enyne **1a** with arylsulfonylazide **2a**..12

Supplementary Table 2. The effect of bases on the cycloaromatization of sila-enyne **1a** with arylsulfonylazide **2a**...13

Supplementary Table 3. The effect of solvents on the cycloaromatization of sila-enyne **1a** with

arylsulfonylazide **2a** 13

Supplementary Table 4. The effect of the temperature on the cycloaromatization of sila-enyne **1a** with arylsulfonylazide **2a** 14

Supplementary Table 5. The effect of the dosage of arylsulfonylazide on the cycloaromatization of sila-enyne **1a** with arylsulfonylazide **2a** 14

Supplementary Table 6. Control experiments 14

IV. General Procedures and Characterization Data of Products. 14

1. The procedure for the photocatalyzed cycloaromatization of vinylsilanes **1** with arylsulfonyl azides **2a**. 14
2. The coupling-cyclization of allylic dimethylsilanes **4** with arylsulfonyl azides **2**. 19
3. Arylsulfonylazide scope. 21

V. **Synthetic Application**. 26

VI. **Photo-Physical Properties**. 26

1. General information. 26

2. Photophysical characterization of compound **8**. 26

3. Determination of triplet state energy level. 27

VII. Control Experiments for Mechanistic Investigation. 27

1. Photocatalyzed cycloaromatization of sulfonylhydrazine **10** with vinylsilane **1a** 27
2. The effect of the radical trapper TEMPO on the cycloaromatization of vinylsilane **1a** with 4-methylbenzenesulfonyl azide **2a**. 28
3. Photocatalyzed coupling-cyclization of *ortho*-alkynylaryl vinylether **1u** with 4-chlorobenzenesulfonyl azide **2h** 28
4. Photocatalyzed cycloaromatization of vinylsilane **1a** with *ortho*-methylphenylsulfonyl azide **2s** 29
5. Cyclic voltammetry experiments 30

6. Stern-Volmer quenching studies...30

7. Determination of the structure of compound **14** 31

8. The possible mechanism for carbocyclization of allyldimethylsilane **4** 33

VIII. Computational details 33

1. Computational methods 33

2. Optimized cartesian coordinates 34

IX. Single Crystal Structure and Crystallographic Data. 42

X. Supplementary Reference…………………………………………………………………….46

Appendix-I: Spectral Copies of ^{1}H and ^{13}C NMR of Compounds………………………..…...47

Appendix-II: Spectral Copies of HR-MS Spectrum for All the Isolated Products…………111

I. General Experimental Information

All reactions were conducted in flame-dried glassware with magnetic stirring. Unless otherwise noted, all solvents were used as received without further purification. Reactions were performed in 10 mL vials with two Kessil LEDs in air (30 W, 456 nm, approximately 3 cm away from the light source). Purifications of reaction products were carried out by flash chromatography using Qingdao Haiyang Chemical Co. Ltd silica gel (300-400 mesh) or SiO_2 gel column chromatography. IR spectra were obtained on a Nicolet 210 spectrophotometer and reported in terms of frequency of absorption (cm^{-1}) in potassium bromide (KBr) pellet. ^1H NMR and ^{13}C NMR spectra were recorded with tetramethylsilane (TMS) as internal standard at ambient temperature unless otherwise indicated on a Bruker Avance DPX 600 Fourier transform spectrometer operating at 400 MHz or 500 MHz for ^1H NMR and 101 MHz or 126 MHz for ^{13}C NMR. ^1H and ^{13}C NMR spectral data are reported as chemical shifts (δ) in parts per million (ppm) relative to the solvent peak using the Bruker internal referencing procedure (edlock). Coupling constants (*J*) are measured in Hertz (Hz). The following abbreviations are used to describe multiplicities s = singlet, d = doublet, t = triplet, q = quartet, m = multiplet, dd = doublet of doublets. NMR spectra were processed in MestreNova. High resolution mass spectra (HRMS, *m/z*) were recorded on an IF-TOF spectrometer (Micromass). The geometries of all species were fully optimized using Truhlar's pure functional M06L with the triple-zeta 6-311G (d, p) basis set. Crystal data were collected on a Bruker D8 Advance employing graphite monochromated Mo - Kα radiation (λ = 0.71073 Å) at 293(2) K and operating in the φ-ω scan mode. The structure was solved by direct methods SHELXS-97. Cyclic voltammetry was performed on conventional three-electrode system at CHI660E electrochemical workstation (Chenhua, Shanghai, China). All phosphorescence spectra were detected at 77 K with a delay time of 0.05 ms using FluoroMax-4 spectrofluorometer.

II. Materials and Methods

1. General procedure for the preparation of sulfonylazides **2a-2t**[1]

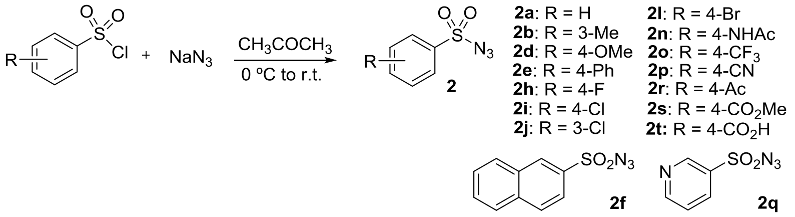

Sulfonyl azides **2a-2t** were prepared using the previously reported methods.[1] To a solution of sulfonyl chloride (5.0 mmol, 1 eq.) in acetone (10.0 mL) at 0 ℃ was added a solution of NaN_3 (0.65 g, 2.0 eq.) in water (3 mL). The mixture was stirred for 1 ~ 2 h at room temperature. The mixture was then concentrated, diluted with water (30.0 mL), and extracted with AcOEt (3 × 10.0 mL). The combined organic extracts were dried over Na_2SO_4 and concentrated. The residue was purified by flash chromatography using ethyl acetate/petroleum ether as eluent to afford the the corresponding sulfonylazides **2a-2t**.

2. The procedure for the preparation of sila-enyne **1a-1l**, **1n-1q**, **1m** and **1s**

General procedure **A** [2]

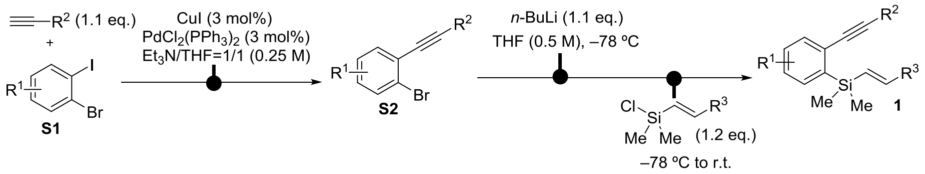

Step 1. To a solution of **S1**, $PdCl_2(PPh_3)_2$ (3 mol %) and CuI (3 mol %) in Et_3N/THF = 1/1 (0.25 M) was added alkynes (1.1 equiv.) at room temperature. After the mixture was stirred for 4 ~ 12 h, the mixture was filtered through Celite cake and the solvent was evaporated under reduced pressure. The residue was subjected to column chromatography on silica gel to give **S2**.

Step 2. To a solution of compound **S2** in THF (0.5 M) was added dropwise *n*-BuLi (1.1 equiv.) at -78 ℃, After the mixture was stirred at -78 ℃ for 1 h, vinylchlorodimethylsilane (1.2 equiv.) was added dropwise to the mixture. Then the reaction mixture was stirred at -78 ℃ for 1 h, and warmed to room temperature for 24 ~ 36 h. After then, the mixture was diluted with saturated NH_4Cl and organic compound was extracted with Et_2O. The organic layer was dried over Na_2SO_4, and the solvent was evaporated. The residue was subjected to column chromatography on silica gel to give compounds **1**.

General procedure **B** [2]

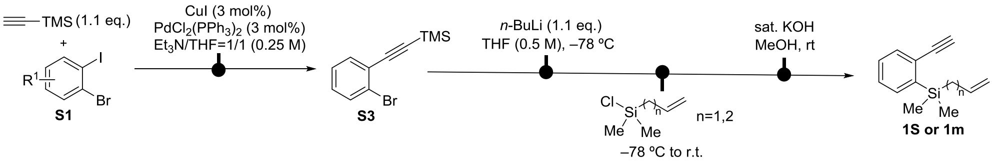

Step 1. To a solution of **S1**, $PdCl_2(PPh_3)_2$ (3 mol %) and CuI (3 mol %) in Et_3N/THF = 1/1 (0.25 M) was added trimethylsilylacetylene (1.1 equiv.) at r.t. After the mixture was stirred for 4 ~ 12 h, the mixture was filtered through Celite cake and the solvent was evaporated under reduced pressure. The residue was subjected to column chromatography on silica gel to give **S3**.

Step 2. To a solution of compound **S3** in THF (0.5 M) was added dropwise *n*-BuLi (1.1 equiv.) at -78 ℃, After the mixture was stirred at -78 ℃ for 1 h, vinylchlorodimethylsilane (1.2 equiv.) was added dropwise to the mixture. The reaction mixture was stirred at -78 ℃ for 1 h, and warmed to room temperature for 24 ~ 36 h. After then, saturated KOH in MeOH was added to the reaction mixture and the whole was stirred at room temperature for 30 min. The mixture was diluted with H_2O and organic compound was extracted with CH_2Cl_2. The organic layer was dried over Na_2SO_4, and the solvent was evaporated. The residue was subjected to column chromatography on silica gel to give compound **1S** and **1m**.

3. Spectroscopic data of vinylsilanes

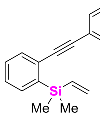
1a

Dimethyl(2-(phenylethynyl)phenyl)(vinyl)silane 1a. By using the general procedure **A**, the title compound was prepared from 1-bromo-2-(phenylethynyl)benzene (1.28 g, 5.0 mmol) and chlorodimethyl(vinyl)silane (660.0 mg, 5.5 mmol) and was purified by column chromatography to give colorless liquid (996 mg, 76%); TCL (petroleum ether): R_f = 0.96; ^{1}H NMR (400 MHz, $CDCl_3$) δ 7.55– 7.50 (m, 4H), 7.29 – 7.23 (m, 5H), 6.50 (dd, J = 20.4, 14.6 Hz, 1H), 6.06 (dd, J = 14.6, 3.6 Hz, 1H), 5.80 (dd, J = 20.4, 3.6 Hz, 1H), 0.50 (s, 6H); ^{13}C NMR (101 MHz, $CDCl_3$) δ 143.4, 140.6, 137.2, 135.3, 135.2, 134.3, 133.9, 131.7, 131.3, 131.1, 131.0, 130.9, 130.2, 126.2, 126.0, 94.9, 93.9, 0.0; IR (KBr): 3823, 3745, 3649, 1516, 678 cm^{-1}; HRMS-ESI (m/z): $[M+H]^+$ calcd. for $C_{18}H_{19}Si$, 263.1251; found, 263.1254.

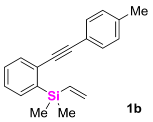

1b

Dimethyl(2-(*p*-tolylethynyl)phenyl)(vinyl)silane 1b. By using the general procedure **A**, the title compound was prepared from 1-bromo-2-(*p*-tolylethynyl)benzene (1.08 g, 4.0 mmol) and chlorodimethyl(vinyl)silane (528.0 mg, 4.4 mmol) and was purified by column chromatography to give colorless liquid (773 mg, 70 %); TCL (petroleum ether): R_f = 0.96; ^{1}H NMR (400 MHz, $CDCl_3$) δ 7.54– 7.51 (m, 2H), 7.41 (d, J = 7.7 Hz, 2H), 7.32 – 7.24 (m, 2H), 7.14 (d, J = 7.4 Hz, 2H), 6.49 (dd, J = 20.3, 14.7 Hz, 1H), 6.06 (dd, J = 14.6, 3.5 Hz, 1H), 5.80 (dd, J = 20.3, 3.4 Hz, 1H), 2.34 (s, 3H), 0.50 (s, 6H); ^{13}C NMR (101 MHz, $CDCl_3$) δ 140.7, 138.4, 138.1, 134.6, 132.6, 132.5, 131.2, 129.3, 129.1, 128.9, 127.4, 120.6, 92.5, 90.6, 21.6, -2.6; IR (KBr): 3748, 3649, 3437, 2919, 1641, 1513 cm^{-1}; HRMS-ESI (m/z): $[M+H]^+$ calcd. for $C_{19}H_{21}Si$, 277.1407; found 277.1414.

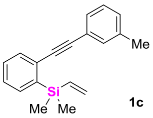

1c

Dimethyl(2-(*m*-tolylethynyl)phenyl)(vinyl)silane 1c. By using the general procedure **A**, the title compound was prepared from 1-bromo-2-(m-tolylethynyl)benzene (1.08 g, 4.0 mmol) and chlorodimethyl(vinyl)silane (528.0 mg, 4.4 mmol) and was purified by column chromatography to give pale yellow liquid (883 mg, 80%); TCL (petroleum ether): R_f = 0.96; ^{1}H NMR (500 MHz, $CDCl_3$) δ 7.53 (dd, J = 12.6, 7.5 Hz, 2H), 7.33 (dd, J = 11.2, 3.5 Hz, 3H), 7.29 (d, J = 7.3 Hz, 1H), 7.23 (dd, J = 12.5, 4.9 Hz, 1H), 7.14 (d, J = 7.6 Hz, 1H), 6.50 (dd, J = 15.0, 1H), 6.07 (dd, J = 5,0 1H), 5.81 (dd, J = 5.0, 3.6 Hz, 1H), 2.35 (s, 3H), 0.50 (s, 6H); ^{13}C NMR (126 MHz, $CDCl_3$) δ 140.8, 138.1, 138.0, 134.6, 132.7, 132.6, 131.8, 129.2, 129.0, 128.7, 128.4, 128.4, 127.4, 123.3, 92.4, 90.8, 21.4, -2.7; IR (KBr): 3728, 3627, 3203, 2958, 2896, 2372, 2319, 1432, 1371, 1264, 1011, 740 cm^{-1}; HRMS-ESI (m/z): $[M+H]^+$ calcd. for $C_{19}H_{21}Si$, 277.1407; found 277.1414.

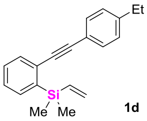

1d

(2-((4-Ethylphenyl)ethynyl)phenyl)dimethyl(vinyl)silane 1d. By using the general procedure **A**, the title compound was prepared from 1-bromo-2-((4-ethylphenyl)ethynyl)benzene (1.14 g, 4.0 mmol) and chlorodimethyl(vinyl)silane (528.0 mg, 4.4 mmol) and was purified by column chromatography to give yellow liquid (824 mg, 71%); TCL (petroleum ether): R_f = 0.96; ^{1}H NMR (500 MHz, $CDCl_3$) δ 7.53 (dd, J = 7.5, 5.7, 2.3 Hz, 2H), 7.44 (d, J = 10.0, 2H), 7.35 – 7.30 (m, 1H), 7.30 – 7.26 (m, 1H), 7.18 (d, J = 7.9 Hz, 2H), 6.49 (dd, 1H), 6.06 (dd, 1H), 5.80 (dd, J = 20.4, 5.2, 3.7 Hz, 1H), 2.66 (q, J = 7.6 Hz, 2H), 1.24 (t, J = 7.6, 3.9 Hz, 3H), 0.49 (s, 6H); ^{13}C NMR (126 MHz, $CDCl_3$) δ 144.7, 140.7, 138.0, 134.6, 132.6, 132.5, 131.3, 129.0, 128.9, 128.0, 127.3, 120.7, 92.4, 90.5, 28.9, 15.4, -2.7; IR (KBr): 3836, 3740, 3229, 2958, 1721, 1512, 1265, 741 cm^{-1}; HRMS-ESI (m/z): $[M+H]^+$ calcd. for $C_{20}H_{23}Si$, 291.1564; found 291.1574.

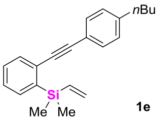

1e

(2-((4-Butylphenyl)ethynyl)phenyl)dimethyl(vinyl)silane 1e. By using the general procedure **A**, the title compound was prepared from 1-bromo-2-((4-butylphenyl)ethynyl)benzene (936.0 mg, 3.0 mmol) and chlorodimethyl(vinyl)silane (396.0 mg, 3.3 mmol) and was purified by column chromatography to give yellow liquid (668 mg, 70 %); TCL (petroleum ether): R_f = 0.94; ^{1}H NMR (500 MHz, $CDCl_3$) δ 7.53 (dd, J = 11.6, 8.0 Hz, 2H), 7.43 (d, J = 7.2 Hz, 2H), 7.33 (t, J = 7.4 Hz, 1H), 7.28 (t, J = 7.3 Hz, 1H), 7.16 (d, J = 7.4 Hz, 2H), 6.50 (dd, J = 19.9, 15.1 Hz, 1H), 6.07 (d, J = 14.6 Hz, 1H), 5.81 (d, J = 20.4 Hz, 1H), 2.61 (q, J = 7.3 Hz, 2H), 1.63 – 1.55 (m, 2H), 1.39 – 1.30 (m, 2H), 0.93 (t, J = 6.6 Hz, 3H), 0.50 (s, 6H); ^{13}C NMR (101 MHz, $CDCl_3$) δ 143.4, 140.7, 138.0, 134.5, 132.6, 132.5, 131.2, 129.0, 128.9, 128.6, 127.3, 120.6, 92.4, 90.5, 35.6, 33.4, 22.4, 14.0, -2.7; IR (KBr):

3154, 1622, 1400, 1109, 459 cm^{-1}; HRMS-ESI (m/z): $[M+H]^+$ calcd. for $C_{22}H_{27}Si$, 319.1877; found 319.1884.

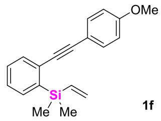
1f

(2-((4-Methoxyphenyl)ethynyl)phenyl)dimethyl(vinyl)silane 1f. By using the general procedure **A**, the title compound was prepared from 1-bromo-2-((4-methoxyphenyl)ethynyl)benzene (858.0 mg, 3.0 mmol) and chlorodimethyl(vinyl)silane (396.0 mg, 3.3 mmol) and was purified by column chromatography to give pale yellow liquid (806 mg, 92 %); TCL (petroleum ether): R_f = 0.92; ^{1}H NMR (500 MHz, $CDCl_3$) δ 7.52 (t, J = 7.6 Hz, 2H), 7.46 (d, J = 8.5 Hz, 2H), 7.32 (t, J = 7.3 Hz, 1H), 7.27 (t, J = 7.2 Hz, 1H), 6.88 (d, J = 8.6 Hz, 2H), 6.49 (dd, 1H), 6.06 (dd, J = 14.6, 3.5 Hz, 1H), 5.80 (dd, J = 20.4, 3.5 Hz, 1H), 3.80 (s, 3H), 0.49 (s, 6H); ^{13}C NMR (126 MHz, $CDCl_3$) δ 159.7, 140.5, 138.0, 134.6, 132.7, 132.6, 132.3, 129.0, 129.0, 127.2, 115.7, 114.1, 92.2, 89.9, 55.3, -2.7; IR (KBr): 3843, 3731, 3621, 3177, 1509, 1399, 1104, 570 cm^{-1}; HRMS-ESI (m/z): $[M+H]^+$ calcd. for $C_{19}H_{21}OSi$, 293.1356; found 293.1356.

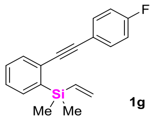
1g

(2-((4-Fluorophenyl)ethynyl)phenyl)dimethyl(vinyl)silane 1g. By using the general procedure **A**, the title compound was prepared from 1-bromo-2-((4-fluorophenyl)ethynyl)benzene (1.12 g, 4.1 mmol) and chlorodimethyl(vinyl)silane (540.0 mg, 4.5 mmol) and was purified by column chromatography to give pale yellow liquid (874 mg, 78 %); TCL (petroleum ether): R_f = 0.97; ^{1}H NMR (500 MHz, $CDCl_3$) δ 7.54 (t, J = 7.0 Hz, 2H), 7.51 – 7.46 (m, 2H), .37 – 7.28 (m, 2H), 7.05 (t, J = 8.6 Hz, 2H), 6.47 (dd, J = 20.4, 14.6 Hz, 1H), 6.07 (dd, J = 14.6, 3.6 Hz, 1H), 5.80 (dd, J = 20.4, 3.6 Hz, 1H), 0.49 (s, 6H); ^{13}C NMR (101 MHz, $CDCl_3$) δ 162.5 (d, J = 250.5 Hz, $^1J_{CF}$), 140.7, 137.9, 134.6, 133.2, 133.1, 132.7, 132.5, 129.1, 128.4, 127.6, 119.6 (d, J = 3.0 Hz, $^3J_{CF}$), 115.8 (d, J = 22.2 Hz, $^2J_{CF}$), 90.9 (d, J = 25.3 Hz, $^2J_{CF}$), -2.7; IR (KBr): 3850, 3733, 3622, 3193, 2952, 1372, 1265, 1060, 741 cm^{-1}; HRMS-ESI (m/z): $[M+H]^+$ calcd. for $C_{18}H_{18}FSi$, 281.1156; found 281.1158.

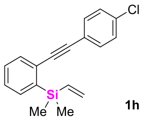
1h

(2-((4-Chlorophenyl)ethynyl)phenyl)dimethyl(vinyl)silane 1h. By using the general procedure **A**, the title compound was prepared from 1-bromo-2-((4-chlorophenyl)ethynyl)benzene (870.0 mg, 3.0 mmol) and chlorodimethyl(vinyl)silane (396.0 mg, 3.3 mmol) and was purified by column chromatography to give colorless liquid (675 mg, 76%); TCL (petroleum ether): R_f = 0.94; ^{1}H NMR (400 MHz, $CDCl_3$) δ 7.58 (t, J = 10.0 Hz, 2H), 7.47 (t, J = 8.7 Hz, 2H), 7.38 – 7.34 (m, 4H), 6.51 (dd, J = 20.4, 14.6 Hz, 1H), 6.11 (dd, J = 14.6, 3.5 Hz, 1H), 5.84 (dd, J = 20.4, 3.5 Hz, 1H), 0.53 (s, 6H); ^{13}C NMR (101 MHz, $CDCl_3$) δ 140.8, 137.8, 134.6, 134.3, 132.7, 132.5, 132.5, 129.1, 128.8, 128.3, 127.7, 122.0, 92.1, 91.0, -2.7; IR (KBr): 3834, 3759, 3651, 3228, 1511, 741 cm^{-1}; HRMS-ESI (m/z): $[M+H]^+$ calcd. for $C_{18}H_{18}ClSi$, 297.0861; found 297.0862.

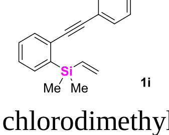
1i

(2-((4-Bromophenyl)ethynyl)phenyl)dimethyl(vinyl)silane 1i. By using the general procedure **A**, the title compound was prepared from 1-bromo-2-((4-bromophenyl)ethynyl)benzene (1.0 g, 3.0 mmol) and chlorodimethyl(vinyl)silane (396.0 mg, 3.3 mmol) and was purified by column chromatography to give colorless liquid (755 mg, 74%); TCL (petroleum ether): R_f = 0.94; ^{1}H NMR (500 MHz, $CDCl_3$) δ 7.53 (dd, J = 10.0, 4.1 Hz, 2H), 7.48 (d, J = 8.4 Hz, 2H), 7.36 (d, J = 8.4 Hz, 2H), 7.34 – 7.29 (m, 2H), 6.46 (dd, J = 20.4, 14.6 Hz, 1H), 6.06 (dd, J = 14.6, 3.6 Hz, 1H), 5.79 (dd, J = 20.4, 3.6 Hz, 1H), 0.48 (s, 6H); ^{13}C NMR (126 MHz, $CDCl_3$) δ 140.8, 137.8, 134.7, 132.7, 132.7, 132.5, 131.8, 129.1, 128.3, 127.8, 122.5, 122.5, 92.3, 91.1, -2.7; IR (KBr): 3730, 3287, 2670, 1498, 1370, 827 cm^{-1}; HRMS-ESI (m/z): $[M+H]^+$ calcd. for $C_{18}H_{18}BrSi$, 341.0356; found 341.0359.

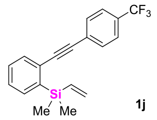

1j

Dimethyl(2-((4-(trifluoromethyl)phenyl)ethynyl)Phenyl)(vinyl)silane 1j. By using the general procedure **A**, the title compound was prepared from 1-bromo-2-((4-(trifluoromethyl)phenyl)ethynyl)benzene (972.0 mg, 3.0 mmol) and chlorodimethyl(vinyl)silane (396.0 mg, 3.3 mmol) and was purified by column chromatography to give colorless liquid (752 mg, 76%); TCL (petroleum ether): R_f = 0.92; ^{1}H NMR (500 MHz, $CDCl_3$) δ 7.61 (s, 4H), 7.58 – 7.54 (m, 2H), 7.38 – 7.32 (m, 2H), 6.47 (dd, J = 20.4, 14.6 Hz, 1H), 6.08 (dd, J = 14.6, 3.5 Hz, 1H), 5.81 (dd, J = 20.4, 3.5 Hz, 1H), 0.50 (s, 6H); ^{13}C NMR (126 MHz, $CDCl_3$) δ 141.1, 137.7, 134.7, 132.8, 132.7, 131.8 (d, J = 6.0 Hz, $^3J_{CF}$), 131.5, 129.1, 128.9, 128.2 (d, J = 78.0 Hz, $^2J_{CF}$), 128.1, 125.4 (q, J = 3.8 Hz, $^3J_{CF}$), 124.0 (d, J = 272.2 Hz, $^1J_{CF}$), 93.5, 90.7, -2.7; IR (KBr): 3730, 3230, 2003, 1443, 1264, 741 cm^{-1}; HRMS-ESI (m/z): $[M+H]^+$ calcd. for $C_{19}H_{18}F_3Si$, 331.1124; found 331.1136.

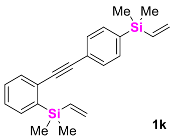

1k

(2-((4-(Dimethyl(vinyl)silyl)phenyl)ethynyl)phenyl)dimethyl(vinyl)silane 1k. By using the general procedure **A**, the title compound was prepared from 1-bromo-2-((4-bromophenyl)ethynyl)benzene (1.7 g, 5.0 mmol) and chlorodimethyl(vinyl)silane (660.0 mg, 5.5 mmol) and was purified by column chromatography to give colorless liquid (657 mg, 38 %); TCL (petroleum ether): R_f = 0.98; ^{1}H NMR (400 MHz, $CDCl_3$) δ 7.56 (d, J = 7.4 Hz, 1H), 7.54 – 7.48 (m, 5H), 7.38 – 7.27 (m, 2H), 6.49 (dd, J = 20.4, 14.6 Hz, 1H), 6.28 (dd, J = 20.2, 14.6 Hz, 1H), 6.07 (dd, J = 14.7, 3.4 Hz, 2H), 5.78 (dd, J = 20.0, 16.2, 3.7 Hz, 2H), 0.50 (s, 6H), 0.36 (s, 6H); ^{13}C NMR (101 MHz, $CDCl_3$) δ 140.8, 139.0, 137.9, 137.6, 134.6, 133.8, 133.2, 132.7, 132.6, 130.4, 129.0, 128.6, 127.5, 124.0, 92.3, 91.7, -2.7, -3.0; IR (KBr): 2928, 1648, 1507, 470 cm^{-1}; HRMS-ESI (m/z): $[M+H]^+$ calcd. for $C_{22}H_{27}Si_2$, 347.1646; found 347.1650.

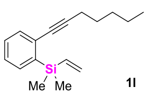

1l

(2-(Hept-1-yn-1-yl)phenyl)dimethyl(vinyl)silane 1l. By using the general procedure **A**, the title compound was prepared from 1-bromo-2-(hept-1-yn-1-yl)benzene (775.0 mg, 3.1 mmol) and chlorodimethyl(vinyl)silane (408.0 mg, 3.4 mmol) and was purified by column chromatography to give colorless liquid (530 mg, 69%); TCL (petroleum ether): R_f = 0.96; ^{1}H NMR (400 MHz, $CDCl_3$) δ 7.50 (d, J = 7.3 Hz, 1H), 7.45 (d, J = 7.4 Hz, 1H), 7.33 – 7.25 (m, 2H), 6.48 (dd, J = 20.4, 14.6 Hz, 1H), 6.09 (dd, J = 14.6, 3.7 Hz, 1H), 5.81 (dd, J = 20.4, 3.7 Hz, 1H), 2.45 (t, J = 7.2 Hz, 2H), 1.69 – 1.62 (m, 2H), 1.51 – 1.44 (m, 2H), 1.40 (dd, J = 14.5, 7.2 Hz, 2H), 0.96 (t, J = 7.2 Hz, 3H), 0.48 (s, 6H); ^{13}C NMR (101 MHz, $CDCl_3$) δ 140.3, 138.2, 134.4, 132.4, 132.2, 129.6, 128.9, 126.7, 93.6, 82.1, 31.3, 28.3, 22.3, 19.6, 14.0, -2.8; IR (KBr): 3731, 3624, 3164, 2959, 1397, 1105 cm^{-1}; HRMS-ESI (m/z): $[M+H]^+$ calcd. for $C_{17}H_{25}Si$, 257.1720; found 257.1731.

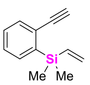

1m

(2-Ethynylphenyl)dimethyl(vinyl)Silane 1m. By using the general procedure **B**, The title compound was prepared from ((2-bromophenyl)ethynyl)trimethylsilane (1.3 g, 5.0 mmol) and chlorodimethyl(vinyl)silane (660.0 mg, 5.5 mmol) and was purified by column chromatography to give yellow liquid (856 mg, 92%); TCL (petroleum ether): R_f = 0.98; ^{1}H NMR (400 MHz, $CDCl_3$) δ 7.60 – 7.55 (m, 2H), 7.39 – 7.34 (m, 2H), 6.52 (dd, J = 20.4, 14.7 Hz, 1H), 6.14 (dd, J = 14.6, 3.5 Hz, 1H), 5.85 (dd, J = 20.4, 3.5 Hz, 1H), 3.27 (s, 1H), 0.53 (s, 6H); ^{13}C NMR (101 MHz, $CDCl_3$) δ 141.3, 137.8, 134.6, 133.4, 132.7, 128.9, 128.0, 127.5, 85.1, 80.4, -2.8; IR (KBr): 3301, 1942, 1511, 1272, 896, 739 cm^{-1}; HRMS-ESI (m/z): $[M]^+$ calcd. for $C_{12}H_{14}Si$, 186.0865; found 186.1276.

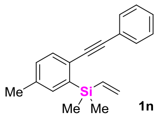

1n

Dimethyl(5-methyl-2-(phenylethynyl)phenyl)(vinyl)silane 1n. By using the general procedure **A**, the title compound was prepared from

2-bromo-4-methyl-1-(phenylethynyl)benzene (810.0 mg, 3.0 mmol) and chlorodimethyl(vinyl)silane (396.0 mg, 3.3 mmol) and was purified by column chromatography to give pale yellow liquid (704 mg, 85%); TCL (petroleum ether): R_f = 0.96; ^{1}H NMR (400 MHz, $CDCl_3$) δ 7.57 (dd, J = 7.7, 1.6 Hz, 2H), 7.53 (d, J = 7.8 Hz, 1H), 7.43 – 7.37 (m, 4H), 7.21 (d, J = 7.8 Hz, 1H), 6.56 (dd, J = 20.4, 14.6 Hz, 1H), 6.13 (dd, J = 14.6, 3.6 Hz, 1H), 5.87 (dd, J = 20.4, 3.6 Hz, 1H), 2.42 (s, 3H), 0.56 (s, 6H); ^{13}C NMR (101 MHz, $CDCl_3$) δ 140.6, 138.1, 137.4, 135.4, 132.6, 132.6, 131.2, 129.9, 128.4, 128.1, 125.6, 123.8, 91.5, 91.3, 21.6, -2.6; IR (KBr): 3730, 3594, 3179, 2920, 2313, 1735, 1378, 823 cm^{-1}; HRMS-ESI (m/z): $[M+H]^+$ calcd. for $C_{19}H_{21}Si$, 277.1407; found 277.1415.

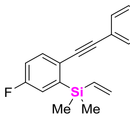
1o

(5-Fluoro-2-(phenylethynyl)phenyl)dimethyl(vinyl)silane 1o. By using the general procedure **A**, the title compound was prepared from 2-bromo-4-fluoro-1-(phenylethynyl)benzene (1.1 g, 4.0 mmol) and chlorodimethyl(vinyl)silane (528.0 mg, 4.4 mmol) and was purified by column chromatography to give pale yellow liquid (952 mg, 85%); TCL (petroleum ether): R_f = 0.94; ^{1}H NMR (400 MHz, $CDCl_3$) δ 7.62 – 7.55 (m, 3H), 7.40 (d, J = 5.5 Hz, 3H), 7.27 (t, J = 7.7 Hz, 1H), 7.07 (t, J = 8.5 Hz, 1H), 6.51 (dd, J = 20.3, 14.7 Hz, 1H), 6.15 (dd, J = 14.6, 2.9 Hz, 1H), 5.88 (dd, J = 20.4, 3.2 Hz, 1H), 0.56 (s, 6H); ^{13}C NMR (101 MHz, $CDCl_3$) δ 162.1 (d, J = 243.4 Hz, $^1J_{CF}$), 144.2 (d, J = 4.0 Hz, $^3J_{CF}$), 137.1, 134.6 (d, J = 7.0 Hz, $^3J_{CF}$), 133.3, 131.6, 131.2, 128.5, 128.4, 123.4, 121.4 (d, J = 19.2 Hz, $^2J_{CF}$), 116.1 (d, J = 22.2 Hz, $^2J_{CF}$), 91.7, 90.2, -2.9; IR (KBr): 3730, 3627, 3226, 2955, 1458, 1369, 1062, 828 cm^{-1}; HRMS-ESI (m/z): $[M+H]^+$ calcd. for $C_{18}H_{18}FSi$, 281.1156; found 281.1165.

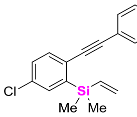
1p

(5-Chloro-2-(phenylethynyl)phenyl)dimethyl(vinyl)silane 1p. By using the general procedure **A**, the title compound was prepared from 2-bromo-4-chloro-1-(phenylethynyl)benzene (1.16 g, 4.0 mmol) and chlorodimethyl(vinyl)silane (528.0 mg, 4.4 mmol) and was purified by column chromatography to give colorless liquid (722 mg, 61%); TCL (petroleum ether): R_f = 0.92; ^{1}H NMR (400 MHz, $CDCl_3$) δ 7.57 – 7.53 (m,3H), 7.51 (s, 1H), 7.43 – 7.39 (m, 3H), 7.35 (dd, J = 8.2, 2.2 Hz, 1H), 6.50 (dd, J = 20.4, 14.7 Hz, 1H), 6.15 (dd, J = 14.7, 3.5 Hz, 1H), 5.87 (dd, J = 20.4, 3.5 Hz, 1H), 0.55 (s, 6H); ^{13}C NMR (101 MHz, $CDCl_3$) δ 143.4, 137.0, 134.5, 134.0, 133.9, 133.4, 131.3, 129.1, 128.5, 126.9, 123.2, 93.0, 90.1, -2.9; IR (KBr): 3731, 3684, 3162, 2960, 2322, 1955, 1734, 1399, 1101, 510 cm^{-1}; HRMS-ESI (m/z): $[M+H]^+$ calcd. for $C_{18}H_{18}ClSi$, 297.0861; found 297.0869.

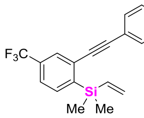
1q

Dimethyl(2-(phenylethynyl)-4-(trifluoromethyl)phenyl)(vinyl)silane 1q. By using the general procedure **A**, the title compound was prepared from 1-bromo-2-(phenylethynyl)-4-(trifluoromethyl)benzene (1.3 g, 4.0 mmol) and chlorodimethyl(vinyl)silane (528.0 mg, 4.4 mmol) and was purified by column chromatography to give colorless liquid (884 mg, 67%); TCL (petroleum ether): R_f = 0.90; ^{1}H NMR (500 MHz, $CDCl_3$) δ 7.80 (s, 1H), 7.65 (d, J = 7.8 Hz, 1H), 7.54 – 7.52 (m, 3H), 7.40 – 7.36 (m, 3H), 6.47 (dd, J = 20.4, 14.7 Hz, 1H), 6.11 (dd, J = 14.7, 3.4 Hz, 1H), 5.83 (dd, J = 20.4, 3.5 Hz, 1H), 0.52 (s, 6H); ^{13}C NMR (126 MHz, $CDCl_3$) δ 145.4, 136.9, 135.1, 133.5, 131.4, 131.3 (q, J = 32.8 Hz, $^2J_{CF}$), 129.5, 128.9 (q, J = 3.8 Hz, $^3J_{CF}$), 128.8, 128.6, 123.8 (d, J = 272.2 Hz, $^1J_{CF}$), 123.6 (q, J = 3.8 Hz, $^3J_{CF}$), 122.9, 93.5, 89.8, -2.9; IR (KBr): 3729, 3625, 3161, 2824, 2374, 2317. 1400, 1100, 571 cm^{-1}; HRMS-ESI (m/z): $[M+H]^+$ calcd. for $C_{19}H_{18}F_3Si$, 331.1124; found 331.1129.

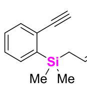
4

Allyl(2-ethynylphenyl) dimethylsilane 4. By using the general procedure **B**, The title compound was prepared from ((2-bromophenyl)ethynyl)trimethylsilane (1.26 g, 5.0

mmol) and chlorodimethyl(vinyl)silane (737.0 mg, 5.5 mmol) and was purified by column chromatography to give yellow liquid (970 mg, 97%); TCL (petroleum ether): R_f = 0.98; ^{1}H NMR (500 MHz, $CDCl_3$) δ 7.59 – 7.56 (m, 1H), 7.54 – 7.50 (m, 1H), 7.38 – 7.34 (m, 2H), 5.88 – 5.79 (m, 1H), 4.95 – 4.87 (m, 2H), 3.29 (s, 1H), 2.03 (d, J = 8.1 Hz, 2H), 0.43 (s, 6H); ^{13}C NMR (101 MHz, $CDCl_3$) δ 141.3, 134.9, 134.4, 133.5, 128.9, 128.0, 127.4, 113.5, 85.2, 80.5, 22.9, -3.2; IR (KBr): 3663, 1790, 1517, 1266, 894, 742 cm^{-1}; HRMS-ESI (m/z): $[M+H]^+$ calcd. for $C_{13}H_{17}Si$, 201.1094; found 201.0743.

4. The procedure for the preparation of sila-enyne **1r**

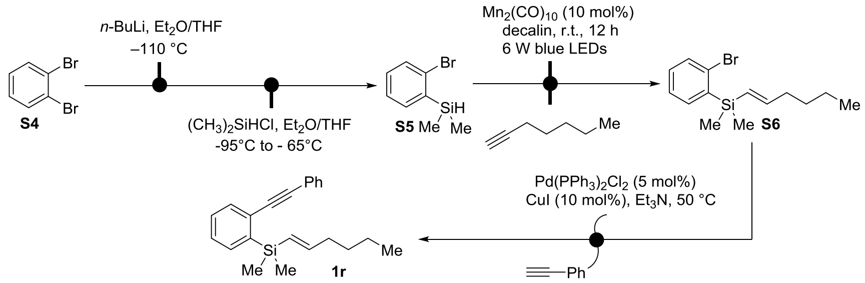

Step 1. A 100 mL oven-dried flask equipped with a magnetic stir bar and a septum was evacuated and backfilled with Ar 5 times. A solution of 1,2-dibromoarene (10 ~ 20 mmol) in 3/1 mixture of Et_2O/THF (80 ~ 100 mL) was injected via syringe into the flask. Solution was stirred at - 110 ℃ for 10 - 15 minutes. Solution of *n*-BuLi (1.6 M in hexane, 1.2 equiv) was cooled to - 90 ℃ in a separate vial and was dropwise added via syringe to the solution of 1,2-dibromoarene in 10 minutes. It is important to add *n*-BuLi solution by the wall of flask as opposed to adding it into reaction solution directly. Mixture was warmed up to -95 ℃ and kept at this temperature for 15 ~ 20 minutes. A cooled solution (- 95 ℃) of dimethyl silyl chloride (15 equiv) in 3/1 mixture of Et_2O/THF (10 ~ 20 mL) was rapidly added into the solution *via* syringe. Reaction mixture was warmed to -65 ℃ (approximately 1 h) and then quenched dropwise with water (20 mL). The crude mixture was extracted with ethyl acetate (3 × 20 mL) and the volatiles were evaporated. Residue was subjected to flash chromatography on silica gel to afford the product **S5**. [3]

Note: This procedure is extremely sensitive to temperature and time. If temperature does not reach -95 ℃ or if it is kept at -95 ℃ less than 15 ~ 20 minutes, the reaction will be incomplete and the separation of starting material and product is very difficult.

Step 2. Alkyne (0.2 mmol, 1.0 equiv), hydrosilane (0.6 mmol, 3.0 equiv), and $Mn_2(CO)_{10}$ (0.1 mmol, 0.1 equiv) were placed in a dry 10 mL Schlenk tube under a Ar atmosphere. Then anhydrous decalin (2.0 mL) was added with a syringe. The reaction mixture was stirred and irradiated by using 6 W blue LEDs at room temperature for 12 h. After completion of the reaction, the solvent was removed under reduced pressure and the residue was purified by flash column chromatography on silica gel to afford the product **S6**.[4]

Step 3. A Schlenk flask was charged with aryl bromide **S6** (1.0 eq.), $Pd(PPh_3)_2Cl_2$ (5 mol %) and CuI (10 mol%) under the atmosphere of Ar. The flask was vacuumed and refilled with Ar for three cycles. Degassed Et_3N and alkyne (1.1 eq.) were then added. The resulting solution was stirred at 60 ℃ overnight. The reaction was quenched by addition of aqueous saturated NH_4Cl, the phases were separated and the aqueous phase was extracted with Et_2O. The combined organics were washed with brine, dried over Na_2SO_4, and concentrated in vacuo. The residue was

chromatographed on silica gel with PE to provide the corresponding product **1r**.[5]

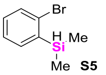

(2-Bromophenyl)dimethylsilane S5. The product was obtained from 1,2-dibromobenzene (1.3 g, 5.7 mmol) and chlorodimethylsilane (8.1 g, 85.8 mmol) and was purified by column chromatography to give colorless liquid (1.605 g, 75%); TCL (petroleum ether): R_f = 0.98; ^{1}H NMR (400 MHz, $CDCl_3$) δ 7.57 (dd, J = 7.8, 0.9 Hz, 1H), 7.50 (dd, J = 7.2, 1.9 Hz, 1H), 7.32 (td, J = 7.3, 1.1 Hz, 1H), 7.26 (td, J = 7.6, 1.9 Hz, 1H), 4.64 – 4.47 (m, 1H), 0.47 (d, J = 3.8 Hz, 6H); ^{13}C NMR (101 MHz, $CDCl_3$) δ 139.3, 136.6, 132.4, 131.1, 130.8, 126.5, -3.6.

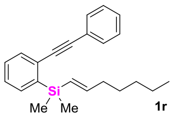

(*E*)-Hex-1-en-1-yldimethyl(2-(phenylethynyl)phenyl)silane 1r. The product was obtained from (*E*)-(2-bromophenyl)(hex-1-en-1-yl) dimethylsilane (177.6 mg, 0.6 mmol) and ethynylbenzene (71.4 mg, 0.7 mmol) and was purified by column chromatography to give yellow liquid (40 mg, 20%); TCL (petroleum ether): R_f = 0.96; ^{1}H NMR (400 MHz, $CDCl_3$) δ 7.59 (d, J = 6.9 Hz, 1H), 7.56 – 7.49 (m, 3H), 7.38 – 7.23 (m, 5H), 6.47 – 6.36 (m, 1H), 5.76 (d, J = 13.9 Hz, 1H), 2.04 (q, J = 7.3 Hz, 2H), 1.28 (dd, J = 14.2, 6.8 Hz, 2H), 1.24 – 1.11 (m, 4H), 0.82 (t, J = 6.6 Hz, 3H), 0.52 (s, 6H); ^{13}C NMR (101 MHz, $CDCl_3$) δ 151.1, 142.7, 135.3, 133.3, 132.1, 129.6, 129.4, 129.2, 128.9, 128.2, 127.8, 124.5, 93.0, 92.1, 34.6, 32.3, 30.0, 23.3, 14.8, 0.0; IR (KBr): 3752, 2956, 2923, 2854, 1461, 550, 430 cm^{-1}; HRMS-ESI (m/z): $[M+H]^+$ calcd. for $C_{23}H_{29}Si$ 333.2023, found 333.2028.

5. The procedure for the preparation of vinylether **1u** [6]

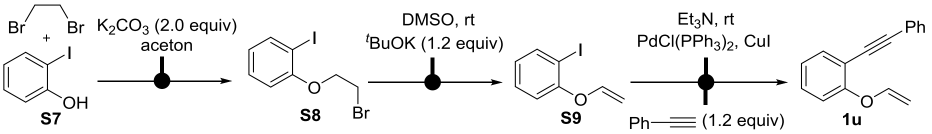

Step 1. To a solution of 2-iodophenol **S7** (5.0 mmol) and 1,2-dibromoethane (5.0 equiv) in acetone (50 mL) was added K_2CO_3 (2.0 equiv). The resulting mixture was stirred at room temperature for 24 h and then reflux for 12 h. The reaction was quenched with water and extracted with CH_2Cl_2. The organic layer was washed with brine, dried over Na_2SO_4, and concentrated. The crude product was purified by a silica gel column chromatography (Petroleum ether/EtOAc = 100:1) to give **S8** as a colorless oil.

Step 2. To a solution of **S8** (4.0 mmol) in DMSO (20 mL) was added KO*t*Bu (1.5 equiv). The resulting mixture was stirred at room temperature for 2 h. The reaction mixture was filtered and washed with CH_2Cl_2. The combined filtrate was concentrated and the residue was purified by a silica gel column chromatography (Petroleum ether/EtOAc = 100:1) to give **S9** as a yellow oil.

Step 3. To a solution of **S9** (1.0 mmol) and alkyne (1.1 equiv) in triethylamine (10 mL) was added $PdCl_2(PPh_3)_2$ (5 mol %) and CuI (5 mol %). The resulting mixture was stirred at room temperature for 24~36 h. The reaction mixture was filtered and washed with Et_2O. The combined filtrate was concentrated and the residue was purified by a silica gel column chromatography (Petroleum ether/EtOAc = 100:1) to give **1u** as a yellow oil.[6]

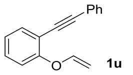

1-(Phenylethynyl)-2-(vinyloxy)benzene 1u. The product was obtained from 1-iodo-2-(vinyloxy)benzene (2.46 g, 10.0 mmol) and ethynylbenzene (1.22 g, 12.0 mmol) and was purified by column chromatography to give yellow liquid (1.54 g, 70%); TCL (petroleum ether): R_f = 0.50; ^{1}H NMR (400 MHz, $CDCl_3$) δ 7.55 – 7.51 (m, 3H), 7.32 (d, J = 5.7

Hz, 3H), 7.28 (d, J = 8.1 Hz, 1H), 7.07 (t, J = 7.5 Hz, 1H), 7.02 (d, J = 8.2 Hz, 1H), 6.68 (dd, J = 13.7, 6.1 Hz, 1H), 4.78 (d, J = 13.7 Hz, 1H), 4.46 (d, J = 6.1 Hz, 1H); ^{13}C NMR (101 MHz, $CDCl_3$) δ 157.1, 148.7, 133.6, 131.7, 129.7, 128.3, 123.4, 123.4, 117.3, 114.7, 95.0, 94.2, 85.1.

6. The procedure for the preparation of sulfonylhydrazine **10** [7]

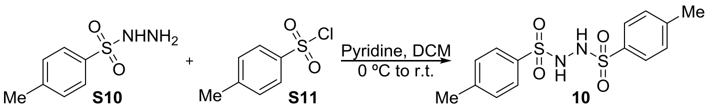

Tosyl chloride **S11** (2.375 g, 12.5 mmol) was added to tosylhydroazine **S10** (1.86 g, 10 mmol) in dichloromethane (30 mL) in an ice bath. Pyridine (1.44 mL, 18 mmol) was added dropwise in a temperature range from 0 to 10 ℃. TLC analysis was done to monitor the reaction. A mixture of water (30 mL) and hexane (30 mL) was added to the solution and stirred vigorously for 30 min. The solution was then suction filtered and washed with a 1:1 ratio of acetone and water (100 mL). The crystals were then added to acetone (30 mL), and the solution was boiled at 80 ℃. Water (15 mL) was added while stirring, and the solution was placed in an ice bath for an hour. The solution was then suction filtered and washed with cold diethyl ether to obtain a white solid.

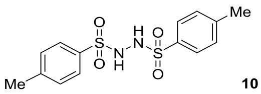

10

4-Methyl-N'-tosylbenzenesulfonohydrazide 10. The product was obtained as white solid (2.55 g, 75%)[7]; TCL (petroleum ether): R_f = 0.20; ^{1}H NMR (400 MHz, DMSO) δ 9.59 (s, 2H), 7.65 (d, J = 7.7 Hz, 4H), 7.39 (d, J = 7.6 Hz, 4H), 2.39 (s, 6H); ^{13}C NMR (126 MHz, DMSO) δ 144.0, 135.9, 129.9, 128.2, 21.5.

III. Optimization of the reaction conditions

Supplementary Table 1. The effect of photocatalysts on the cycloaromatization of sila-enyne **1a** with arylsulfonylazides **2a**[a]

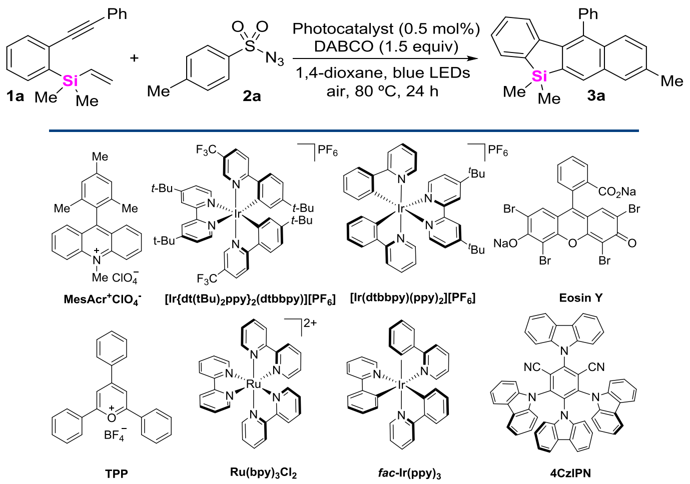

entry	photocatalyst	yield (%)[b]
1	$MesAcr^+ClO_4^-$ (**PC3**)	<5
2	$[Ir\{dt(tBu)_2ppy\}_2(dtbbpy)][PF_6]$ (**PC1**)	69
3	$[Ir(dtbbpy)(ppy)_2][PF_6]$ (**PC5**)	15
4	Eosin Y	<5
5	TPP	0
6	4CzIPN	trace
7	$Ru(bpy)_3Cl_2$ (**PC4**)	0
8	*fac*-$Ir(ppy)_3$ (**PC2**)	0

[a]All the reactions were performed using vinylsilane **1a** (0.20 mmol), TsN_3 (4.0 equiv), and DABCO (1.5 equiv) with photocatalysts (0.5 mol %) in 1,4-dioxane (2 mL) at 80 ˚C for 24 h under air in a sealed tube, followed by flash chromatography on SiO_2; [b]Isolated yield.

Supplementary Table 2. The effect of bases on the cycloaromatization of sila-enyne **1a** with arylsulfonylazides **2a**[a]

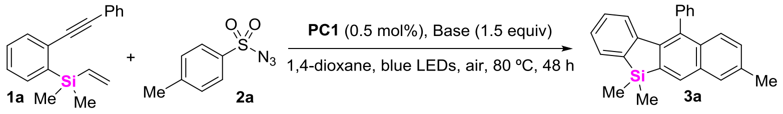

entry	base	yield (%)[b]
1	K_2CO_3	40
2	Cs_2CO_3	33
3	*i*Pr_2NEt	15
4	Pyridine	0
5	K_3PO_4	20
6	Na_2CO_3	30
7	Li_2CO_3	15
8	CsF	25

[a]All the reactions were performed using vinylsilane **1a** (0.20 mmol), TsN_3 (4.0 equiv), and base (1.5 equiv) with **PC1** (0.5 mol %) in 1,4-dioxane (2 mL) at 80 ˚C for 24 h under air in a sealed tube, followed by flash chromatography on SiO_2; [b]Isolated yield.

Supplementary Table 3. The effect of solvents on the cycloaromatization of sila-enyne **1a** with arylsulfonylazides **2a**[a]

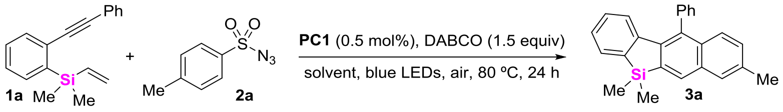

entry	base	yield (%)[b]
1	CH_3CN	trace
2	CH_2Cl_2	0
3	DCE	trace
4	AcOEt	40
5	Toluene	50
6	DMSO	0
7	DMF	0
8	DMA	0
9	HIFP	0
10	Benzotrifluoride	44
11	MeOH	0
12	TFE	0
13	C_6F_6	0

[a]All the reactions were performed using vinylsilane **1a** (0.20 mmol), TsN_3 (4.0 equiv), and DABCO (1.5 equiv) with **PC1** (0.5 mol %) in solvent (2 mL) at 80 ˚C for 24 h under air in a sealed tube, followed by flash chromatography on SiO_2; [b]Isolated yield.

Supplementary Table 4. The effect of the temperature on the cycloaromatization of sila-enyne **1a** with arylsulfonylazides **2a**[a]

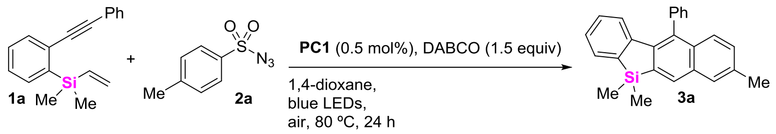

entry	temperature	yield (%)[b]
1	r.t.	38
2	60 ℃	65
3	80 ℃	69

[a]All the reactions were performed using vinylsilane **1a** (0.20 mmol), TsN_3 (4.0 equiv), and DABCO (1.5 equiv) with **PC1** (0.5 mol %) in 1,4-dioxane (2 mL) at x ˚C for 24 h under air in a sealed tube, followed by flash chromatography on SiO_2; [b]Isolated yield.

Supplementary Table 5. The effect of the dosage of arylsulfonylazides on the cycloaromatization of sila-enyne **1a** with arylsulfonylazides **2a**[a]

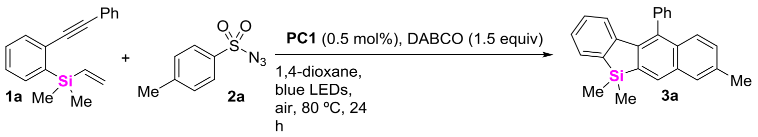

entry	**2a** (x equiv)	yield (%)[b]
1	6	38
2	7	35
3	8	40

[a]All the reactions were performed using vinylsilane **1a** (0.20 mmol), TsN_3 and DABCO (1.5 equiv) with **PC1** (0.5 mol %) in 1,4-dioxane (2 mL) at 80 ˚C for 24 h under air in a sealed tube, followed by flash chromatography on SiO_2; [b]Isolated yield.

Supplementary Table 6. Control experiments [a]

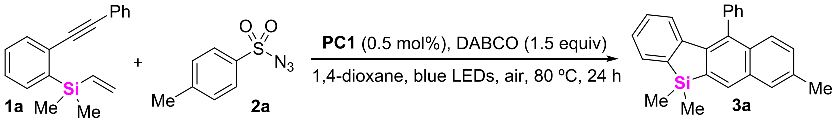

entry	changes to standard conditions	yield (%)[b]
1	none	69
2	no photocatalyst	0
3	no base	0
4	no light	0
5	green LEDs	20
6	Ar instead of air	34

[a]All the reactions were performed using vinylsilane **1a** (0.20 mmol), TsN_3 (4.0 mmol), and DABCO (1.5 equiv) with **PC1** (0.5 mol %) in 1,4-dioxane (2 mL) at 80 ˚C for 24 h under air in a sealed tube, followed by flash chromatography on SiO_2; [b]Isolated yield.

IV. General Procedure and Characterization Data of the Products

1. Photocatalyzed cycloaromatization of vinylsilanes **1** with arylsulfonylazides **2a**

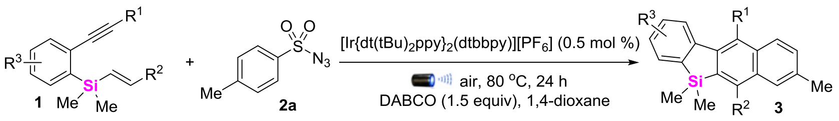

To a 10 mL vial equipped with a magnetic stir bar, was added vinylsilanes **1** (0.2 mmol, 1.0 equiv),

arylsulfonylazides **2a** (0.8 mmol, 4.0 equiv), DABCO (33 mg, 0.3 mmol, 1.5 equiv), [Ir{dt(tBu)$_2$ppy}$_2$(dtbbpy)][PF$_6$] **PC1** (1.3 mg, 0.5 mol %) and 1,4-dioxane (2.0 mL) under air. The vial was equipped with a Teflon septum and stirred at 80 ℃ under blue LED irradiation with two Kessil LEDs (30 W, 456 nm, approximately 3 cm away from the reaction mixture) for 24 hours. The solvent was removed *in vacuo* and the residue was purified by flash column chromatography on silica gel to yield the products **3**.

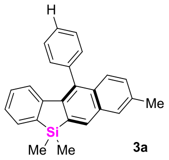

3a

5,5,8-Trimethyl-11-phenyl-5H-benzo[b]naphtho[2,3-d]silole 3a. The product was obtained from dimethyl(2-(phenylethynyl)phenyl)(vinyl)silane **1a** (52.4 mg, 0.20 mmol) and 4-methylbenzenesulfonyl azide **2a** (157.6 mg, 4.0 equiv) and was purified by column chromatography to give white solid (48 mg, 69%); mp: 159.2 – 161.5 ^{o}C; TCL (petroleum ether): R$_f$ = 0.85; ^{1}H NMR (400 MHz, CDCl$_3$) δ 8.06 (s, 1H), 7.62 – 7.60 (m, 2H), 7.58 – 7.52 (m, 3H), 7.36 – 7.34 (m, 2H), 7.27 (d, J = 8.7 Hz, 1H), 7.17 – 7.11 (m, 2H), 7.00 – 6.96 (m, 1H), 6.50 (d, J = 8.2 Hz, 1H), 2.49 (s, 3H), 0.49 (s, 6H); ^{13}C NMR (126 MHz, CDCl$_3$) δ 148.8, 141.0, 140.9, 140.3, 138.4, 135.4, 135.0, 133.4, 132.7, 132.6, 132.6, 130.2, 129.7, 129.3, 128.6, 127.5, 126.9, 126.9, 126.6, 126.3, 21.5, -2.5; IR (KBr): 3729, 3610, 3188, 3062, 1538, 1106, 740 cm^{-1}; HRMS-ESI (m/z): [M+H]$^+$ calcd. for $C_{25}H_{23}Si$, 351.1564; found 351.1571.

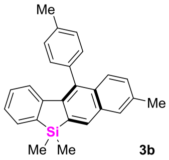

3b

5,5,8-Trimethyl-11-(p-tolyl)-5H-benzo[b]naphtho[2,3-d]silole 3b. The product was obtained from dimethyl(2-(*p*-tolylethynyl)phenyl)(vinyl)silane **1b** (55.2 mg, 0.20 mmol) and 4-methylbenzenesulfonyl azide **2a** (157.6 mg, 4.0 equiv) and was purified by column chromatography to give white solid (44 mg, 60%); mp: 138.0 – 140.7 ℃; TCL (petroleum ether): R$_f$ = 0.86; ^{1}H NMR (400 MHz, CDCl$_3$) δ 8.04 (s, 1H), 7.61 (s, 2H), 7.36 (d, J = 6.5 Hz, 2H), 7.29 (d, J = 7.9 Hz, 1H), 7.23 (d, J = 7.2 Hz, 2H), 7.13 (dd, J = 12.3, 7.2 Hz, 2H), 7.00 (t, J = 6.8 Hz, 1H), 6.60 (d, J = 7.6 Hz, 1H), 2.52 (s, 3H), 2.47 (s, 3H), 0.48 (s, 6 H); ^{13}C NMR (101 MHz, CDCl$_3$) δ 149.0, 141.0, 140.4, 138.3, 137.7, 137.1, 135.3, 135.0, 133.6, 132.7, 132.5, 132.4, 130.0, 130.0, 129.7, 128.5, 127.0, 126.8, 126.5, 126.4, 21.5, 21.5, -2.5; IR (KBr): 3778, 3699, 2920, 1561, 1442, 912, 780 cm^{-1}; HRMS-ESI (m/z): [M+H]$^+$ calcd. for $C_{26}H_{25}Si$, 365.1720; found 365.1726.

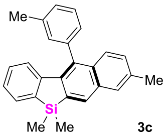

3c

5,5,8-Trimethyl-11-(m-tolyl)-5H-benzo[b]naphtho[2,3-d]silole 3c. The product was obtained from dimethyl(2-(m-tolylethynyl)phenyl)(vinyl)silane **1c** (55.2 mg, 0.20 mmol) and 4-methylbenzenesulfonyl azide **2a** (157.6 mg, 4.0 equiv) and was purified by column chromatography to give white solid (46 mg, 63%); mp: 131.7 – 138.4 ℃; TCL (petroleum ether): R$_f$ = 0.85; ^{1}H NMR (400 MHz, CDCl$_3$) δ 8.05 (s, 1H), 7.61 (s, 2H), 7.45 (t, J = 7.6 Hz, 1H), 7.31 (dd, J = 16.1, 8.1 Hz, 2H), 7.16 – 7.12 (m, 4H), 7.00 (t, J = 7.7 Hz, 1H), 6.55 (d, J = 8.2 Hz, 1H), 2.48 (s, 3H), 2.42 (s, 3H), 0.49 (s, 6H); ^{13}C NMR (101 MHz, CDCl$_3$) δ 148.9, 141.0, 140.7, 140.2, 138.9, 138.3, 135.4, 135.2, 133.4, 132.7, 132.6, 132.4, 130.7, 129.7, 129.1, 128.6, 128.2, 127.1, 127.0, 126.8, 126.6, 126.4, 21.6, 21.5, -2.5; IR (KBr): 3729, 3182, 3047, 2823, 2318, 1372, 1095 cm^{-1}. HRMS-ESI (m/z): [M+H]$^+$ calcd. for $C_{26}H_{25}Si$, 365.1720; found 365.1723.

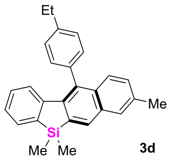

3d

11-(4-Ethylphenyl)-5,5,8-trimethyl-5H-benzo[b]naphtho[2,3-d]silole 3d. The product was obtained from (2-((4-ethylphenyl)ethynyl)phenyl)dimethyl(vinyl)silane **1d** (58.0 mg, 0.20 mmol) and 4-methylbenzenesulfonyl azide **2a** (157.6 mg, 4.0 equiv) and was purified by column chromatography to give white solid (50 mg, 66%); mp: 104.9 – 107.1 ℃; TCL (petroleum ether): R$_f$ = 0.84; ^{1}H NMR

(500 MHz, $CDCl_3$) δ 8.04 (d, J = 6.3 Hz, 1H), 7.60 (d, J = 5.5 Hz, 2H), 7.38 (d, J = 7.8 Hz, 2H), 7.31 (t, J = 7.8 Hz, 1H), 7.24 (dd, J = 9.8, 4.7 Hz, 2H), 7.17 – 7.09 (m, 2H), 7.01 – 6.95 (m, 1H), 6.53 (t, J = 8.0 Hz, 1H), 2.84 – 2.80 (m, 2H), 2.47 (d, J = 4.0 Hz, 3H), 1.3940 – 1.35 (m, 3H), 0.48 – 0.47 (m, 6H); ^{13}C NMR (126 MHz, $CDCl_3$) δ 149.0, 143.5, 141.0, 140.4, 138.3, 137.9, 135.3, 135.1, 133.6, 132.7, 132.6, 132.4, 130.0, 129.7, 128.8, 128.6, 127.0, 126.8, 126.5, 126.4, 28.9, 21.5, 15.8, -2.5; IR (KBr): 3652, 1402, 1266, 741 cm^{-1}; HRMS-ESI (m/z): $[M+H]^+$ calcd. for $C_{27}H_{27}Si$, 379.1877; found 379.1883.

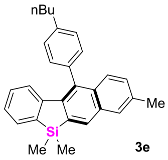

3e

11-(4-Butylphenyl)-5,5,8-trimethyl-5H-benzo[b]naphtho[2,3-d]silole 3e. The product was obtained from (2-((4-butylphenyl)ethynyl)phenyl)dimethyl(vinyl)silane **1e** (63.6 mg, 0.20 mmol) and 4-methylbenzenesulfonyl azide **2a** (157.6 mg, 4.0 equiv) and was purified by column chromatography to give white solid (53 mg, 65%); mp: 76.1 – 77.5 ℃; TCL (petroleum ether): R_f = 0.84; ^{1}H NMR (500 MHz, $CDCl_3$) δ 8.05 (s, 1H), 7.61 – 7.59 (m, 2H), 7.36 (d, J = 7.9 Hz, 2H), 7.33 (d, J = 8.7 Hz, 1H), 7.24 (d, J = 7.9 Hz, 2H), 7.16 (dd, J = 8.7, 1.6 Hz, 1H), 7.11 (dd, J = 10.6, 3.8 Hz, 1H), 6.98 – 6.94 (m, 1H), 6.50 (d, J = 8.2 Hz, 1H), 2.78 (t, J = 7.6 Hz, 2H), 2.47 (s, 3H), 1.78 – 1.72 (m, 2H), 1.46 (dd, J = 14.9, 7.4 Hz, 2H), 1.01 (t, J = 7.4 Hz, 3H), 0.48 (s, 6H); ^{13}C NMR (126 MHz, $CDCl_3$) δ 149.0, 142.1, 141.0, 140.5, 138.3, 137.9, 135.4, 135.2, 133.6, 132.7, 132.5, 132.4, 129.9, 129.6, 129.4, 128.6, 127.0, 126.8, 126.5, 126.4, 35.6, 33.8, 22.4, 21.5, 14.2, -2.5; IR (KBr): 3832, 3730, 3613, 3182, 2373, 2316, 1539, 1402, 1105, 500 cm^{-1}; HRMS-ESI (m/z): $[M+H]^+$ calcd. for $C_{29}H_{31}Si$, 407.2190; found 407.2198.

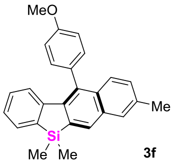

3f

11-(4-Methoxyphenyl)-5,5,8-trimethyl-5H-benzo[b]naphtho[2,3-d]silole 3f. The product was obtained from (2-((4-methoxyphenyl)ethynyl)phenyl)dimethyl(vinyl)silane **1f** (58.4 mg, 0.20 mmol) and 4-methylbenzenesulfonyl azide **2a** (157.6 mg, 4.0 equiv) and was purified by column chromatography to give white solid (52 mg, 68%); mp: 185.5 – 186.3 ℃; TCL (petroleum ether): R_f = 0.85; ^{1}H NMR (500 MHz, $CDCl_3$) δ 8.05 (s, 1H), 7.61 (s, 2H), 7.32 (d, J = 8.6 Hz, 1H), 7.25 (d, J = 7.7 Hz, 2H), 7.16 (t, J = 5.0 Hz, 2H), 7.11 (d, J = 7.7 Hz, 2H), 7.03 (t, J = 7.7 Hz, 1H), 6.63 (d, J = 8.1 Hz, 1H), 3.94 (s, 3H), 2.48 (s, 3H), 0.49 (s, 6H); ^{13}C NMR (126 MHz, $CDCl_3$) δ 159.1, 149.0, 141.0, 140.6, 138.3, 135.3, 134.7, 133.8, 132.9, 132.7, 132.6, 132.5, 131.1, 129.8, 128.6, 126.9, 126.8, 126.6, 126.3, 114.7, 55.4, 21.5, -2.5; IR (KBr): 3450, 2920, 2847, 1605, 1511, 1439, 1246, 725 cm^{-1}; HRMS-ESI (m/z): $[M+H]^+$ calcd. for $C_{26}H_{25}OSi$, 381.1669; found 381.1675.

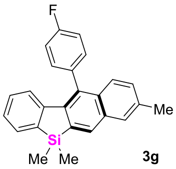

3g

11-(4-Fluorophenyl)-5,5,8-trimethyl-5H-benzo[b]naphtho[2,3-d]silole 3g. The product was obtained from (2-((4-fluorophenyl)ethynyl)phenyl)dimethyl(vinyl) silane **1g** (56.0 mg, 0.20 mmol) and 4-methylbenzenesulfonyl azide **2a** (157.6 mg, 4.0 equiv) and was purified by column chromatography to give white solid (37 mg, 50%); mp: 150.1 – 152.3 ℃; TCL (petroleum ether): R_f = 0.85; ^{1}H NMR (500 MHz, $CDCl_3$) δ 8.07 (s, 1H), 7.62 (s, 2H), 7.31 (t, J = 6.8 Hz, 2H), 7.27 (t, J = 9.4 Hz, 2H), 7.23 (s, 1H), 7.16 (dd, J = 15.0, 7.9 Hz, 2H), 7.03 (t, J = 7.7 Hz, 1H), 6.53 (d, J = 8.2 Hz, 1H), 2.49 (s, 3H), 0.49 (s, 6H); ^{13}C NMR (126 MHz, $CDCl_3$) δ 162.5 (d, J = 247.0 Hz, $^{1}J_{CF}$), 148.6, 141.1, 140.6, 138.4, 136.7 (d, J = 2.5 Hz, $^{3}J_{CF}$) 135.5, 133.7, 133.5, 132.8, 132.7 (d, J = 2.5 Hz, $^{3}J_{CF}$), 131.8, 131.7, 129.7, 128.8, 126.9, 126.7, 126.6, 126.1, 116.4 (d, J = 20.2 Hz, $^{2}J_{CF}$), 21.5, -2.6; IR (KBr): 3842, 3734, 3623, 3174, 3069, 1540, 1377, 1265, 1108, 742 cm^{-1}; HRMS-ESI (m/z): $[M+H]^+$ calcd. for $C_{25}H_{22}FSi$**,** 369.1469; found 369.1475.

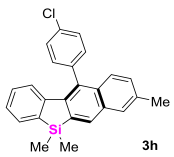
3h

11-(4-Chlorophenyl)-5,5,8-trimethyl-5H-benzo[b]naphtho[2,3-d]silole 3h. The product was obtained from (2-((4-chlorophenyl)ethynyl)phenyl)dimethyl(vinyl)silane **1h** (59.2 mg, 0.20 mmol) and 4-methylbenzenesulfonyl azide **2a** (157.6 mg, 4.0 equiv) and was purified by column chromatography to give white solid (50 mg, 65%); mp: 135.9 – 143.2 °C; TCL (petroleum ether): R_f = 0.82; ^{1}H NMR (500 MHz, $CDCl_3$) δ 8.07 (s, 1H), 7.62 (d, J = 6.0 Hz, 2H), 7.55 (d, J = 8.0 Hz, 2H), 7.29 (d, J = 8.0 Hz, 2H), 7.21 (d, J = 9.3 Hz, 1H), 7.17 (d, J = 9.0 Hz, 2H), 7.04 (t, J = 7.7 Hz, 1H), 6.59 (d, J = 8.1 Hz, 1H), 2.48 (s, 3H), 0.49 (s, 6H); ^{13}C NMR (126 MHz, $CDCl_3$) δ 148.5, 141.1, 140.4, 139.4, 138.4, 135.6, 133.5, 133.5, 133.2, 132.9, 132.7, 132.7, 131.7, 129.8, 129.6, 128.8, 127.0, 126.8, 126.5, 126.1, 21.5, -2.6; IR (KBr): 3727, 3198, 2970, 2823, 1372, 1102, 739 cm^{-1}; HRMS-ESI (m/z): $[M+H]^+$ calcd. for $C_{25}H_{22}ClSi$, 385.1174; found 385.1178.

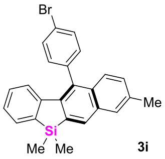
3i

11-(4-Bromophenyl)-5,5,8-trimethyl-5H-benzo[b]naphtho[2,3-d]silole 3i. The product was obtained from (2-((4-bromophenyl)ethynyl)phenyl)dimethyl(vinyl)silane **1i** (68.0 mg, 0.20 mmol) and 4-methylbenzenesulfonyl azide **2a** (157.6 mg, 4.0 equiv) and was purified by column chromatography to give white solid (60 mg, 70%); mp: 99.8 – 100.0 °C; TCL (petroleum ether): R_f = 0.82; ^{1}H NMR (500 MHz, $CDCl_3$) δ 8.07 (s, 1H), 7.71 (d, J = 7.9 Hz, 2H), 7.62 (d, J = 6.6 Hz, 2H), 7.24 (d, J = 6.7 Hz, 2H), 7.21 (d, J = 8.7 Hz, 1H), 7.18 – 7.15 (m, 2H), 7.05 (t, J = 7.7 Hz, 1H), 6.60 (d, J = 8.1 Hz, 1H), 2.48 (s, 3H), 0.49 (s, 6H); ^{13}C NMR (126 MHz, $CDCl_3$) δ 148.5, 141.1, 140.3, 139.9, 138.4, 135.6, 133.4, 133.1, 132.9, 132.7, 132.7, 132.5, 132.0, 129.8, 128.8, 126.9, 126.8, 126.5, 126.1, 121.7, 21.4, -2.6; IR (KBr): 2921, 1654, 1577, 1063, 710 cm^{-1}; HRMS-ESI (m/z): $[M+H]^+$ calcd. for $C_{25}H_{22}BrSi$, 429.0669; found 429.0668.

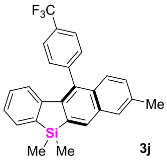
3j

5,5,8-Trimethyl-11-(4-(trifluoromethyl)phenyl)-5H-benzo[b]naphtho[2,3-d]silole 3j. The product was obtained from dimethyl(2-((4-(trifluoromethyl)phenyl)ethynyl)phenyl)(vinyl)silane **1j** (66.0 mg, 0.20 mmol) and 4-methylbenzenesulfonyl azide **2a** (157.6 mg, 4.0 equiv) and was purified by column chromatography to give white solid (60 mg, 71%); mp: 147.6 – 148.9 °C; TCL (petroleum ether): R_f = 0.80; ^{1}H NMR (400 MHz, $CDCl_3$) δ 8.16 (s, 1H), 7.91 (d, J = 7.6 Hz, 2H), 7.70 (s, 2H), 7.57 (d, J = 7.6 Hz, 2H), 7.25 – 7.05 (m, 3H), 7.07 (t, J = 7.5 Hz, 1H), 6.51 (d, J = 6.1 Hz, 1H), 2.55 (s, 3H), 0.57 (s, 6H); ^{13}C NMR (101 MHz, $CDCl_3$) δ 148.3, 145.0, 141.2, 140.3, 138.4, 135.7, 133.2 133.1, 132.9, 132.9, 132.7, 130.8, 129.9 (q, J = 32.3 Hz, $^2J_{CF}$), 129.8, 129.0, 127.0, 126.9, 126.4, 126.3 (q, J = 4.0 Hz, $^3J_{CF}$), 124.4 (d, J = 272.7 Hz, $^1J_{CF}$), 21.5, -2.6; IR (KBr): 2917, 1614, 1489, 848, 780, 681 cm^{-1}; HRMS-ESI (m/z): $[M+H]^+$ calcd. for $C_{26}H_{22}F_3Si$, 419.1437; found 419.1444.

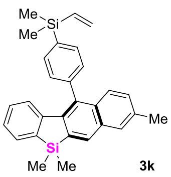
3k

11-(4-(Dimethyl(vinyl)silyl)phenyl)-5,5,8-trimethyl-5H-benzo[b]naphtho[2,3-d]silole 3k. The product was obtained from compund **1k** (69.2 mg, 0.20 mmol) and 4-methylbenzenesulfonyl azide **2a** (157.6 mg, 4.0 equiv) and was purified by column chromatography to give white solid (43 mg, 50%); mp: 138.3 – 140.5 °C; TCL (petroleum ether): R_f = 0.80; ^{1}H NMR (400 MHz, $CDCl_3$) δ 8.11 (s, 1H), 7.76 (d, J = 7.7 Hz, 2H), 7.65 (d, J = 8.0 Hz, 2H), 7.39 (d, J = 7.6 Hz, 2H), 7.34 (d, J = 8.6 Hz, 1H), 7.25 – 7.14 (m, 2H), 7.00 (t, J = 8.1 Hz, 1H), 6.53 – 6.40 (m, 2H), 6.18 (dd, J = 14.6, 3.7 Hz, 1H), 5.89 (dd, J = 20.3, 3.7 Hz, 1H), 2.53 (s, 3H), 0.53 (d, J = 8.4 Hz, 12H); ^{13}C NMR (101 MHz, $CDCl_3$) δ 148.8, 141.5, 140.9, 140.3, 138.3, 138.3, 137.3, 135.4, 134.9, 134.8, 133.3, 132.9, 132.7, 132.5, 132.5, 130.1, 129.6, 129.5, 129.3, 128.6, 127.0, 126.8, 126.5, 126.3, 21.5, -2.6, -2.7; IR

(KBr): 3650, 3218, 2943, 2323, 1789, 1371, 739 cm^{-1}; HRMS-ESI (m/z): $[M+H]^+$ calcd. for $C_{29}H_{31}Si_2$, 435.1959; found 435.1965.

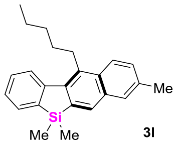
3l

5,5,8-Trimethyl-11-pentyl-5H-benzo[b]naphtho[2,3-d]silole 3l. The product was obtained from (2-(hept-1-yn-1-yl)phenyl)dimethyl(vinyl)silane **1l** (51.2 mg, 0.20 mmol) and 4-methylbenzenesulfonyl azide (157.6 mg, 4.0 equiv) and was purified by column chromatography to give colorless oil (34 mg, 50%); TCL (petroleum ether): R_f = 0.90; ^{1}H NMR (500 MHz, $CDCl_3$) δ 8.06 (d, J = 10.0 Hz, 1H), 8.01 (d, J = 8.1 Hz, 1H), 7.90 (s, 1H), 7.71 (d, J = 7.0 Hz, 1H), 7.58 (s, 1H), 7.52 – 7.49 (m, 1H), 7.37 (dd, J = 8.7, 1.5 Hz, 1H), 7.31 (t, J = 7.1 Hz, 1H), 3.44 – 3.41 (m, 2H), 2.51 (s, 3H), 1.96 – 1.89 (m, 2H), 1.71 – 1.65 (m, 2H), 1.54 – 1.49 (m, 2H), 1.02 (t, J = 7.3 Hz, 3H), 0.44 (s, 6H); ^{13}C NMR (126 MHz, $CDCl_3$) δ 149.5, 141.5, 140.7, 138.7, 135.1, 134.5, 133.1, 133.0, 132.8, 131.3, 130.2, 128.8, 127.6, 126.5, 126.2, 124.4, 32.4, 29.7, 29.6, 29.4, 22.6, 21.4, 14.3, -2.5; IR (KBr): 3396, 2923, 2854, 1591, 1462, 1442, 1245, 782 cm^{-1}; HRMS-ESI (m/z): $[M+H]^+$ calcd. for $C_{24}H_{29}Si$, 345.2033; found 345.2041.

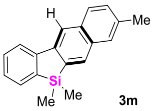
3m

5,5,8-Trimethyl-5H-benzo[b]naphtho[2,3-d]silole 3m. The product was obtained from (2-ethynylphenyl)dimethyl(vinyl)silane **1m** (37.2 mg, 0.20 mmol) and 4-methylbenzenesulfonyl azide **2a** (157.6 mg, 4.0 equiv) and was purified by column chromatography to give colorless oil (8 mg, 15%); TCL (petroleum ether): R_f = 0.88; ^{1}H NMR (400 MHz, $CDCl_3$) δ 8.21 (s, 1H), 8.05 (s, 1H), 8.03 (d, J = 7.8 Hz, 1H), 7.80 (d, J = 8.3 Hz, 1H), 7.70 (d, J = 7.0 Hz, 1H), 7.63 (s, 1H), 7.52 – 7.48 (m, 1H), 7.35 (dd, J = 3.3, 1.1 Hz, 1H), 7.34 (d, J = 2.5 Hz, 1H), 2.55 (s, 3H), 0.51 (s, 6H); ^{13}C NMR (101 MHz, $CDCl_3$) δ 147.9, 143.6, 139.5, 137.3, 135.4, 133.5, 133.1, 132.9, 132.8, 130.3, 128.9, 128.2, 127.4, 127.1, 121.3, 118.7, 21.7, -2.7; IR (KBr): 3713, 3651, 3612, 3564, 1716, 1489, 1388, 901 cm^{-1}; HRMS-ESI (m/z): $[M+H]^+$ calcd. for $C_{19}H_{19}Si$, 275.1251; found 275.1249.

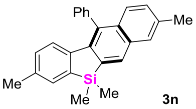
3n

3,5,5,8-Tetramethyl-11-phenyl-5H-benzo[b]naphtho[2,3-d]silole 3n. The product was obtained from dimethyl(5-methyl-2-(phenylethynyl)phenyl)(vinyl)silane **1n** (55.2 mg, 0.20 mmol) and 4-methylbenzenesulfonyl azide **2a** (157.6 mg, 4.0 equiv) and was purified by column chromatography to give pale yellow solid (50 mg, 68%); mp: 199.9 – 202.1 ℃; TCL (petroleum ether): R_f = 0.80; ^{1}H NMR (400 MHz, $CDCl_3$) δ 8.12 (s, 1H), 7.68 (s, 1H), 7.68 – 7.59 (m, 3H), 7.50 (s, 1H), 7.42 (d, J = 6.4 Hz, 2H), 7.34 (d, J = 8.6 Hz, 1H), 7.22 (d, J = 8.6 Hz, 1H), 6.88 (d, J = 8.3 Hz, 1H), 6.44 (d, J = 8.3 Hz, 1H), 2.55 (s, 3H), 2.35 (s, 3H), 0.56 (d, J = 1.3 Hz, 6H); ^{13}C NMR (101 MHz, $CDCl_3$) δ 146.1, 141.1, 140.9, 140.4, 138.4, 136.2, 135.2, 134.4, 133.4, 133.3, 132.5, 132.5, 130.7, 130.2, 129.3, 128.6, 127.5, 126.8, 126.8, 126.1, 21.5, 21.0, -2.5; IR (KBr): 3731, 3173, 1402, 1106, 741 cm^{-1}; HRMS-ESI (m/z): $[M+H]^+$ calcd. for $C_{26}H_{25}Si$, 365.1720; found 365.1730.

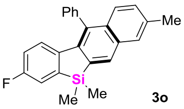
3o

3-Fluoro-5,5,8-trimethyl-11-phenyl-5H-benzo[b]naphtho[2,3-d]silole 3o. The product was obtained from (5-fluoro-2-(phenylethynyl)phenyl)dimethyl(vinyl) silane **1o** (56.0 mg, 0.20 mmol) and 4-methylbenzenesulfonyl azide **2a** (157.6 mg, 4.0 equiv) and was purified by column chromatography to give white solid (43 mg, 58%); mp: 145.0 – 146.8 ℃; TCL (petroleum ether): R_f = 0.82; ^{1}H NMR (400 MHz, $CDCl_3$) δ 8.04 (s, 1H), 7.61 (s, 1H), 7.57 – 7.52 (m, 3H), 7.33 (d, J = 7.5 Hz, 2H), 7.27 – 7.24 (m, 2H), 7.16 (d, J = 8.6 Hz, 1H), 6.67 – 6.62 (m, 1H), 6.43 (dd, J = 8.9, 4.8 Hz, 1H), 2.47 (s, 3H), 0.49 (s, 6H); ^{13}C NMR (101 MHz, $CDCl_3$) δ 161.8 (d, J = 251.5 Hz, $^1J_{CF}$), 144.60 (d, J = 3.0 Hz, $^3J_{CF}$), 144.1 (d, J = 5.1 Hz, $^3J_{CF}$), 140.6, 139.4, 137.9, 135.4, 134.5, 133.4, 132.7, 132.4, 130.1, 129.4, 128.8, 127.9,

127.8, 127.6, 126.8, 126.8, 118.6 (d, J = 19.2 Hz, $^{2}J_{CF}$), 116.4 (d, J = 22.2 Hz, $^{2}J_{CF}$), 21.4, -2.7; IR (KBr): 3458, 2924, 2855, 1578, 1443, 1267, 820, 780, 700 cm^{-1}; HRMS-ESI (m/z): $[M+H]^+$ calcd. for $C_{25}H_{22}FSi$, 369.1469; found 369.1470.

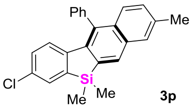

3-Chloro-5,5,8-trimethyl-11-phenyl-5H-benzo[b]naphtho[2,3-d]silole 3p. The product was obtained from (5-chloro-2-(phenylethynyl)phenyl)dimethyl(vinyl)silane **1p** (59.2 mg, 0.20 mmol) and 4-methylbenzenesulfonyl azide **2a** (157.6 mg, 4.0 equiv) and was purified by column chromatography to give pale yellow solid (46 mg, 60%); mp: 149.9 – 151.0 ℃; TCL (petroleum ether): R_f = 0.80; ^{1}H NMR (400 MHz, $CDCl_3$) δ 8.11 (s, 1H), 7.67 (s, 1H), 7.63 – 7.56 (m, 4H), 7.38 (d, J = 6.6 Hz, 2H), 7.32 (d, J = 8.6 Hz, 1H), 7.22 (d, J = 8.6 Hz, 1H), 6.98 (d, J = 8.6 Hz, 1H), 6.42 (d, J = 8.7 Hz, 1H), 2.53 (s, 3H), 0.55 (s, 6H); ^{13}C NMR (101 MHz, $CDCl_3$) δ 147.0, 143.5, 140.5, 139.3, 137.7, 135.7, 135.1, 133.3, 132.9, 132.7, 132.7, 132.2, 130.0, 129.6, 129.4, 128.9, 127.7, 127.4, 126.9, 126.9, 21.5, -2.7; IR (KBr): 3452, 2922, 2218, 1616, 1442, 1242, 909 cm^{-1}; HRMS-ESI (m/z): $[M+H]^+$ calcd. for $C_{25}H_{22}ClSi$, 385.1174; found: 385.1174.

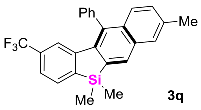

5,5,8-Trimethyl-11-phenyl-3-(trifluoromethyl)-5H-benzo[b]naphtho[2,3-d]silole 3q. The product was obtained from dimethyl(2-(phenylethynyl)-4-(trifluoromethyl)phenyl)(vinyl)silane (66.0 mg, 0.20 mmol) **1q** and 4-methylbenzenesulfonyl azide **2a** (157.6 mg, 4.0 equiv) and was purified by column chromatography to give white solid (54 mg, 65%); mp: 112.6 – 123.5 ℃; TCL (petroleum ether): R_f = 0.75; ^{1}H NMR (400 MHz, $CDCl_3$) δ 8.16 (s, 1H), 7.75 (d, J = 7.3 Hz, 1H), 7.71 (s, 1H), 7.68 – 7.61 (m, 3H), 7.43 (t, J = 9.2 Hz, 4H), 7.27 (d, J = 9.1 Hz, 1H), 6.74 (s, 1H), 2.56 (s, 3H), 0.58 (s, 6H); ^{13}C NMR (101 MHz, $CDCl_3$) δ 149.4, 145.3, 140.0, 139.1, 137.6, 136.0, 135.9, 133.2, 133.0, 132.8, 132.7, 131.6 (d, J = 31.3 Hz, $^{2}J_{CF}$), 129.8, 129.6, 129.0, 128.0, 127.1, 126.9, 126.5 (d, J = 209.1 Hz, $^{1}J_{CF}$), 122.9 (q, J = 4.0 Hz, $^{3}J_{CF}$), 122.7 (q, J = 3.0 Hz, $^{3}J_{CF}$), 21.5, -2.8; IR (KBr): 3182, 3051, 2326, 1374, 1266, 1098, 740 cm^{-1}; HRMS-ESI (m/z): $[M+H]^+$ calcd. for $C_{26}H_{22}F_3Si$, 419.1437; found 419.1443.

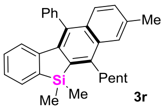

5,5,8-Trimethyl-6-pentyl-11-phenyl-5H-benzo[b]naphtho[2,3-d]silole 3r. The product was obtained from indoles **1r** (66.4 mg, 0.20 mmol) and 4-methylbenzenesulfonyl azide **2a** (157.6 mg, 4.0 equiv) and was purified by column chromatography to give colorless oil (8 mg, 12%); TCL (petroleum ether): R_f = 0.86; ^{1}H NMR (400 MHz, $CDCl_3$) δ 7.72 (s, 1H), 7.54 – 7.41 (m, 4H), 7.30 – 7.16 (m, 3H), 7.06 (dd, J = 15.2, 7.9 Hz, 2H), 6.88 (t, J = 7.7 Hz, 1H), 6.37 (d, J = 8.2 Hz, 1H), 3.19 – 3.05 (m, 2H), 2.45 (s, 3H), 1.81 – 1.67 (m, 2H), 1.52 (dd, J = 14.8, 7.6 Hz, 2H), 1.41 (dd, J = 14.5, 7.2 Hz, 2H), 0.92 (t, J = 7.2 Hz, 3H), 0.47 (s, 6H); ^{13}C NMR (101 MHz, $CDCl_3$) δ 148.8, 144.6, 141.3, 141.0, 140.0, 136.8, 135.1, 134.1, 133.8, 132.1, 130.8, 130.4, 129.5, 129.2, 128.1, 127.7, 127.4, 126.5, 126.3, 123.0, 36.1, 32.8, 31.9, 22.7, 21.9, 14.2, -2.0; IR (KBr): 3449, 1716, 1651, 1541, 1508, 1264, 956 cm^{-1}; HRMS-ESI (m/z): $[M+H]^+$ calcd. for $C_{25}H_{22}Si$, 421.2346; found 421.2342.

2. The coupling-cyclization of allylic dimethylsilanes **4** with arylsulfonyl azides **2**

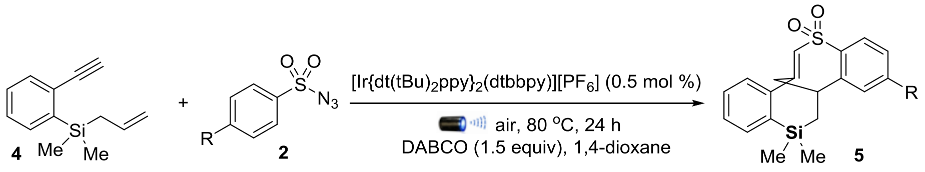

To a 10 mL vial equipped with a magnetic stir bar, was added vinylsilanes **4** (0.2 mmol, 1.0 equiv), arylsulfonylazides **2** (0.8 mmol, 4.0 equiv), DABCO (33 mg, 0.3 mmol, 1.5 equiv), [Ir{dt(tBu)$_2$ppy}$_2$(dtbbpy)][PF$_6$] **PC1** (1.3 mg, 0.5 mol %) and 1,4-dioxane (2.0 mL) under air cinditions. The vial was equipped with a Teflon septum and stirred at 80 ℃ under blue LED irradiation with two Kessil LEDs (30 W, 456 nm, approximately 3 cm away from the reaction mixture) for 24 hour. The solvent was removed in vacuo and the residue was purified by flash column chromatography on silica gel to yield the products **5** (For the possible reaction mechanism, see **Fig. S10**).

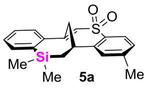

2,12,12-Trimethyl-13,14-dihydro-12H-7,14-methanodibenzo[b,g][1,6]thiasilecine 5,5-dioxide 5a. The product was obtained from allyl(2-ethynylphenyl)dimethylsilane **4** (40.0 mg, 0.20 mmol) and 4-methylbenzenesulfonyl azide **2a** (157.6 mg, 4.0 equiv) and was purified by column chromatography to give white solid (45 mg, 63%); mp: 236.5 – 238.7 ℃; TCL (petroleum ether): R_f = 0.75; ^{1}H NMR (500 MHz, $CDCl_3$) δ 7.58 (d, J = 8.1 Hz, 2H), 7.28 (d, J = 7.3 Hz, 1H), 7.21 (d, J = 7.7 Hz, 1H), 7.18 (d, J = 7.3 Hz, 1H), 7.15 (d, J = 7.8 Hz, 2H), 7.10 – 7.06 (m, 1H), 4.01 (d, J = 14.5 Hz, 1H), 3.89 – 3.83 (m, 1H), 3.78 (d, J = 14.5 Hz, 1H), 2.25 (s, 2H), 1.39 – 1.36 (m, 1H), 1.28 (t, J = 13.5 Hz, 1H), 0.22 (s, 3H), -0.00 (s, 3H); ^{13}C NMR (126 MHz, $CDCl_3$) δ 147.0, 145.8, 140.3, 139.3, 138.0, 135.8, 132.0, 131.9, 131.1, 130.5, 129.6, 129.3, 65.3, 60.5, 29.1, 23.6, 1.9, -0.0; IR (KBr): 3613, 1716, 1697, 1508, 1338, 881, 431 cm^{-1}; HRMS-ESI (m/z): [M+H]$^+$ calcd. for $C_{20}H_{23}O_2SSi$, 355.1183; found 355.1180.

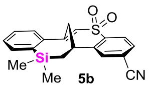

12,12-Dimethyl-13,14-dihydro-12H-7,14-methanodibenzo[b,g][1,6]thiasilecine-2-carbonitrile 5,5-dioxide 5b. The product was obtained from allyl(2-ethynylphenyl)dimethylsilane **4** (40.0 mg, 0.20 mmol) and 4-cyanobenzenesulfonyl azide **2p** (166.4 mg, 4.0 equiv) and was purified by column chromatography to give white solid (43 mg, 59%); mp: 251.8 – 254.4 ℃; TCL (petroleum ether:ethyl acetate, 100:1 v/v): R_f = 0.70; ^{1}H NMR (400 MHz, $CDCl_3$) δ 8.29 (d, J = 8.3 Hz, 1H), 7.72 – 7.63 (m, 3H), 7.50 – 7.43 (m, 3H), 6.91 (s, 1H), 4.62 (d, J = 15.1 Hz, 1H), 3.63 (dt, J = 11.4, 5.7 Hz, 1H), 2.93 (ddd, J = 15.1, 6.2, 2.0 Hz, 1H), 1.41 – 1.34 (m, 1H), 1.22 (d, J = 14.9 Hz, 1H), 0.50 (s, 3H), 0.31 (s, 3H); ^{13}C NMR (101 MHz, $CDCl_3$) δ 160.6, 147.4, 146.1, 144.7, 138.7, 136.4, 136.2, 131.1, 130.7, 130.2, 128.5, 127.6, 118.4, 117.8, 38.0, 37.0, 26.5, -0.0, -0.7; IR (KBr): 3714, 3667, 3613, 1647, 1508, 1292, 518, 430 cm^{-1}; HRMS-ESI (m/z): [M+H]$^+$ calcd. for $C_{20}H_{20}NO_2SSi$, 366.0979; found 366.0984.

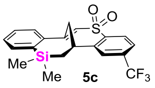

12,12-Dimethyl-2-(trifluoromethy)-13,14-dihydro-12H-7,14-methanodibenzo[b,g][1,6]thiasilecine 5,5-dioxide 5c. The product was obtained from allyl(2-ethynylphenyl)dimethylsilane **4** (40.0 mg, 0.20 mmol) and 4-(trifluoromethyl)benzenesulfonyl azide **2o** (200.8 mg, 4.0 equiv) and was purified by column chromatography to give white solid (49 mg, 65%); mp: 184.8 – 188.2 ℃; TCL (petroleum ether:ethyl acetate, 100:1 v/v): R_f = 0.80; ^{1}H NMR (400 MHz, $CDCl_3$) δ 8.32 (d, J = 8.3 Hz, 1H), 7.70 – 7.61 (m, 3H), 7.52 – 7.40 (m, 3H), 6.93 (s, 1H), 4.65 (d, J = 15.1 Hz, 1H), 3.69 (dt, J = 11.7, 5.7 Hz, 1H), 2.94 (ddd, J = 15.0, 6.1, 1.7 Hz, 1H), 1.46 – 1.36 (m, 1H), 1.27 (d, J = 12.8 Hz, 1H), 0.52 (s, 3H), 0.31 (s, 3H); ^{13}C NMR (101 MHz, $CDCl_3$) δ 160.4, 147.5, 145.9, 144.0, 138.8, 136.2, 135.6 (q, J = 32.3 Hz, $^2J_{CF}$), 131.4, 130.6, 130.1, 129.6 (q, J = 4.0 Hz, $^3J_{CF}$), 128.4, 127.4, 124.3 (d, J = 273.7 Hz, $^1J_{CF}$), 124.7 (q, J = 4.0 Hz, $^3J_{CF}$), 38.1, 37.1, 26.4, -0.7; IR (KBr): 3613, 1716, 1508,

1290, 906, 753 cm^{-1}; HRMS-ESI (m/z): $[M+H]^+$ calcd. for $C_{20}H_{20}O_2SF_3Si$, 409.0900; found 409.0900.

3. Arylsulfonylazide scope

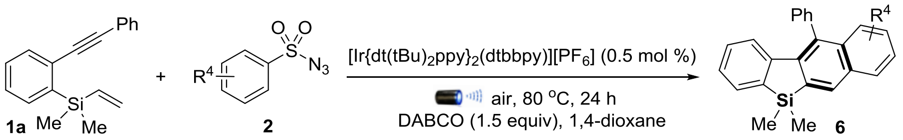

To a 10 mL vial equipped with a magnetic stir bar, was added vinylsilanes **1a** (0.2 mmol, 1.0 equiv), arylsulfonylazides **2** (0.8 mmol, 4.0 equiv), DABCO (33 mg, 0.3 mmol, 1.5 equiv), [Ir{dt(tBu)$_2$ppy}$_2$(dtbbpy)][PF$_6$] **PC1** (1.3 mg, 0.5 mol %) and 1,4-dioxane (2.0 mL) under air. The vial was equipped with a Teflon septum and stirred at 80 ℃ under blue LED irradiation with two Kessil LEDs (30 W, 456 nm, approximately 3 cm away from the reaction mixture) for 24 hour. The solvent was removed in vacuo and the residue was purified by flash column chromatography on silica gel to yield the products **6**.

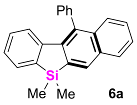

5,5-Dimethyl-11-phenyl-5H-benzo[b]naphtho[2,3-d]silole 6a. The product was obtained from dimethyl(2-(phenylethynyl)phenyl)(vinyl)silane **1a** (52.4 mg, 0.20 mmol) and benzenesulfonyl azide **2a** (146.4 mg, 4.0 equiv) and was purified by column chromatography to give white solid (40 mg, 60%); mp: 185.2 – 186.7 ℃; TCL (petroleum ether): R_f = 0.85; ^{1}H NMR (500 MHz, CDCl$_3$) δ 8.15 (s, 1H), 7.85 (d, J = 8.0 Hz, 1H), 7.62 (d, J = 6.9 Hz, 1H), 7.59 – 7.52 (m, 3H), 7.43 (t, J = 7.2 Hz, 1H), 7.37 (t, J = 8.4 Hz, 3H), 7.33 (d, J = 7.4 Hz, 1H), 7.15 (t, J = 7.1 Hz, 1H), 6.99 (t, J = 7.7 Hz, 1H), 6.52 (d, J = 8.2 Hz, 1H), 0.50 (s, 6H); ^{13}C NMR (126 MHz, CDCl$_3$) δ 148.7, 141.1, 141.1, 140.7, 138.3, 135.2, 135.1, 133.2, 132.6, 132.5, 130.1, 129.7, 129.4, 127.7, 127.6, 127.0, 126.8, 126.5, 126.4, 125.7, -2.6; IR (KBr): 3832, 3649, 3564, 1264, 741 cm^{-1}; HRMS-ESI (m/z): $[M+H]^+$ calcd. for $C_{24}H_{21}Si$, 337.1407; found 337.1413.

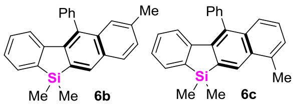

5,5,9-Trimethyl-11-phenyl-5H-benzo[b]naphtho[2,3-d]silole 6b and 5,5,7-Trimethyl-11-phenyl-5H-benzo[b]naphtha [2,3-d]silole 6c. The product was obtained from dimethyl(2-(phenylethynyl)phenyl)(vinyl)silane **1a** (52.4 mg, 0.20 mmol) and 3-methylbenzenesulfonyl azide **2c** (157.6 mg, 4.0 equiv) and was purified by column chromatography to give pale yellow solid [52 mg, **6b** + **6c** = 74% (1:1)]; mp: 218.5 – 219.6 ℃; TCL (petroleum ether): R_f = 0.83; ^{1}H NMR (400 MHz, CDCl$_3$) δ 8.41 (s, 1H), 8.18 (s, 1H), 7.82 (d, J = 8.2 Hz, 1H), 7.70 (t, J = 6.6 Hz, 2H), 7.63 (dd, J = 6.2, 4.2 Hz, 6H), 7.43 (d, J = 6.7 Hz, 4H), 7.34 (d, J = 6.6 Hz, 3H), 7.30 (d, J = 8.7 Hz, 1H), 7.22 (q, J = 8.2 Hz, 3H), 7.08 – 7.04 (m, 2H), 6.55 (t, J = 7.3 Hz, 2H), 2.86 (s, 3H), 2.43 (s, 3H), 0.60 (s, 6H), 0.57 (s, 6H); ^{13}C NMR (101 MHz, CDCl$_3$) δ 148.8, 148.7, 141.2, 141.2, 141.1, 140.9, 140.8, 138.2, 137.1, 136.2, 135.6, 135.3, 135.3, 134.6, 134.0, 133.0, 132.6, 131.4, 130.8, 130.2, 130.2, 129.8, 129.7, 129.4, 129.3, 129.1, 127.9, 127.7, 127.6, 126.8, 126.7, 126.7, 126.5, 126.1, 126.1, 125.6, 22.1, 19.7, -2.4, -2.5; IR (KBr): 3046, 2920, 2855, 1647, 1440, 1246, 1125, 1066, 700 cm^{-1}; HRMS-ESI (m/z): $[M+H]^+$ calcd. for $C_{25}H_{23}Si$, 351.1564; found 351.1572.

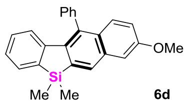

8-Methoxy-5,5-dimethyl-11-phenyl-5H-benzo[b]naphtho[2,3-d]silole 6d. The product was obtained from dimethyl(2-(phenylethynyl)phenyl)(vinyl)silane **1a** (52.4 mg, 0.20 mmol) and 4-methoxybenzenesulfonyl azide **2d** (170.4 mg, 4.0 equiv) and was purified by column

chromatography to give white solid (51 mg, 70%); mp: 121.7 – 122.5 ℃; TCL (petroleum ether): R_f = 0.84; ^{1}H NMR (500 MHz, $CDCl_3$) δ 8.06 (s, 1H), 7.60 (d, J = 6.7 Hz, 1H), 7.57 – 7.50 (m, 3H), 7.36 – 7.28 (m, 2H), 7.29 (d, J = 9.3 Hz, 1H), 7.18 (d, J = 2.5 Hz, 1H), 7.12 (t, J = 7.2 Hz, 1H), 7.00 – 6.96 (m, 2H), 6.47 (d, J = 8.2 Hz, 1H), 3.91 (s, 3H), 0.49 (s, 6H); ^{13}C NMR (126 MHz, $CDCl_3$) δ 157.6, 148.9, 140.8, 140.6, 139.3, 139.1, 135.2, 133.7, 132.6, 132.0, 130.5, 130.1, 129.7, 129.3, 128.7, 127.6, 126.4, 126.0, 118.7, 106.0, 55.4, -2.5; IR (KBr): 3438, 2924, 2852, 1618, 1442, 1226, 1035, 706 cm^{-1}; HRMS-ESI (m/z): $[M+H]^+$ calcd. for $C_{25}H_{23}OSi$, 367.1513; found 367.1519.

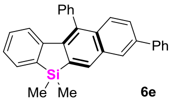

5,5-Dimethyl-8,11-diphenyl-5H-benzo[b]naphtho[2,3-d]silole 6e. The product was obtained from dimethyl(2-(phenylethynyl)phenyl)(vinyl)silane **1a** (52.4 mg, 0.20 mmol) and [1,1'-biphenyl]-4-sulfonyl azide **2e** (207.2 mg, 4.0 equiv) and was purified by column chromatography to give white solid (35 mg, 43%); mp: 217.3 – 218.6 ℃; TCL (petroleum ether): R_f = 0.86; ^{1}H NMR (500 MHz, $CDCl_3$) δ 8.22 (s, 1H), 8.06 (s, 1H), 7.70 (d, J = 7.2 Hz, 2H), 7.63 (d, J = 6.6 Hz, 1H), 7.61 – 7.52 (m, 4H), 7.46 (dd, J = 13.6, 7.6 Hz, 3H), 7.38 (dd, J = 17.7, 7.0 Hz, 3H), 7.15 (t, J = 7.0 Hz, 1H), 7.00 (t, J = 7.2 Hz, 1H), 6.54 (d, J = 8.0 Hz, 1H), 0.52 (s, 6H); ^{13}C NMR (126 MHz, $CDCl_3$) δ 148.6, 141.2, 141.1, 141.0, 140.6, 138.9, 138.3, 134.9, 134.4, 133.4, 132.7, 132.7, 130.1, 129.8, 129.4, 128.9, 127.7, 127.6, 127.4, 127.3, 126.8, 126.5, 126.0, 125.6, -2.5; IR (KBr): 3400, 2922, 2852, 1590, 1442, 1246, 772 cm^{-1}; HRMS-ESI (m/z): $[M+H]^+$ calcd. for $C_{30}H_{25}Si$, 413.1720; found 413.1728.

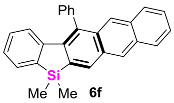

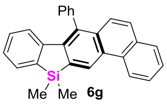

5,5-Dimethyl-13-phenyl-5H-anthra[2,3-b]benzo[d]silole 6f and 12,12-dimethyl-7-phenyl-12H-benzo [b]phenanthro[2,3-d]silole 6g. The product was obtained from dimethyl(2-(phenylethynyl)phenyl)(vinyl)silane **1a** (52.4 mg, 0.20 mmol) and naphthalene-2-sulfo nyl azide **2f** (186.4 mg, 4.0 equiv) and was purified by column chromatography to give white solid [47 mg, **6f** + **6g** = 61% (2:1)]; mp: 133.9 – 138.7 ℃; TCL (petroleum ether): R_f = 0.84; ^{1}H NMR (500 MHz, $CDCl_3$) δ 9.04 (s, 1H), 8.84 (d, J = 8.3 Hz, 1H), 8.38 (s, 0.3H), 8.32 (s, 0.3H), 7.96 (d, J = 8.4 Hz, 0.3H), 7.90 (s, 0.3H), 7.83 (d, J = 7.9 Hz, 1H), 7.76 (d, J = 8.4 Hz, 0.3H), 7.68 (d, J = 7.2 Hz, 0.7H), 7.66 – 7.62 (m, 2H), 7.62 – 7.53 (m, 6H), 7.43 (d, J = 7.7 Hz, 0.7H), 7.41 – 7.37 (m, 3H), 7.37 – 7.33 (m, 0.8H), 7.18 – 7.12 (m, 1.3H), 7.04 – 6.97 (m, 1.4H), 6.55 (d, J = 8.2 Hz, 0.3H), 6.49 (d, J = 8.2 Hz, 1H), 0.55 (d, J = 2.6 Hz, 6H), 0.53 (d, J = 2.2 Hz, 2H); ^{13}C NMR (126 MHz, $CDCl_3$) δ 150.9, 144.8, 143.7, 143.3, 143.3, 143.0, 142.0, 141.1, 140.5, 138.3, 136.7, 136.2, 136.0, 135.6, 135.0, 134.9, 134.4, 134.0, 133.8, 133.1, 132.6, 132.5, 132.3, 132.1, 131.8, 131.7, 131.2, 131.0, 130.7, 130.2, 130.0, 130.0, 129.4, 129.4, 129.2, 129.1, 129.1, 129.0, 128.9, 128.5, 128.5, 128.3, 127.9, 127.7, 127.6, 125.2, 0.0, -0.3; IR (KBr): 3451, 2922, 2853, 1441, 1246, 743, 700 cm^{-1}; HRMS-ESI (m/z): $[M+ H]^+$ calcd. for $C_{28}H_{23}Si$, 387.1564; found 387.1567.

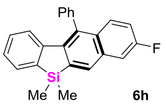

8-Fluoro-5,5-dimethyl-11-phenyl-5H-benzo[b]naphtho[2,3-d]silole 6h. The product was obtained from dimethyl(2-(phenylethynyl)phenyl)(vinyl)silane **1a** (52.4 mg, 0.20 mmol) and 4-fluorobenzenesulfonyl azide **2h** (160.8 mg, 4.0 equiv) and was purified by column chromatography to give white solid (38 mg, 53%); mp: 139.6 – 141.1 ℃; TCL (petroleum ether): R_f = 0.82; ^{1}H NMR (400 MHz, $CDCl_3$) δ 8.08 (s, 1H), 7.61 (d, J = 6.8 Hz, 1H), 7.55 (t, J = 6.9 Hz, 3H), 7.45 (dd, J = 9.4, 2.5 Hz, 1H), 7.39 – 7.32 (m, 3H), 7.14 (t, J = 7.2 Hz, 1H), 7.08 (td, J = 8.9, 2.6 Hz, 1H), 6.98 (t, J = 7.7 Hz, 1H), 6.49 (d, J = 8.2 Hz, 1H), 0.50 (s, 6H); ^{13}C NMR (101 MHz, $CDCl_3$) δ 160.6 (d, J = 248.5 Hz, $^1J_{CF}$), 148.4, 140.8, 140.6 (d, J = 2.0 Hz, $^4J_{CF}$), 140.4, 139.9, 135.1 (d, J = 2.0 Hz, $^4J_{CF}$), 133.3 (d, J = 8.1 Hz, $^3J_{CF}$), 132.6, 132.3 (d,

J = 5.1 Hz, $^{3}J_{CF}$), 132.2, 130.0, 129.8, 129.6 (d, J = 24.2 Hz, $^{2}J_{CF}$), 129.4, 127.8, 126.8, 126.3, 116.3 (d, J = 3.0 Hz, $^{4}J_{CF}$), 110.7 (d, J = 19.2 Hz, $^{2}J_{CF}$), -2.6; IR (KBr): 3731, 3397, 2921, 2853, 1503, 1442, 1210, 1143, 782 cm^{-1}; HRMS-ESI (m/z): $[M+H]^+$ calcd. for $C_{24}H_{20}FSi$, 355.1313; found 355.1329.

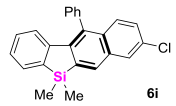

6i

8-Chloro-5,5-dimethyl-11-phenyl-5H-benzo[b]naphtho[2,3-d]silole 6i. The product was obtained from dimethyl(2-(phenylethynyl)phenyl)(vinyl)silane **1a** (52.4 mg, 0.20 mmol) and 4-chlorobenzenesulfonyl azide **2i** (173.6 mg, 4.0 equiv) and was purified by column chromatography to give colorless solid (48 mg, 65%); mp: 145.9 – 146.2 °C; TCL (petroleum ether): R_f = 0.80; ^{1}H NMR (400 MHz, $CDCl_3$) δ 8.05 (s, 1H), 7.82 (s, 1H), 7.62 (d, J = 6.9 Hz, 1H), 7.56 (d, J = 6.8 Hz, 3H), 7.36 – 7.29 (m, 3H), 7.24 (d, J = 10.0 Hz, 1H), 7.15 (t, J = 7.1 Hz, 1H), 6.99 (t, J = 7.7 Hz, 1H), 6.50 (d, J = 8.1 Hz, 1H), 0.50 (s, 6H); ^{13}C NMR (101 MHz, $CDCl_3$) δ 148.3, 141.4, 141.0, 140.1, 140.0, 135.0, 133.5, 133.1, 132.6, 132.1, 131.5, 130.0, 129.8, 129.4, 128.7, 127.8, 127.1, 127.0, 126.5, 126.3, -2.7; IR (KBr): 3648, 3342, 2920, 1647, 777, 702 cm^{-1}; HRMS-ESI (m/z): $[M+H]^+$ calcd. for $C_{24}H_{20}ClSi$, 371.1017; found 371.1032.

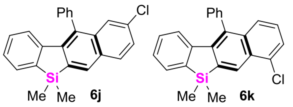

6j 6k

9-Chloro-5,5-dimethyl-11-phenyl-5H-benzo[b]naphtho[2,3-d]silole 6j and 7-chloro-5,5-dimethyl-11-phenyl-5H-benzo[b]naphtho[2,3-d]silole 6k. The product was obtained from dimethyl(2-(phenylethynyl)phenyl)(vinyl)silane **1a** (52.4 mg, 0.20 mmol) and 3-chlorobenzenesulfonyl azide **2j** (173.6 mg, 4.0 equiv) and was purified by column chromatography to give colorless oil [50 mg, **6j** + **6k** = 68% (1:1)]; TCL (petroleum ether): R_f = 0.80; ^{1}H NMR (400 MHz, $CDCl_3$) δ 8.67 (s, 1H), 8.18 (s, 1H), 7.83 (d, J = 8.4 Hz, 1H), 7.70 (t, J = 7.2 Hz, 2H), 7.65 – 7.62 (m,5H), 7.57 (d, J = 7.4 Hz, 1H), 7.44 – 7.37 (m, 7H), 7.30 – 7.28 (m, 1H), 7.23 (dd, J = 11.8, 7.2 Hz, 3H), 7.06 (t, J = 7.8 Hz, 2H), 6.56 (d, J = 8.2 Hz, 2H), 0.60 (s, 6H), 0.57 (s, 6H); ^{13}C NMR (101 MHz, $CDCl_3$) δ 148.2, 148.1, 142.2, 141.9, 141.4, 141.2, 140.4, 140.0, 139.9, 138.8, 136.6, 136.1, 135.1, 134.3, 132.9, 132.7, 132.7, 132.4, 132.1, 130.7, 130.1, 130.1, 129.9, 129.6, 129.5, 129.3, 129.1, 128.0, 127.9, 127.2; 127.1, 126.6, 126.5, 126.4, 126.0, 126.0, 125.9, -2.5, -2.6; IR (KBr): 3746, 3439, 2922, 1441, 917, 781, 702 cm^{-1}; HRMS-ESI (m/z): $[M+H]^+$ calcd. for $C_{24}H_{20}ClSi$, 371.1017; found 371.1027.

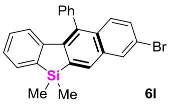

6l

8-Bromo-5,5-dimethyl-11-phenyl-5H-benzo[b]naphtho[2,3-d]silole 6l. The product was obtained from dimethyl(2-(phenylethynyl)phenyl)(vinyl)silane **1a** (52.4 mg, 0.20 mmol) and 4-bromobenzenesulfonyl azide **2l** (208.8 mg, 4.0 equiv) and was purified by column chromatography to give colorless oil (58 mg, 70%); TCL (petroleum ether): R_f = 0.80; ^{1}H NMR (500 MHz, $CDCl_3$) δ 8.04 (s, 1H), 7.99 (s, 1H), 7.62 (d, J = 6.9 Hz, 1H), 7.58 – 7.53 (m, 3H), 7.37 (d, J = 9.0 Hz, 1H), 7.33 (d, J = 6.8 Hz, 2H), 7.24 (d, J = 8.0 Hz, 1H), 7.15 (t, J = 7.1 Hz, 1H), 6.99 (t, J = 7.7 Hz, 1H), 6.50 (d, J = 8.1 Hz, 1H), 0.50 (s, 6H); ^{13}C NMR (126 MHz, $CDCl_3$) δ 148.3, 141.5, 141.1, 140.1, 140.0, 135.0, 133.7, 133.5, 132.7, 132.1, 130.0, 129.8, 129.7, 129.6, 129.5, 128.9, 127.8, 127.0, 126.5, 119.8, -2.7; IR (KBr): 3777, 3447, 2924, 1439, 1384, 901 cm^{-1}; HRMS-ESI (m/z): $[M+H]^+$ calcd. for $C_{24}H_{20}BrSi$, 415.0512; found 415.2125.

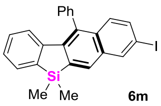

6m

8-Iodo-5,5-dimethyl-11-phenyl-5H-benzo[b]naphtho[2,3-d]silole 6m. The product was obtained from dimethyl(2-(phenylethynyl)phenyl)(vinyl)silane **1a** (52.4 mg, 0.20 mmol) and 4-iodobenzenesulfonyl azide **2m** (247.2 mg, 4.0 equiv) and was purified by column chromatography to give white solid (19 mg, 21%); mp: 181.2 –

183.4 ℃; TCL (petroleum ether): R_f = 0.82; ^{1}H NMR (400 MHz, $CDCl_3$) δ 8.28 (s, 1H), 8.06 (s, 1H), 7.67 (d, J = 7.0 Hz, 1H), 7.64 – 7.56 (m, 4H), 7.38 (d, J = 7.4 Hz, 2H), 7.20 (t, J = 7.1 Hz, 1H), 7.14 (d, J = 9.0 Hz, 1H), 7.04 (t, J = 7.7 Hz, 1H), 6.55 (d, J = 8.2 Hz, 1H), 0.54 (s, 6H); ^{13}C NMR (101 MHz, $CDCl_3$) δ 148.3, 141.6, 141.1, 140.0, 139.8, 136.4, 135.0, 134.9, 134.1, 134.0, 132.7, 131.9, 130.0, 129.8, 129.5, 128.8, 127.8, 127.0, 126.6, 91.5, -2.7; IR (KBr): 3528, 2921, 2851, 1714, 1509, 1338, 900, 752 cm^{-1}; HRMS-ESI (m/z): $[M+H]^+$ calcd. for $C_{24}H_{20}ISi$, 463.0374; found 463.0374.

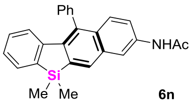
6n

***N*-(5,5-Dimethyl-11-phenyl-5H-benzo[b]naphtho[2,3-d]silol-8-yl)acetamide 6n**. The product was obtained from dimethyl(2-(phenylethynyl)phenyl)(vinyl)silane **1a** (52.4 mg, 0.20 mmol) and 4-acetamidobenzenesulfonyl azide **2n** (192.0 mg, 4.0 equiv) and was purified by column chromatography to give white solid (31 mg, 40%); mp: 129.6 – 130.6 ℃; TCL (petroleum ether:ethyl acetate, 4:1 v/v): R_f = 0.12; ^{1}H NMR (400 MHz, DMSO) δ 10.20 (s, 1H), 8.40 (s, 1H), 8.25 (s, 1H), 7.66 (d, J = 30.3 Hz, 3H), 7.44 (d, J = 7.6 Hz, 1H), 7.34 (s, 2H), 7.19 – 7.14 (m, 2H), 6.96 (s, 1H), 6.39 (d, J = 5.9 Hz, 1H), 2.12 (s, 3H), 0.49 (s, 6H); ^{13}C NMR (101 MHz, DMSO) δ 169.1, 148.5, 141.1, 140.4, 139.5, 138.8, 137.4, 134.9, 133.4, 133.1, 131.4, 130.1, 130.0, 128.3, 127.3, 127.1, 125.8, 120.8, 115.4, 24.6, -2.3; IR (KBr): 3650, 3526, 1715, 1650, 1508, 1287, 954, 745 cm^{-1}; HRMS-ESI (m/z): $[M+H]^+$ calcd. for $C_{26}H_{24}NOSi$, 394.1622; found 394.1616.

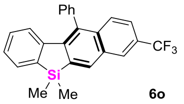
6o

5,5-Dimethyl-11-phenyl-8-(trifluoromethyl)-5H-benzo[b]naphtho[2,3-d]silole 6o. The product was obtained from dimethyl(2-(phenylethynyl)phenyl)(vinyl)silane **1a** (52.4 mg, 0.20 mmol) and 4-(trifluoromethyl)benzenesulfonyl azide **2o** (200.8 mg, 4.0 equiv) and was purified by column chromatography to give white solid (44 mg, 55%); mp: 149.2 – 150.3 ℃; TCL (petroleum ether:ethyl acetate, 100:1 v/v): R_f = 0.80; ^{1}H NMR (500 MHz, $CDCl_3$) δ 8.23 (s, 1H), 8.15 (s, 1H), 7.64 (d, J = 7.0 Hz, 1H), 7.60 – 7.55 (m, 3H), 7.50 – 7.46 (m, 2H), 7.34 (d, J = 7.0 Hz, 2H), 7.18 (t, J = 7.2 Hz, 1H), 7.01 (t, J = 7.7 Hz, 1H), 6.55 (d, J = 8.2 Hz, 1H), 0.51 (s, 6H); ^{13}C NMR (126 MHz, $CDCl_3$) δ 148.1, 143.2, 141.3, 140.2, 140.0, 136.6, 134.9, 133.8, 132.8, 131.3, 130.0, 129.9, 129.6, 128.0, 128.0, , 127.4, 127.3 (q, J = 32.8 Hz, $^2J_{CF}$), 126.8, 125.4 (q, J = 5.0 Hz, $^3J_{CF}$), 124.5 (d, J = 272.2 Hz, $^1J_{CF}$), 121.9 (q, J = 2.5 Hz, $^4J_{CF}$), -2.7; IR (KBr): 3432, 2921, 1632, 1426, 1344, 1302, 1109, 917, 783 cm^{-1}; HRMS-ESI (m/z): $[M+H]^+$ calcd. for $C_{25}H_{20}F_3Si$, 405.1281; found 405.1285.

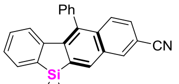
6p

5,5-Dimethyl-11-phenyl-5H-benzo[b]naphtho[2,3-d]silole-8-carbonitrile 6p. The product was obtained from dimethyl(2-(phenylethynyl)phenyl)(vinyl)silane **1a** (52.4 mg, 0.20 mmol) and 4-cyanobenzenesulfonyl azide **2p** (166.4 mg, 4.0 equiv) and was purified by column chromatography to give white solid (52 mg, 72%); mp: 122.8 – 123.6 ℃; TCL (petroleum ether:ethyl acetate, 100:1 v/v): R_f = 0.75; ^{1}H NMR (400 MHz, $CDCl_3$) δ 8.27 (s, 1H), 8.23 (s, 1H), 7.70 (d, J = 7.0 Hz, 1H), 7.65 – 7.63 (m, 3H), 7.49 (s, 2H), 7.39 – 7.37 (m, 2H), 7.25 (t, J = 7.2 Hz, 1H), 7.06 (t, J = 7.6 Hz, 1H), 6.59 (d, J = 8.2 Hz, 1H), 0.57 (s, 6H); ^{13}C NMR (101 MHz, $CDCl_3$) δ 147.7, 144.3, 141.5, 140.9, 139.5, 136.7, 135.0, 133.7, 133.3, 132.8, 131.3, 130.0, 130.0, 129.7, 128.2, 127.7, 127.0, 126.9, 119.5, 108.8, -2.7; IR (KBr): 3455, 2922, 2218, 1616, 1442, 1242, 701 cm^{-1}; HRMS-ESI (m/z): $[M+H]^+$ calcd. for $C_{25}H_{20}NSi$, 362.1360; found 362.1363.

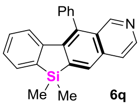
6q

6,6-Dimethyl-11-phenyl-6H-benzo[4,5]silolo[2,3-g]isoquinoline 6q. The product was obtained from dimethyl(2-(phenylethynyl)phenyl)(vinyl)silane **1a** (52.4 mg,

0.20 mmol) and pyridine-3-sulfonyl azide **2q** (147.2 mg, 4.0 equiv) and was purified by column chromatography to give white solid (28 mg, 42%); mp: 151.7 – 154.3 ℃; TCL (petroleum ether): R_f = 0.80; ^{1}H NMR (500 MHz, $CDCl_3$) δ 8.10 (s, 1H), 7.64 (d, J = 7.0 Hz, 1H), 7.62 – 7.56 (m, 3H), 7.50 (d, J = 6.5 Hz, 2H), 7.40 (s, 2H), 7.28 (s, 1H), 7.17 (t, J = 7.2 Hz, 1H), 7.03 (t, J = 7.7 Hz, 1H), 6.73 (d, J = 8.1 Hz, 1H), 0.52 (s, 6H); ^{13}C NMR (126 MHz, $CDCl_3$) δ 148.4, 145.8, 141.1, 140.8, 140.0, 138.4, 137.2, 133.1, 132.7, 129.6, 129.5, 129.1, 128.2, 127.6, 127.1, 126.5, 125.7, 123.8, -2.7; IR (KBr): 3459, 2924, 1654, 1561, 1243, 785 cm^{-1}; HRMS-ESI (m/z): $[M+H]^+$ calcd. for $C_{23}H_{20}NSi$, 338.1360; found 338.3418.

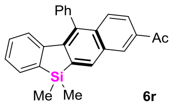

6r

1-(5,5-Dimethyl-11-phenyl-5H-benzo[b]naphtho[2,3-d]silol-8-yl)ethan-1-one 6r. The product was obtained from dimethyl(2-(phenylethynyl)phenyl)(vinyl)silane **1a** (52.4 mg, 0.20 mmol) and 4-acetylbenzenesulfonyl azide **2r** (180.0 mg, 4.0 equiv) and was purified by column chromatography to give white solid (42 mg, 55%); mp: 135.0 – 140.4 ℃; TCL (petroleum ether:ethyl acetate, 100:1 v/v): R_f = 0.60; ^{1}H NMR (400 MHz, $CDCl_3$) δ 8.48 (s, 1H), 8.27 (s, 1H), 7.87 (d, J = 8.9 Hz, 1H), 7.64 (d, J = 6.9 Hz, 1H), 7.57 (t, J = 9.7 Hz, 3H), 7.44 (d, J = 8.9 Hz, 1H), 7.35 (d, J = 6.4 Hz, 2H), 7.18 (t, J = 6.9 Hz, 1H), 7.01 (t, J = 7.7 Hz, 1H), 6.55 (d, J = 8.2 Hz, 1H), 2.71 (s, 3H), 0.52 (s, 6H); ^{13}C NMR (101 MHz, $CDCl_3$) δ 198.0, 148.1, 143.7, 141.4, 140.1, 139.6, 137.5, 135.0, 134.5, 134.0, 132.7, 131.6, 130.0, 129.9, 129.8, 129.5, 127.9, 127.4, 127.4, 126.9, 124.4, 26.7, -2.7; IR (KBr): 3713, 3666, 3612, 1749, 1508, 1288, 907 cm^{-1}; HRMS-ESI (m/z): $[M+H]^+$ calcd. for $C_{26}H_{23}OSi$, 379.1513; found 379.1509.

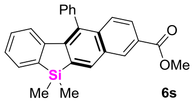

6s

Methyl-5,5-dimethyl-11-phenyl-5H-benzo[b]naphtho[2,3-d]silole-8-carboxylate 6s. The product was obtained from dimethyl(2-(phenylethynyl)phenyl)(vinyl)silane **1a** (52.4 mg, 0.20 mmol) and methyl 4-(azidosulfonyl)benzoate **2s** (192.8 mg, 4.0 equiv) and was purified by column chromatography to give white solid (45 mg, 57%); mp: 177.2 – 180.3 ℃; TCL (petroleum ether:ethyl acetate, 50:1 v/v): R_f = 0.63; ^{1}H NMR (500 MHz, $CDCl_3$) δ 8.61 (s, 1H), 8.25 (s, 1H), 7.89 (d, J = 8.7 Hz, 1H), 7.64 (d, J = 6.4 Hz, 1H), 7.58 (d, J = 7.0 Hz, 3H), 7.42 (d, J = 8.5 Hz, 1H), 7.35 (d, J = 6.2 Hz, 2H), 7.18 (t, J = 6.0 Hz, 1H), 7.01 (t, J = 7.1 Hz, 1H), 6.54 (d, J = 7.8 Hz, 1H), 3.96 (s, 3H), 0.52 (s, 6H); ^{13}C NMR (126 MHz, $CDCl_3$) δ 167.3, 148.1, 143.4, 141.4, 140.1, 139.4, 137.4, 134.9, 134.3, 132.7, 131.6, 130.7, 130.0, 129.8, 129.5, 127.9, 127.3, 127.2, 126.9, 126.8, 125.7, 52.2, -2.7; IR (KBr): 3523, 2923, 1655, 1560, 1438, 1385, 1262, 999, 754 cm^{-1}; HRMS-ESI (m/z): $[M+H]^+$ calcd. for $C_{26}H_{23}O_2Si$, 395.1462; found 395.1458.

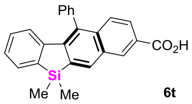

6t

5,5-Dimethyl-11-phenyl-5H-benzo[b]naphtho[2,3-d]silole-8-carboxylic acid 6t. The product was obtained from dimethyl(2-(phenylethynyl)phenyl)(vinyl)silane **1a** (52.4 mg, 0.20 mmol) and 4-(azidosulfonyl)benzoic acid **2t** (181.6 mg, 4.0 equiv) and was purified by column chromatography to give white solid (40 mg, 53%); mp: 223.1 – 225.9 ℃; TCL (petroleum ether:aceton:acetic acid, 300:50:1 v/v/v): R_f = 0.10; ^{1}H NMR (400 MHz, DMSO) δ 8.61 (s, 1H), 8.52 (s, 1H), 7.88 (d, J = 8.9 Hz, 1H), 7.74 (d, J = 6.9 Hz, 1H), 7.69 – 7.58 (m, 3H), 7.40 – 7.29 (m, 3H), 7.18 (t, J = 7.1 Hz, 1H), 6.98 (t, J = 7.7 Hz, 1H), 6.44 (d, J = 8.2 Hz, 1H), 0.50 (s, 6H); ^{13}C NMR (101 MHz, DMSO) δ 169.7, 167.7, 149.8, 144.9, 143.7, 141.9, 141.4, 138.9, 137.0, 136.8, 135.5, 133.6, 132.7, 132.0, 130.5, 128.9, 128.4, -0.5; IR (KBr): 3680, 1757, 1507, 1289, 952 cm^{-1}; HRMS-ESI (m/z): $[M+H]^+$ calcd. for $C_{25}H_{21}O_2Si$, 381.1305; found 381.1301.

V. Synthetic Application

The procedure for the preparation of compound **8** [8]

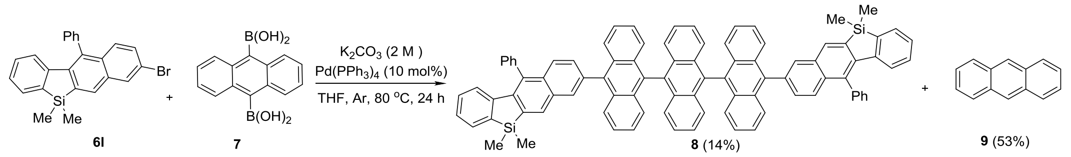

The compound 8-bromo-5,5-dimethyl-11-phenyl-5H-benzo[b]naphtho[2,3-d]silole **6l** (199 mg, 0.48 mmol) and compound anthracene-9,10-diyldiboronic acid **7** (45 mg, 0.2 mmol) were added to a 10 mL round-bottom flask under argon, followed by addition of 10 mL THF. After the mixture was bubbled with argon stream for 15 min, a K_2CO_3 aqueous solution (2 M) was added and then argon bubbling was continued for another 15 min. Afterwards, tetrakispalladium (23 mg, 0.02 mmol) was added under argon. The mixture was stirred at 80 ^oC for 24 h. Finally, the solvent was removed in vacuo and the residue was purified by flash column chromatography on silica gel (petrol ether) to obtain the product **8** (14%) and the byproduct **9** (53%).[9]

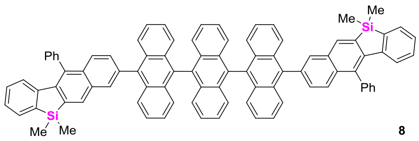

8

10,10''-Bis(5,5-dimethyl-11-phenyl-5H-benzo[b]naphtho[2,3-d]silol-8-yl)-9,9':10',9''-teranthracene 8. The product was obtained from the compound **6l** (199 mg, 0.48 mmol) and anthracene-9,10-diyldiboronic acid **7** (45 mg, 0.20 mmol) and was purified by column chromatography to give yellow solid (31 mg, 14%); mp: 255.9 – 256.4 ℃; TCL (petroleum ether): R_f = 0.5; ^{1}H NMR (400 MHz, $CDCl_3$) δ 8.51 (s, 2H), 8.19 (s, 2H), 8.05 (d, J = 8.5 Hz, 4H), 7.94 (s, 2H), 7.72 (d, J = 8.8 Hz, 4H), 7.67 – 7.54 (m, 11H), 7.50 (d, J = 7.1 Hz, 4H), 7.43 (dd, J = 17.7, 8.8 Hz, 8H), 7.35 – 7.28 (m, 5H), 7.18 (t, J = 7.2 Hz, 3H), 7.03 (t, J = 7.8 Hz, 3H), 6.61 (d, J = 8.2 Hz, 2H), 0.53 (s, 12H); ^{13}C NMR (101 MHz, $CDCl_3$) δ 148.6, 141.4, 141.2, 140.7, 139.0, 136.8, 136.2, 135.1, 134.5, 133.2, 132.7, 132.4, 131.4, 130.4, 130.2, 130.1, 129.8, 129.5, 128.4, 127.8, 127.0, 127.0, 126.9, 126.7, 126.6, 125.4, 125.2, -2.6; IR (KBr): 3713, 3653, 3447, 1714, 1642, 1516, 1288, 902, 754 cm^{-1}; HRMS-ESI (m/z): $[M+H]^+$ calcd. for $C_{90}H_{63}Si_2$, 1199.4463; found 1199.6145.

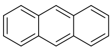

9

Anthracene 9 [9]. The product was obtained as white solid (19 mg, 53%); TCL (petroleum ether): R_f = 0.98; ^{1}H NMR (500 MHz, $CDCl_3$) δ 8.42 (s, 2H), 8.00 (dd, J = 6.4, 3.2 Hz, 4H), 7.45 (dd, J = 6.5, 3.1 Hz, 4H). ^{13}C NMR (101 MHz, $CDCl_3$) δ 131.7, 128.2, 126.3, 125.4.

VI. Photo-Physical Properties

1. General information

Ultraviolet-visible (UV-vis) absorption spectra were measured with Perkin-Elmer Lambda 950-PKA UV/vis spectrophotometer. Steady state photoluminescence (PL) and phosphorescence spectra at ambient temperature and 77 K were recorded using FluoroMax-4 spectrofluorometer. The solution was excited at 360 nm to detect luminescence. Also, photoluminescence quantum yield (PLQY) of the solution was measured with Qtsuka absolute PL quantum yield spectrometer (QE-2100).

2. Photophysical characterization of compound **8**

UV-vis absorption and PL spectra of compound **8** were measured in diluted toluene solution (10^{-5} M) (Supplementary Fig. 1).

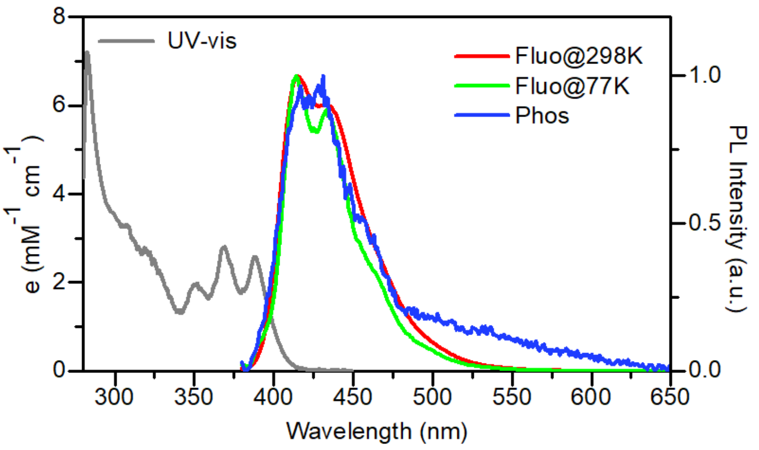

Supplementary Fig. 1 UV-vis absorption and PL spectra of benzosilole derivative **8** measured in diluted toluene solution (10^{-5} M) (a.u. refers to arbitrary unit. Fluo@298K refers to the fluorescence spectra measured at 298 K. Fluo@77K refers to the fluorescence spectra measured at 77 K. Phos refers to the phosphorescence spectra.).

3. Determination of triplet state energy level

Triplet state energies of [Ir{dt(tBu)$_2$ppy}$_2$(dtbbpy)][PF$_6$] (**PC1**), *ortho*-alkynylaryl vinylsilane (**1a**) and TsN$_3$ (**2a**) were evaluated to help with mechanism studies. All phosphorescence spectra were detected at 77 K with a delay time of 0.05 ms using FluoroMax-4 spectrofluorometer. According to the Phosphorescence spectra of **PC1**, *ortho*-alkynylaryl vinylsilane **1a** and **2a** to get the onset values (black lines shown in Supplementary Fig. 2), using the formula Eg (eV) =1240/λ (onset values) to get the lowest triplet energies of **PC1** (2.58 eV), **1a** (2.79 eV) and **2a** (3.10 eV), respectively.

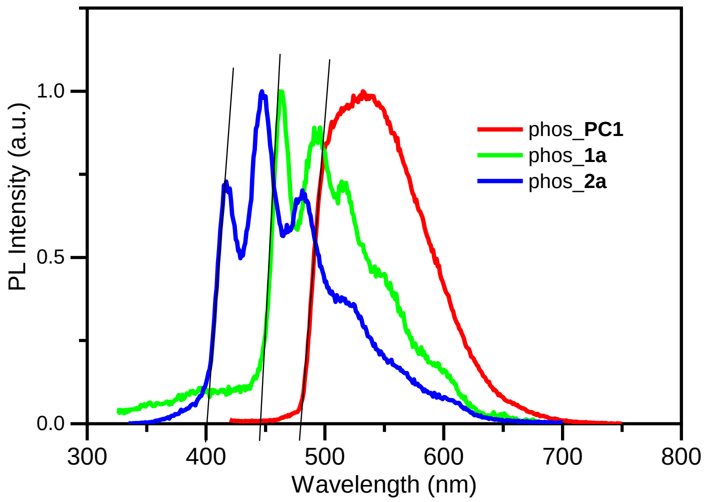

Supplementary Fig. 2 Phosphorescence spectra of **PC1**, triplet state energies of *ortho*-alkynylaryl vinylsilane **(1a)** and **TsN$_3$** (**2a**) measured in toluene solutions at 77 K with 0.05 ms delay time.

VII. Control Experiments for Mechanistic Investigation

1. Photocatalyzed cycloaromization of sulfonylhydrazine **10** with vinylsilane **1a**

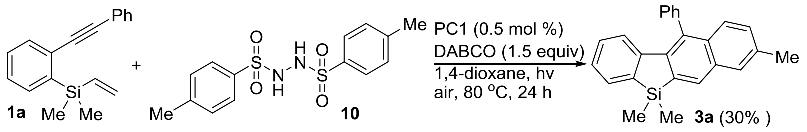

To a 10 mL vial equipped with a magnetic stir bar, was added vinylsilane **1a** (52 mg, 0.2 mmol), sulfonylhydrazine **10** (204 mg, 0.6 mmol), DABCO (34 mg, 0.3 mmol), **PC1** (1.3 mg, 0.001 mmol) and 1,4-dioxane (2.0 mL) under air conditions. The vial was equipped with a Teflon septum and stirred at 80 ℃ under blue LED irradiation with two Kessil LEDs (30 W, 456 nm, approximately 3 cm away from the reaction mixture) for 20 hours. The desired product **3a** was achieved in 30% yield.

2. The effect of the radical trapper TEMPO on the cycloaromatization of vinylsilane **1a** with 4-methylbenzenesulfonyl azide **2a**

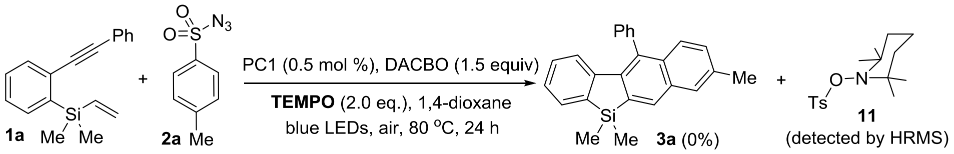

To a 10 mL vial equipped with a magnetic stir bar, was added vinylsilane **1a** (52 mg, 0.2 mmol), 4-methylbenzenesulfonyl azide **2a** (163 ul, 0.8 mmol), DABCO (34 mg, 0.3 mmol), **PC1** (1.2 mg, 0.001 mmol) and 1,4-dioxane (2.0 mL) under air conditions. Then the TEMPO (63 mg, 2.0 equiv), was added. The vial was equipped with a teflon septum and stirred at 80 ℃ under blue LED irradiation with two Kessil LEDs (30 W, 456 nm, approximately 3 cm away from the reaction mixture) for 24 hours. The corresponding reaction mixture was detected according to both TLC and HRMS. It is noteworthy that no desired product **3a** was detected. However, radical coupling product **11** was detected by high-resolution mass spectrometry, which means that sulfonyl radicals were involved in this transformation. HRMS-ESI (m/z): $[M+Na]^+$ calcd. for $C_{16}H_{25}NNaO_3S$, 334.1447; found 334.1448.

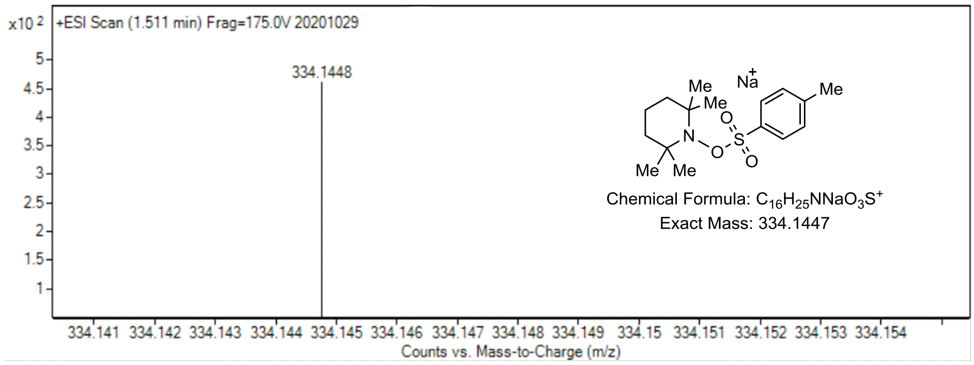

Supplementary Fig. 3 HR-MS of the radical coupling product **11**

3. Photocatalyzed coupling-cyclization of *ortho*-alkynylaryl vinylether **1u** with 4-chlorobenzenesulfonyl azide **2h**

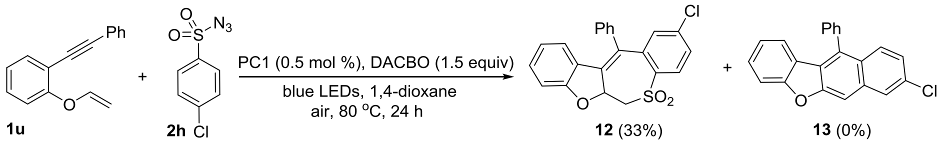

To a 10 mL vial equipped with a magnetic stir bar, was added *ortho*-alkynylaryl vinylether **1u** (44 mg, 0.2 mmol), 4-chlorobenzenesulfonyl azide **2h** (174 mg, 0.8 mmol), DABCO (34 mg, 0.3 mmol), **PC1** (1.2 mg, 0.001 mmol) and 1,4-dioxane (2.0 mL) under air conditions. The vial was equipped with a teflon septum and stirred at 80 ℃ under blue LED irradiation with two Kessil LEDs (30 W, 456 nm, approximately 3 cm away from the reaction mixture) for 48 hours. The solvent was removed in vacuo and the residue was purified by flash column chromatography on silica gel (Petroleum ether/EtOAc= 25:1) to obtain seven-membered cyclic benzo[b]thiepine 1,1-dioxide **12** (33%) instead of naphtho[2,3-b]benzofuran **13**.

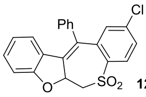

12

10-Chloro-12-phenyl-5a,6-dihydrobenzo[6,7]thiepino[3,4-b]benzofuran 7,7-dioxide 12. The product was obtained from *ortho*-alkynylaryl vinylether **1u** (44 mg, 0.2 mmol) and 4-chlorobenzenesulfonyl azide **2h** (174 mg, 0.8 mmol) and was purified by column chromatography to give yellow solid (26 mg, 33%); mp: 193.5 – 194.4 ℃; TCL (petroleum ether:ethyl acetate, 25:1 v/v): R_f = 0.5; ^{1}H NMR (400 MHz, $CDCl_3$) δ 8.12 (d, J = 8.4 Hz, 1H), 7.51 (t, J = 6.4 Hz, 3H), 7.46 (d, J = 6.7 Hz, 2H), 7.41 (d, J = 8.5 Hz, 1H), 7.21 (t, J = 7.8 Hz, 1H), 7.17 (s, 1H), 6.88 (d, J = 8.1 Hz, 1H), 6.72 (t, J = 7.6 Hz, 1H), 6.44 (d, J = 7.8 Hz, 1H), 5.46 (dd, J = 10.7, 7.1 Hz, 1H), 4.40 (dd, J = 13.2, 7.0 Hz, 1H), 4.04 – 3.88 (m, 1H); ^{13}C NMR (101 MHz, $CDCl_3$) δ 162.5, 142.9, 140.5, 138.5, 138.1, 136.9, 131.6, 130.6, 129.5, 129.3, 128.8, 127.6, 125.3, 123.1, 121.7, 111.2, 77.2, 69.3; IR (KBr): 3714, 3670, 3613, 1716, 1508, 1289, 750, 518, 428 cm^{-1}; HRMS-ESI (m/z): $[M+H]^+$ calcd. for $C_{22}H_{16}ClO_3S$, 395.0503; found 395.1511.

4. Photocatalyzed cycloaromatization of vinylsilane **1a** with *ortho*-methylphenylsulfonyl azide **2s**

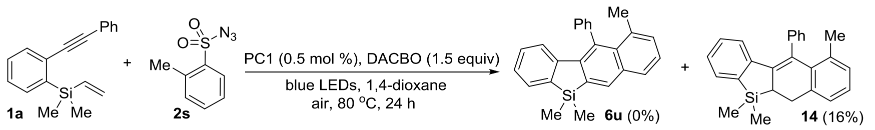

To a 10 mL vial equipped with a magnetic stir bar, was added vinylsilane **1a** (52 mg, 0.2 mmol), *ortho*-methylphenylsulfonyl azide **2s** (158 mg, 0.8 mmol), DABCO (34 mg, 0.3 mmol), **PC1** (1.3 mg, 0.001 mmol) and 1,4-dioxane (2.0 mL) under air. The vial was equipped with a teflon septum and stirred at 80 ℃ under blue LED irradiation with two Kessil LEDs (30 W, 456 nm, approximately 3 cm away from the reaction mixture) for 48 hours. The solvent was removed in vacuo and the residue was purified by flash column chromatography on silica gel (Petroleum ether) to afforded 13% yield of SO_2-containing thiepane 1,1-dioxide derivative **14** (0% for **6u**).

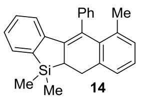

14

4,7,7-Trimethyl-12-phenyl-6a,7-dihydro-6H-benzo[b]benzo[4,5]silolo[3,2-e]thiepine5,5-dioxide 14. The product was obtained from vinylsilane **1a** (52 mg, 0.2 mmol) and *ortho*-methylphenylsulfonyl azide **2s** (158 mg, 0.8 mmol) and was purified by column chromatography to give white solid (11 mg, 16%); mp: 100.5 – 102.4 ℃; TCL (petroleum ether): R_f = 0.83; ^{1}H NMR (500 MHz, $CDCl_3$) δ 7.54 (d, J = 7.1 Hz, 1H), 7.41 – 7.30 (m, 5H), 7.10 (dd, J = 15.6, 7.9 Hz, 2H), 7.01 (t, J = 7.4 Hz, 1H), 6.94 (t, J = 7.7 Hz, 1H), 6.84 (dd, J = 13.3, 7.9 Hz, 2H), 2.84 (dd, J = 15.2, 4.0 Hz, 1H), 2.71 (t, J = 16.2 Hz, 1H), 2.05 (dd, J = 17.1, 3.9 Hz, 1H), 1.64 (s, 3H), 0.39 (s, 3H), 0.28 (s, 3H); ^{13}C NMR (101 MHz, $CDCl_3$) δ 147.8, 144.0,

143.1, 142.4, 141.3, 137.9, 136.9, 136.5, 132.6, 131.3, 130.9, 129.3, 128.7, 127.2, 126.9, 126.5, 126.5, 124.3, 32.3, 25.0, 23.8, -1.7, -4.2; IR (KBr): 3712, 3652, 3443, 1642, 1513, 1290, 956, 755, 429 cm^{-1}; HRMS-ESI (m/z): $[M+H]^+$ calcd. for $C_{25}H_{25}Si$, 353.1720; found 353.1715.

5. Cyclic voltammetry experiments

Cyclic voltammetry (CV) was performed on conventional three-electrode system at CHI660E electrochemical workstation (Chenhua, Shanghai, China) and a three-electrode set-up, composed of a glassy carbon working electrode, a Pt wire counter electrode, and the $Ag/AgNO_3$ reference electrode. The cyclic voltammograms was carried out in 0.1 M of Bu_4NClO_4/MeCN at a scan rate of 100 mV/s with argon and kept under an inert atmosphere. The concentration of the analyte was 2 mM. The final values are referenced to SCE by using ferrocene (Fc/Fc^+ couple) as the internal standard (+0.38 V in 0.1 M n-Bu_4NPF_6 in MeCN).[10]

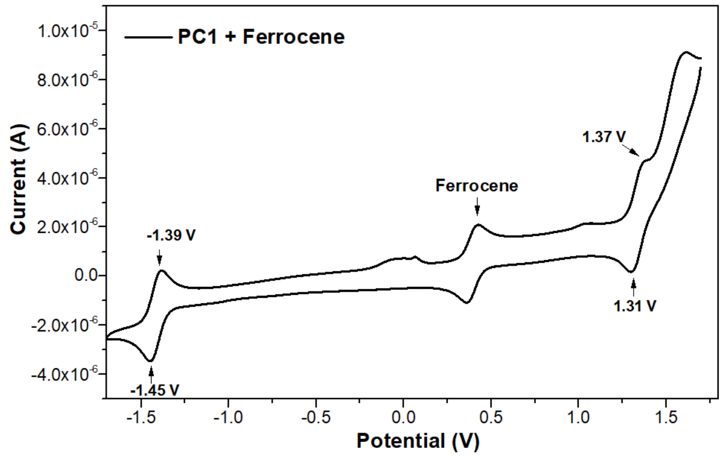

Supplementary Fig. 4 The CV of **PC1** (2 mM) in Bu_4NClO_4 (0.1 M) in MeCN using ferrocene as internal standard

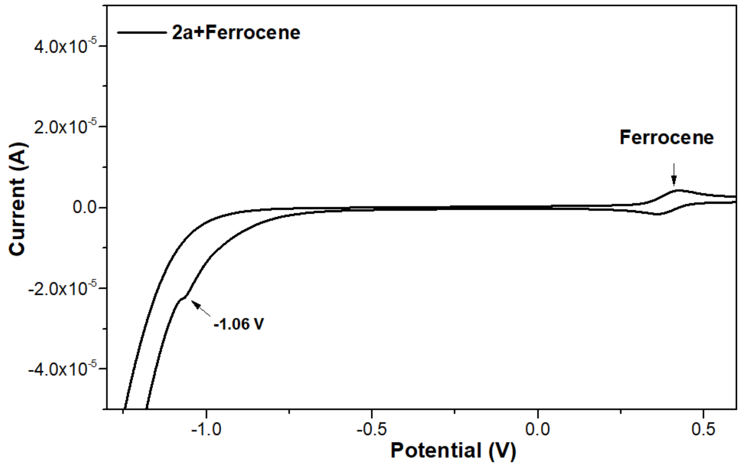

Supplementary Fig. 5 The CV of TsN_3 **2a** (2 mM) in Bu_4NClO_4 (0.1 M) in MeCN using ferrocene as internal standard

6. Stern-Volmer quenching studies

Fluorescence spectra were collected on Hitachi Fluorescence Spectrophotometer F-7000 for all experiments. The solution of **PC1** was excited at 400 nm and the emission intensity was collected at 420 nm. In a typical experiment, standard solutions of **PC1** and TsN_3 were prepared in dioxane.

The solution of **PC1** (1×10^{-5} M) and various concentrations of quencher TsN_3 were irradiated. The significant decrease of **PC1** luminescence could be observed in the presence of TsN_3.

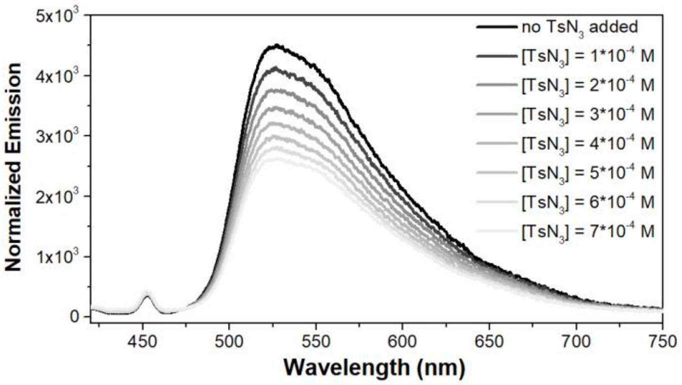

Supplementary Fig. 6 Luminescence quenching of **PC1** with various concentrations of TsN_3

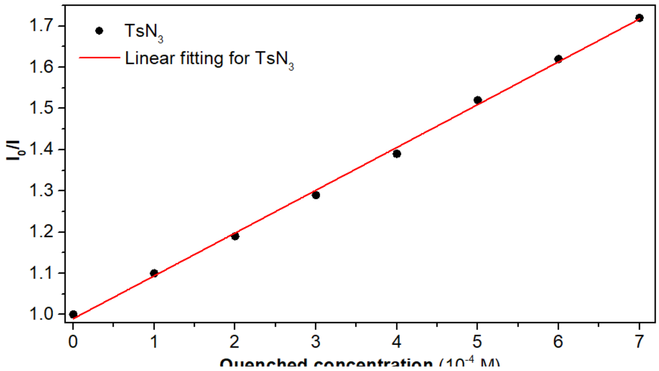

Supplementary Fig. 7 **PC1** emission quenching by TsN_3 at various concentrations

7. Determination of the structure of compound **14**

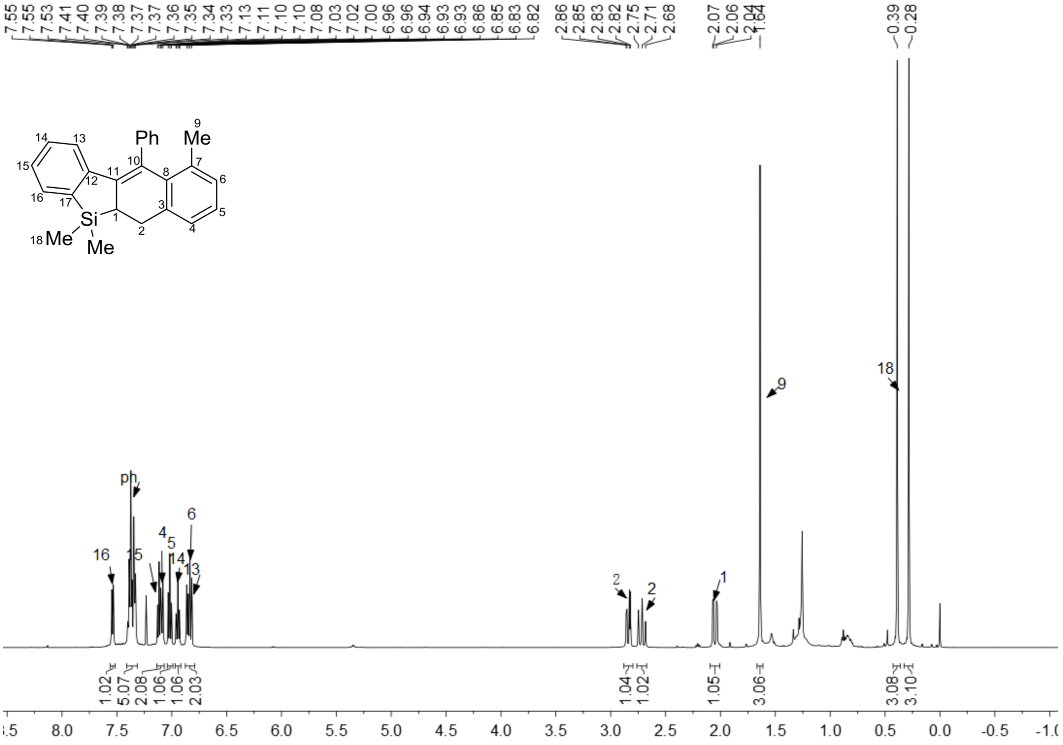

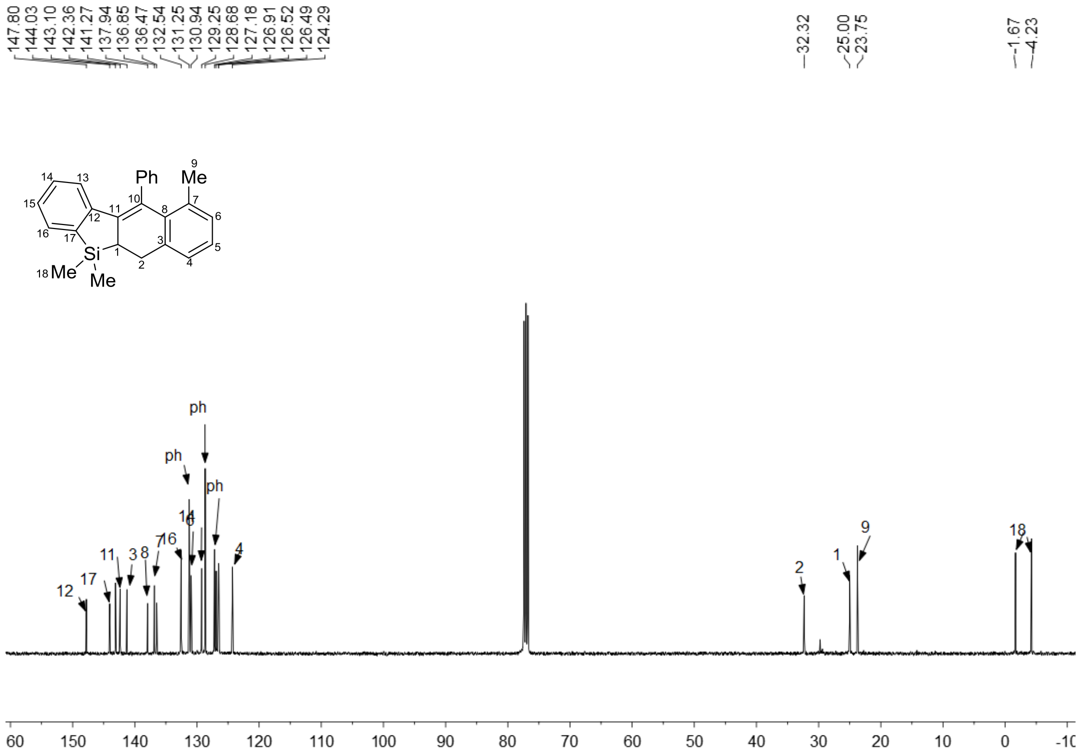

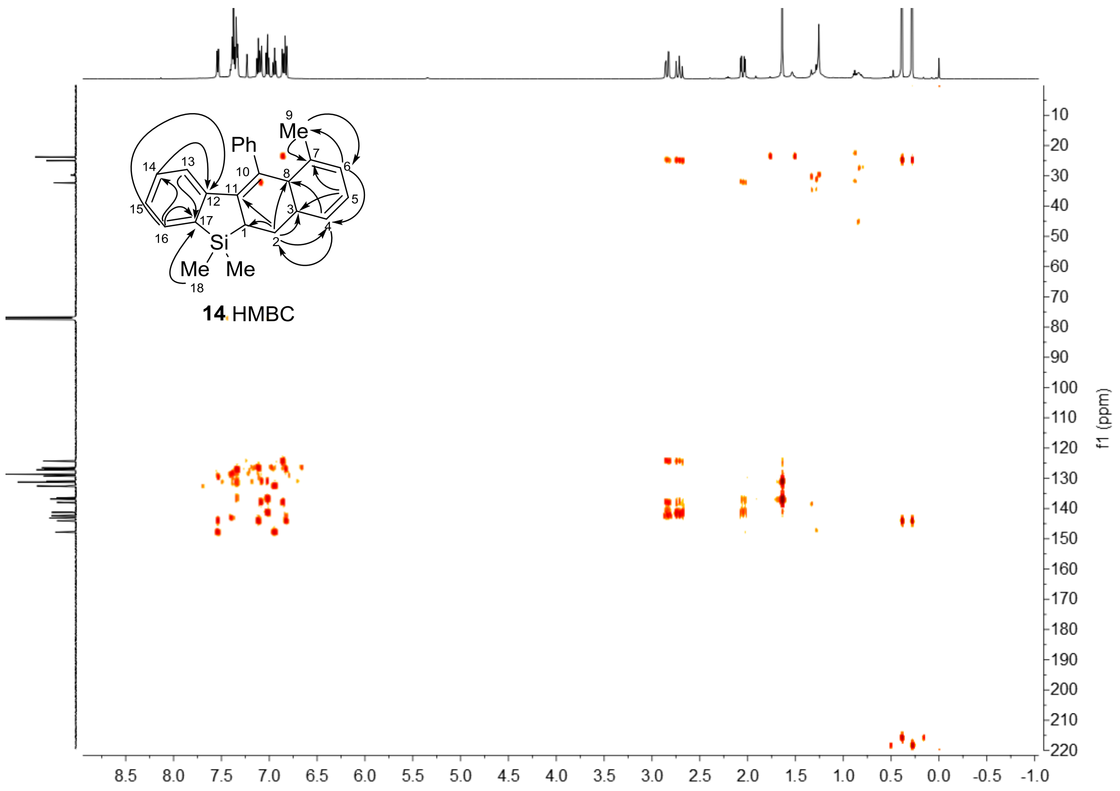

Supplementary Fig. 8 The ^{1}H NMR, ^{13}C NMR and HMBC spectrum of the compound **14**

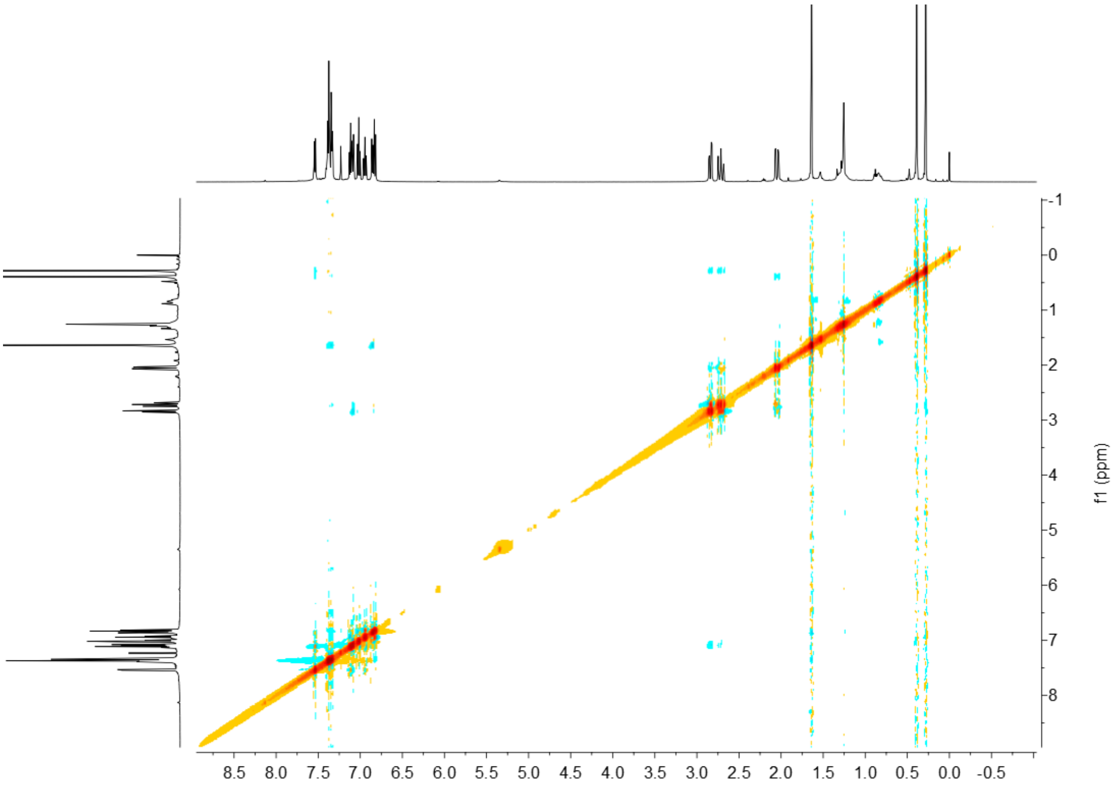

Supplementary Fig. 9 ^{1}H - ^{1}H NOE spectrum of the compound **14**

8. The possible mechanism for carbocyclization of allyldimethylsilane **4**

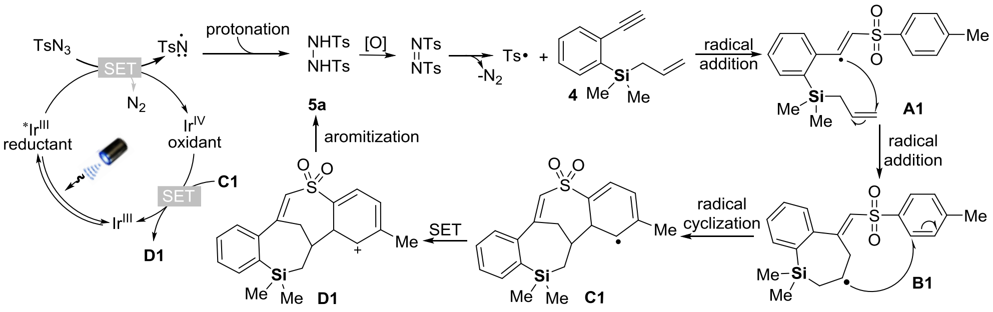

Supplementary Fig. 10 The possible mechanism for carbocyclization of allyldimethylsilane **4**

VIII. Computational details

1. Computational methods:

The geometries of all species were fully optimized using Truhlar's pure functional M06L[11] with the triple-zeta 6-311G (d,p) basis set. Harmonic vibrational frequencies were calculated at the same level of theory for the characterization of stationary points and for the zero-point energy corrections. The Gibbs free energy (*G*) was calculated using statistical thermodynamics with the rigid rotor harmonic oscillator approximation to the contributions of translation, rotation, and vibration. All the DFT calculations in the current work were carried out using the Gaussian09 programs.[12]

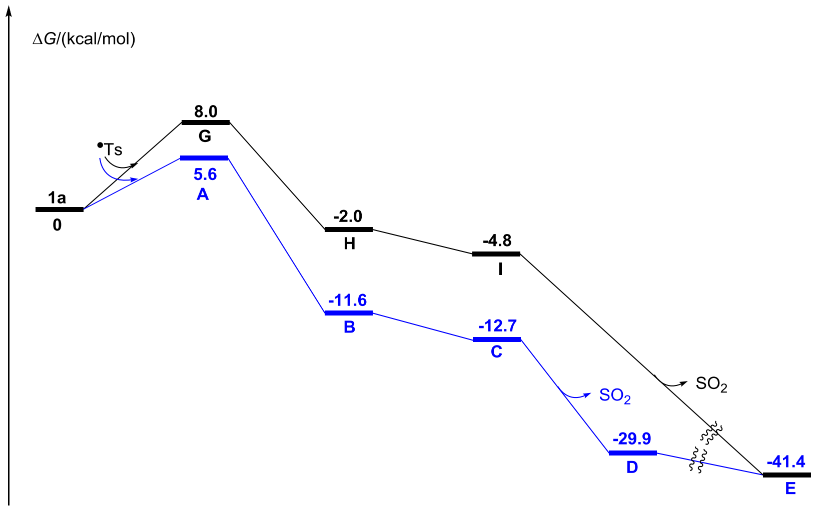

Supplementary Fig. 11 Calculated free energies for the cycloaromatization between vinylsilanes and arylsulfonyl azides at the M06L/6-311G(d,p) level of theory

2. Optimized cartesian coordinates

SO_2

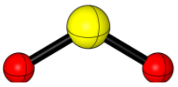

S	0.00000000	0.37217900	0.00000000
O	1.25913800	-0.37218000	0.00000000
O	-1.25913800	-0.37217800	0.00000000

1a

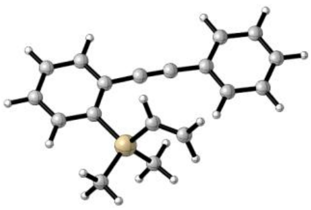

C	3.66670500	0.46143300	0.01642900
C	2.28402900	0.25586700	0.04537500
C	1.44846200	1.40387000	-0.02468200
C	2.01361400	2.68757000	-0.11796500
C	3.39168500	2.84950500	-0.14414200
C	4.22386600	1.73535000	-0.07771900
H	4.33104600	-0.39952700	0.06429000
H	1.35544400	3.55027200	-0.16907900
H	3.81654000	3.84675700	-0.21807700
H	5.30331800	1.85748500	-0.09999300
C	0.44313800	-1.79190500	-1.31360700
H	0.56115400	-1.16771900	-2.20243100
C	0.03817100	1.24780700	-0.00468000
C	-1.16498700	1.04923800	0.01538000
C	-2.55082100	0.75519500	0.04533900
C	-2.97594900	-0.57775200	0.20161100
C	-3.51997600	1.76691600	-0.07967600
C	-4.32924000	-0.88370300	0.22530900
H	-2.22560300	-1.35870400	0.29070900
C	-4.87131500	1.44927800	-0.05367100
H	-3.19630700	2.79683200	-0.19844700
C	-5.28150000	0.12600500	0.09730500
H	-4.64400700	-1.91695600	0.34336000
H	-5.61030400	2.23930700	-0.15311500
H	-6.33989400	-0.11749200	0.11539600
C	-0.49135100	-2.74712200	-1.35449900
H	-1.12301100	-2.92179400	-2.22428700
H	-0.67800000	-3.40823300	-0.50650600
Si	1.56646800	-1.48711900	0.15935600
C	2.99223200	-2.71329800	0.12455800
H	3.59354800	-2.62766500	-0.78535400
H	3.66126000	-2.58457100	0.98174000
H	2.60651300	-3.73679500	0.16261700
C	0.59657300	-1.72406400	1.75092000
H	0.10237800	-2.70158900	1.76894600
H	1.25025500	-1.67381300	2.62663800
H	-0.17490200	-0.95726800	1.86309600

A

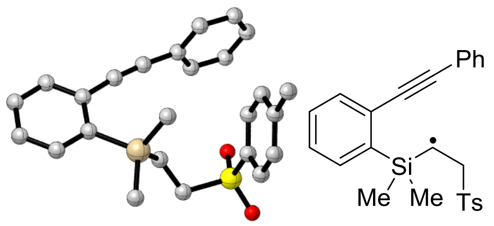

C	4.53107200	-1.23100300	-0.23835800
C	3.35257600	-0.47803100	-0.21680600
C	3.47776600	0.93609700	-0.17456600
C	4.74531800	1.53780700	-0.16120800
C	5.89323700	0.75596200	-0.18585800
C	5.78897900	-0.63120600	-0.22370400
H	4.46669800	-2.31747000	-0.26579300
H	4.81560800	2.62121000	-0.12723000
H	6.87012400	1.23148000	-0.17298900
H	6.68478500	-1.24563600	-0.24007800
C	0.72072600	-0.83022800	1.28175500
H	0.77839400	0.19443600	1.65396700
C	2.29750300	1.73001100	-0.12852700
C	1.22049500	2.29791900	-0.07193500
C	-0.07860000	2.86360100	0.03193100
C	-0.79142000	3.25569800	-1.11533900
C	-0.70143000	2.96776600	1.28838900
C	-2.09242200	3.73007400	-1.00265800
H	-0.31538600	3.16927400	-2.08834200
C	-2.00545400	3.43496100	1.38985600
H	-0.16418900	2.64730400	2.17576200
C	-2.70511000	3.81663200	0.24705000
H	-2.63513900	4.02711900	-1.89621300
H	-2.48193400	3.47723600	2.36454700
H	-3.72696600	4.17565900	0.32821500
C	-0.13750000	-1.72595300	2.08021800
H	0.09159200	-1.71168700	3.15448800
H	-0.14156800	-2.77014100	1.74962000
S	-1.90521800	-1.20799700	2.08127400
C	-2.31961300	-1.17770100	0.34516800
C	-2.36078500	0.04132500	-0.32468000
C	-2.59213600	-2.37359100	-0.31587900
C	-2.67439600	0.05613900	-1.67827700
H	-2.16457000	0.96054100	0.22008200
C	-2.89521300	-2.34036700	-1.67211500
H	-2.58796400	-3.31024800	0.23582000
C	-2.93434700	-1.12919400	-2.37430100
H	-2.70698200	1.00787300	-2.20546900
H	-3.11344600	-3.26740300	-2.19779400
C	-3.20658200	-1.10642200	-3.84596500
H	-3.76884800	-0.21666000	-4.14144600
H	-2.26827200	-1.09267100	-4.41345400
H	-3.76606900	-1.98723000	-4.17070700
O	-1.95620900	0.16982600	2.57931700
O	-2.66750800	-2.28517600	2.71881500
Si	1.65727100	-1.31450100	-0.25500800
C	1.88586000	-3.18081900	-0.23958200
H	0.91405800	-3.68432400	-0.29473200
H	2.46331400	-3.52035800	-1.10521000
H	2.39226900	-3.54131400	0.66113900
C	0.74440100	-0.86261600	-1.83513600
H	1.36309800	-1.08745600	-2.71010800
H	-0.18077100	-1.44328600	-1.91662300
H	0.47604200	0.19636200	-1.86962100

B

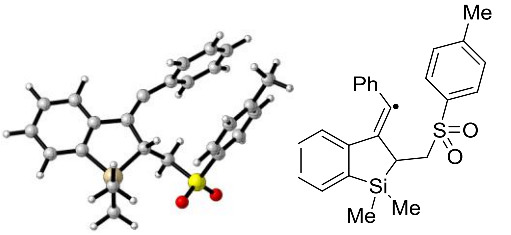

C	-5.02152100	0.47023800	0.65770100
C	-3.66197500	0.27568100	0.39607800
C	-2.85375700	1.40084400	0.12709300
C	-3.40934400	2.68714400	0.12604400
C	-4.76087400	2.85409900	0.38940500
C	-5.57181600	1.74757400	0.65697400
H	-5.66116100	-0.38634600	0.86775800
H	-2.77133300	3.54401400	-0.08208900
H	-5.19197000	3.85200400	0.38802100
H	-6.62958200	1.88849200	0.86301800
C	-1.04005300	-0.36156700	-0.07778600
H	-0.34164500	-0.48727600	0.76336000
C	-1.42548100	1.13147800	-0.11591400
C	-0.48434700	2.03743500	-0.30536500
C	0.86903300	2.34766900	-0.45840900
C	1.45726400	2.51639400	-1.74184400
C	1.69953500	2.53576900	0.68091700
C	2.79892800	2.83358300	-1.86941000
H	0.83339300	2.38640200	-2.62257800
C	3.03606800	2.86272700	0.53465400

H	1.26392900	2.41020000	1.66840000
C	3.59719100	3.01380800	-0.73642100
H	3.23040500	2.94778400	-2.86013600
H	3.65358400	2.99697600	1.41953400
H	4.64778800	3.27058600	-0.84300700
C	-0.31840300	-0.79708000	-1.34658400
H	0.39146400	-0.04636000	-1.71543800
H	-0.99242500	-1.05162500	-2.17256900
S	0.67074000	-2.27960900	-1.06751700
C	2.01097300	-1.63111800	-0.08293200
C	1.98286900	-1.76452200	1.30270900
C	3.04243300	-0.94334300	-0.71884800
C	3.00474700	-1.19838700	2.05628600
H	1.16850800	-2.31253100	1.76962800
C	4.05586300	-0.38682000	0.04893900
H	3.04727200	-0.85610500	-1.80233900
C	4.05196400	-0.50159600	1.44361000
H	2.99046400	-1.29452100	3.13969200
H	4.86108400	0.15786300	-0.43847200
C	5.15319400	0.09941400	2.26077300
H	4.77981400	0.50701900	3.20466000
H	5.91233300	-0.64834500	2.51649300
H	5.66180400	0.90308200	1.72099700
O	1.20988300	-2.71206500	-2.35801300
O	-0.11240100	-3.18962200	-0.21862100
Si	-2.65691600	-1.30556300	0.31800200
C	-2.56545100	-2.22271800	1.94784900
H	-1.89688800	-3.08320100	1.85473600
H	-2.18684200	-1.57933500	2.74767400
H	-3.54708600	-2.59202600	2.26050400
C	-3.22664600	-2.43169600	-1.06652000
H	-3.31235200	-1.89772200	-2.01780900
H	-2.53319300	-3.26669100	-1.19927400
H	-4.21458500	-2.84728600	-0.84342100

C

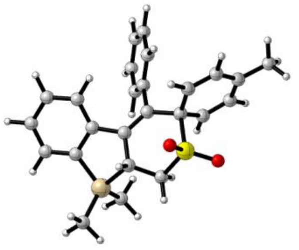

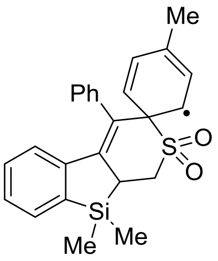

C	4.53691300	0.83419900	-0.28209300
C	3.34721900	0.12422300	-0.10711200
C	2.12674000	0.67316200	-0.56683100
C	2.13960800	1.90794700	-1.23135800
C	3.33406100	2.59449100	-1.40225900
C	4.53445100	2.06955500	-0.92106800
H	5.47767500	0.41558500	0.07219600
H	1.21878100	2.32516100	-1.62754000
H	3.33248700	3.54596300	-1.92745200
H	5.46306600	2.61634200	-1.06160000
C	1.31483300	-1.64069200	-0.27780900
H	1.51651000	-2.02023900	-1.29059200
C	0.92436100	-0.17655100	-0.36938900
C	-0.33267100	0.31725500	-0.20412400
C	-0.55144300	1.75761600	0.11289000
C	0.08582900	2.29778500	1.24033600
C	-1.38502700	2.59773200	-0.63682800
C	-0.08820500	3.62849100	1.59788000
H	0.73161300	1.65282700	1.83284700
C	-1.55588600	3.93261300	-0.28254800
H	-1.88549000	2.20918900	-1.51837000
C	-0.90953600	4.45445700	0.83420500
H	0.41702000	4.02107800	2.47631000
H	-2.19568500	4.56915400	-0.88760200
H	-1.04690100	5.49644300	1.10955800
C	0.28881100	-2.53332300	0.37621700
H	0.07468400	-2.25534400	1.41518700
H	0.55643400	-3.59467300	0.35076000
S	-1.26953300	-2.37459200	-0.50131800
C	-1.63914100	-0.48385900	-0.16217800
C	-2.55723800	-0.17242700	-1.29186500
C	-2.30431200	-0.39295700	1.16030100
C	-3.90543600	-0.05367000	-1.11118900
H	-2.11941900	-0.14698800	-2.28566900
C	-3.65584600	-0.24766300	1.28997600
H	-1.66868400	-0.45779500	2.04174000
C	-4.50897000	-0.10514200	0.16985300
H	-4.54072700	0.11032600	-1.98009400
H	-4.09321000	-0.21547600	2.28630100
C	-5.98921100	-0.01653100	0.32937700
H	-6.44588600	0.60511500	-0.44689700
H	-6.46000900	-1.00584000	0.25710900
H	-6.26840200	0.39484500	1.30393900
O	-2.30126700	-3.17913700	0.15786500
O	-0.99402400	-2.53313800	-1.93538300

Si	3.00592100	-1.55974300	0.60832600
C	4.19664100	-2.91397200	0.10448400
H	3.83905700	-3.89958300	0.41889900
H	4.33519400	-2.94014700	-0.97964600
H	5.18132300	-2.77097200	0.55995600
C	2.74146600	-1.54106600	2.46742800
H	1.97054200	-0.81134700	2.73817800
H	2.42098300	-2.51834400	2.84320000
H	3.65292800	-1.26725900	3.00617500

D

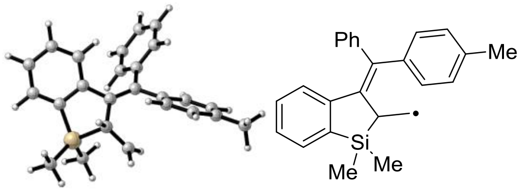

C	3.38530100	1.76738500	-1.62383800
C	4.47030600	0.99547800	-1.20099300
C	4.24203400	-0.19091800	-0.51187000
C	2.93957400	-0.60720100	-0.22977000
C	1.84029800	0.18548000	-0.64220400
C	2.08306900	1.37175400	-1.35261300
H	3.56030700	2.68286700	-2.18316200
H	5.48507800	1.31540400	-1.42248500
H	5.08861400	-0.80029600	-0.19839300
H	1.24955600	1.97393700	-1.70414100
C	0.54845900	-1.89098400	-0.28821100
C	-0.49886600	-2.63557400	0.41250100
C	-0.61918800	0.39091200	-0.13004200
C	0.49770900	-0.37164900	-0.36757300
H	-0.75219900	-3.64999400	0.12253200
Si	2.25402400	-2.13684400	0.57778500
C	2.04285900	-1.97937000	2.43431000
H	1.52488600	-2.85225400	2.84435300
H	3.00112400	-1.88390800	2.95271600
H	1.44322800	-1.09637600	2.67720100
C	3.08924200	-3.75051100	0.12255700
H	3.20793300	-3.84830500	-0.95996000
H	4.08390000	-3.82893700	0.57224800
H	2.50763700	-4.60791700	0.47558900
C	-1.98901800	-0.15384900	-0.18817200
C	-2.35341900	-1.12076400	-1.13903700
C	-2.99152400	0.30117300	0.68342600
C	-3.65010700	-1.61417000	-1.20463600
C	-4.28319800	-0.20332600	0.62264400
H	-2.74453300	1.06001700	1.42214800
C	-4.63892400	-1.17399000	-0.32035300
H	-3.90582600	-2.35192100	-1.96319200
H	-5.03566100	0.16438900	1.31787700
C	-6.02839500	-1.73063500	-0.37110100
H	-6.25659000	-2.15834000	-1.35129400
H	-6.16757700	-2.52943300	0.36718300
H	-6.78065400	-0.96675200	-0.15299200
H	-0.95343000	-2.24156100	1.31623300
H	-1.60857600	-1.45912800	-1.85490400
C	-0.51702500	1.83114400	0.20537100
C	0.41066200	2.29227800	1.15200500
C	-1.37471200	2.77151200	-0.38624800
C	0.49727400	3.64095200	1.47275700
H	1.06856400	1.57505100	1.63756700
C	-1.28508800	4.12262800	-0.07062400
H	-2.11319100	2.42854700	-1.10731900
C	-0.34654800	4.56473200	0.85873400
H	1.22075300	3.97295000	2.21273200
H	-1.95266000	4.83329500	-0.55091200
H	-0.28003800	5.61940500	1.11108400
H	0.72836200	-2.30983100	-1.29180400

E

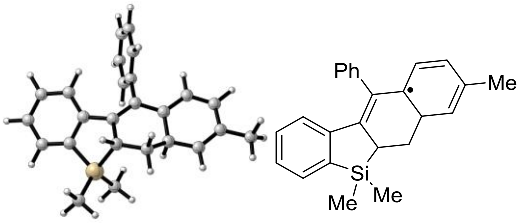

C	4.05807500	-1.09537000	0.20115300
C	2.67935000	-1.16728100	0.01168500
C	1.97267300	-0.02453700	-0.45563600
C	2.68174400	1.15230100	-0.73946000
C	4.05899500	1.19356500	-0.55244700
C	4.75225800	0.08016900	-0.07878500
H	4.60327800	-1.96516100	0.56600200
H	2.16746900	2.02727100	-1.12546300
H	4.60072900	2.10609700	-0.78796400
H	5.82868700	0.12895500	0.06266800
C	0.22623700	-1.69316900	-0.91218300
H	0.70230800	-1.89130100	-1.88687100
C	0.52280300	-0.24971000	-0.58625800

C	-0.47601800	0.65956600	-0.29548700
C	-0.15530100	2.08801600	-0.02268900
C	-0.40115900	3.07825200	-0.97990800
C	0.39711000	2.46595600	1.20544500
C	-0.09154800	4.41105200	-0.72044000
H	-0.83337400	2.79160000	-1.93635600
C	0.71221300	3.79585000	1.46455100
H	0.59216100	1.70010100	1.95335000
C	0.47041300	4.77256800	0.50129300
H	-0.28545600	5.16763200	-1.47611400
H	1.14748700	4.07102500	2.42168200
H	0.71597700	5.81141600	0.70345900
C	-1.25206900	-2.04003800	-1.01575200
H	-1.62624900	-1.80039600	-2.02047200
H	-1.42141900	-3.11721600	-0.87856700
Si	1.44930600	-2.55756600	0.26509400
C	0.79766200	-2.64189100	2.02559300
H	-0.05704300	-3.32238400	2.10473100
H	1.56074500	-2.99274800	2.72673400
H	0.46919700	-1.65330200	2.36304700
C	2.03003600	-4.24322800	-0.31587500
H	1.22747200	-4.98568700	-0.26303600
H	2.38397800	-4.20818800	-1.34996900
H	2.85362700	-4.61780100	0.30046100
C	-1.83340200	0.22963800	-0.13250200
C	-2.08713500	-1.25451500	-0.00062000
C	-2.90331300	1.11297700	0.02799100
C	-3.53272700	-1.62829000	-0.02957900
H	-1.69047000	-1.54433300	0.99648800
C	-4.21336200	0.67247600	0.14484500
H	-2.70410000	2.18215700	0.02319700
C	-4.54011700	-0.72179800	0.06716500
H	-3.76419500	-2.69190800	-0.09556200
H	-5.01845500	1.39701700	0.24817400
C	-5.97981200	-1.13306100	0.08312000
H	-6.09225900	-2.21742800	0.00654400
H	-6.47587500	-0.80882800	1.00513300
H	-6.53614100	-0.67766300	-0.74392500

F

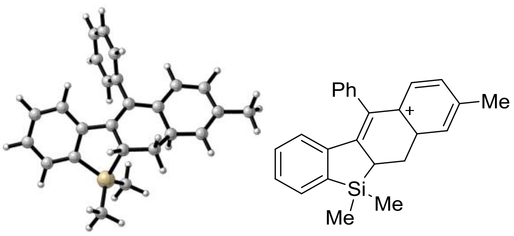

C	4.04627500	-1.13769100	0.18716300
C	2.66657900	-1.20225700	0.01156600
C	1.97124000	-0.05090700	-0.45739100
C	2.67356400	1.13174800	-0.74400200
C	4.05090100	1.15913600	-0.57830900
C	4.73644100	0.03679700	-0.10782900
H	4.59384500	-2.00209700	0.55571900
H	2.15877800	2.00465400	-1.13125900
H	4.60087500	2.06213000	-0.82379500
H	5.81310100	0.08146700	0.02653100
C	0.21646500	-1.68095000	-0.91401700
H	0.74727400	-1.91337400	-1.84828500
C	0.53194800	-0.27583000	-0.58117100
C	-0.46168700	0.66119500	-0.26928200
C	-0.12818600	2.09009300	-0.01458200
C	-0.37091500	3.06218600	-0.99059100
C	0.43261800	2.47312000	1.20802700
C	-0.04725400	4.39527300	-0.74992100
H	-0.80596800	2.76786400	-1.94338400
C	0.75604100	3.80496300	1.44592900
H	0.62360600	1.71946000	1.96938000
C	0.51943800	4.76706000	0.46659600
H	-0.23523300	5.14230400	-1.51494900
H	1.19184300	4.09239600	2.39804200
H	0.77325800	5.80593100	0.65305200
C	-1.25310500	-2.03799900	-1.04380100
H	-1.60400100	-1.82675800	-2.06299300
H	-1.42312200	-3.10847100	-0.88173900
Si	1.43324500	-2.58071300	0.30952400
C	0.72008500	-2.58335400	2.04062800
H	-0.14915900	-3.24548700	2.11234500
H	1.45095100	-2.93812700	2.77251500
H	0.41407800	-1.57866500	2.35066700
C	1.93163900	-4.27688800	-0.27386400
H	1.09140600	-4.97696900	-0.24857900
H	2.32314900	-4.25825100	-1.29409200
H	2.71098000	-4.69665400	0.36947700
C	-1.79474000	0.22485200	-0.10215100

C	-2.10983000	-1.24006900	-0.06039400
C	-2.85262100	1.12639300	0.15064400
C	-3.55064800	-1.59405200	-0.11481500
H	-1.79125800	-1.54677700	0.95915700
C	-4.15368500	0.69158800	0.21437700
H	-2.63425400	2.18631300	0.23656000
C	-4.54324700	-0.68089400	0.03831800
H	-3.79018000	-2.64999200	-0.22977800
H	-4.94000600	1.42749700	0.37276900
C	-5.99370000	-1.03792300	0.03340100
H	-6.14573400	-2.10868000	-0.11063600
H	-6.47278600	-0.75258500	0.97525500
H	-6.52857800	-0.51068700	-0.76248000

G

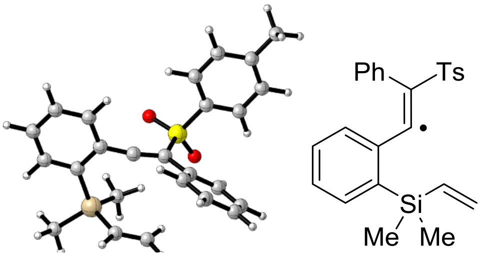

C	-4.21783400	-1.91596600	-0.72463400
C	-3.23766000	-0.99213300	-0.37087300
C	-1.91618600	-1.20196900	-0.89857800
C	-1.65929200	-2.31316300	-1.75166200
C	-2.66973500	-3.19984600	-2.06710300
C	-3.95653100	-3.00867200	-1.55644200
H	-5.22728400	-1.78374800	-0.33911600
H	-0.65425500	-2.45279400	-2.14112100
H	-2.45836800	-4.04861000	-2.71162500
H	-4.75179800	-3.70565600	-1.80588400
C	-3.15526800	2.06113200	-0.11493600
H	-3.71802100	2.35107100	-1.00637200
C	-0.89505700	-0.31786300	-0.62241700
C	0.14163200	0.31014800	-0.13434600
C	0.72292500	1.55877700	-0.66409400
C	0.52554000	1.85868100	-2.02096100
C	1.41895200	2.47808100	0.13224700
C	1.00496600	3.04121800	-2.56547200
H	-0.01859000	1.14779800	-2.63858500
C	1.90072300	3.66102700	-0.42072300
H	1.54932200	2.28242500	1.19121900
C	1.69842800	3.94892800	-1.76661200
H	0.83983300	3.25434500	-3.61804000
H	2.43074500	4.36717900	0.21275500
H	2.07594800	4.87483500	-2.19115000
C	-2.16787300	2.87219100	0.27924600
H	-1.90593000	3.79021500	-0.24709100
H	-1.55023600	2.64416900	1.14916000
Si	-3.62488900	0.47474100	0.76052700
C	-2.68789700	0.33498600	2.37709100
H	-3.09562200	1.03099400	3.11720600
H	-2.75931300	-0.67488600	2.79060200
H	-1.62227300	0.55838200	2.27436600
C	-5.47696300	0.45806200	1.08151500
H	-5.76220100	1.30697400	1.70942300
H	-6.05832200	0.52623400	0.15672400
H	-5.79058800	-0.45098900	1.60464300
S	0.89380800	-0.50262900	1.34186200
C	2.55519700	-0.85468000	0.80572700
O	0.19204200	-1.77247600	1.52269100
O	0.96770100	0.47900200	2.43377900
C	2.77914300	-1.96858100	-0.00177500
C	3.60193900	-0.01999100	1.18473200
C	4.07008900	-2.24269200	-0.43037500
H	1.94768600	-2.61820000	-0.26276900
C	4.88900800	-0.31187300	0.74911600
H	3.40300900	0.82897900	1.83158600
C	5.14319200	-1.41871000	-0.06687100
H	4.25667500	-3.11648100	-1.05072800
H	5.71573800	0.32694500	1.05130300
C	6.52760100	-1.70843600	-0.55743400
H	6.71586300	-2.78322800	-0.62688100
H	6.68554600	-1.29315300	-1.55935200
H	7.28778700	-1.27126800	0.09460200

H

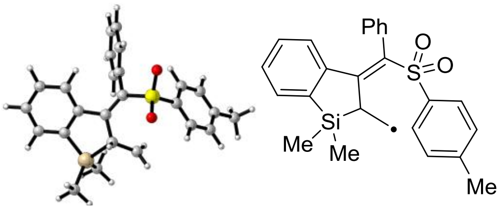

C	-4.70557400	0.29086000	0.61413600
C	-3.39165400	0.67434500	0.33450500
C	-2.47447100	-0.27317300	-0.17933000
C	-2.91766700	-1.58452800	-0.42410500
C	-4.22613700	-1.94487400	-0.13860800
C	-5.12438800	-1.01518200	0.38931300

H	-5.41194300	1.02090400	1.00611500
H	-2.24722400	-2.31741600	-0.85939200
H	-4.55569000	-2.96008400	-0.34225200
H	-6.14728400	-1.30941400	0.60864400
C	-1.09161600	1.75558100	-0.66544700
H	-1.37433700	2.04745100	-1.68596700
C	-1.13037000	0.23830500	-0.54025100
C	-0.05239400	-0.58993500	-0.70006200
C	0.05421400	-1.92118200	-0.06602300
C	0.30002900	-3.11799900	-0.75853700
C	-0.10129000	-1.98042800	1.33015800
C	0.38563900	-4.32368700	-0.07118900
H	0.41597700	-3.09371300	-1.83479600
C	-0.02006000	-3.18771200	2.01163900
H	-0.28417900	-1.05439300	1.87214300
C	0.22701700	-4.36657100	1.31203300
H	0.57080600	-5.24029700	-0.62471600
H	-0.14172400	-3.20696100	3.09122300
H	0.29508300	-5.31284200	1.84166400
C	0.13652400	2.44305200	-0.24519000
H	0.47831100	3.34114200	-0.74693700
H	0.65399700	2.13815000	0.66063600
Si	-2.54972900	2.32724500	0.45626500
C	-1.93747300	2.70800900	2.18710600
H	-1.30715900	3.60253700	2.19289000
H	-2.76592300	2.87599300	2.88174100
H	-1.33953400	1.87976200	2.58043600
C	-3.50033200	3.77878900	-0.24721300
H	-2.86520500	4.66765000	-0.31287900
H	-3.87966600	3.56443900	-1.24982600
H	-4.35571200	4.04032300	0.38324400
S	1.35530400	-0.11154300	-1.73371100
C	2.67317800	0.35051400	-0.62218600
O	1.00846900	1.05725500	-2.54524900
O	1.79165300	-1.34677700	-2.39564200
C	3.20856000	-0.59834600	0.24860300
C	3.18686200	1.64302500	-0.67476300
C	4.25030400	-0.22969300	1.09056600
H	2.81966800	-1.61345900	0.25838800
C	4.23892700	1.98996100	0.16382800
H	2.75425500	2.35203400	-1.37384300
C	4.78316900	1.06445700	1.06083500
H	4.66794800	-0.96126400	1.77862000
H	4.64820600	2.99729100	0.12443700
C	5.93448600	1.43861100	1.94234900
H	5.92999500	0.87292500	2.87781300
H	6.89174100	1.23181400	1.44986500
H	5.92704600	2.50366600	2.18853900

I

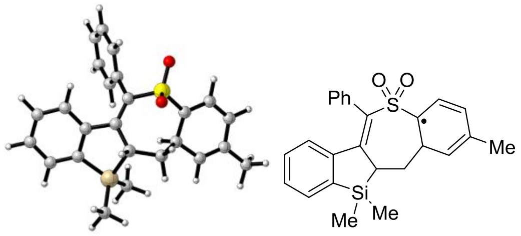

C	-3.61061400	2.58046500	0.41257300
C	-2.33977500	2.08698900	0.11131600
C	-2.18893900	0.74747400	-0.31712300
C	-3.33022900	-0.05474800	-0.47256300
C	-4.58506900	0.45771500	-0.17587100
C	-4.73193900	1.76966400	0.27906800
H	-3.72919300	3.61088300	0.74422700
H	-3.23958300	-1.07136900	-0.84212900
H	-5.46240900	-0.16906400	-0.31039200
H	-5.72042800	2.15760000	0.51020000
C	0.04800100	1.51447800	-1.05474700
H	-0.26627400	1.82419500	-2.06340200
C	-0.80706800	0.31955400	-0.66827200
C	-0.42593500	-0.98850000	-0.58035600
C	-1.14679800	-2.03124200	0.17382200
C	-1.53316900	-3.24968200	-0.40246400
C	-1.45958700	-1.79747100	1.52132300
C	-2.23465000	-4.18964800	0.34344900
H	-1.28094400	-3.45186300	-1.43804700
C	-2.15814700	-2.73932400	2.26520700
H	-1.15198200	-0.85719000	1.97514700
C	-2.55257200	-3.93839500	1.67617200
H	-2.53468200	-5.12541900	-0.12003100
H	-2.39162000	-2.53917200	3.30725300
H	-3.09967100	-4.67722000	2.25519400
C	1.58555900	1.48759100	-1.01037600
H	2.01310700	1.18540800	-1.96947600
H	1.93825100	2.51593400	-0.83957100
Si	-0.66950700	2.89753600	0.05899400
C	0.19656300	2.96635400	1.72549400
H	1.26498000	3.18261600	1.61443700
H	-0.22744900	3.75179500	2.35856400

H	0.10368900	2.02233400	2.27117600
C	-0.61373700	4.58127600	-0.76039000
H	0.41699700	4.90904900	-0.93075700
H	-1.12395800	4.57370400	-1.72737700
H	-1.09269800	5.34422300	-0.13877200
S	1.10004500	-1.51653900	-1.36976900
C	2.34875700	-0.82430200	-0.33087500
O	1.16173900	-0.86125900	-2.68423500
O	1.20594200	-2.97561700	-1.28730700
C	2.18310500	0.59900700	0.10386700
C	3.44715200	-1.58091600	-0.01412100
C	3.44997100	1.14114600	0.68335900
H	1.43021000	0.60516900	0.92383300
C	4.53669700	-1.00894700	0.66048600
H	3.46716200	-2.62225900	-0.32375100
C	4.54757300	0.38124600	0.97302200
H	3.45751800	2.20553700	0.92082600
H	5.40283800	-1.61900200	0.90491500
C	5.76861000	0.97841000	1.60234700
H	5.65630800	2.05282400	1.76564600
H	5.98303700	0.51304500	2.57055100
H	6.65516300	0.82322400	0.97838600

3-1a

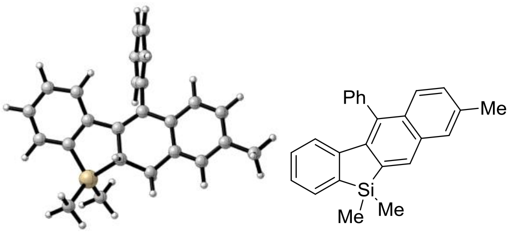

C	4.20940500	-0.52160000	-0.32491000
C	2.85142300	-0.80722900	-0.17938500
C	1.88257000	0.20852500	-0.39759000
C	2.32575300	1.48493900	-0.78933100
C	3.68252800	1.74357400	-0.93268300
C	4.63284000	0.75066400	-0.69432700
H	4.94833300	-1.30411800	-0.15633400
H	1.61590000	2.27412900	-1.00869500
H	4.00307400	2.73392700	-1.24594700
H	5.69152300	0.96778000	-0.80970200
C	0.31704900	-1.73381600	-0.35367200
H	0.23794200	-1.98521600	-1.42605600
C	0.47777300	-0.22564000	-0.25199300
C	-0.62096600	0.56584600	-0.06336400
C	-0.49984800	2.02730600	0.17449100
C	0.04732000	2.48924600	1.37694300
C	-0.89272400	2.97060300	-0.78426600
C	0.21037400	3.85047300	1.61116300
H	0.36212700	1.76207500	2.12199300
C	-0.72973400	4.33321600	-0.55167300
H	-1.30450400	2.62379000	-1.72944200
C	-0.17585500	4.77752700	0.64667500
H	0.64205700	4.18896600	2.54923300
H	-1.03044700	5.04963600	-1.31170000
H	-0.04641300	5.84106200	0.82711100
C	-0.92035500	-2.23178800	0.37574300
H	-0.74696100	-2.16034800	1.46236100
H	-1.09966100	-3.29478700	0.17140300
C	-1.97188500	-0.02111000	-0.09049700
C	-3.13033300	0.75508500	-0.24571900
C	-2.13357000	-1.41770000	0.04758100
C	-4.38837000	0.16915400	-0.31929100
H	-3.04829100	1.83561700	-0.31087700
C	-3.39792600	-1.98612800	-0.03104200
C	-4.54784000	-1.21402900	-0.22777300
H	-5.26594700	0.79995800	-0.44756900
H	-3.49366400	-3.06646500	0.08155200
C	-5.89716300	-1.85384900	-0.33877800
H	-6.70081400	-1.13198000	-0.17026500
H	-6.05594600	-2.28656000	-1.33376000
H	-6.02091700	-2.66957600	0.38029400
Si	1.98962100	-2.40593700	0.24006000
C	2.61481300	-3.90448700	-0.69916300
H	1.99177800	-4.78439800	-0.50991600
H	2.62184400	-3.72639800	-1.77798200
H	3.63568900	-4.16472900	-0.40211800
C	1.95126200	-2.76143300	2.08495600
H	1.56401700	-1.90210000	2.64167700
H	1.31492200	-3.62164900	2.31750500
H	2.95042700	-2.97959100	2.47381200

IX. Single Crystal Structure and Crystallographic Data

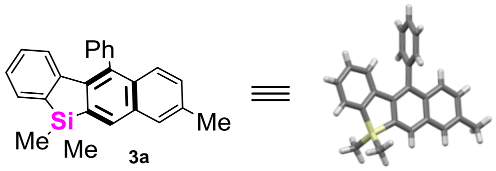

Supplementary Fig. 12 The crystal structure of **3a** (the ellipsoid contour probability level is 30%)

Supplementary Table 7 Crystal data and structure refinement for **3a**

Identification code	**3a**
Empirical formula	$C_{25}H_{22}Si$
Formula weight	350.51
Temperature/K	100.01(10)
Crystal system	orthorhombic
Space group	$P2_12_12_1$
a/Å	8.1584(5)
b/Å	8.9348(6)
c/Å	26.9612(16)
α/°	90
β/°	90
γ/°	90
Volume/Å^3	1965.3(2)
Z	4
ρ_{calc}g/cm^3	1.185
μ/mm^{-1}	0.124
F(000)	744.0
Crystal size/mm^3	0.14 × 0.13 × 0.12
Radiation	Mo Kα (λ = 0.71073)
2Θ range for data collection/°	4.802 to 49.998
Index ranges	$-7 \le h \le 9$, $-10 \le k \le 7$, $-26 \le l \le 32$
Reflections collected	6192
Independent reflections	3436 [R_{int} = 0.0393, R_{sigma} = 0.0692]
Data/restraints/parameters	3436/0/238
Goodness-of-fit on F^2	1.072
Final R indexes [I>=2σ (I)]	R_1 = 0.0521, wR_2 = 0.1242
Final R indexes [all data]	R_1 = 0.0589, wR_2 = 0.1308
Largest diff. peak/hole / e Å^{-3}	0.60/-0.30
Flack parameter	-0.18(14)

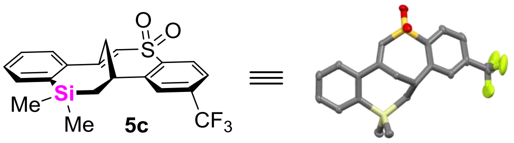

Supplementary Fig. 13 The single crystal structure of **5c** (the ellipsoid contour probability level is 30%)

Supplementary Table 8 Crystal data and structure refinement for **5c**

Identification code	**5c**
Empirical formula	$C_{20}H_{19}F_3O_2SSi$
Formula weight	408.50
Temperature/K	150.00(10)
Crystal system	monoclinic
Space group	$P2_1/c$
a/Å	9.9843(6)
b/Å	11.6667(8)
c/Å	16.1487(10)
α/°	90
β/°	91.534(6)
γ/°	90
Volume/Å^3	1880.4(2)
Z	4
ρ_{calc}g/cm^3	1.443
μ/mm^{-1}	0.277
F(000)	848.0
Crystal size/mm^3	0.13 × 0.12 × 0.11
Radiation	MoKα (λ = 0.71073)
2Θ range for data collection/°	4.08 to 58.708
Index ranges	-11 ≤ h ≤ 12, -14 ≤ k ≤ 15, -22 ≤ l ≤ 16
Reflections collected	10487
Independent reflections	4522 [R_{int} = 0.0249, R_{sigma} = 0.0366]
Data/restraints/parameters	4522/18/273
Goodness-of-fit on F^2	1.049
Final R indexes [I>=2σ (I)]	R_1 = 0.0438, wR_2 = 0.1391
Final R indexes [all data]	R_1 = 0.0545, wR_2 = 0.1501
Largest diff. peak/hole / e Å^{-3}	0.41/-0.45

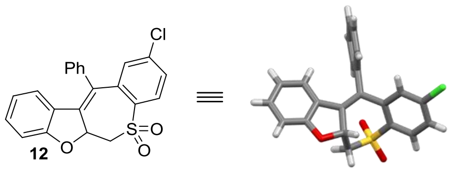

Supplementary Fig. 14 The crystal structure of the compound **12** (the ellipsoid contour probability level is 30%)

Supplementary Table 9 Crystal data and structure refinement for compound **12**

Identification code	**12**
Empirical formula	$C_{22}H_{15}ClO_3S$
Formula weight	394.85
Temperature/K	100.00(10)
Crystal system	triclinic
Space group	P-1
a/Å	9.7734(4)
b/Å	13.1044(8)
c/Å	15.9088(10)
α/°	68.630(5)
β/°	72.317(5)
γ/°	78.591(4)
Volume/Å^3	1799.04(19)
Z	4
ρ_{calc}g/cm^3	1.458
μ/mm^{-1}	0.349
F(000)	816.0
Crystal size/mm^3	0.14 × 0.12 × 0.11
Radiation	Mo Kα (λ = 0.71073)
2Θ range for data collection/°	4.396 to 50
Index ranges	-11 ≤ h ≤ 11, -15 ≤ k ≤ 12, -18 ≤ l ≤ 18
Reflections collected	11993
Independent reflections	6341 [R_{int} = 0.0318, R_{sigma} = 0.0565]
Data/restraints/parameters	6341/0/487
Goodness-of-fit on F^2	1.028
Final R indexes [I>=2σ (I)]	R_1 = 0.0412, wR_2 = 0.0911
Final R indexes [all data]	R_1 = 0.0536, wR_2 = 0.0993
Largest diff. peak/hole / e Å^{-3}	0.29/-0.47

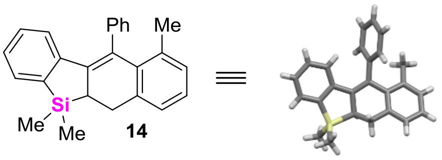

Supplementary Fig. 15 The crystal structure of the compound **14** (the ellipsoid contour probability level is 30%)

Supplementary Table 10 Crystal data and structure refinement for compound **14**

Identification code	**14**
Empirical formula	$C_{25}H_{24}Si$
Formula weight	352.53
Temperature/K	150.00(10)
Crystal system	orthorhombic
Space group	$P2_12_12_1$
a/Å	20.9403(3)
b/Å	15.0638(3)
c/Å	6.30330(10)
α/°	90
β/°	90
γ/°	90
Volume/Å^3	1988.32(6)
Z	4
ρ_{calc}g/cm^3	1.178
μ/mm^{-1}	1.053
F(000)	752.0
Crystal size/mm^3	0.16 × 0.12 × 0.1
Radiation	Cu Kα (λ = 1.54184)
2Θ range for data collection/°	7.23 to 147.124
Index ranges	$-25 \leq h \leq 25$, $-18 \leq k \leq 18$, $-6 \leq l \leq 7$
Reflections collected	16931
Independent reflections	3953 [R_{int} = 0.0441, R_{sigma} = 0.0358]
Data/restraints/parameters	3953/0/239
Goodness-of-fit on F^2	1.040
Final R indexes [I>=2σ (I)]	R_1 = 0.0333, wR_2 = 0.0813
Final R indexes [all data]	R_1 = 0.0395, wR_2 = 0.0835
Largest diff. peak/hole / e Å^{-3}	0.25/-0.28
Flack parameter	0.47(3)

X. Supplementary References

1. Jiang, T.; Gu, Z. Y.; Yin, L.; Wang, S. Y.; Ji, S. J. *J. Org. Chem*. **2017**, *82*, 7913−7919.
2. Takamoto, K.; Yoshioka, S.; Fujioka, H,; Arisawa, M. *Org. Lett.* **2018**, *20*, 1773−1776; Yoshioka, Shohei.; Fujii, Y.; Tsujino, H.; Uno, T.; Fujioka, H.; Arisawa, M. *Chem. Commun.*, **2017**,*53*, 5970−5973.
3. Mesgar, M.; Daugulis, O. *Org. Lett.* **2017**, *19*, 4247−4250.
4. Liang, H.; Ji, Y. X.; Wang, R. H.; Zhang, Z. H.; Zhang, B. *Org. Lett.* **2019**, *21*, 2750−2754.
5. Huang, L.; Rudolph, M.; Rominger, F.; Hashmi, S. K. *Angew. Chem., Int. Ed.* **2016**, *55*,4808−4813.
6. Sakiyama, N.; Noguchi, K.; Tanaka,K. *Angew. Chem., Int. Ed*. **2012**, *51*, 5976−5980.
7. Chaminda Lakmal, H. H.; Xu, X. Z.; Xue, X.; Ahmed, B.; Fong, C.; S, D. J.; Ramig, K.; Sygula, A.; Webster, C. E.; Zhang, D. M.; Cui, X. *J. Org. Chem.* **2018**, *83*, 9497−9503.
8. Huang, Y. G.; Xu, F. G.; Ganzer, L.; Camargo, F. V. A.; Nagahara, T.; Teyssandier, J.; Gorp, H. V.; Basse, K.; Straasø, L. A.; Nagyte, V.; Casiraghi, C.; Hansen, M. R.; Feyter, S. D.; Yan, D.; Müllen, K.; Feng, X. L.; Feng, X. L.; Cerullo, G.; Mai, Y. Y. *J. Am. Chem. Soc.* **2018**, *140*, 10416−10420.
9. Yu, X. F.; Lu, X. Y. *Adv. Synth. Catal.* **2011**, *353*, 569−574.
10. Pavlischuk, V. V.; Addison, A. W. *Inorg. Chim. Acta.* **2000**, *298*, 97-102.
11. Zhao, Y.; Truhlar, D. G. *J. Chem. Phys.* **2006**, *125*, 194101.
12. Gaussian 09, Revision D.01, M. J. Frisch, G. W. Trucks, H. B. Schlegel, G. E. Scuseria, M. A. Robb, J. R. Cheeseman, G. Scalmani, V. Barone, G. A. Petersson, H. Nakatsuji, X. Li, M. Caricato, A. Marenich, J. Bloino, B. G. Janesko, R. Gomperts, B. Mennucci, H. P. Hratchian, J. V. Ortiz, A. F. Izmaylov, J. L. Sonnenberg, D. Williams-Young, F. Ding, F. Lipparini, F. Egidi, J. Goings, B. Peng, A. Petrone, T. Henderson, D. Ranasinghe, V. G. Zakrzewski, J. Gao, N. Rega, G. Zheng, W. Liang, M. Hada, M. Ehara, K. Toyota, R. Fukuda, J. Hasegawa, M. Ishida, T. Nakajima, Y. Honda, O. Kitao, H. Nakai, T. Vreven, K. Throssell, J. A. Montgomery, Jr., J. E. Peralta, F. Ogliaro, M. Bearpark, J. J. Heyd, E. Brothers, K. N. Kudin, V. N. Staroverov, T. Keith, R. Kobayashi, J. Normand, K. Raghavachari, A. Rendell, J. C. Burant, S. S. Iyengar, J. Tomasi, M. Cossi, J. M. Millam, M. Klene, C. Adamo, R. Cammi, J. W. Ochterski, R. L. Martin, K. Morokuma, O. Farkas, J. B. Foresman, and D. J. Fox, Gaussian, Inc., Wallingford CT, 2013.

Appendix-I: Spectral Copies of ^{1}H and ^{13}C NMR of Compounds

^{1}H NMR spectrum (400 MHz, $CDCl_3$) of **1a**

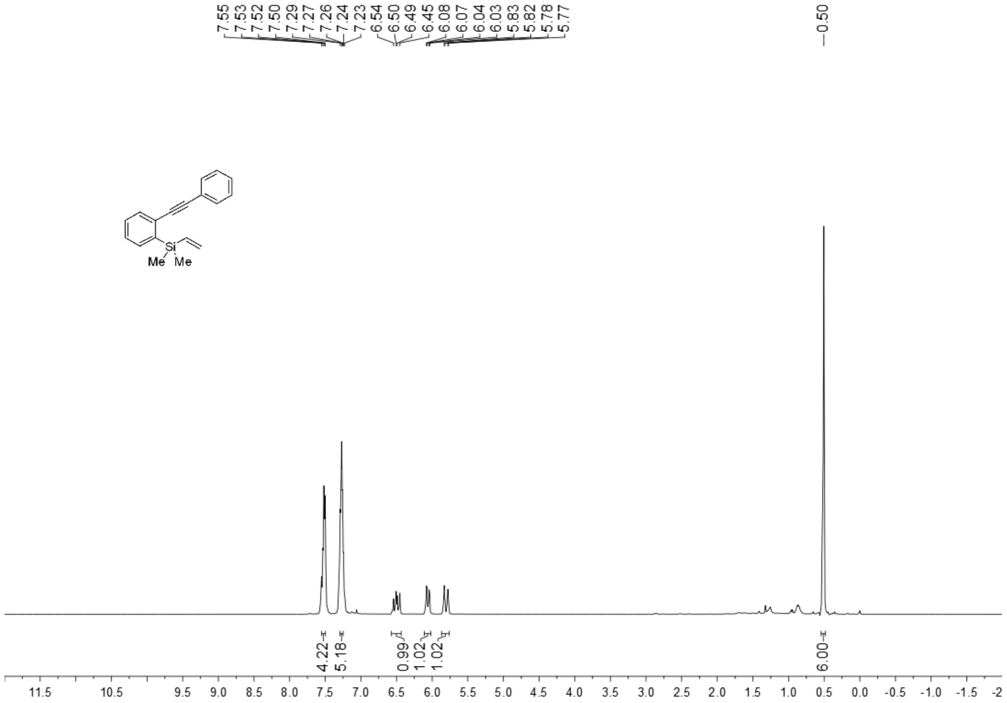

^{13}C NMR spectrum (101 MHz, $CDCl_3$) of **1a**

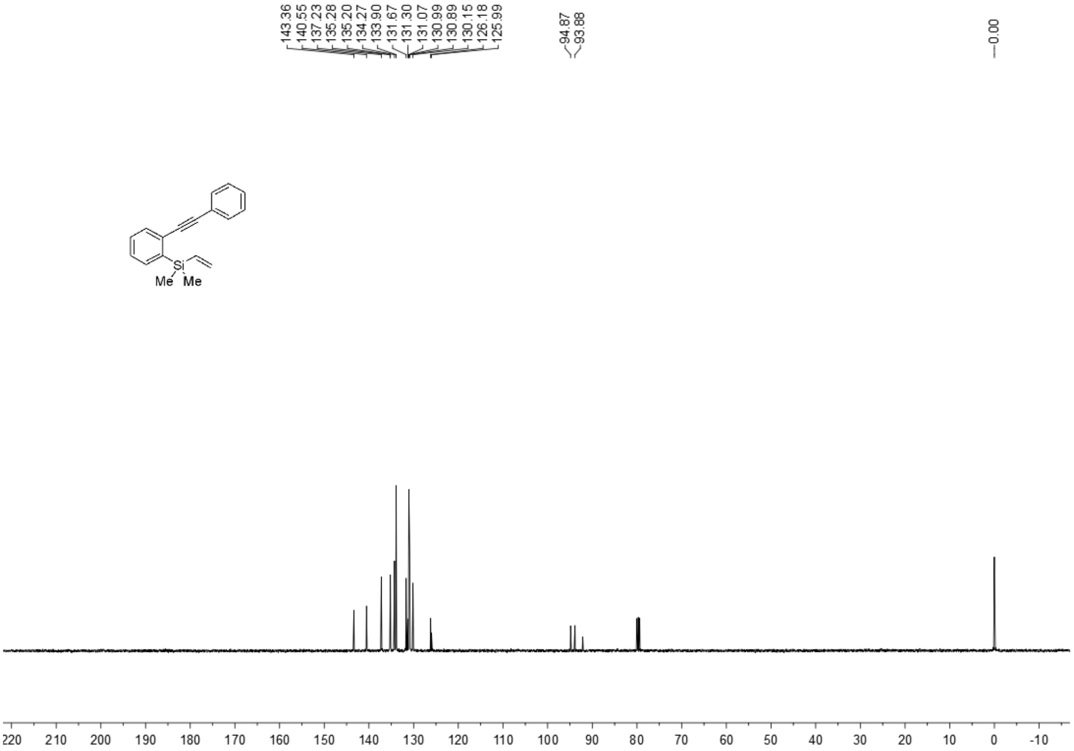

^{1}H NMR spectrum (400 MHz, $CDCl_3$) of **1b**

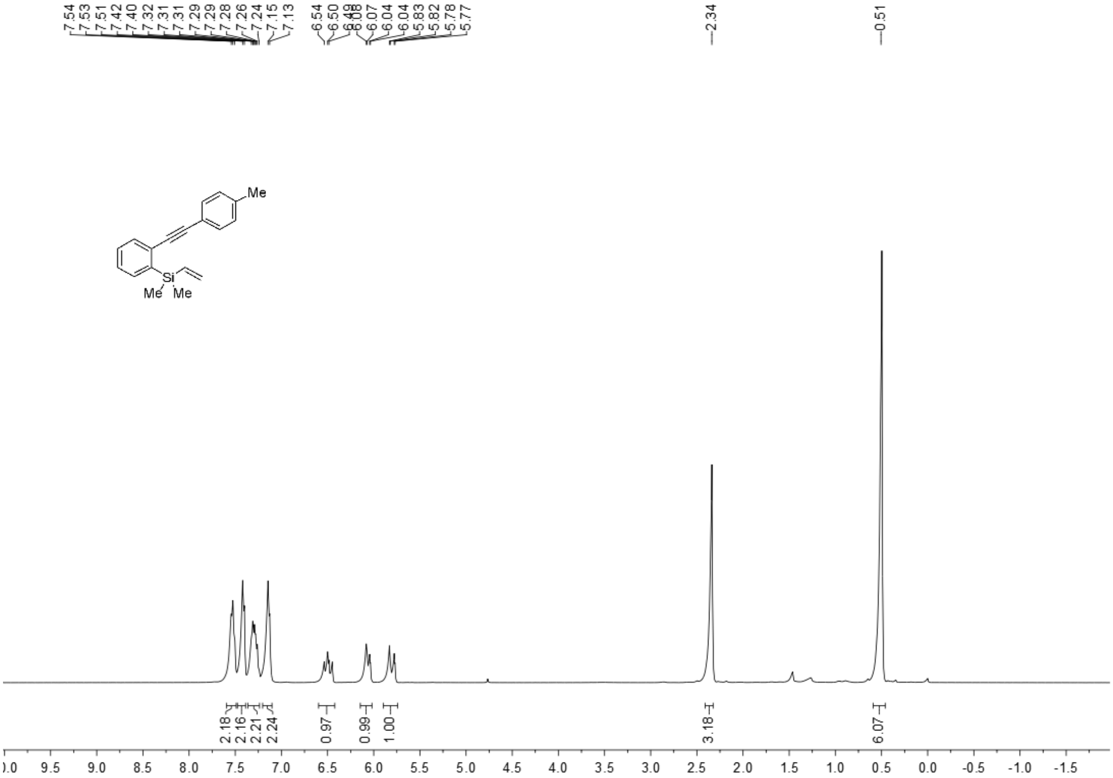

^{13}C NMR spectrum (101 MHz, $CDCl_3$) of **1b**

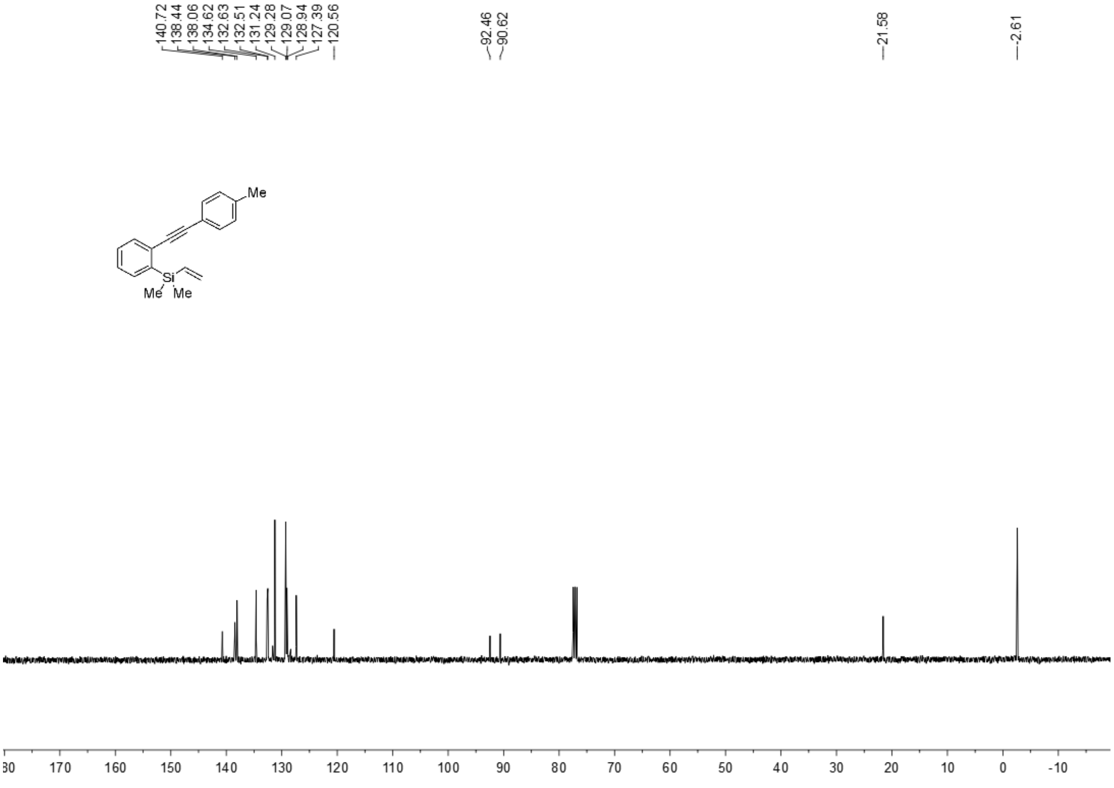

^{1}H NMR spectrum (500 MHz, $CDCl_3$) of **1c**

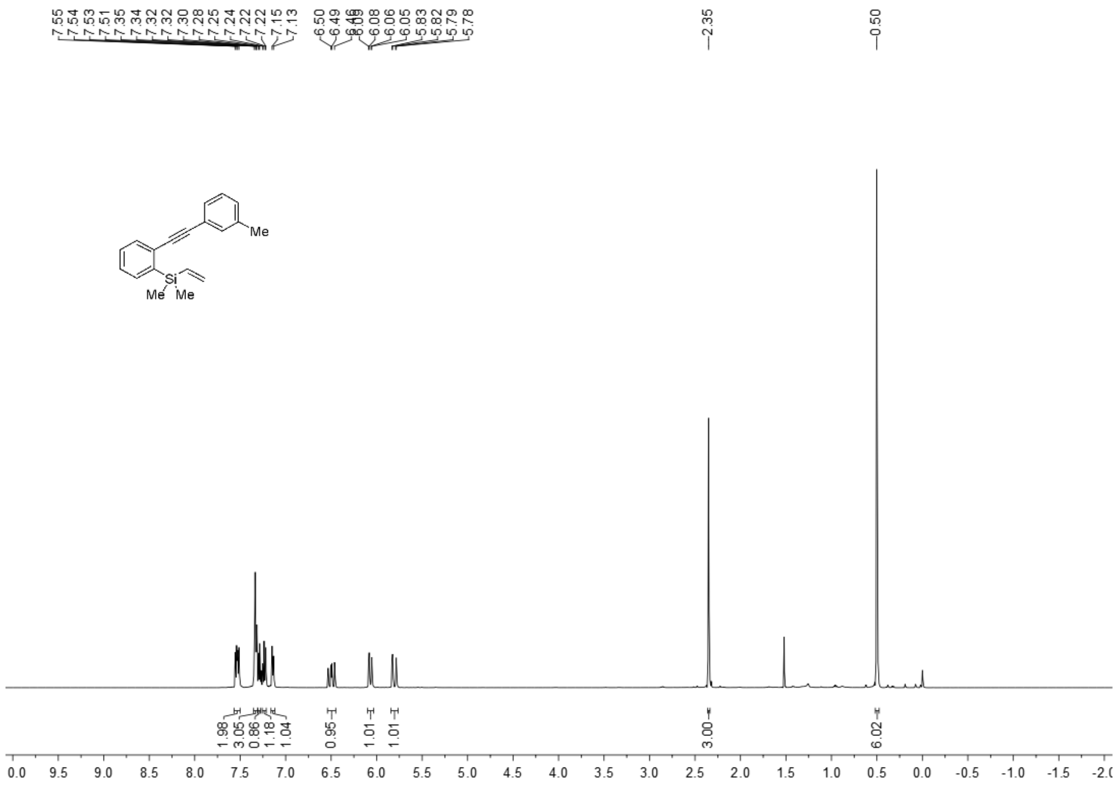

^{13}C NMR spectrum (126 MHz, $CDCl_3$) of **1c**

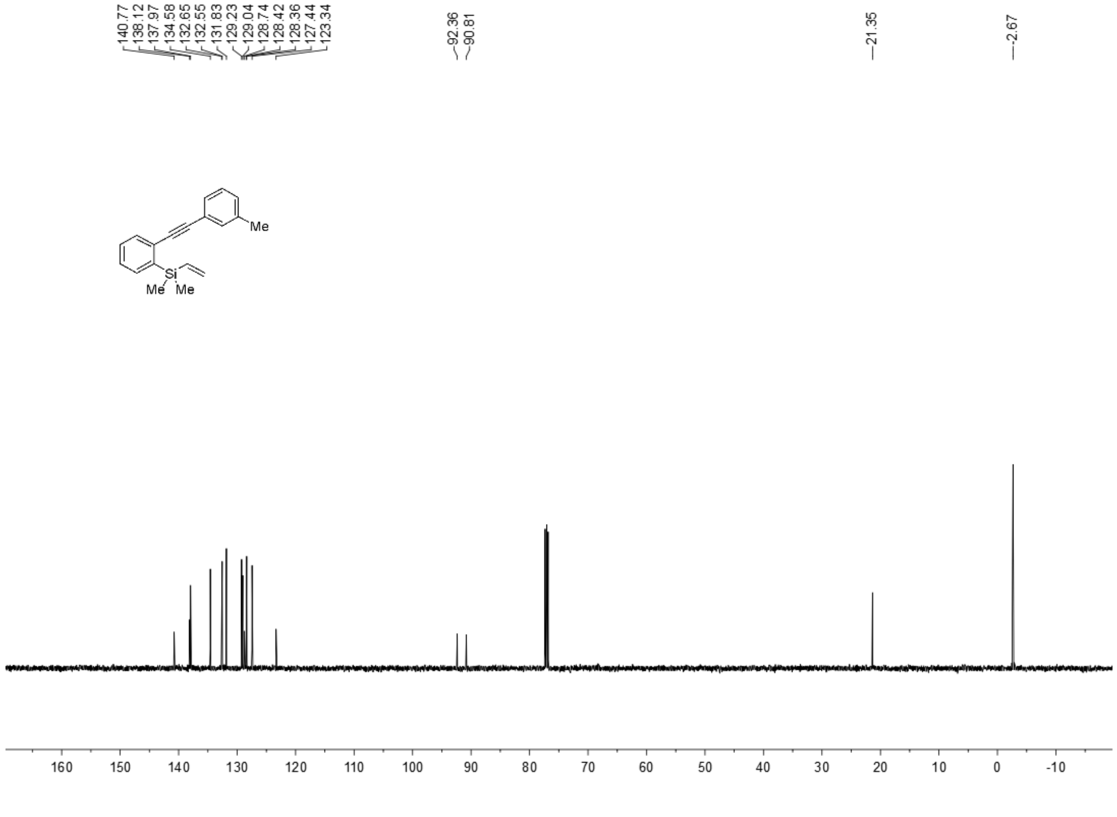

^{1}H NMR spectrum (500 MHz, $CDCl_3$) of **1d**

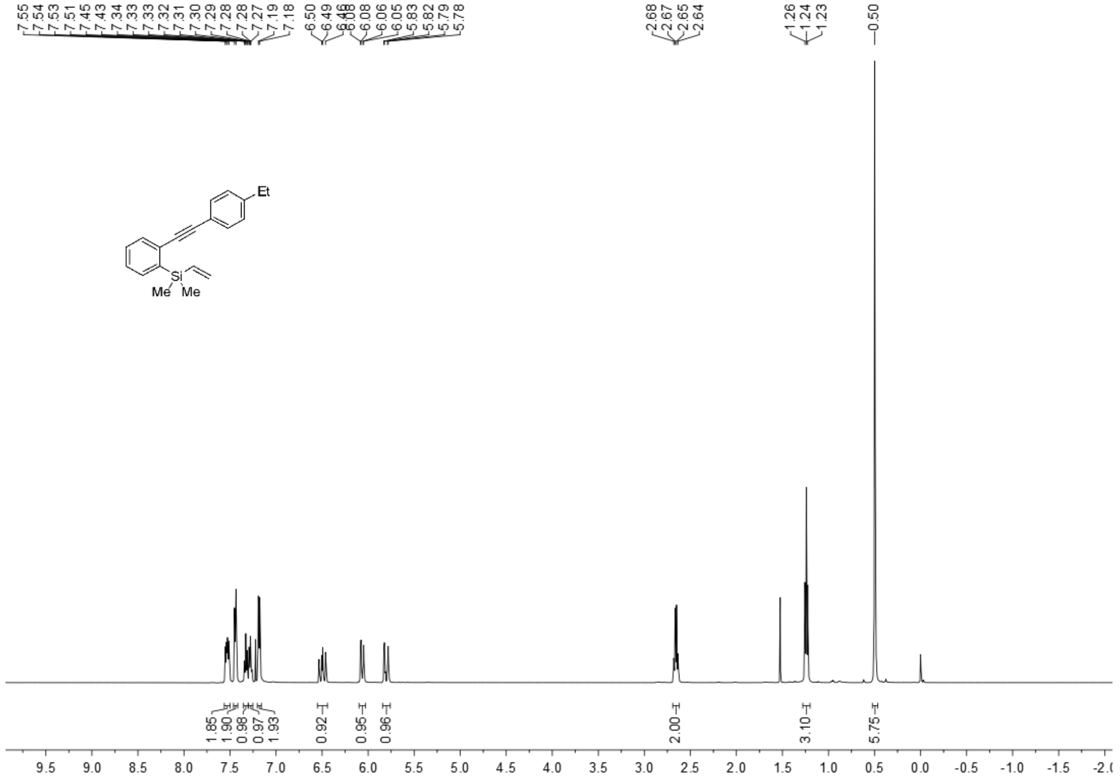

^{13}C NMR spectrum (126 MHz, $CDCl_3$) of **1d**

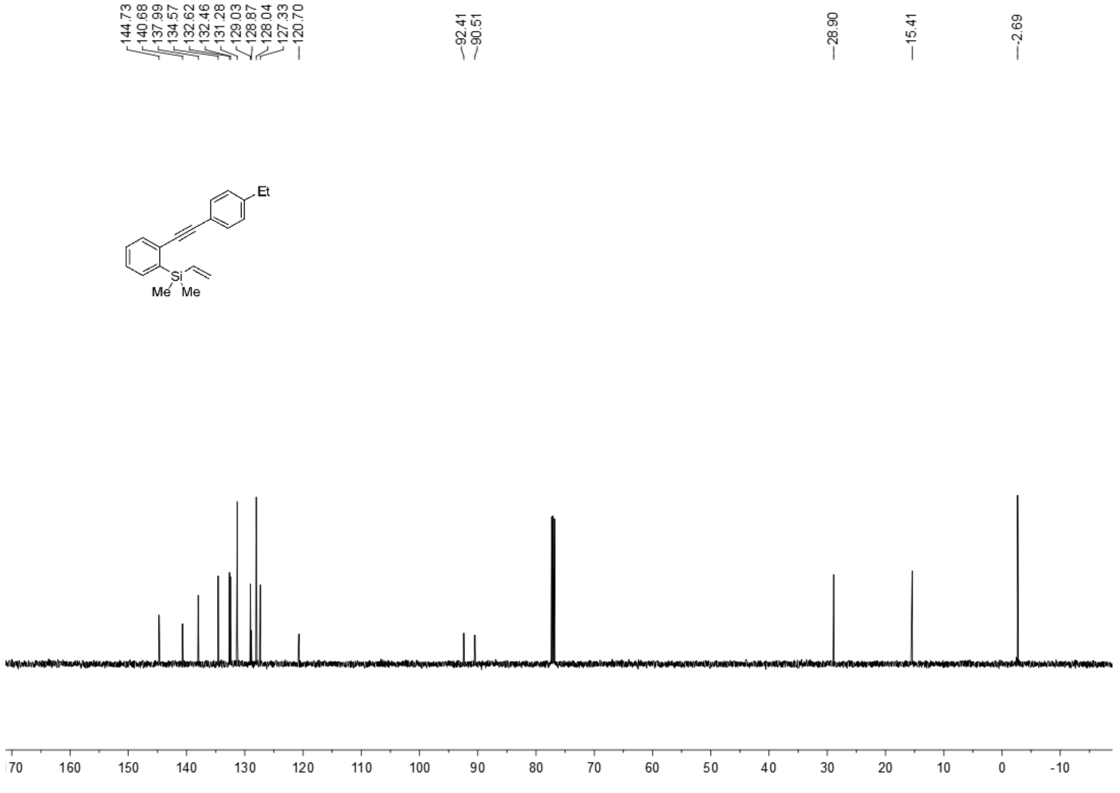

^{1}H NMR spectrum (500 MHz, $CDCl_3$) of **1e**

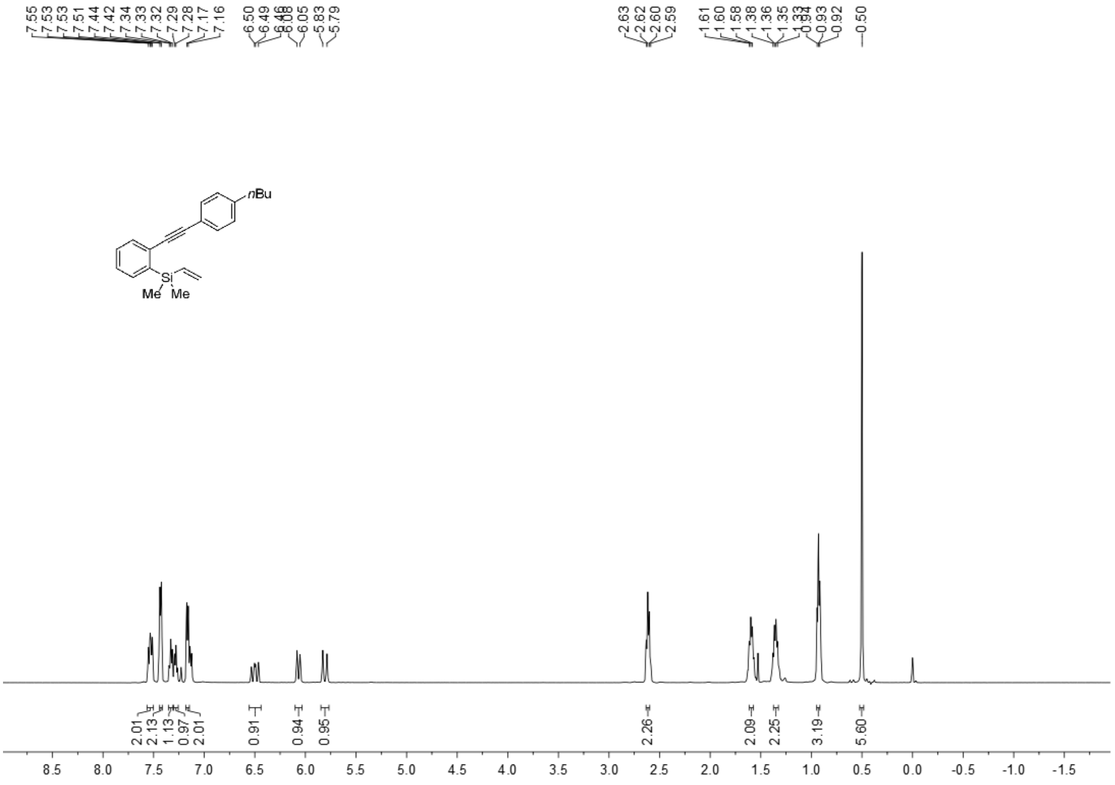

^{13}C NMR spectrum (101 MHz, $CDCl_3$) of **1e**

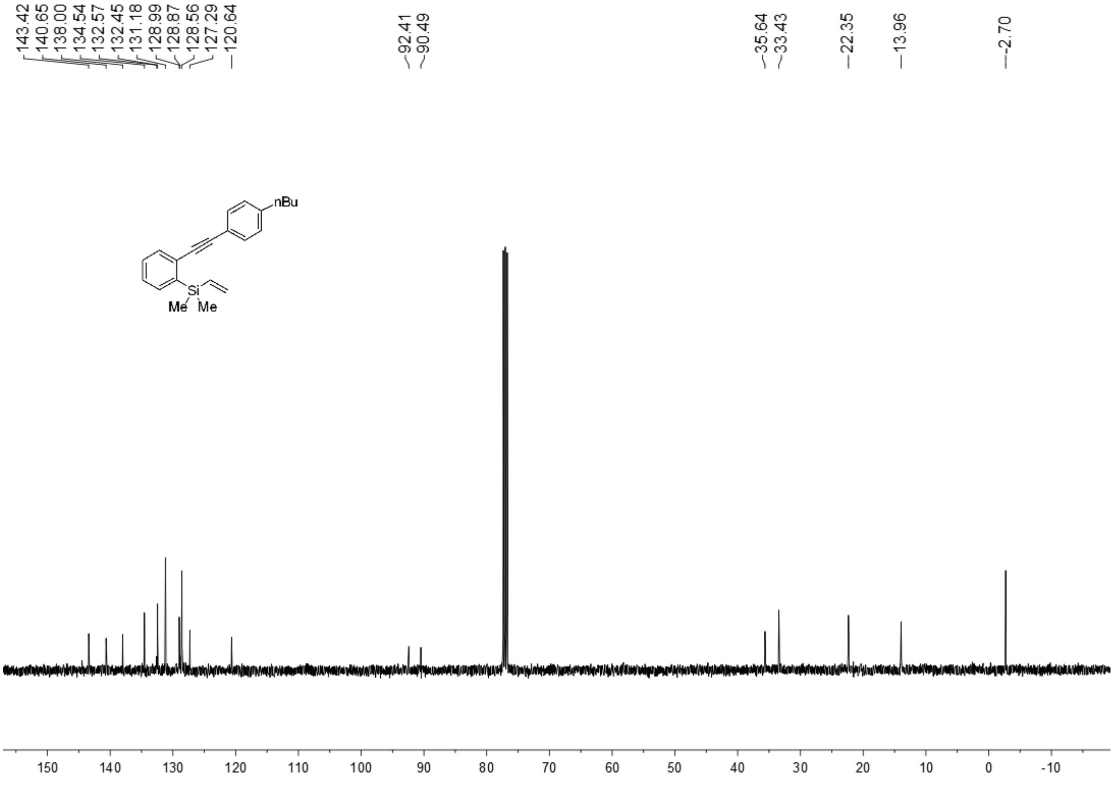

^{1}H NMR spectrum (500 MHz, $CDCl_3$) of **1f**

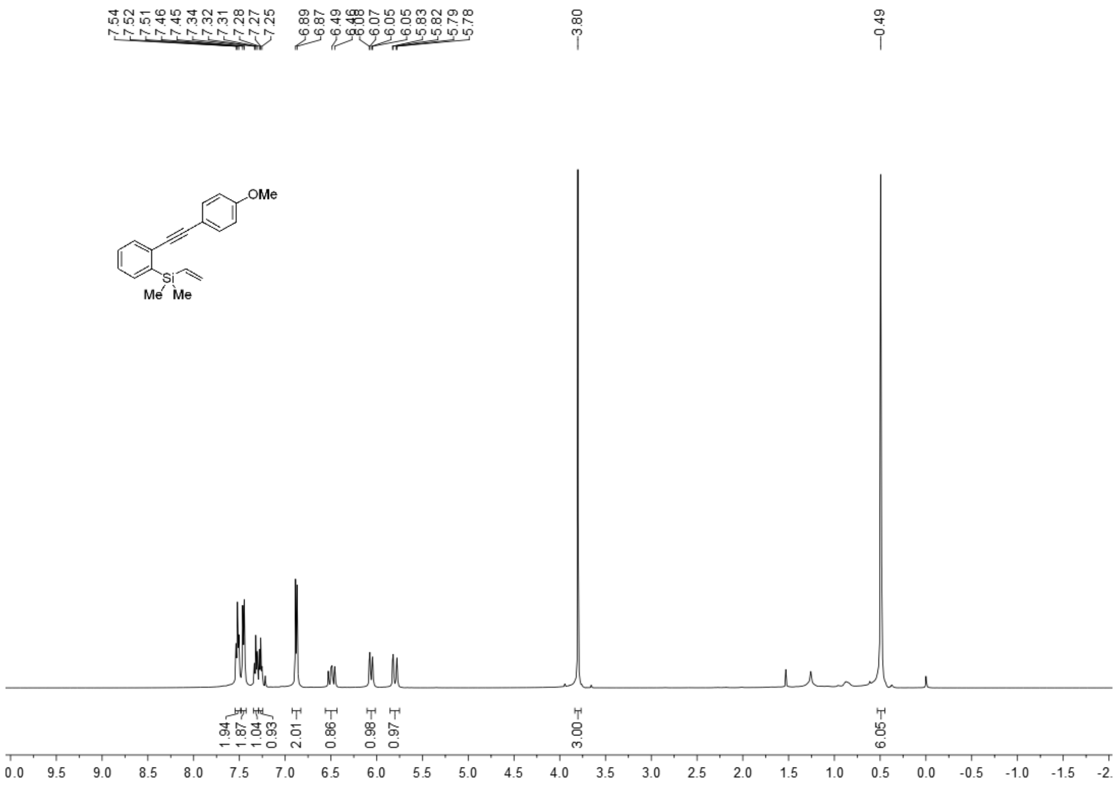

^{13}C NMR spectrum (126 MHz, $CDCl_3$) of **1f**

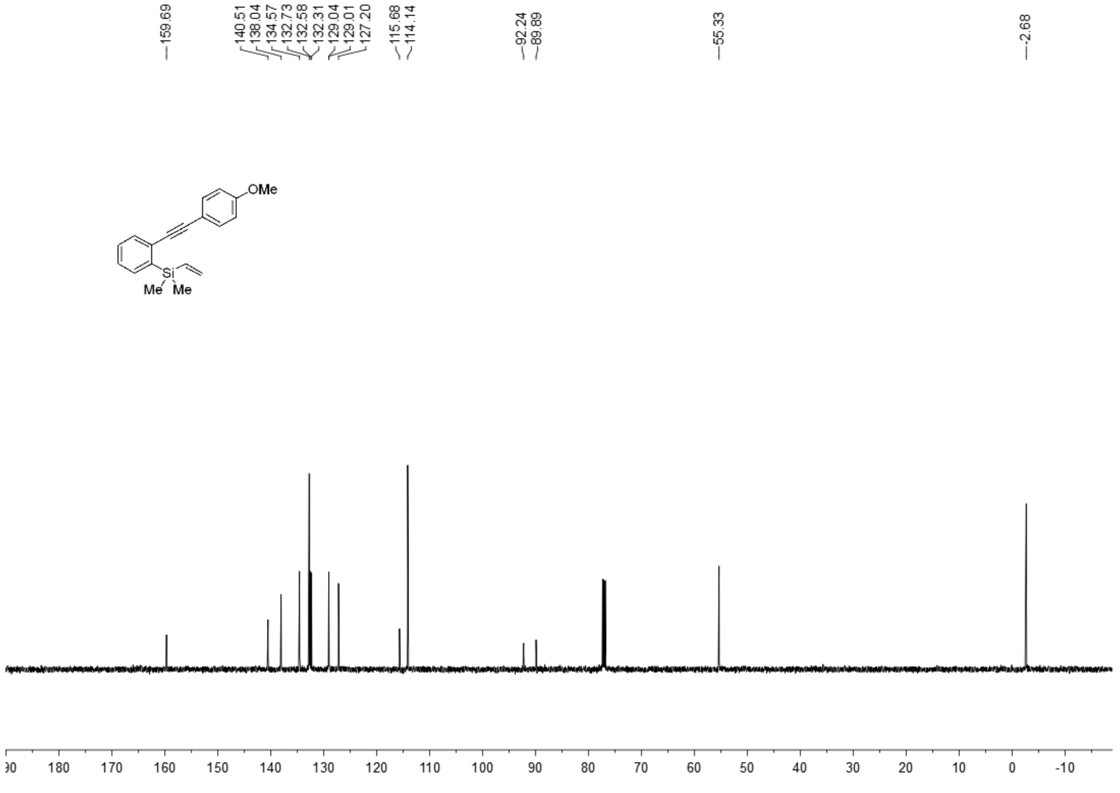

^{1}H NMR spectrum (500 MHz, $CDCl_3$) of **1g**

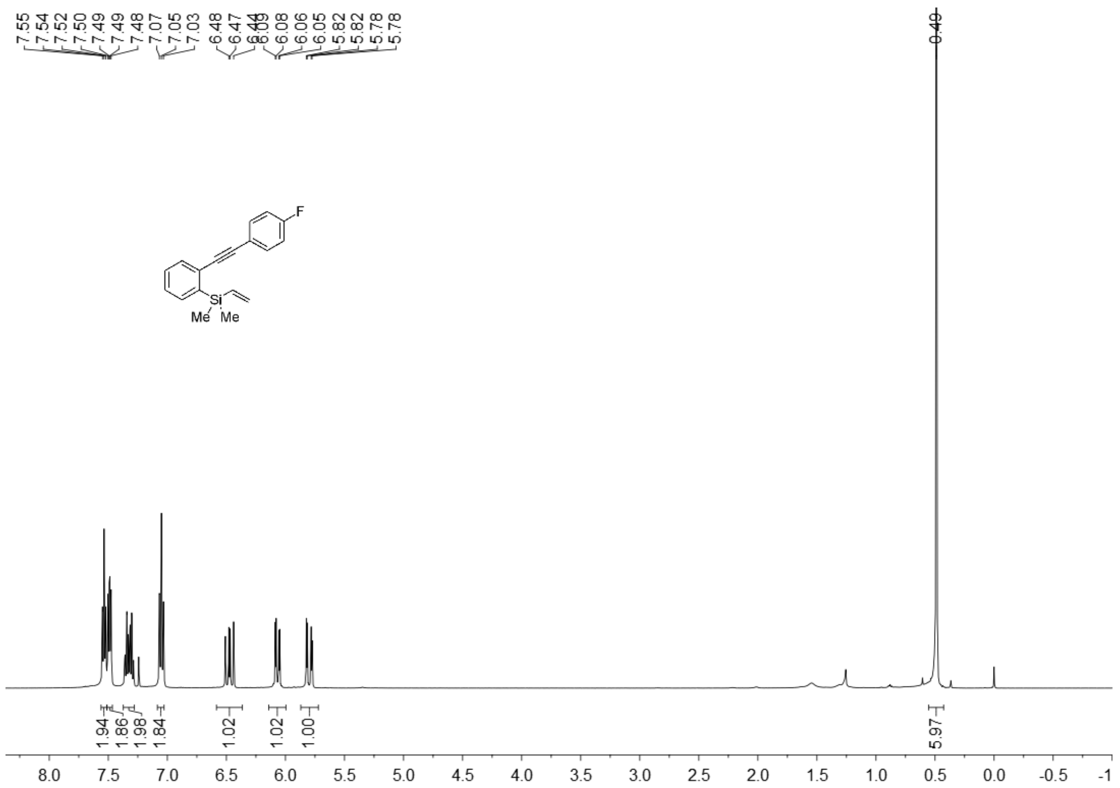

^{13}C NMR spectrum (101 MHz, $CDCl_3$) of **1g**

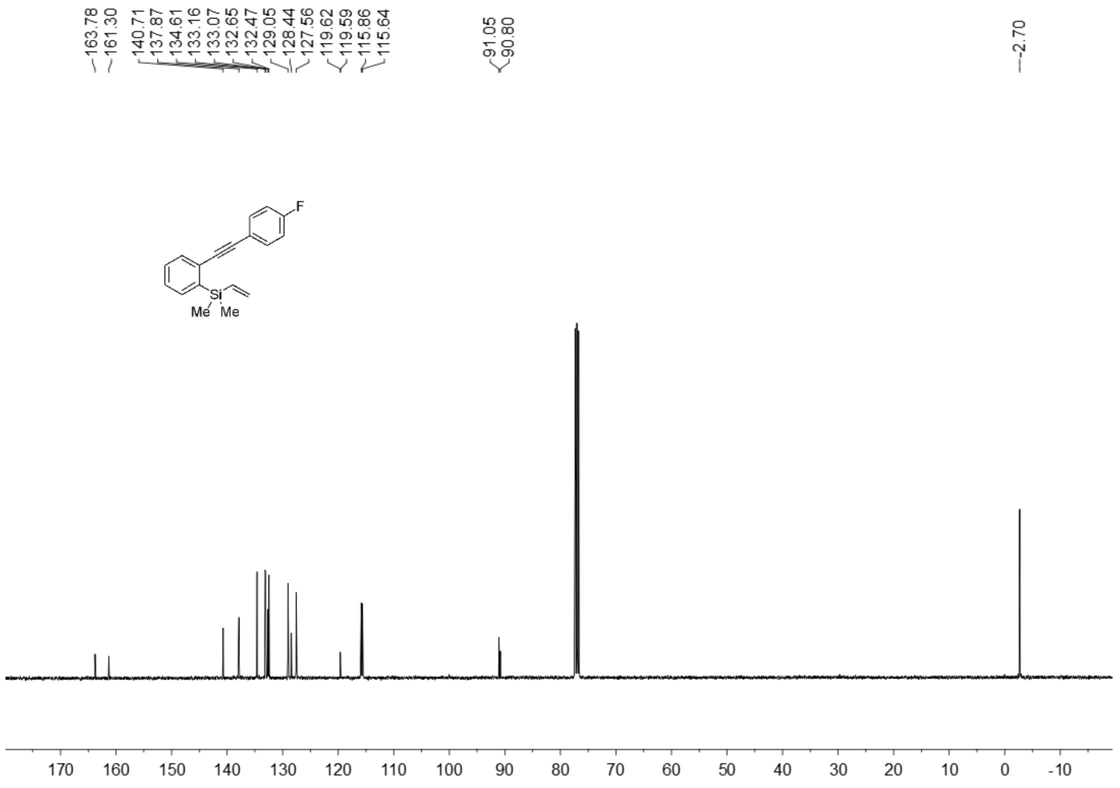

^{1}H NMR spectrum (400 MHz, $CDCl_3$) of **1h**

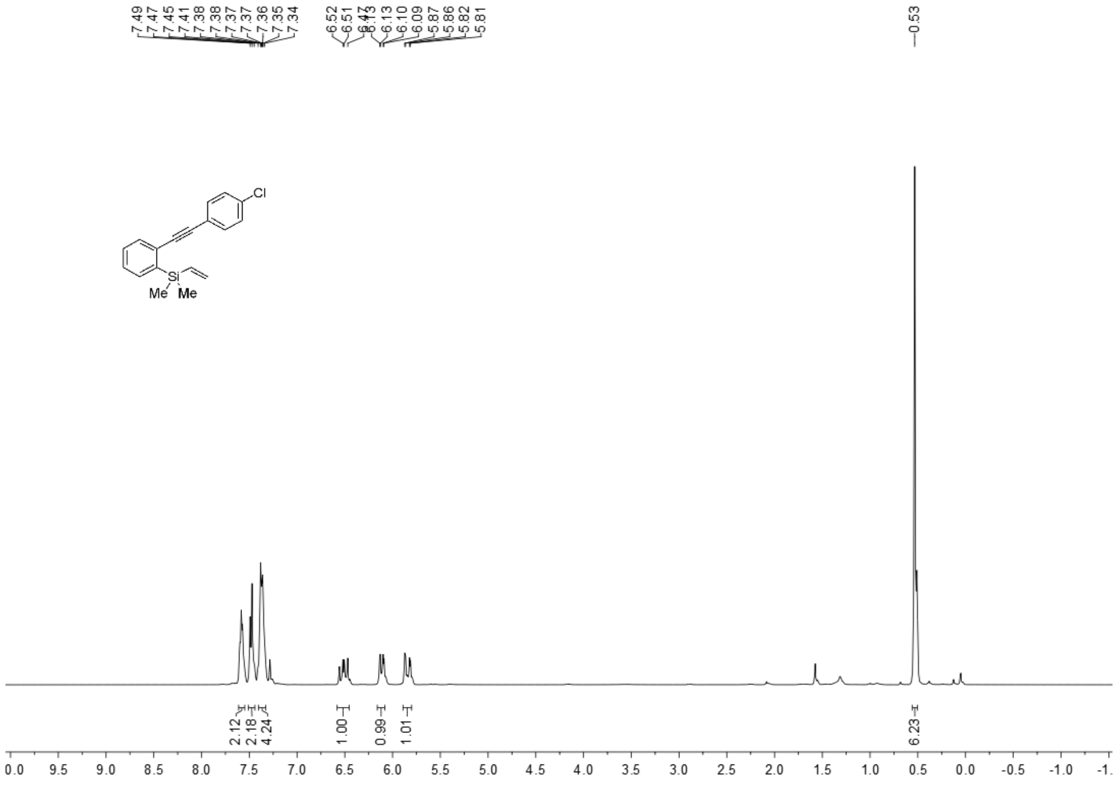

^{13}C NMR spectrum (101 MHz, $CDCl_3$) of **1h**

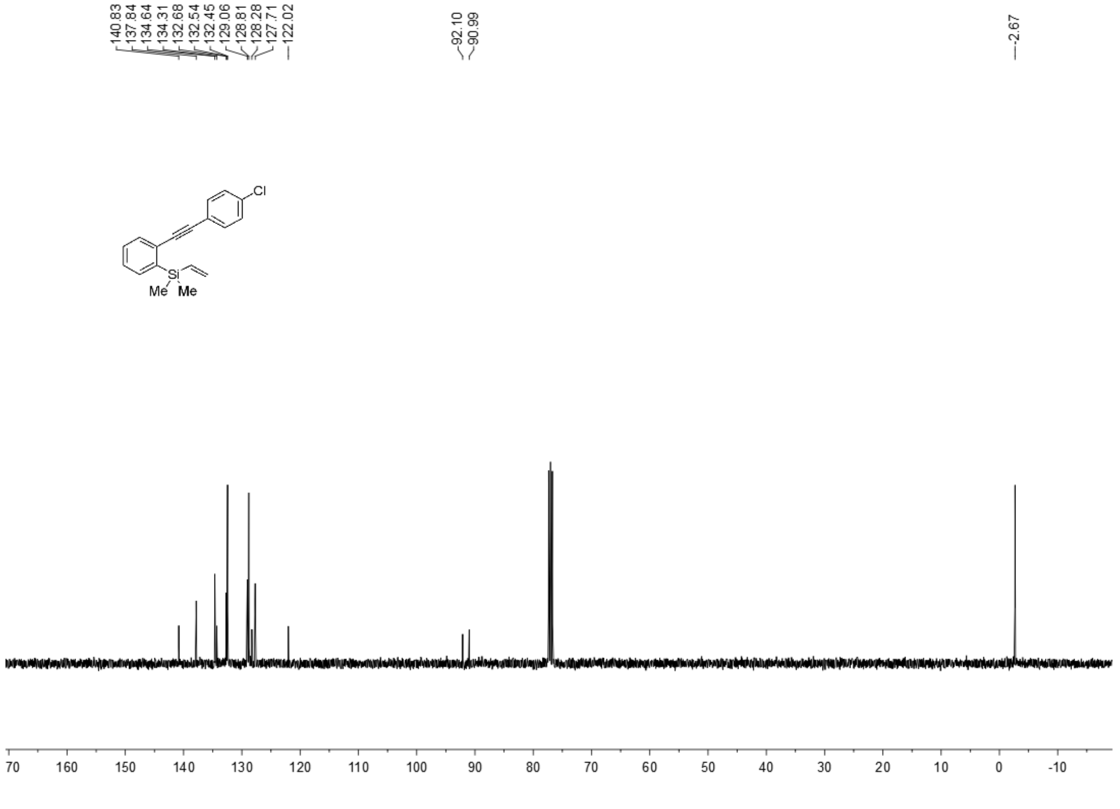

^{1}H NMR spectrum (500 MHz, $CDCl_3$) of **1i**

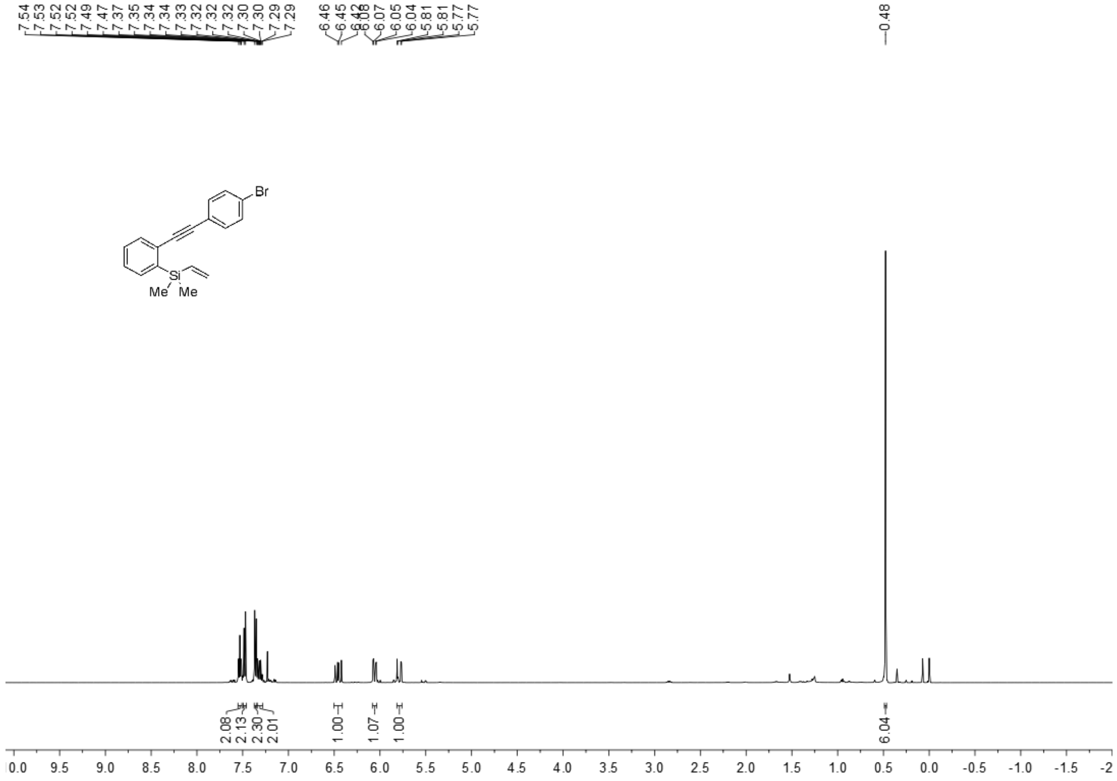

^{13}C NMR spectrum (126 MHz, $CDCl_3$) of **1i**

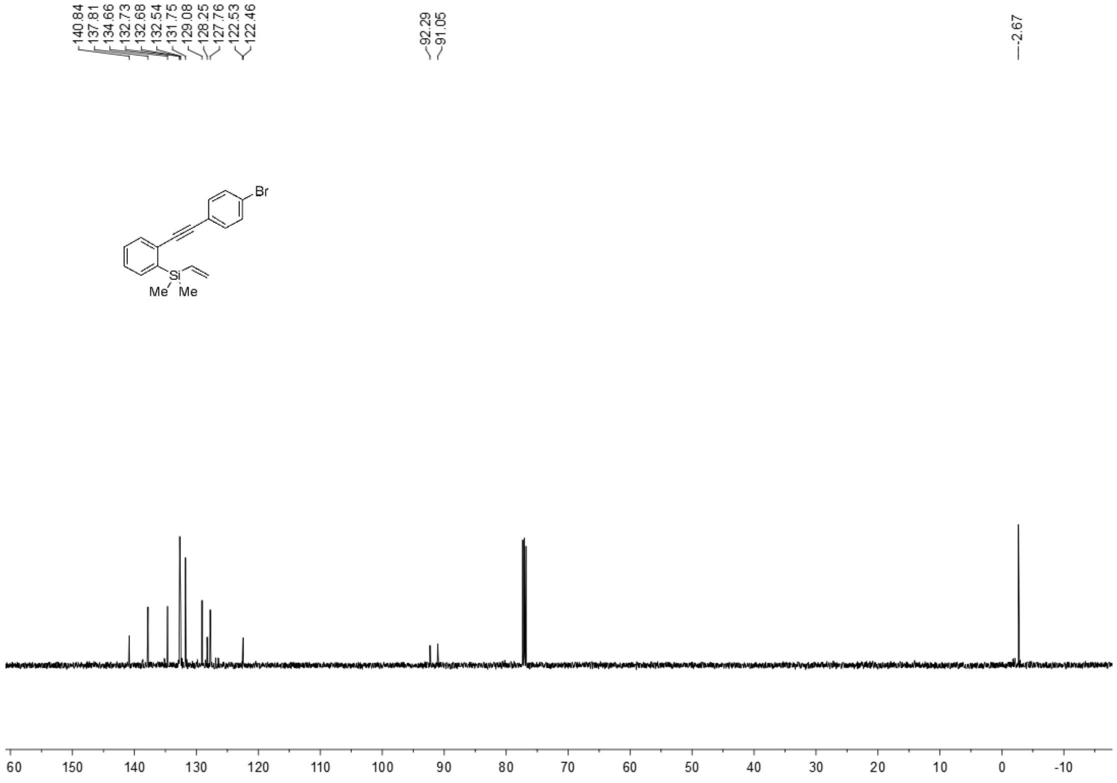

^{1}H NMR spectrum (500 MHz, $CDCl_3$) of **1j**

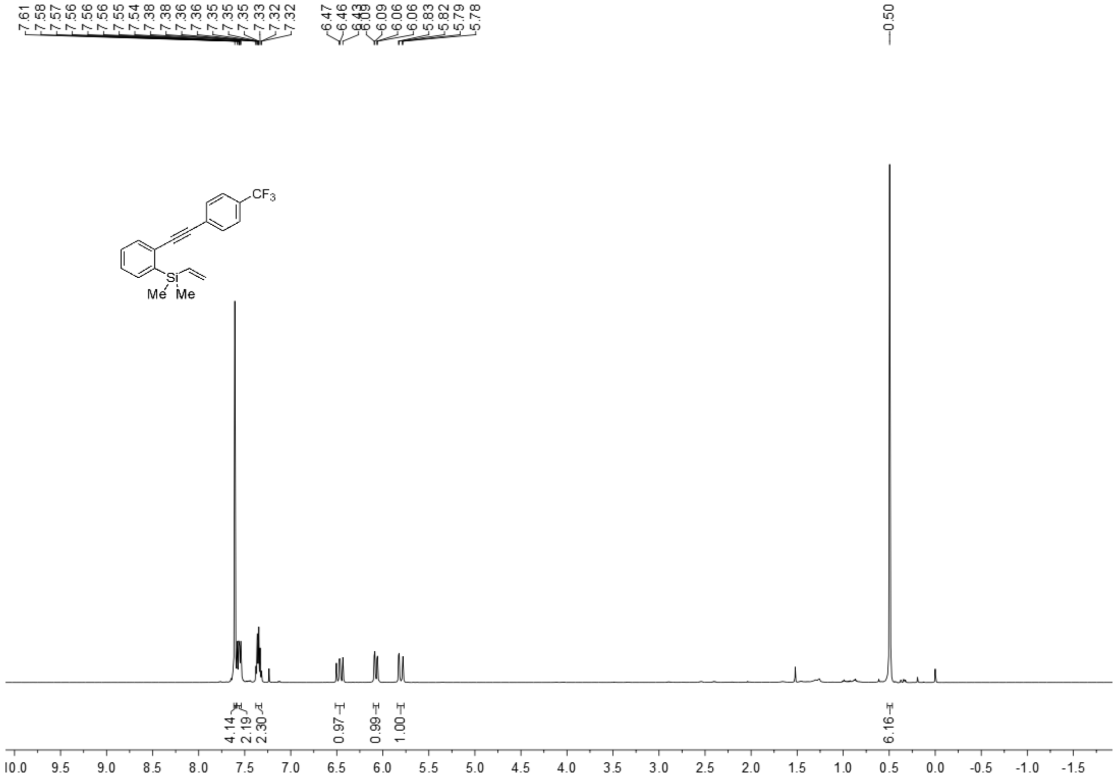

^{13}C NMR spectrum (126 MHz, $CDCl_3$) of **1j**

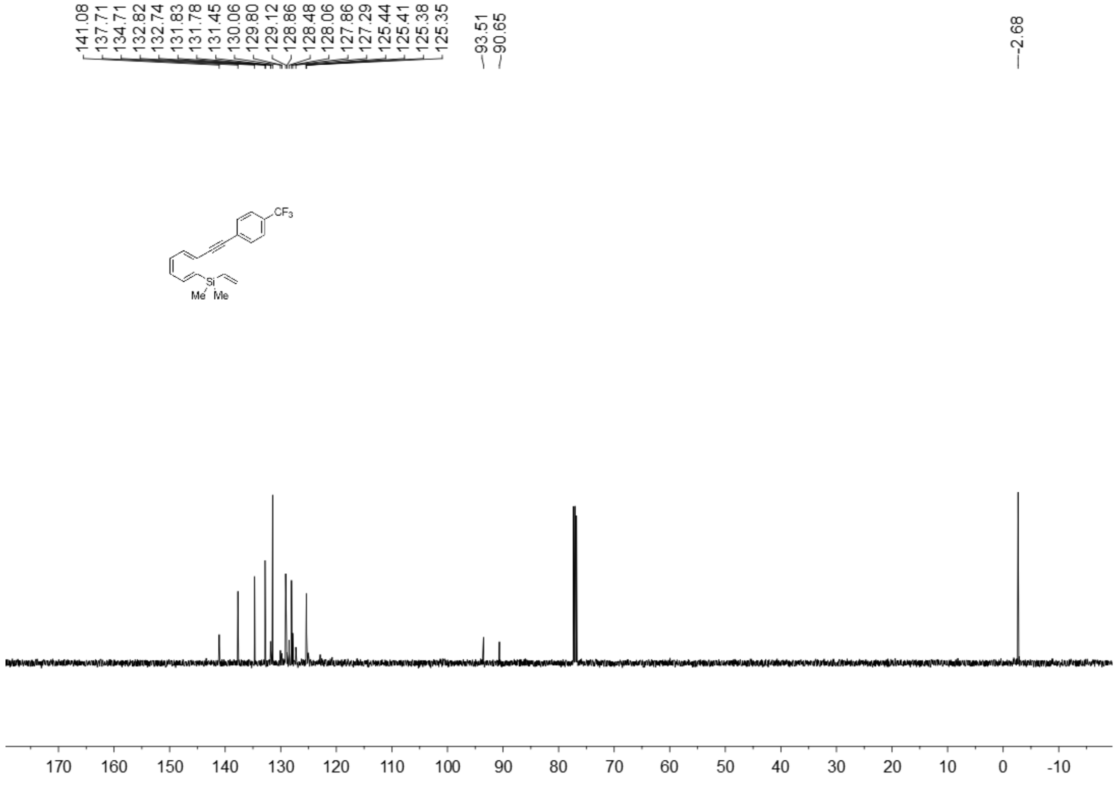

^{1}H NMR spectrum (500 MHz, $CDCl_3$) of **1k**

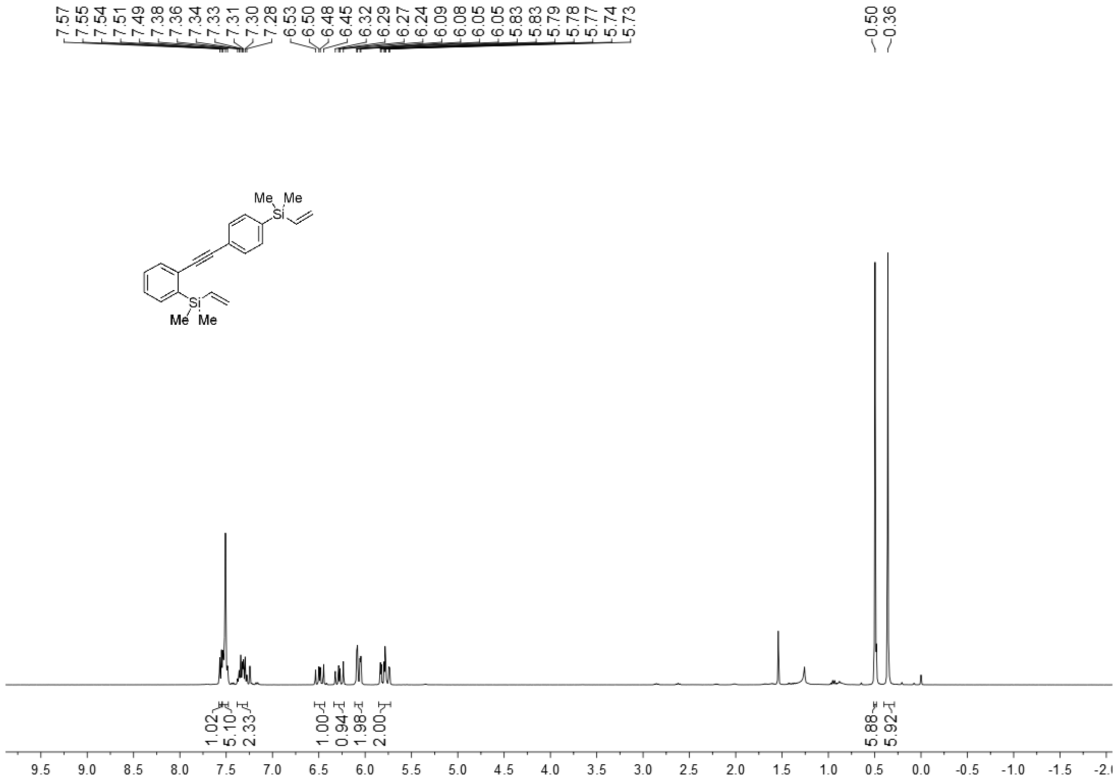

^{13}C NMR spectrum (101 MHz, $CDCl_3$) of **1k**

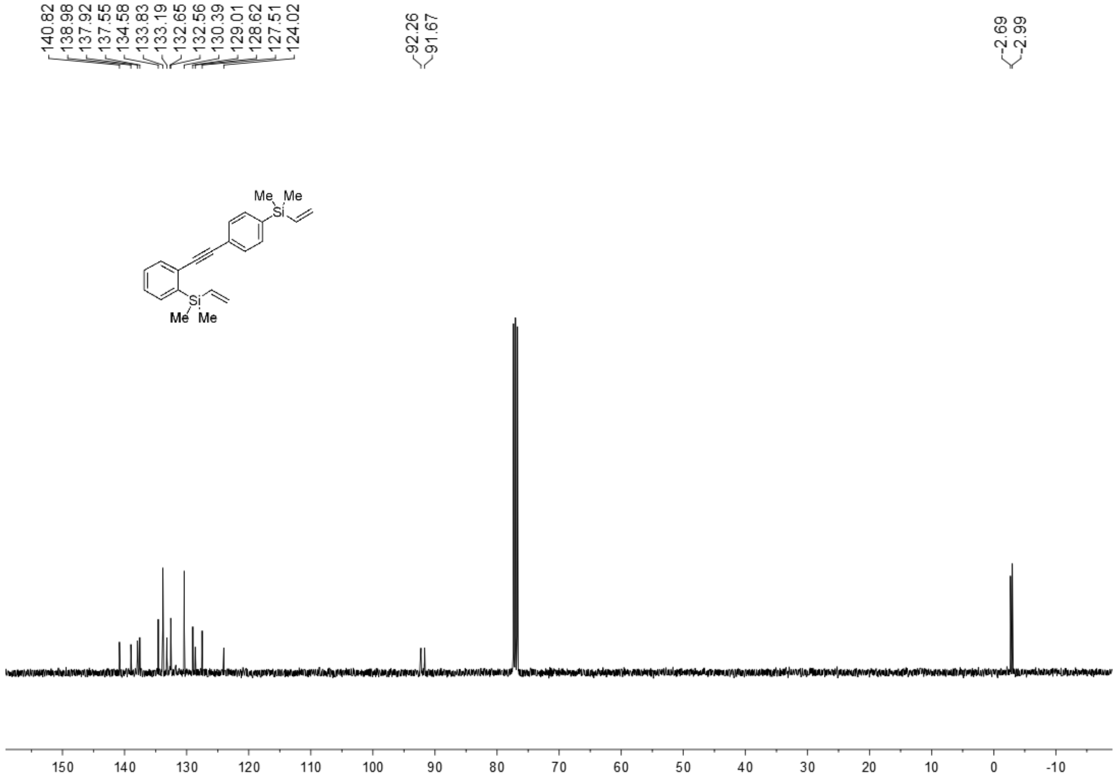

^{1}H NMR spectrum (400 MHz, $CDCl_3$) of **1l**

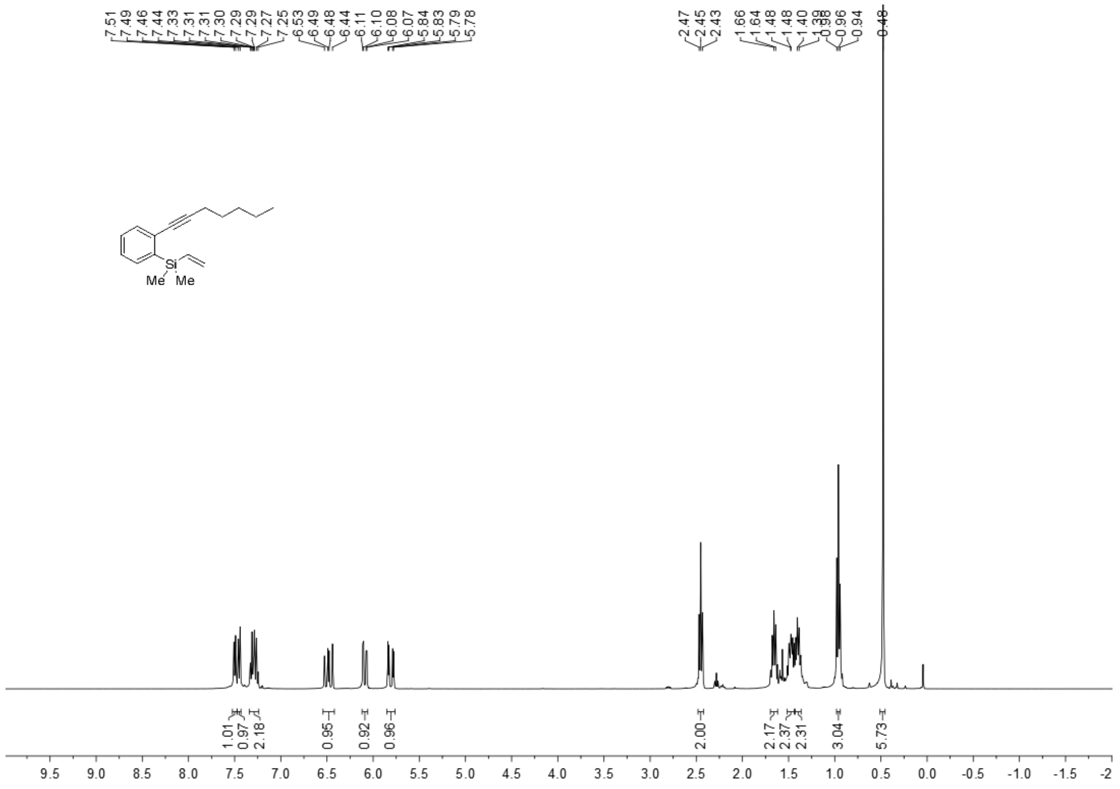

^{13}C NMR spectrum (101 MHz, $CDCl_3$) of **1l**

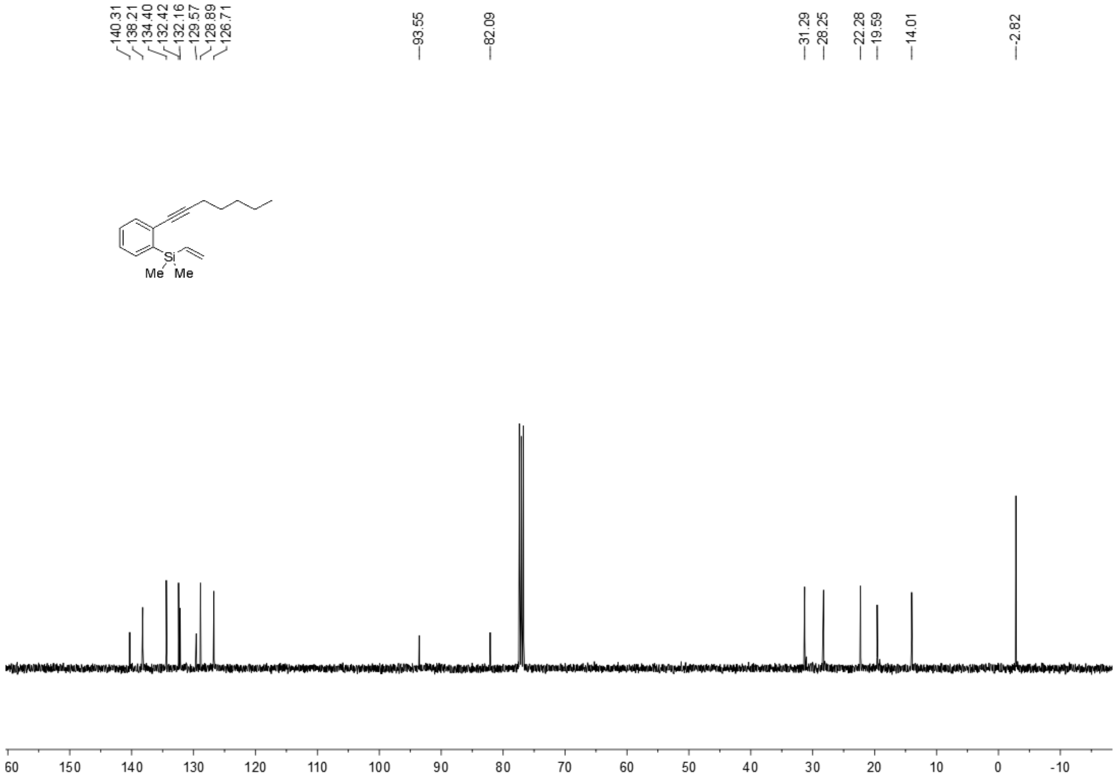

^{1}H NMR spectrum (400 MHz, $CDCl_3$) of **1m**

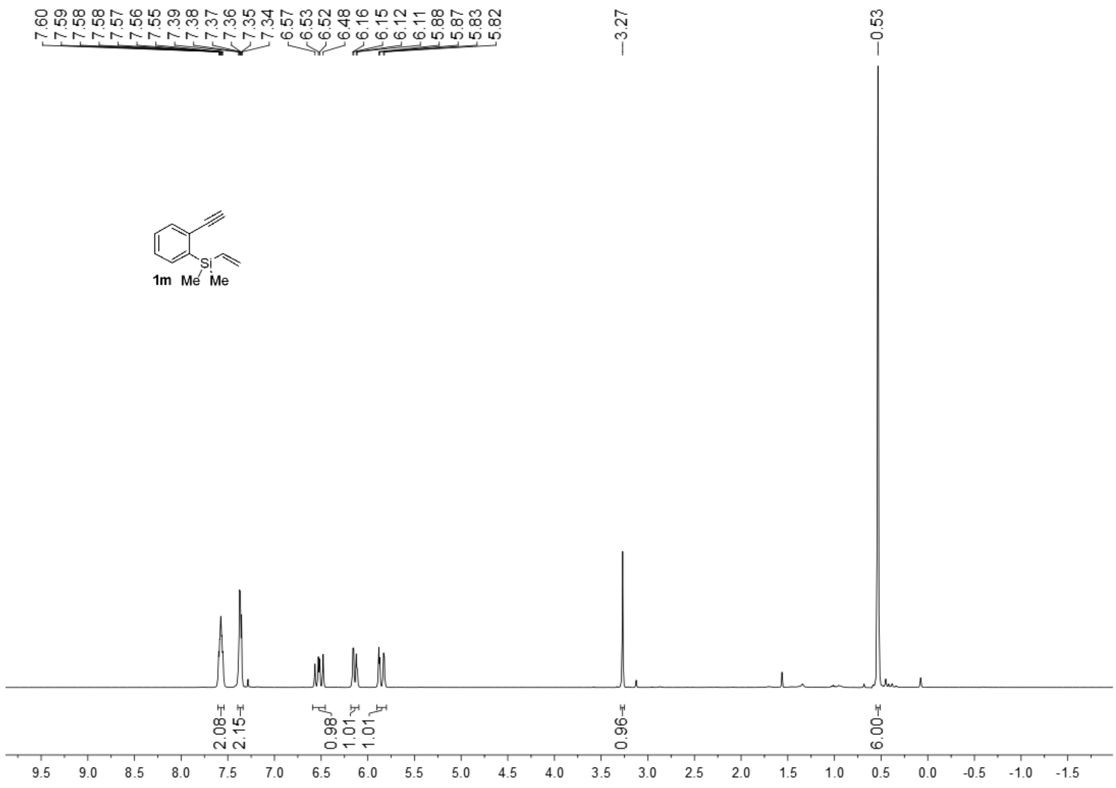

^{13}C NMR spectrum (101 MHz, $CDCl_3$) of **1m**

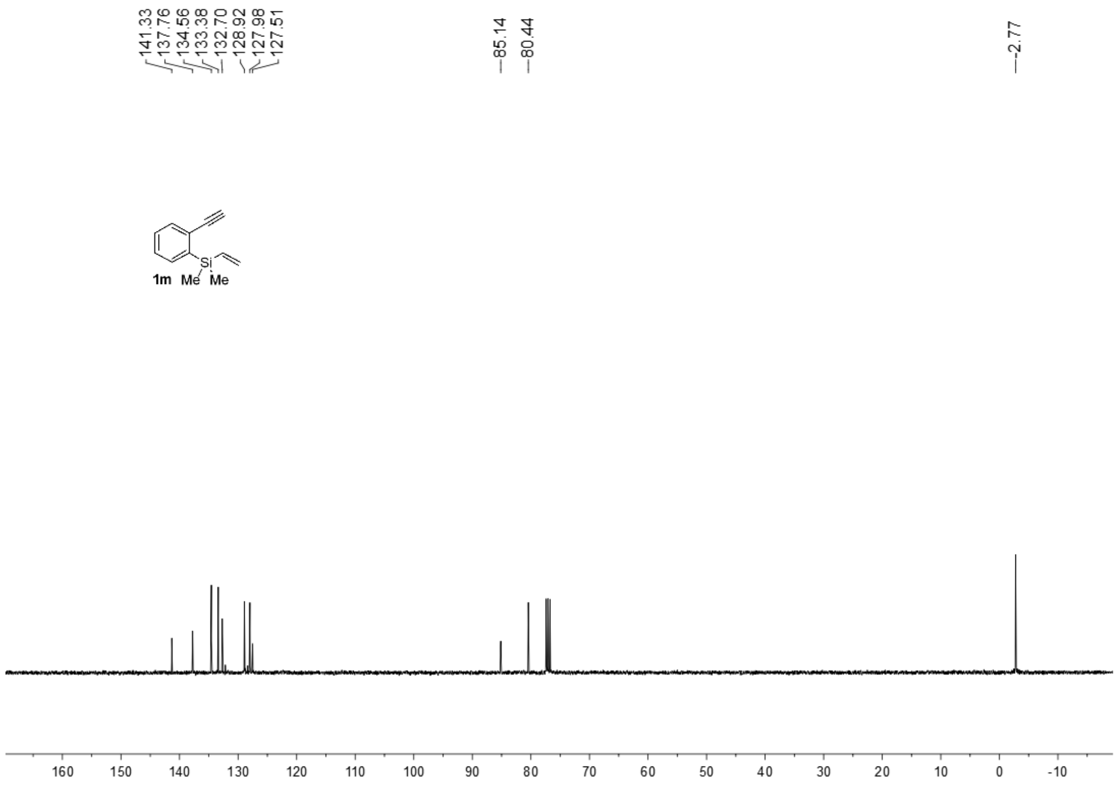

^{1}H NMR spectrum (400 MHz, $CDCl_3$) of **1n**

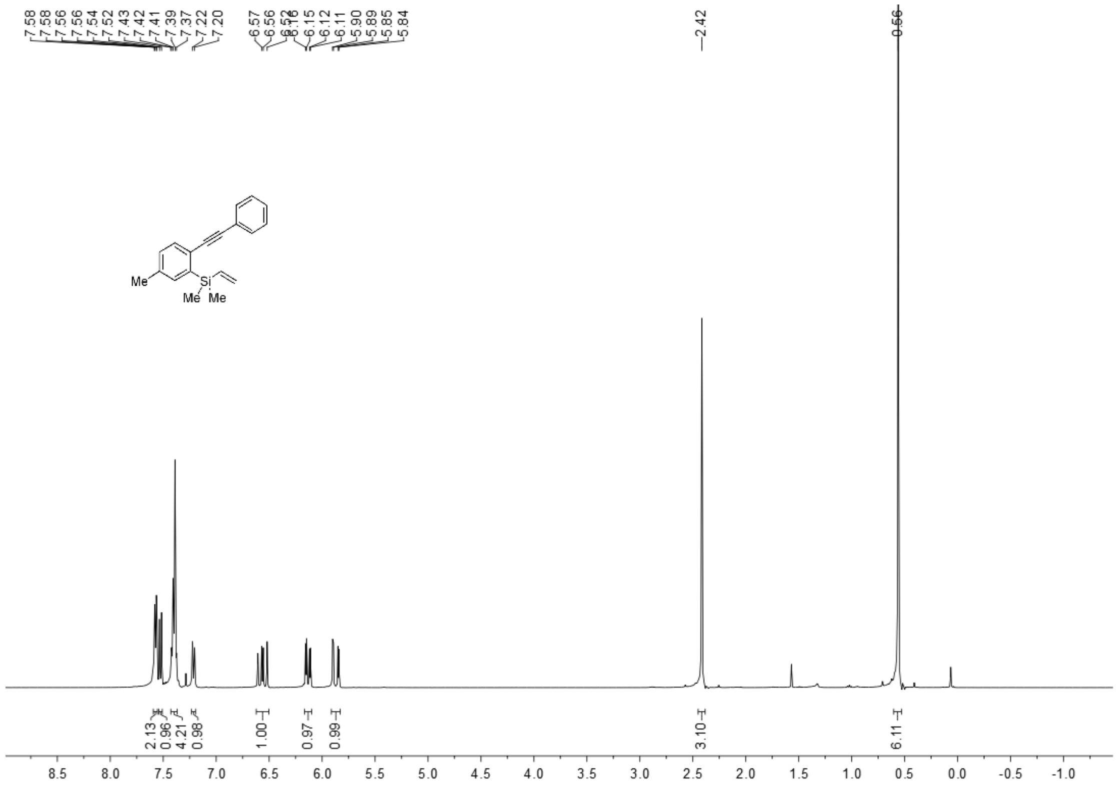

^{13}C NMR spectrum (101 MHz, $CDCl_3$) of **1n**

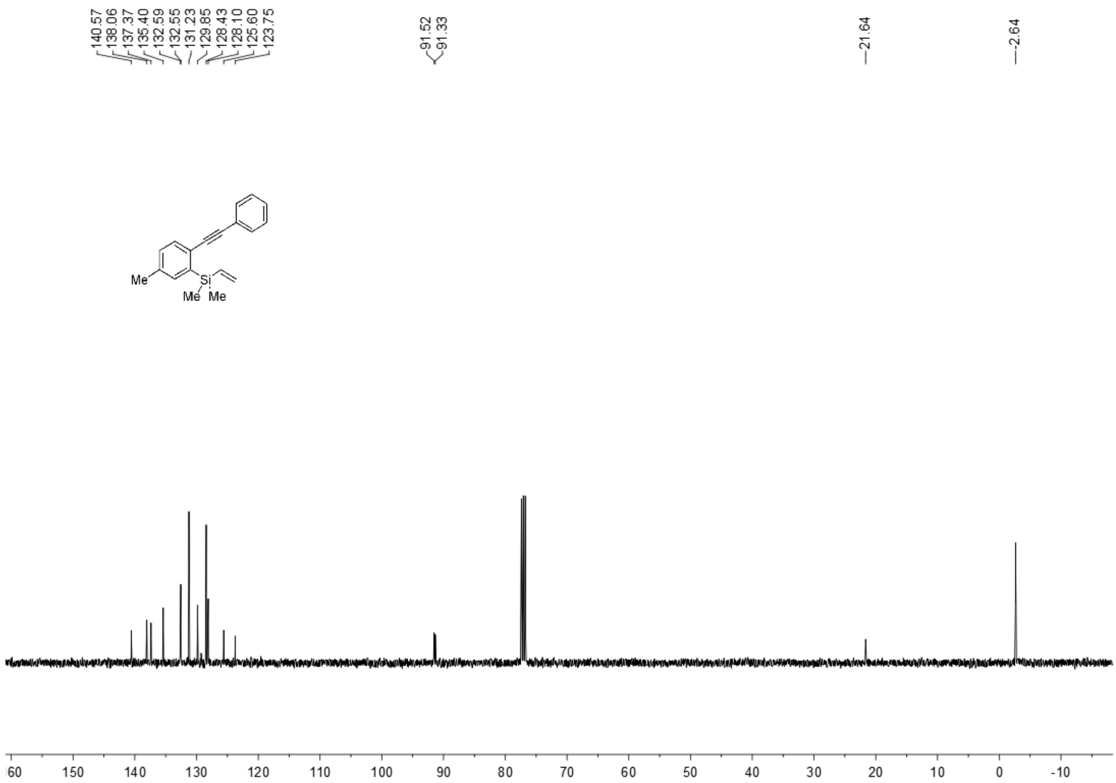

^{1}H NMR spectrum (400 MHz, $CDCl_3$) of **1o**

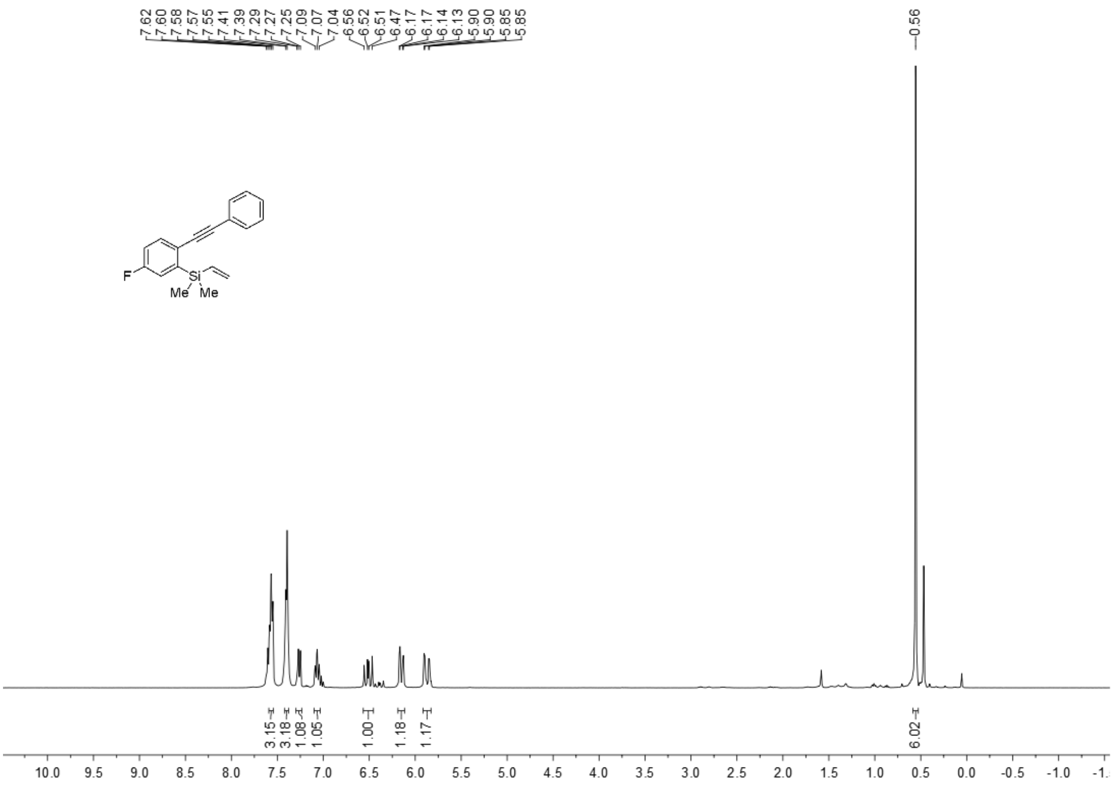

^{13}C NMR spectrum (101 MHz, $CDCl_3$) of **1o**

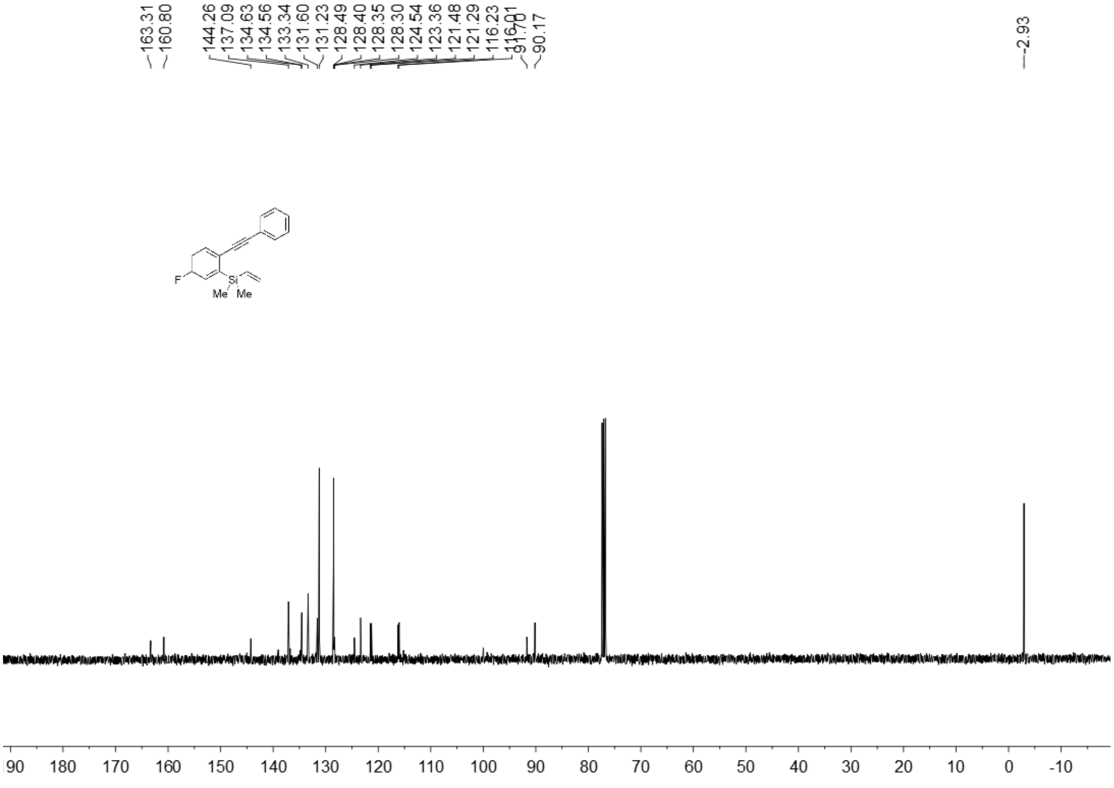

^{1}H NMR spectrum (400 MHz, $CDCl_3$) of **1p**

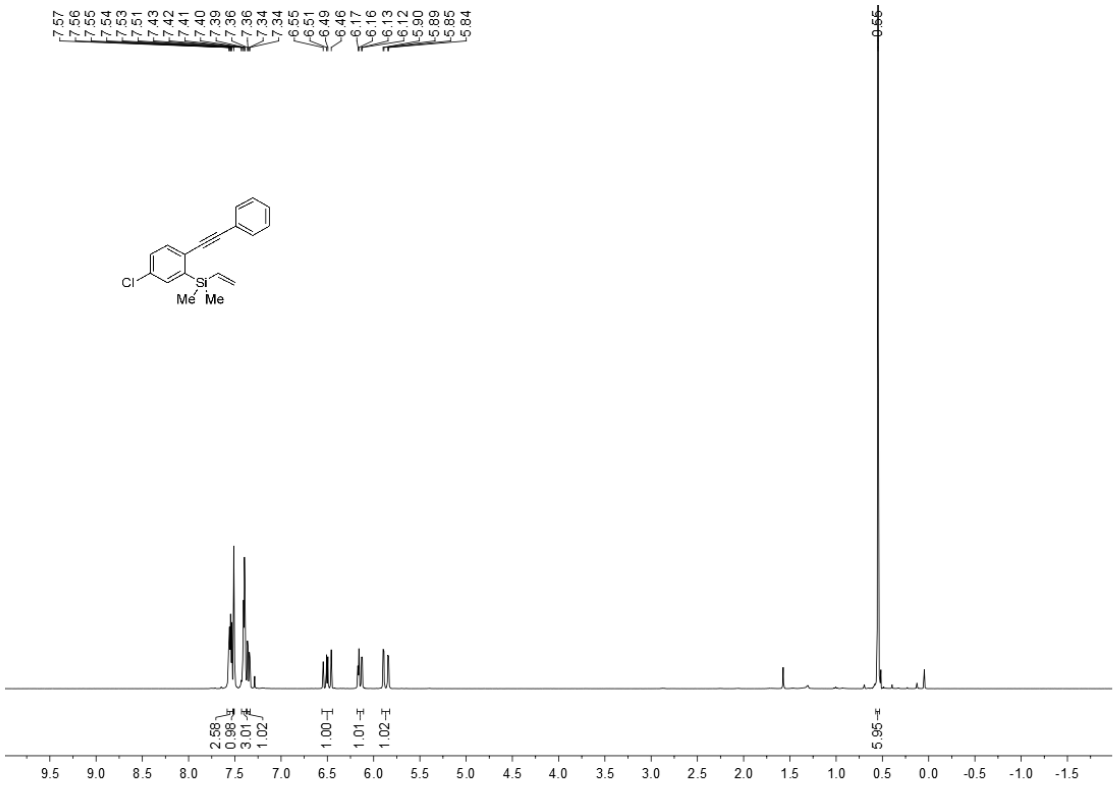

^{13}C NMR spectrum (101 MHz, $CDCl_3$) of **1p**

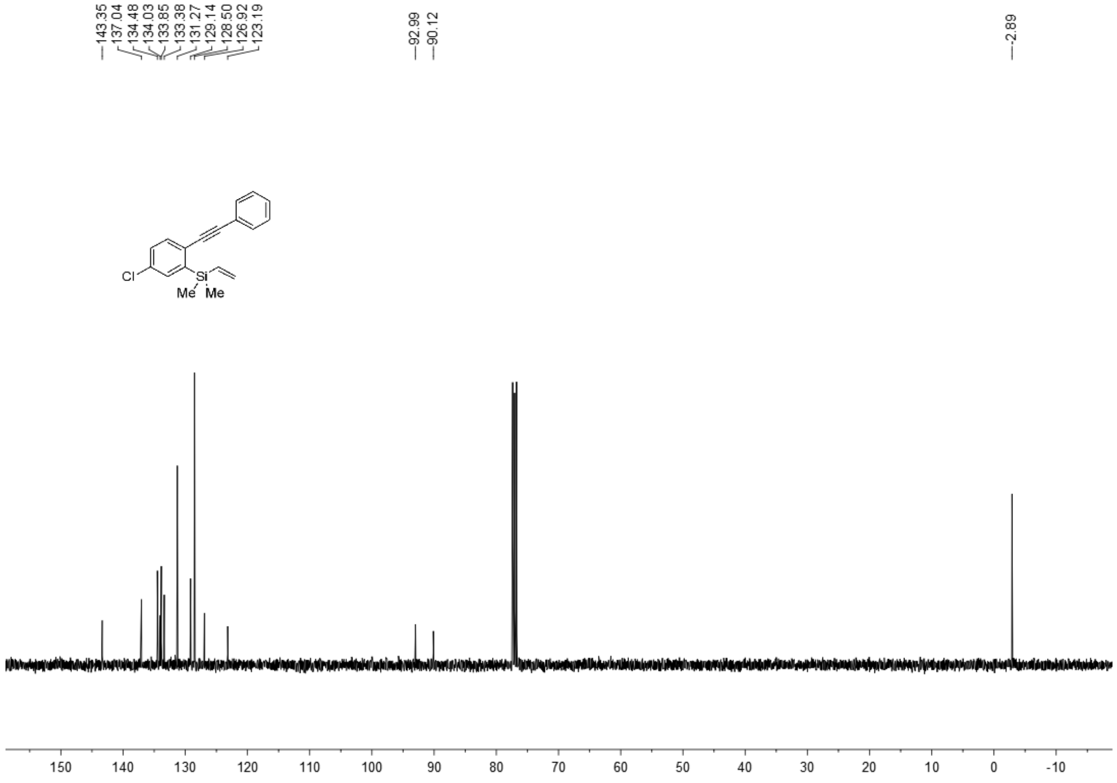

^{1}H NMR spectrum (500 MHz, $CDCl_3$) of **1q**

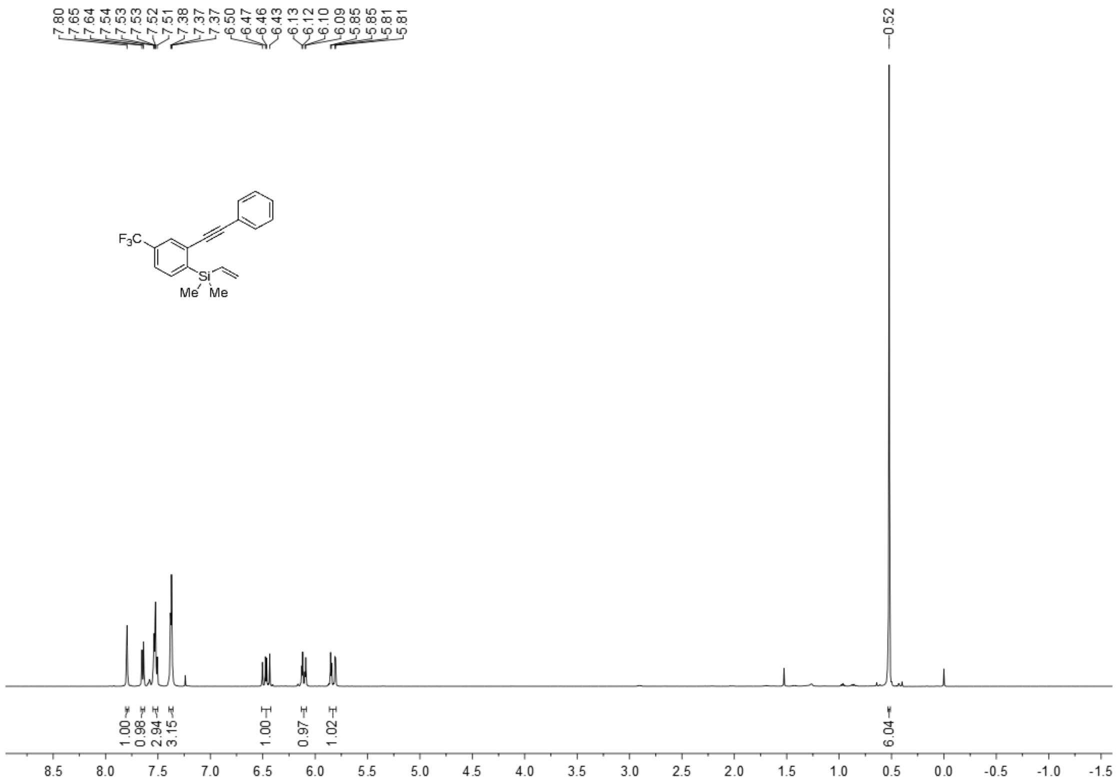

^{13}C NMR spectrum (126 MHz, $CDCl_3$) of **1q**

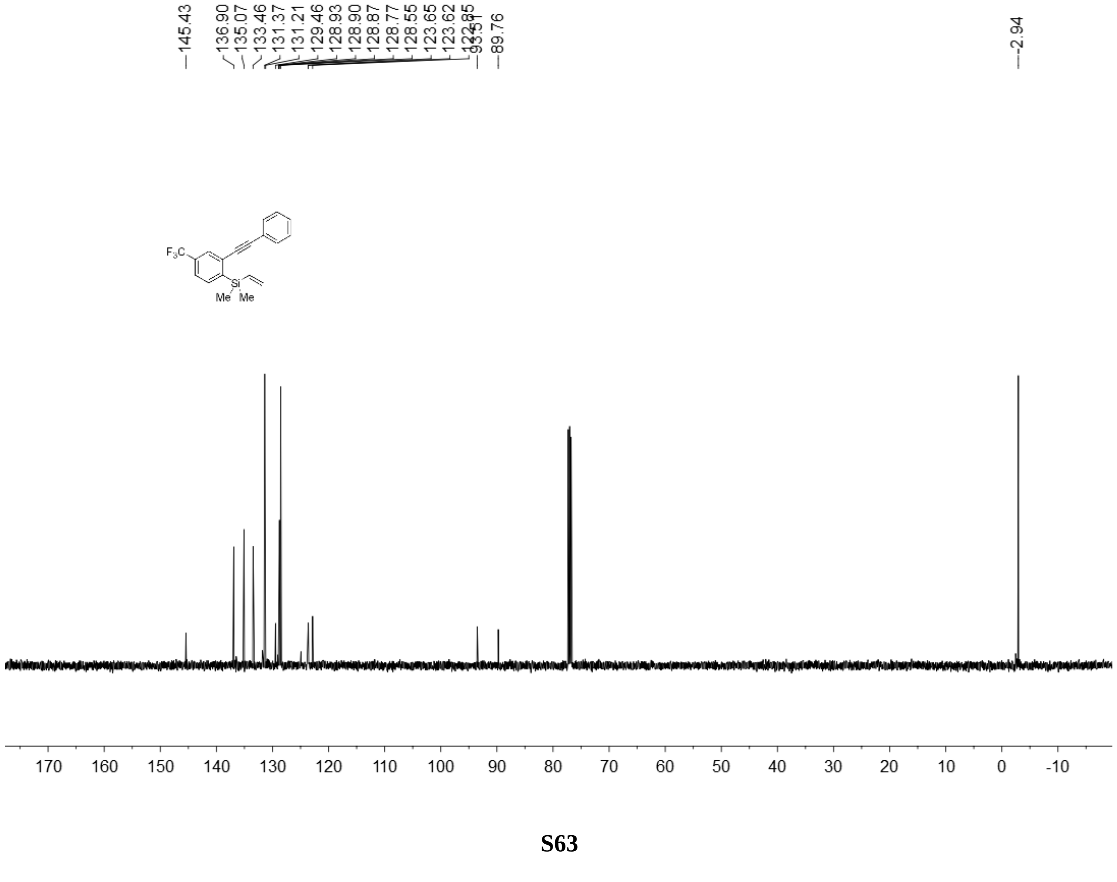

^{1}H NMR spectrum (500 MHz, $CDCl_3$) of **4**

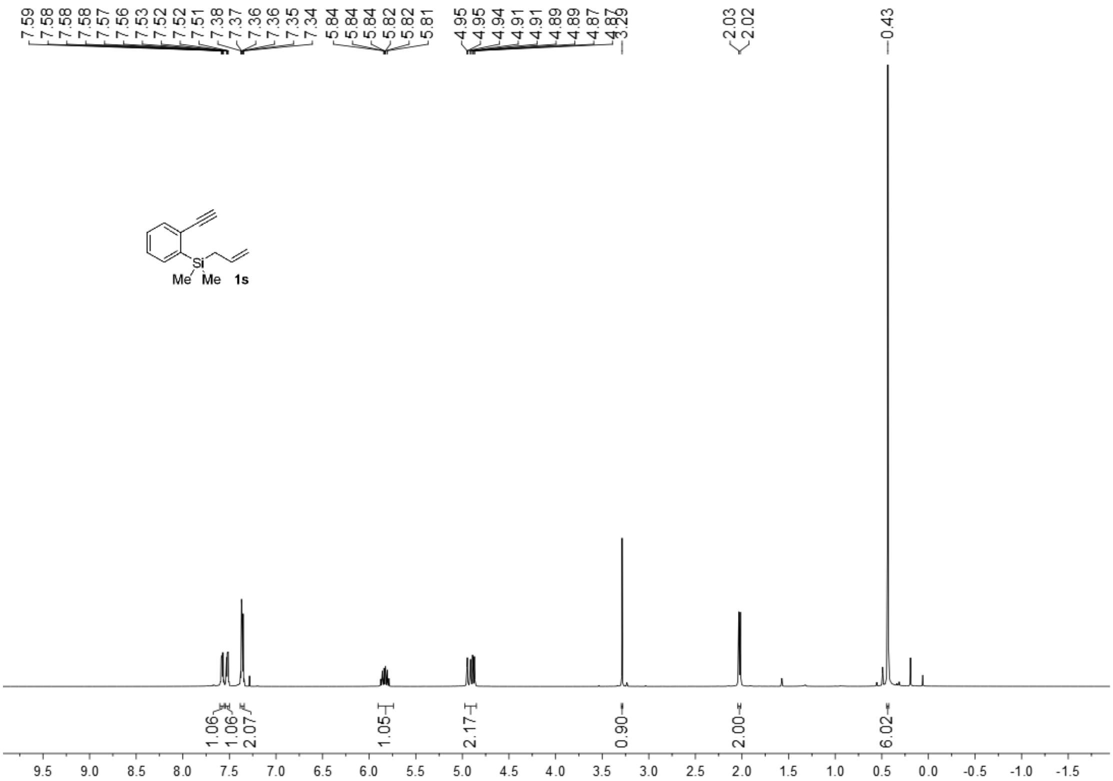

^{13}C NMR spectrum (101 MHz, $CDCl_3$) of **4**

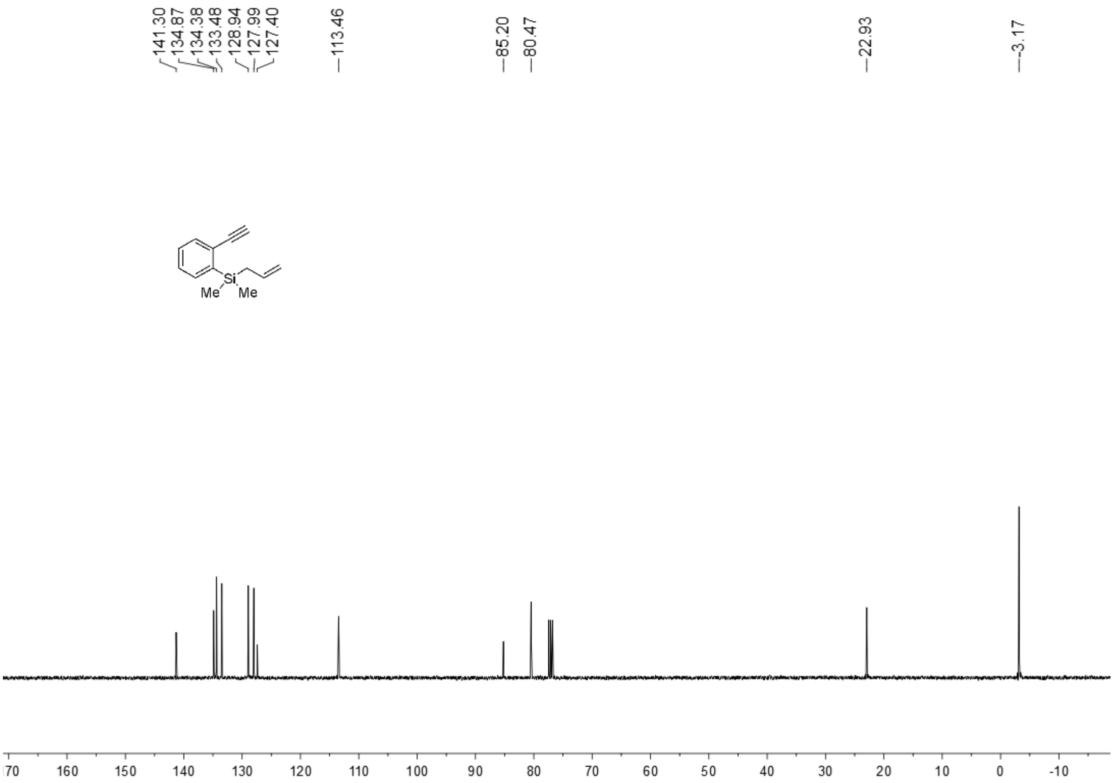

^{1}H NMR spectrum (400 MHz, $CDCl_3$) of **1r**

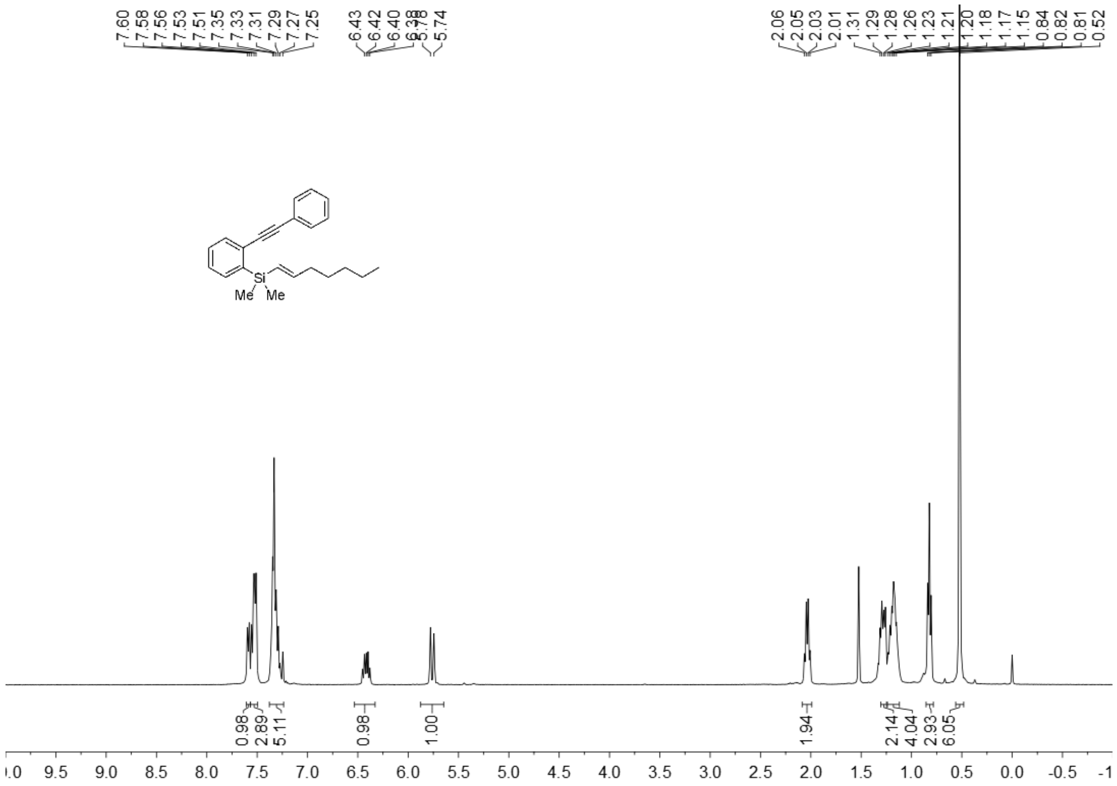

^{13}C NMR spectrum (101 MHz, $CDCl_3$) of **1r**

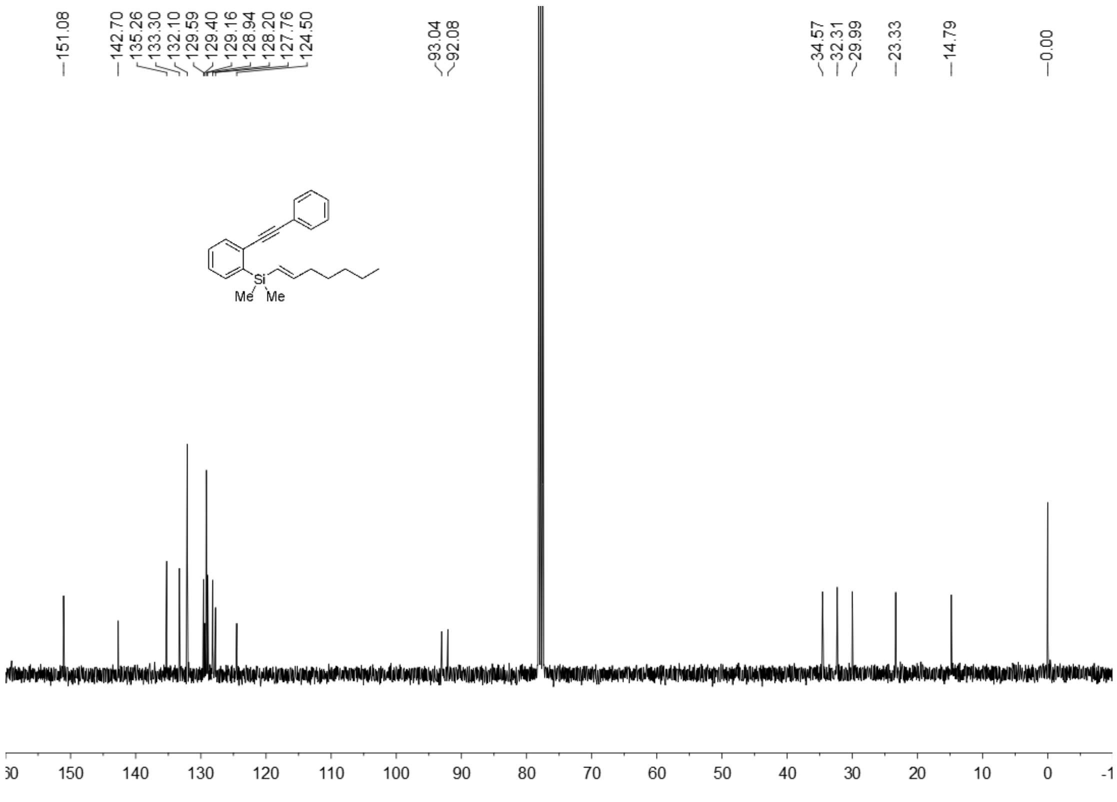

^{1}H NMR spectrum (400 MHz, $CDCl_3$) of **1u**

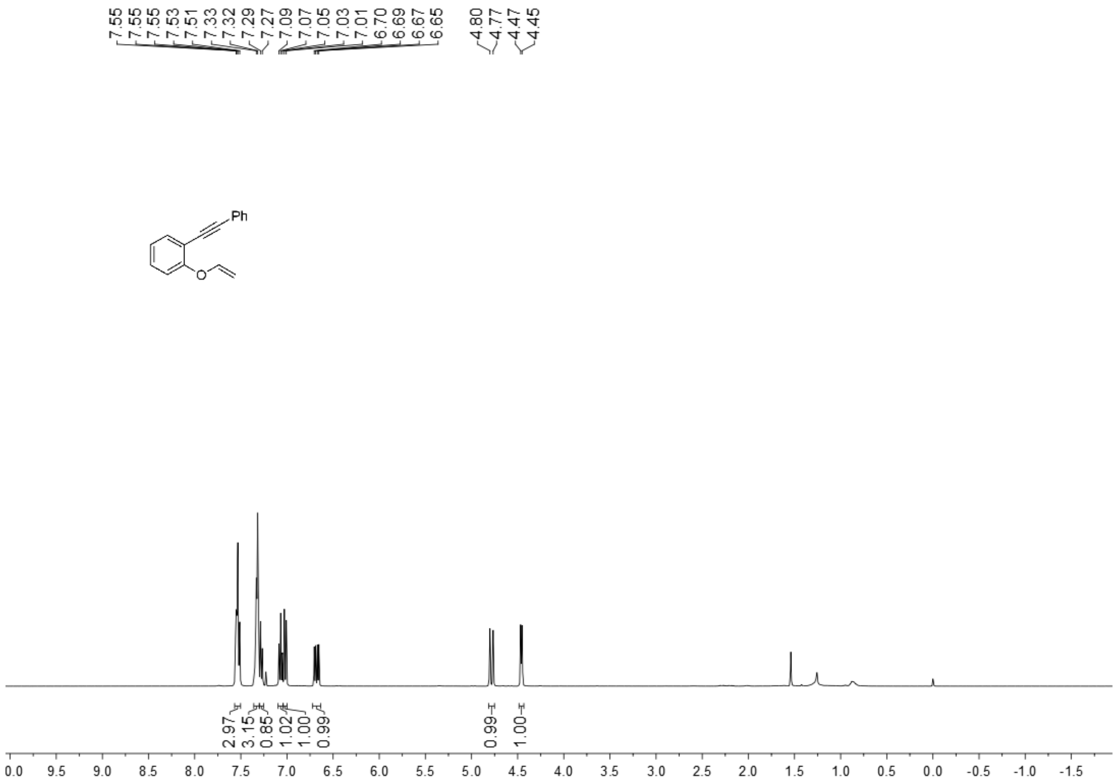

^{13}C NMR spectrum (101 MHz, $CDCl_3$) of **1u**

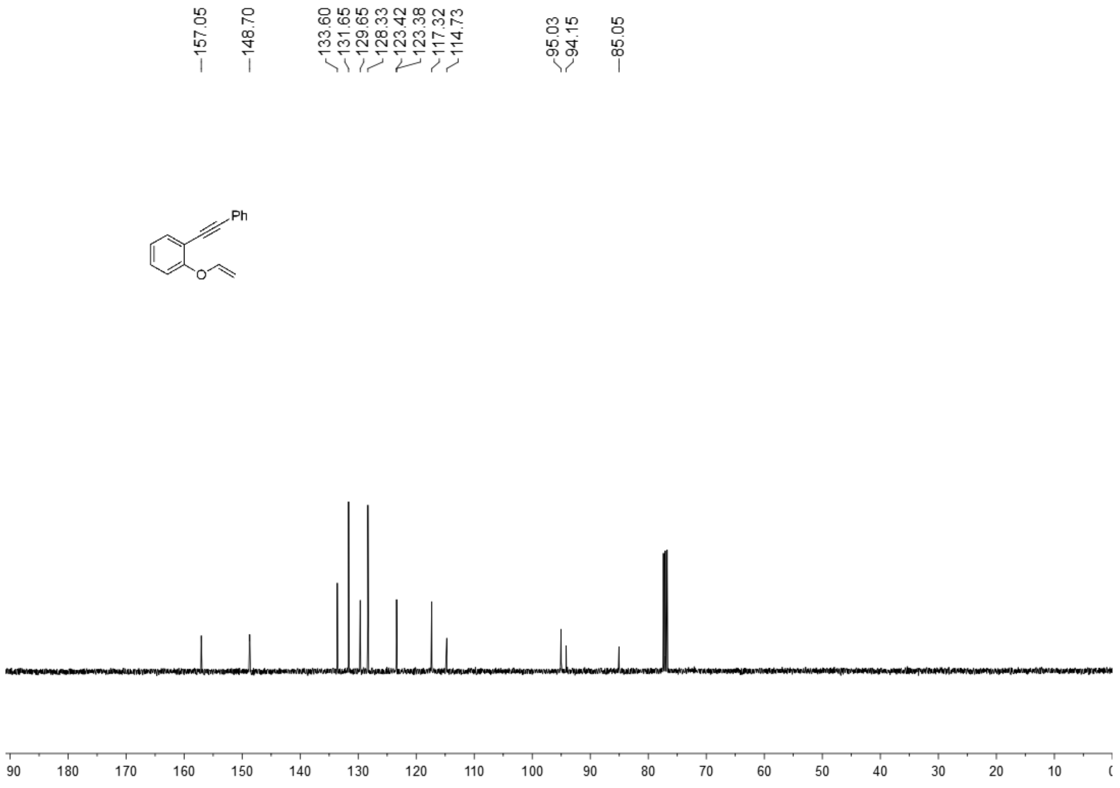

^{1}H NMR spectrum (400 MHz, $CDCl_3$) of **S5**

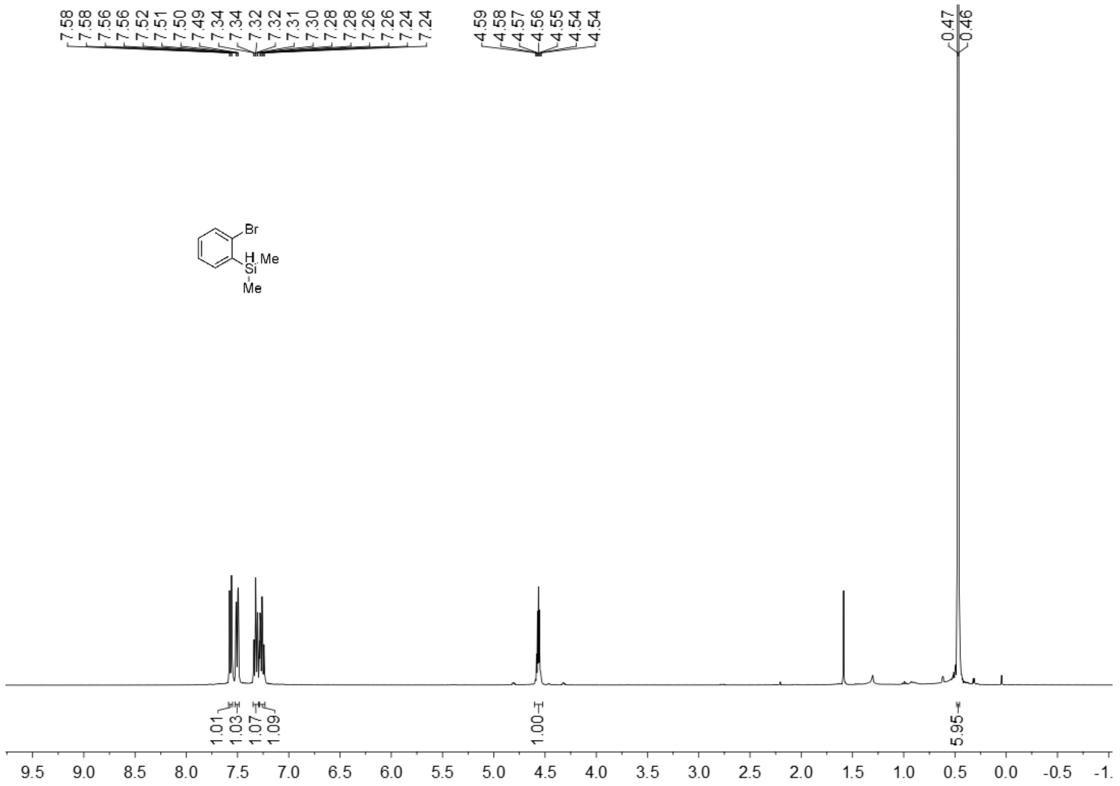

^{13}C NMR spectrum (101 MHz, $CDCl_3$) of **S5**

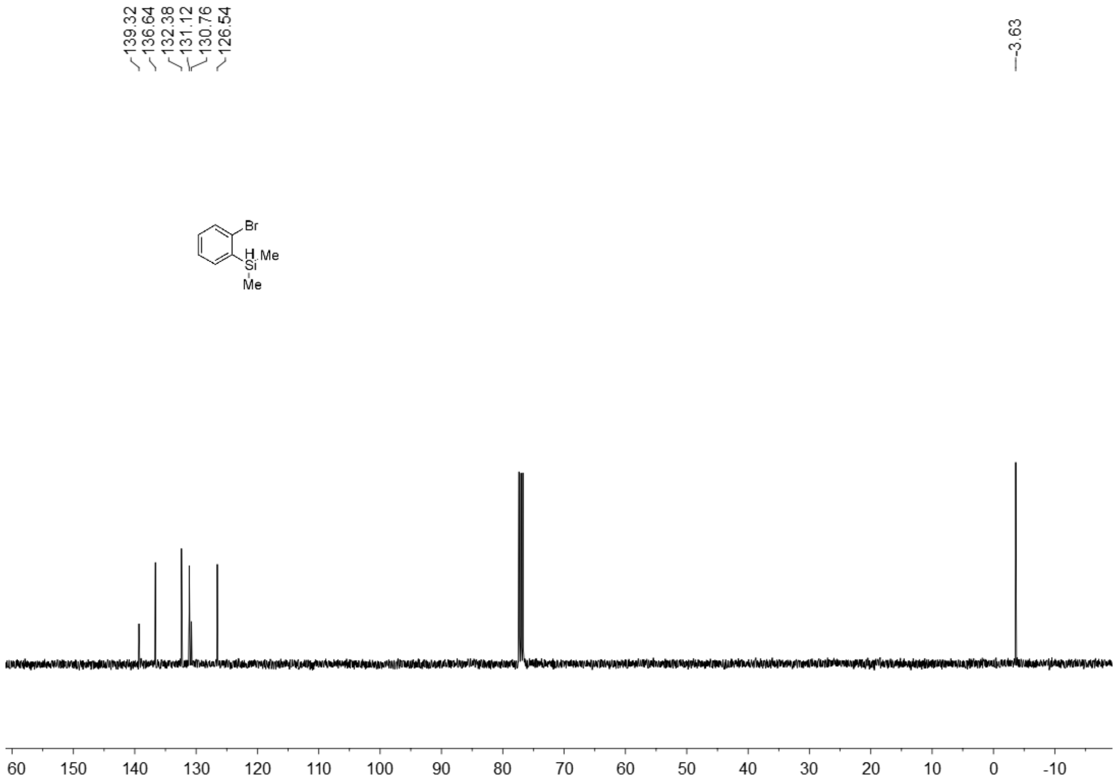

^{1}H NMR spectrum (400 MHz, $CDCl_3$) of **3a**

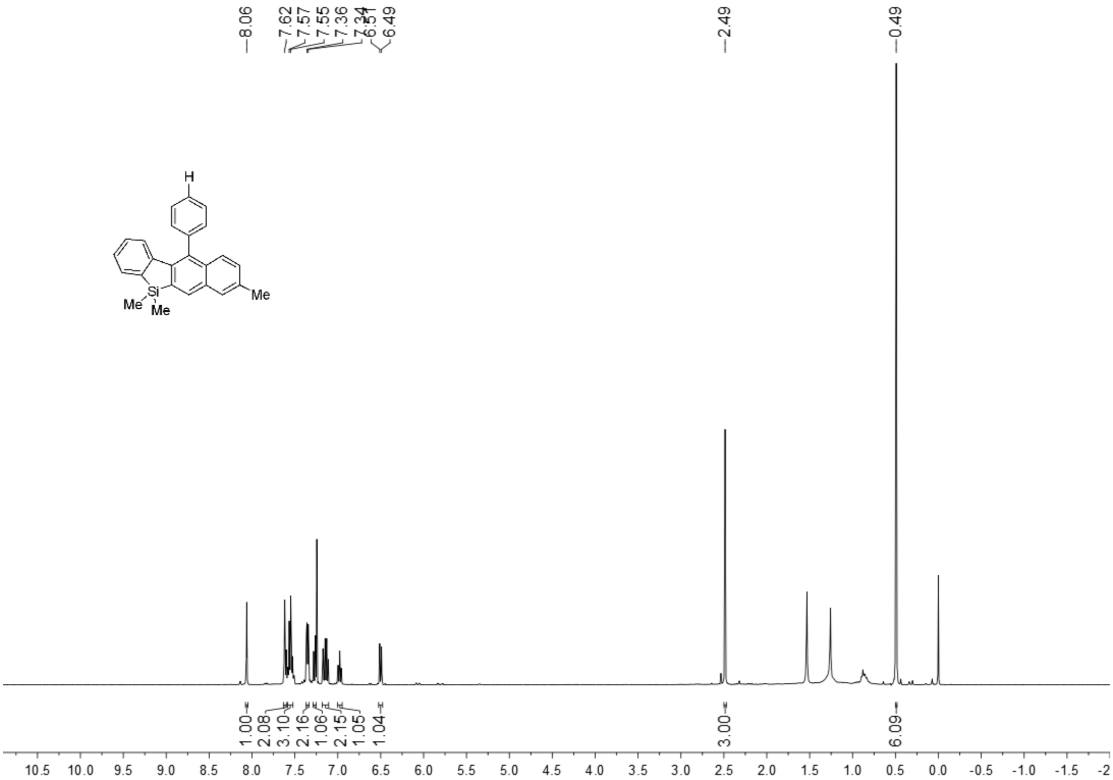

^{13}C NMR spectrum (126 MHz, $CDCl_3$) of **3a**

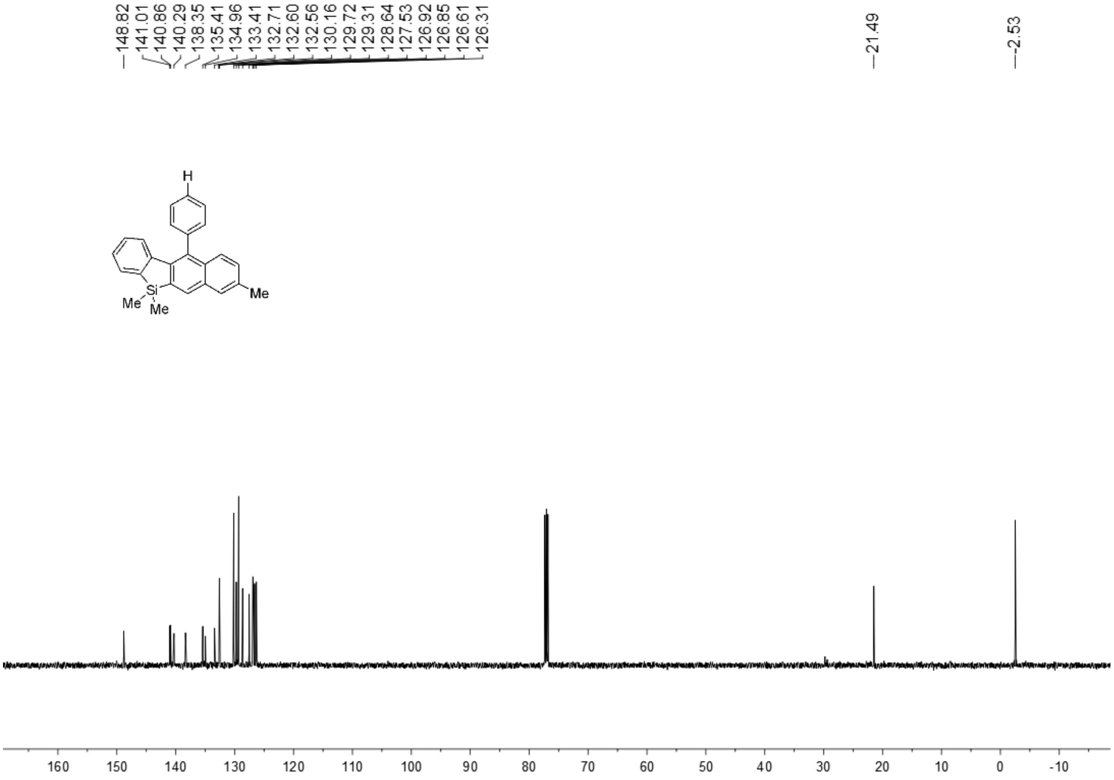

^{1}H NMR spectrum (400 MHz, $CDCl_3$) of **3b**

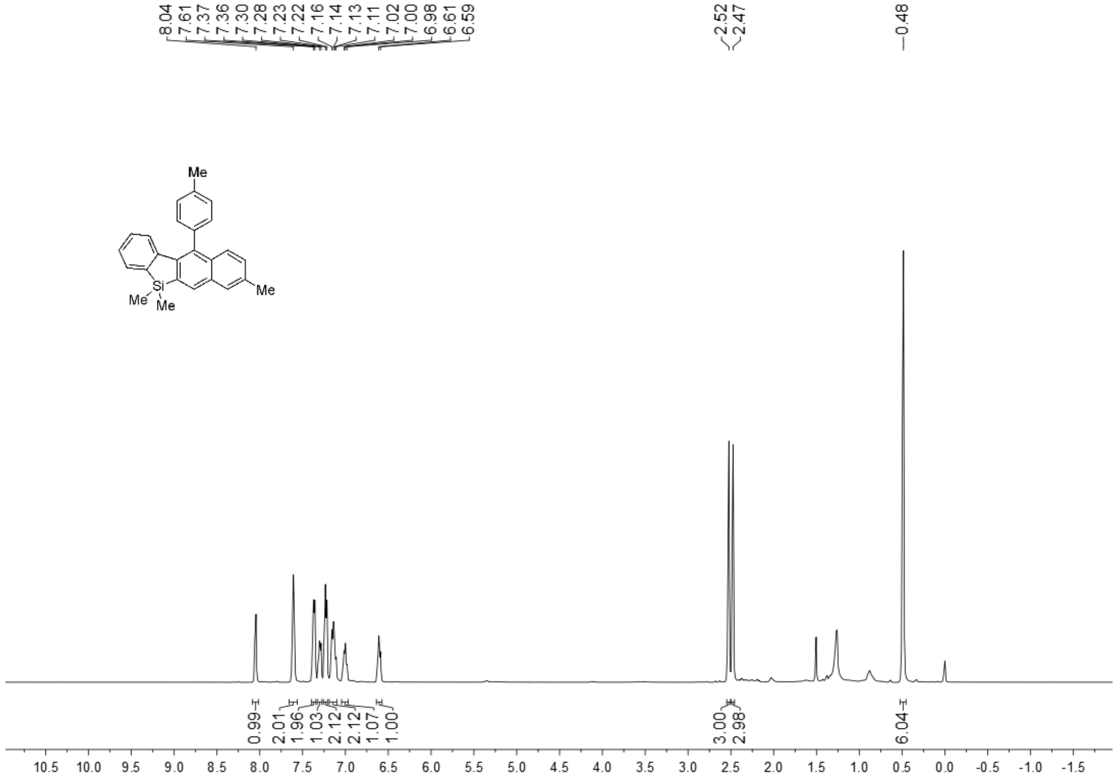

^{13}C NMR spectrum (101 MHz, $CDCl_3$) of **3b**

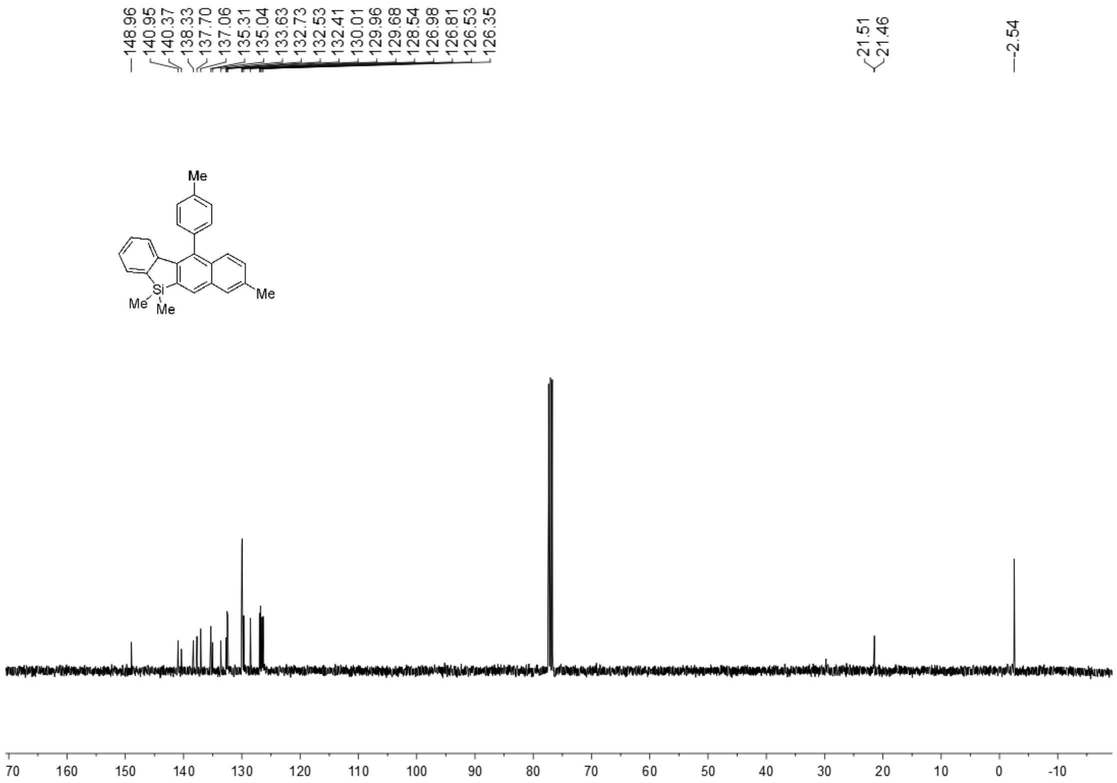

^{1}H NMR spectrum (400 MHz, $CDCl_3$) of **3c**

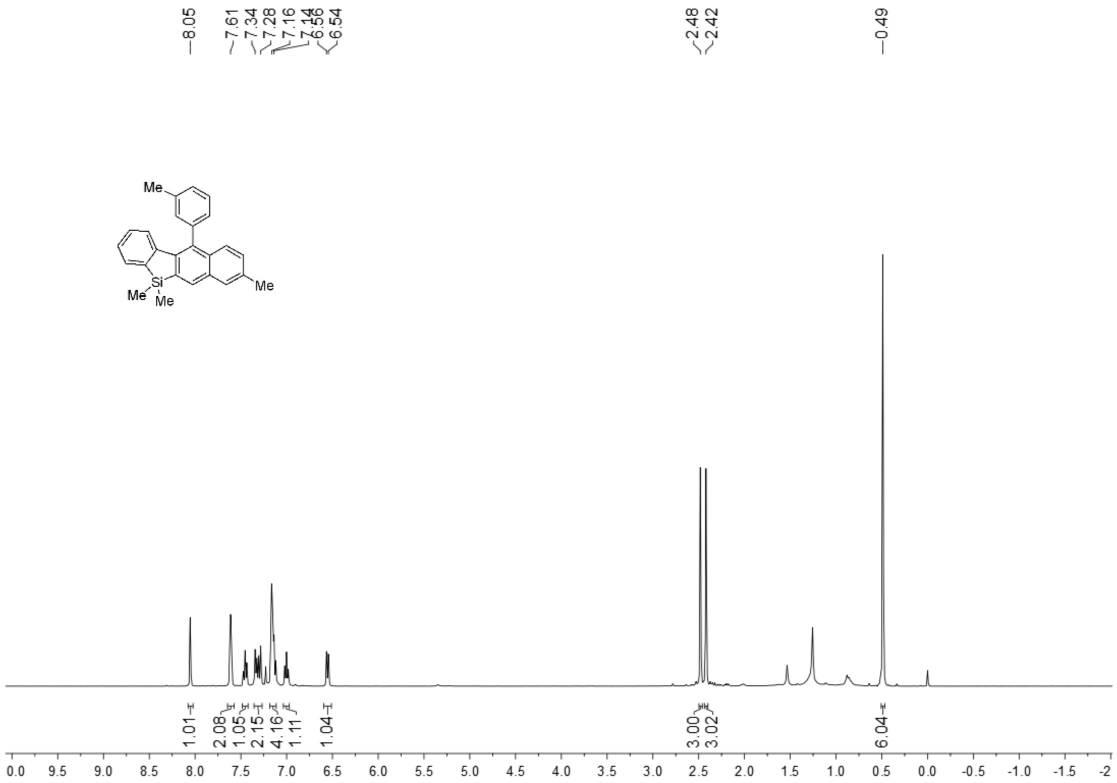

^{13}C NMR spectrum (101 MHz, $CDCl_3$) of **3c**

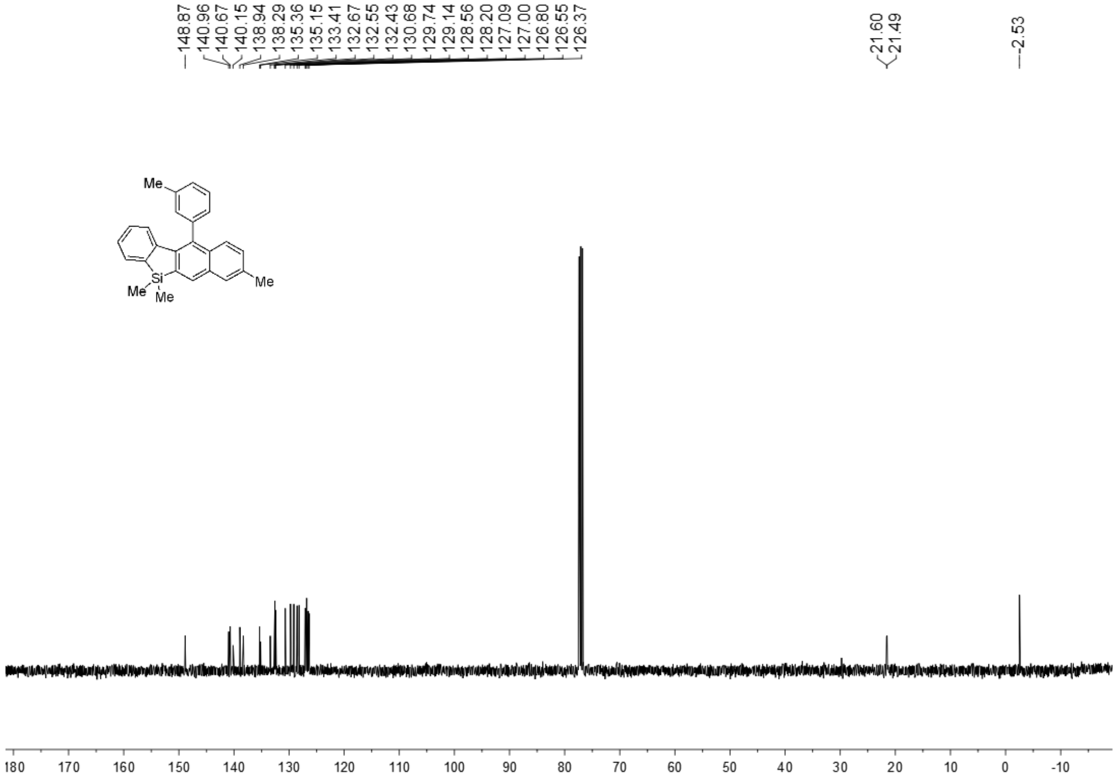

^{1}H NMR spectrum (500 MHz, $CDCl_3$) of **3d**

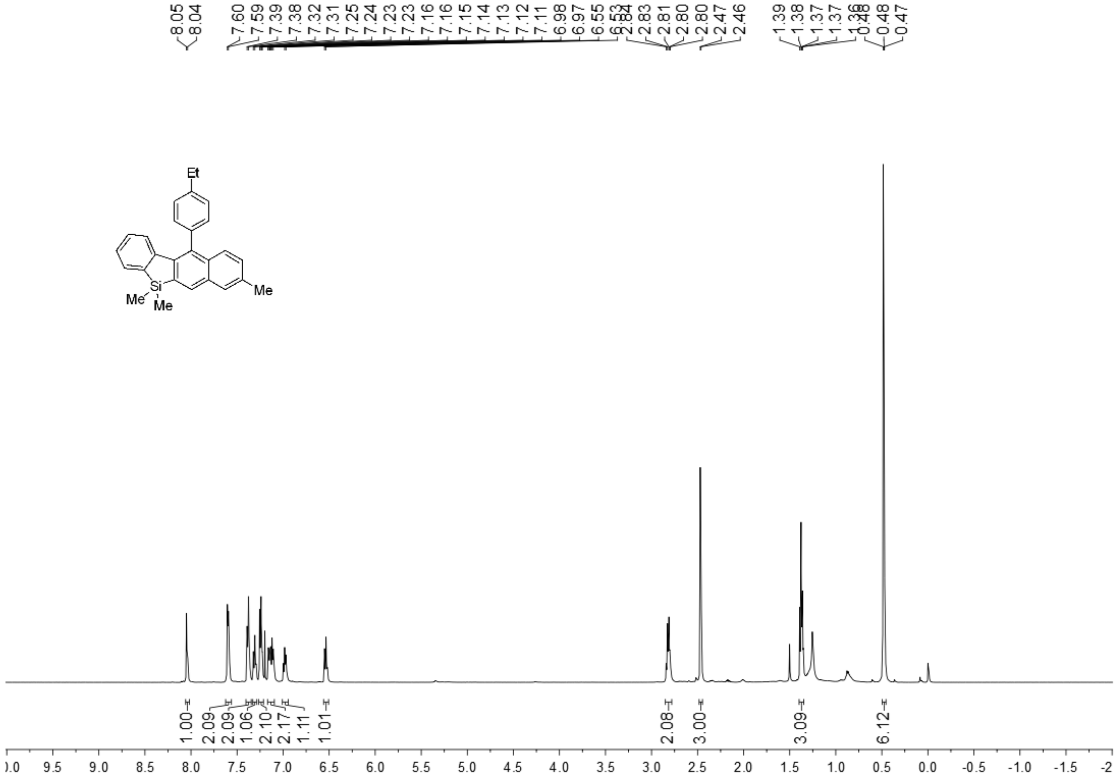

^{13}C NMR spectrum (126 MHz, $CDCl_3$) of **3d**

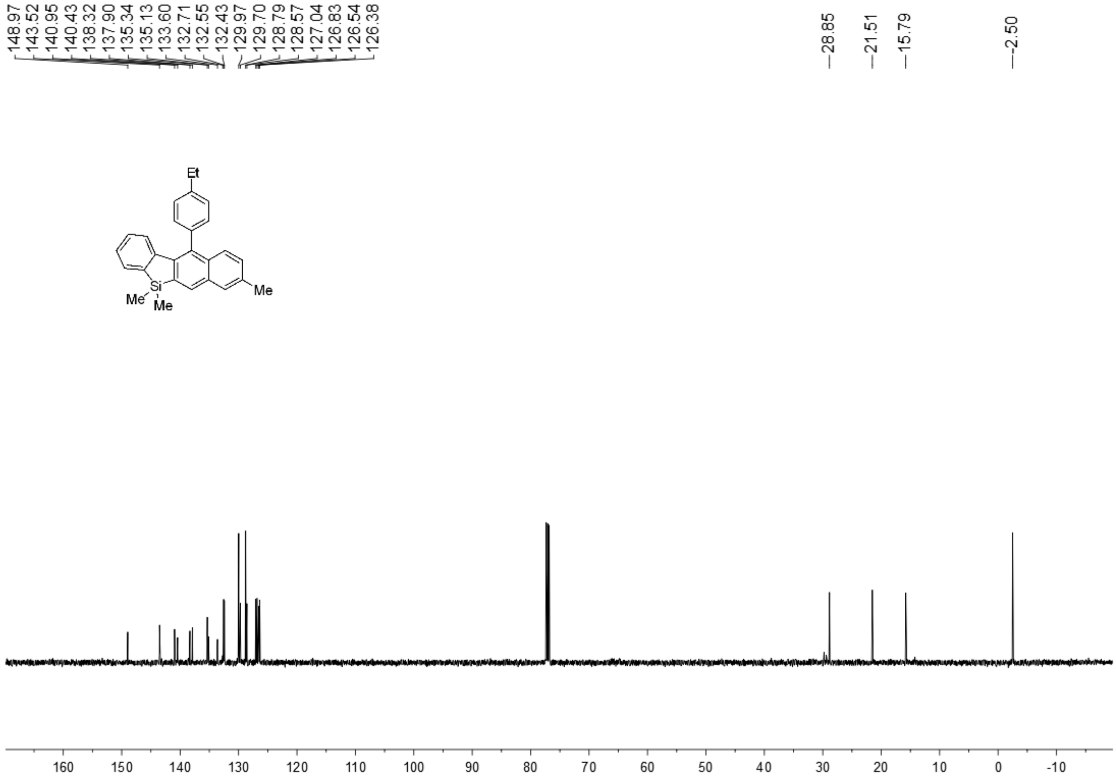

^{1}H NMR spectrum (500 MHz, $CDCl_3$) of **3e**

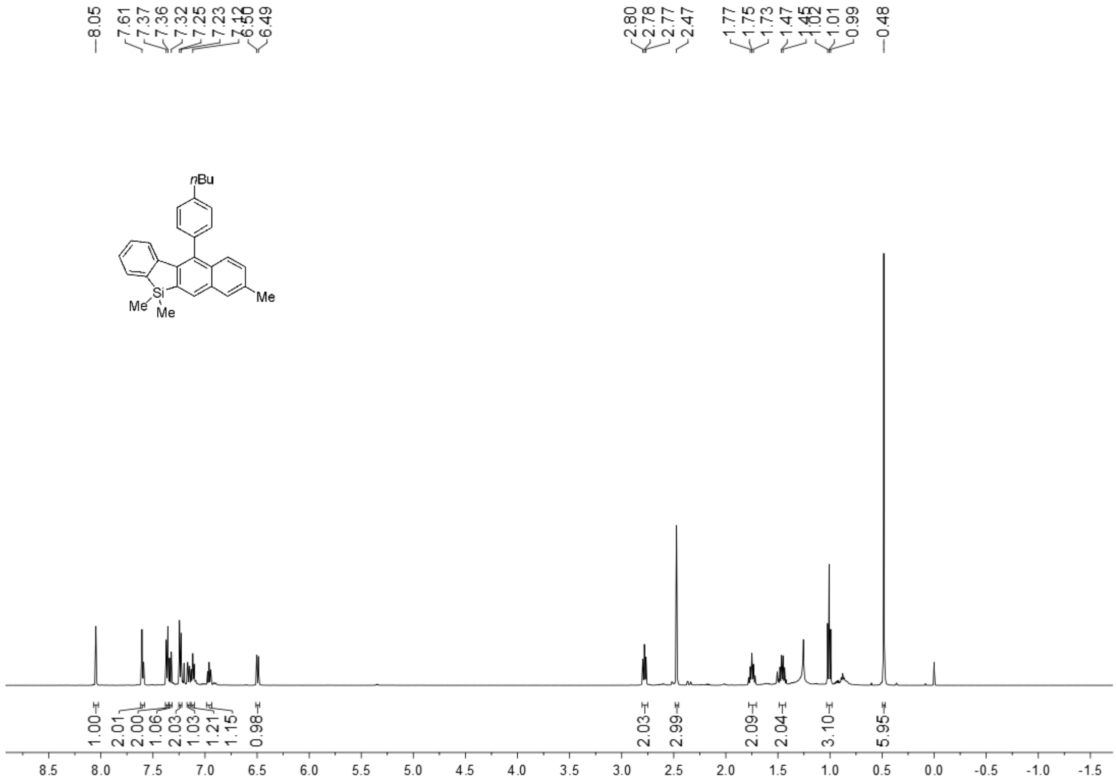

^{13}C NMR spectrum (126 MHz, $CDCl_3$) of **3e**

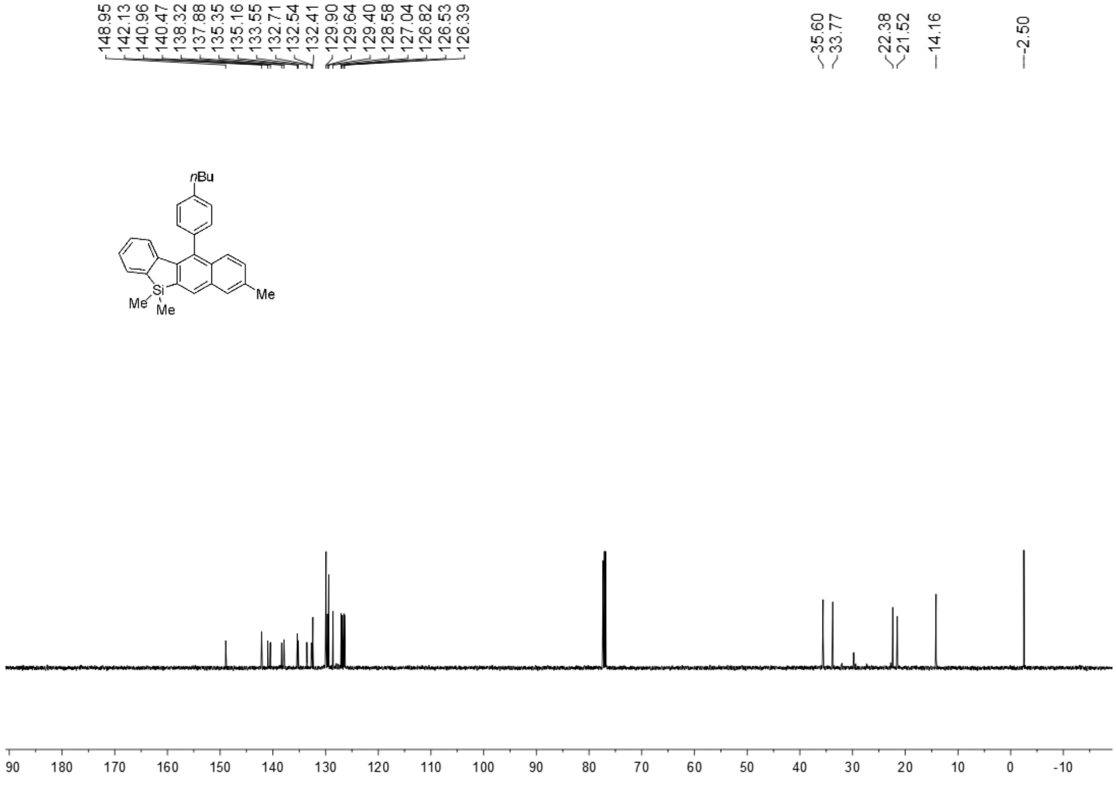

^{1}H NMR spectrum (500 MHz, $CDCl_3$) of **3f**

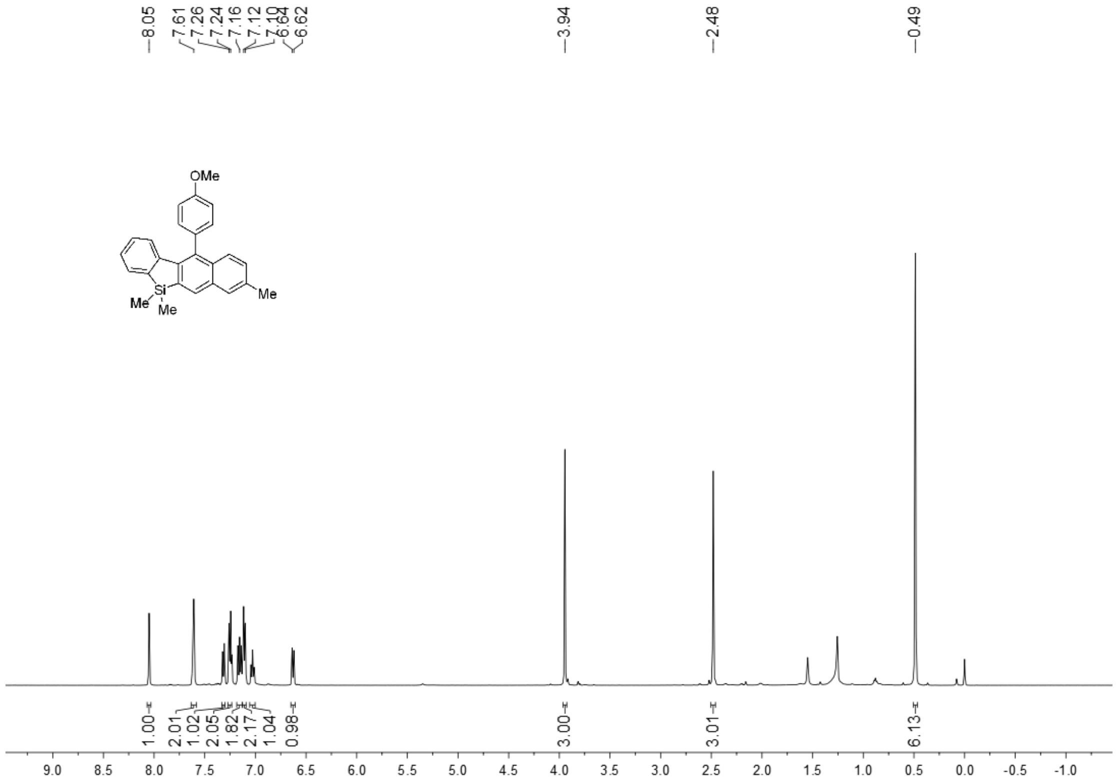

^{13}C NMR spectrum (126 MHz, $CDCl_3$) of **3f**

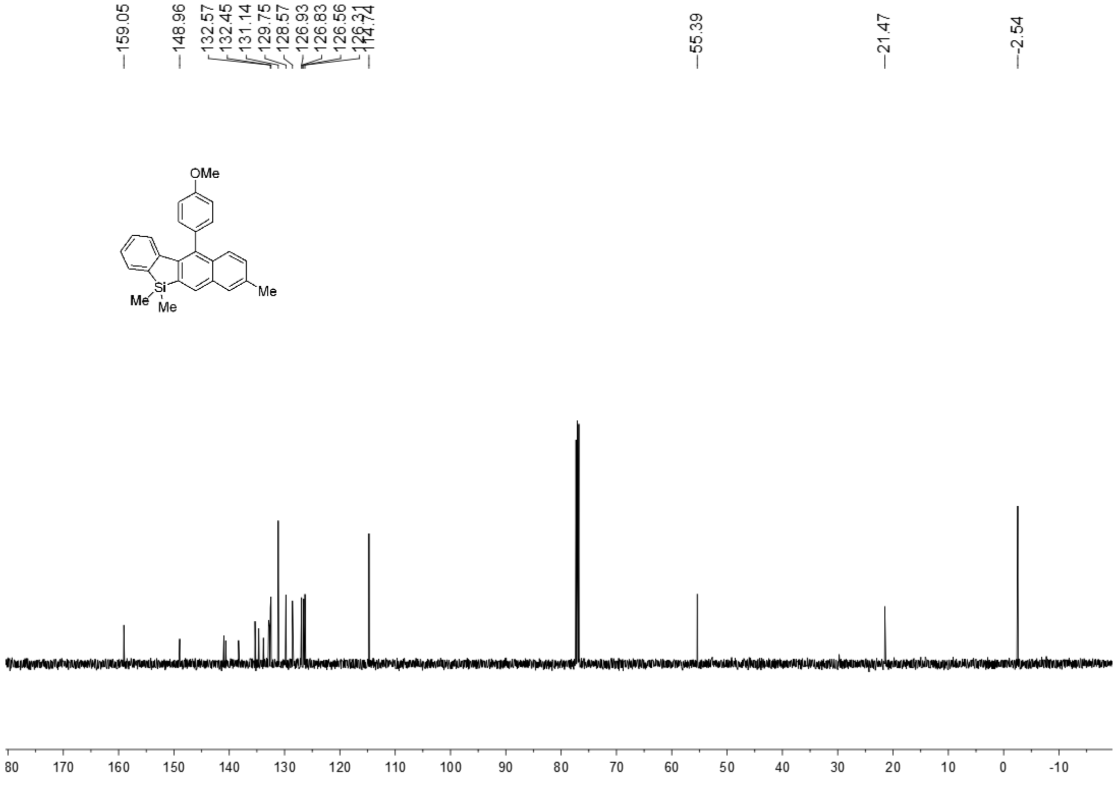

^{1}H NMR spectrum (500 MHz, $CDCl_3$) of **3g**

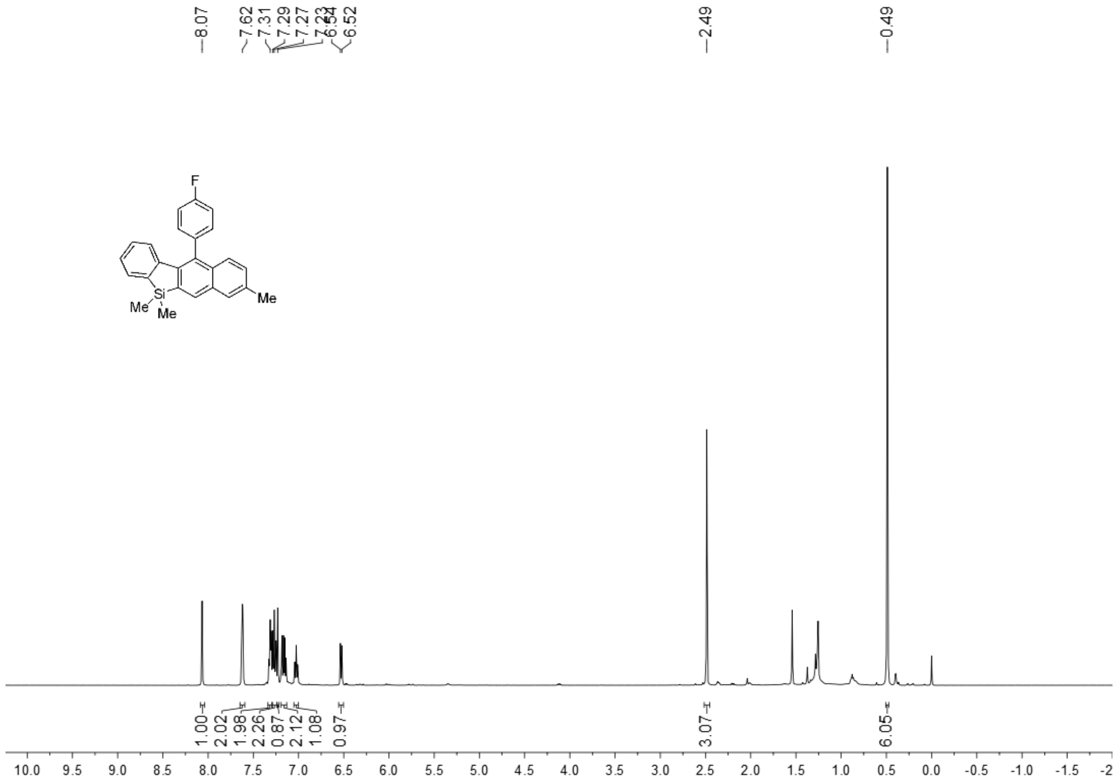

^{13}C NMR spectrum (126 MHz, $CDCl_3$) of **3g**

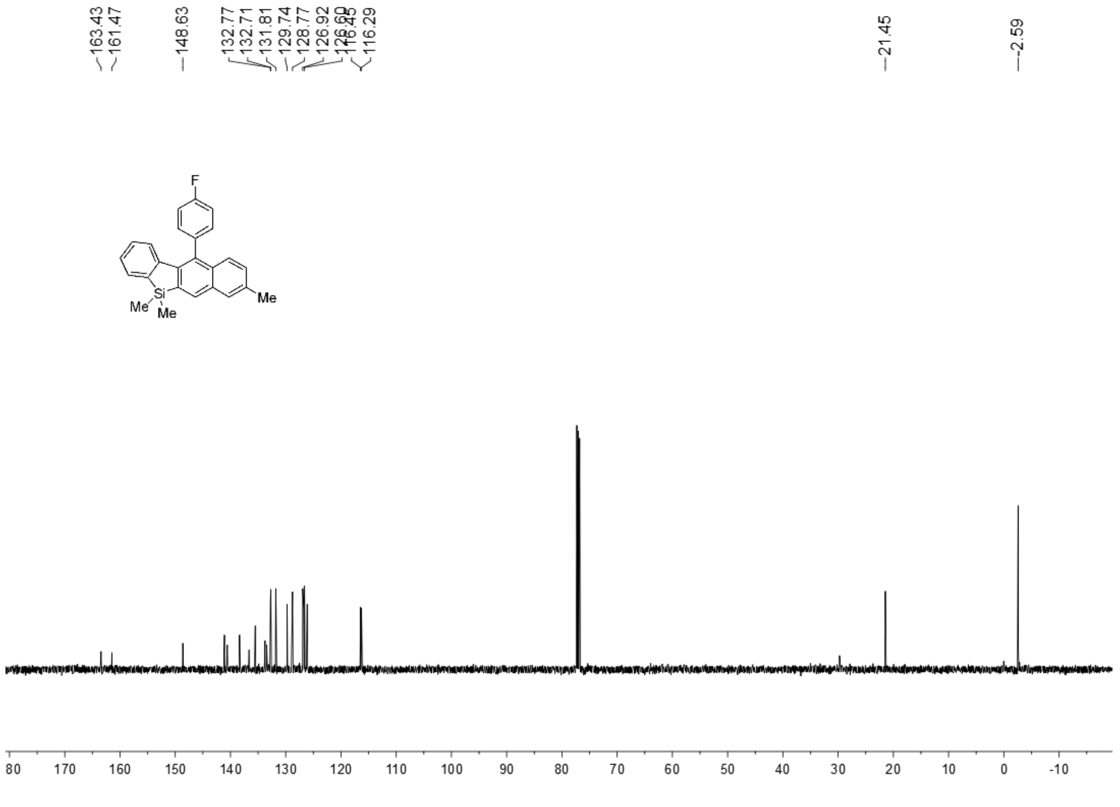

^{1}H NMR spectrum (500 MHz, $CDCl_3$) of **3h**

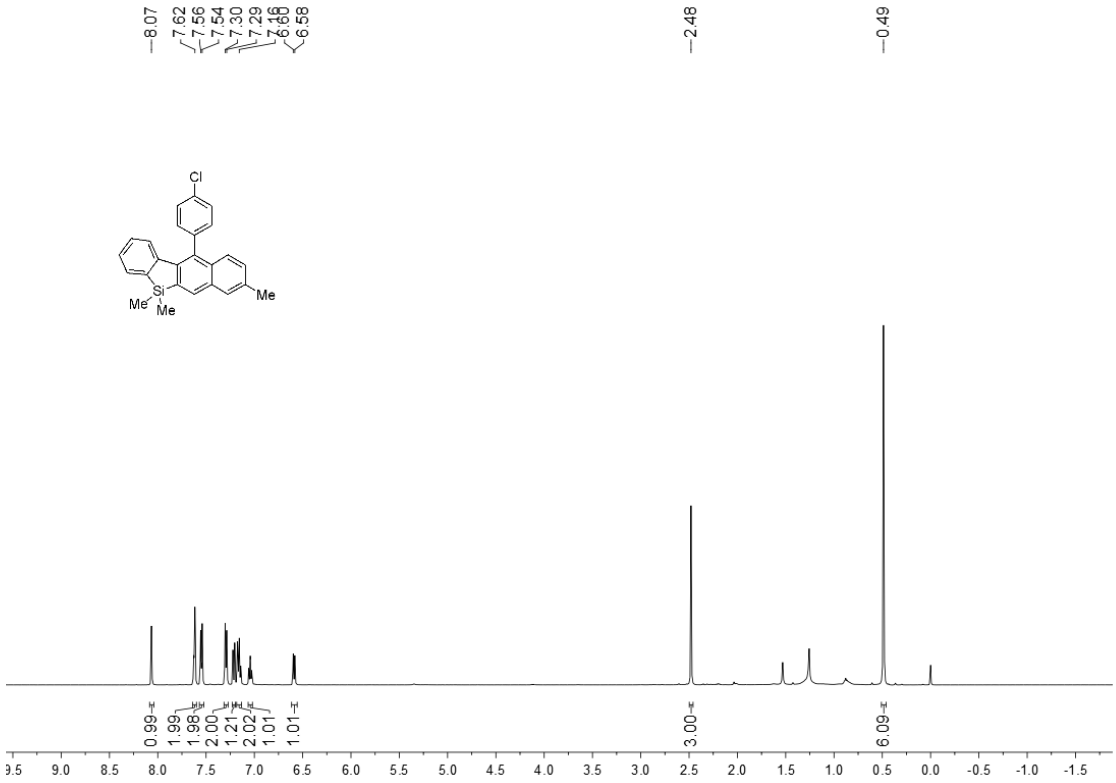

^{13}C NMR spectrum (126 MHz, $CDCl_3$) of **3h**

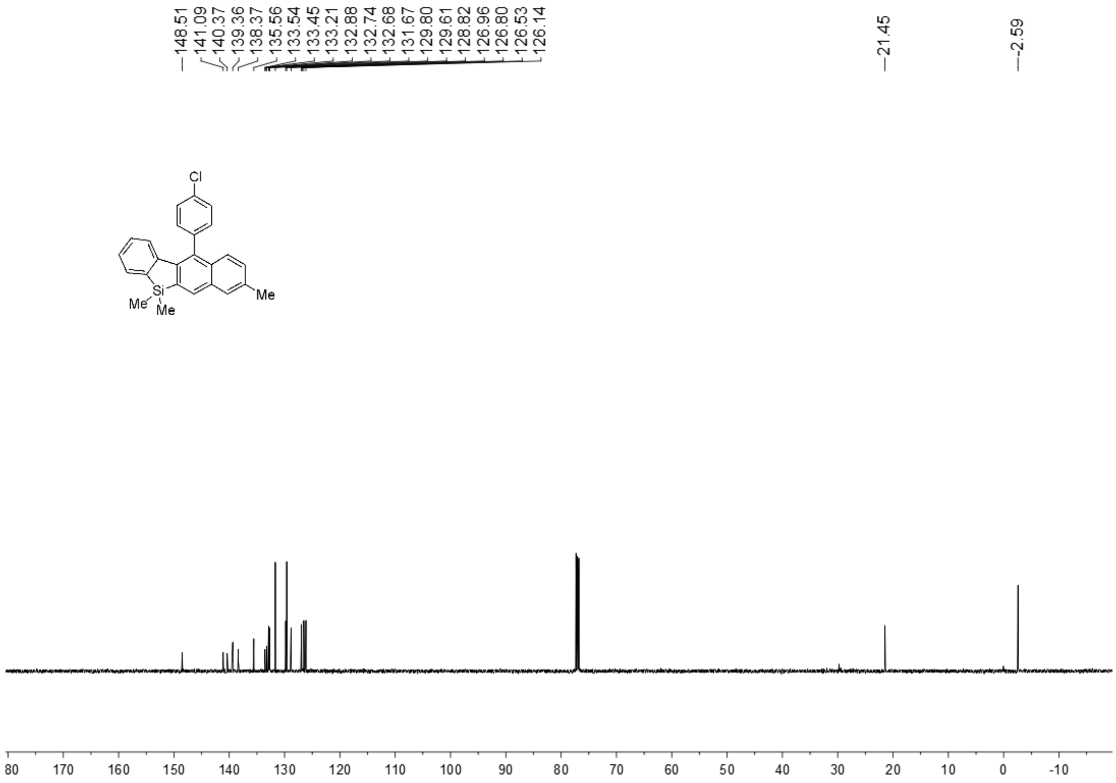

^{1}H NMR spectrum (500 MHz, $CDCl_3$) of **3i**

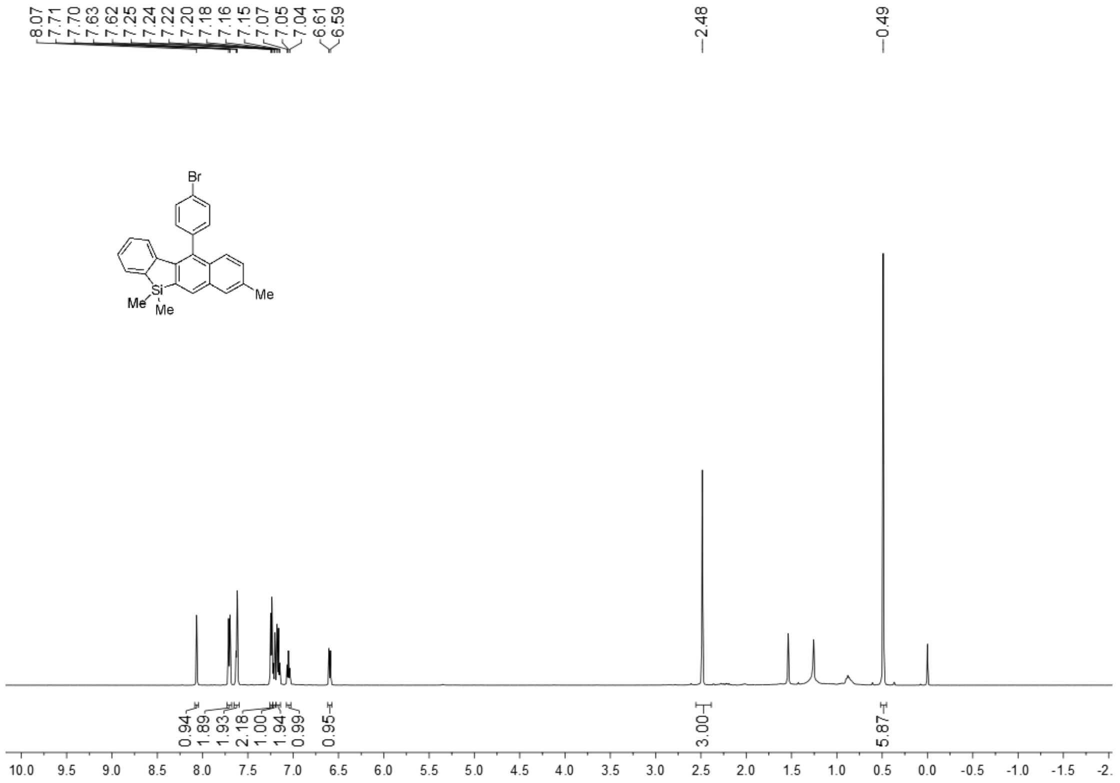

^{13}C NMR spectrum (126 MHz, $CDCl_3$) of **3i**

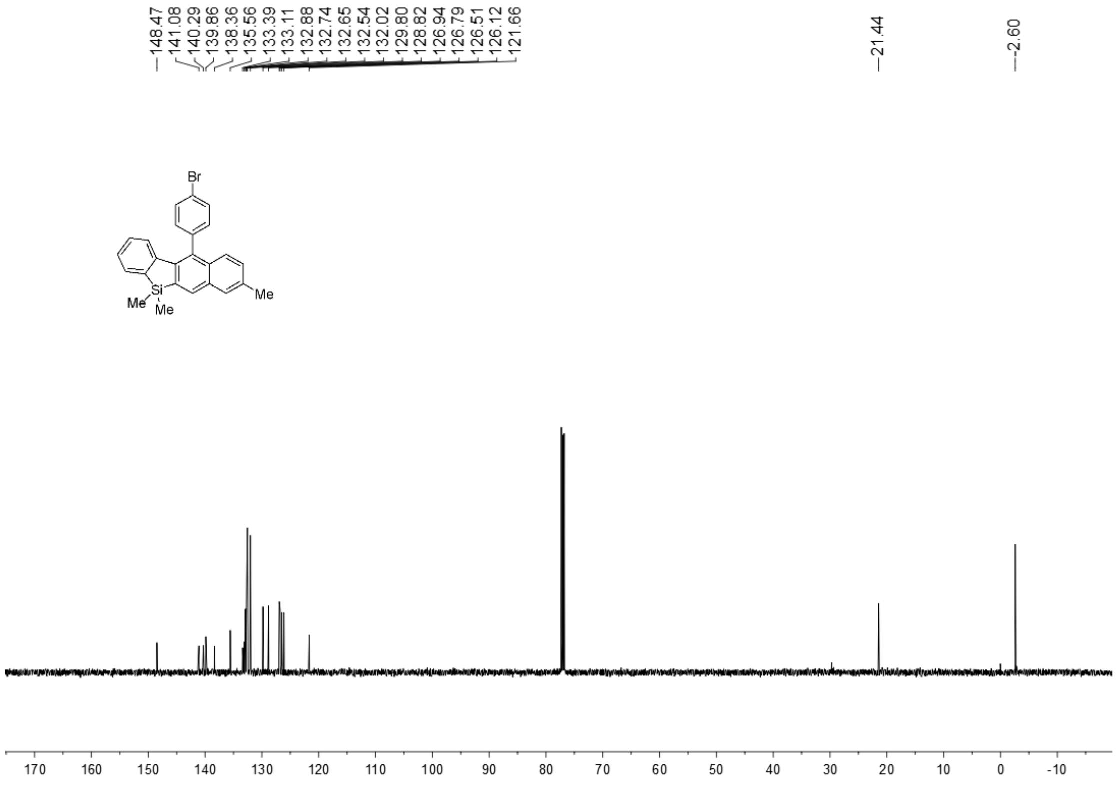

^{1}H NMR spectrum (400 MHz, $CDCl_3$) of **3j**

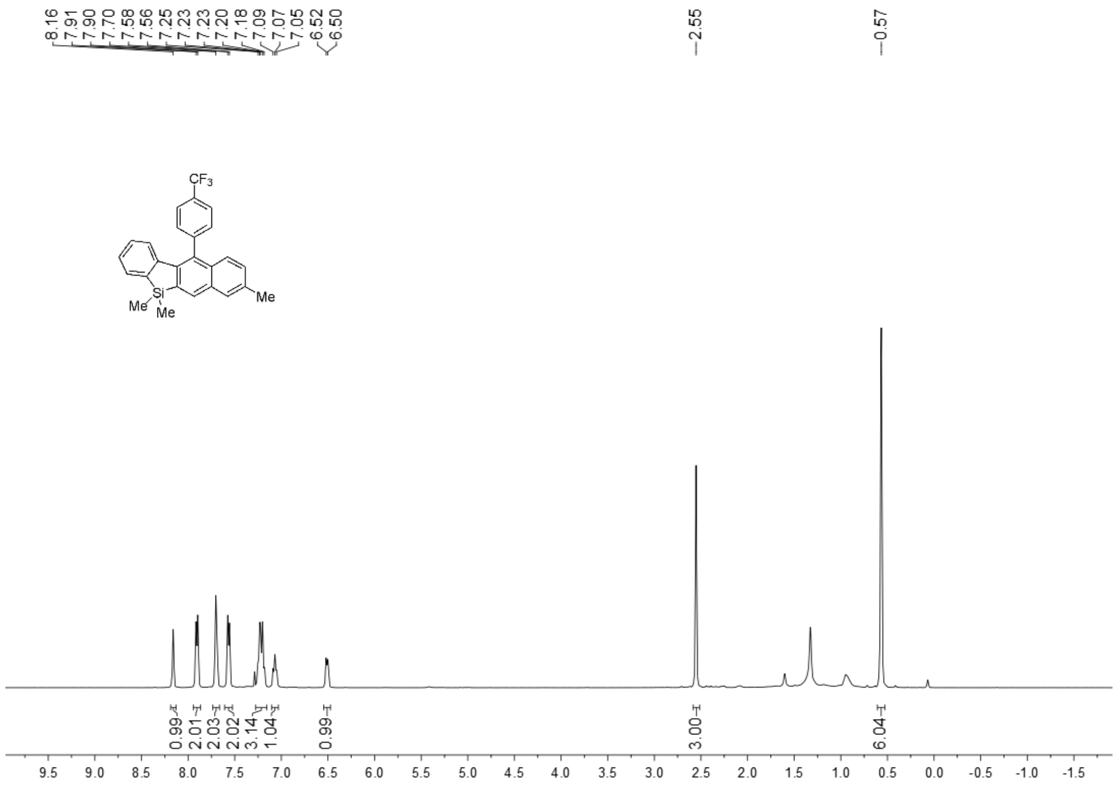

^{13}C NMR spectrum (101 MHz, $CDCl_3$) of **3j**

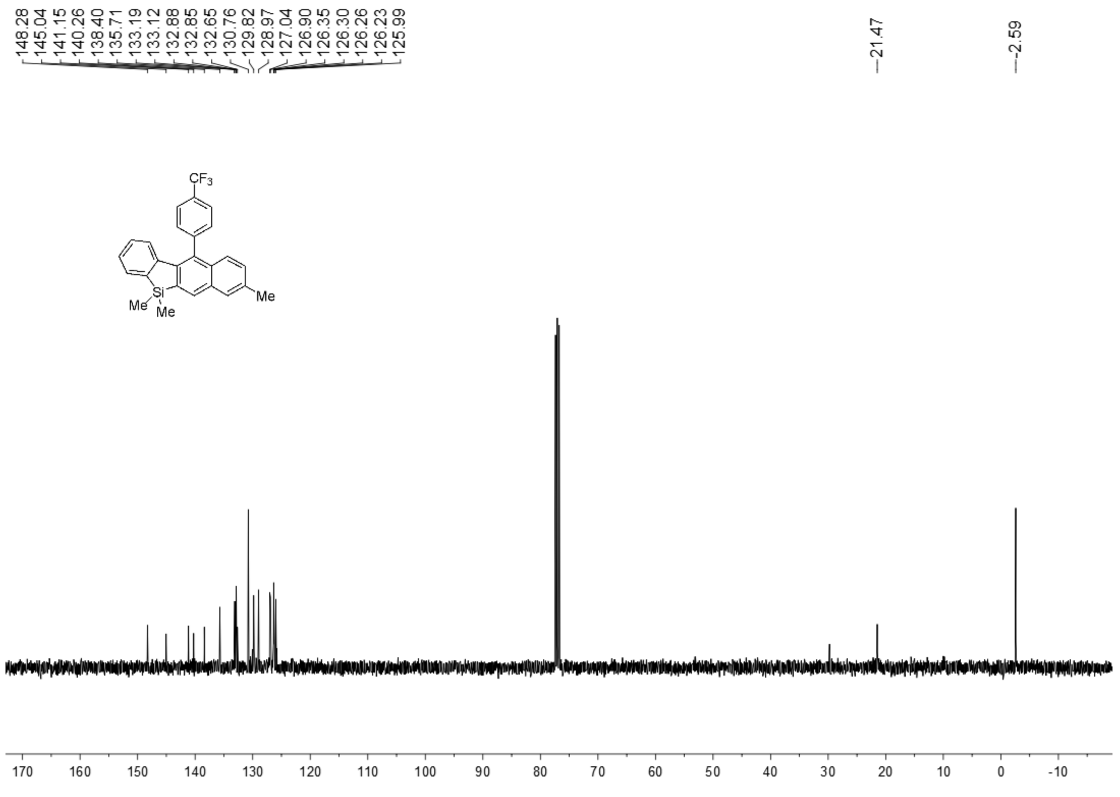

^{1}H NMR spectrum (400 MHz, $CDCl_3$) of **3k**

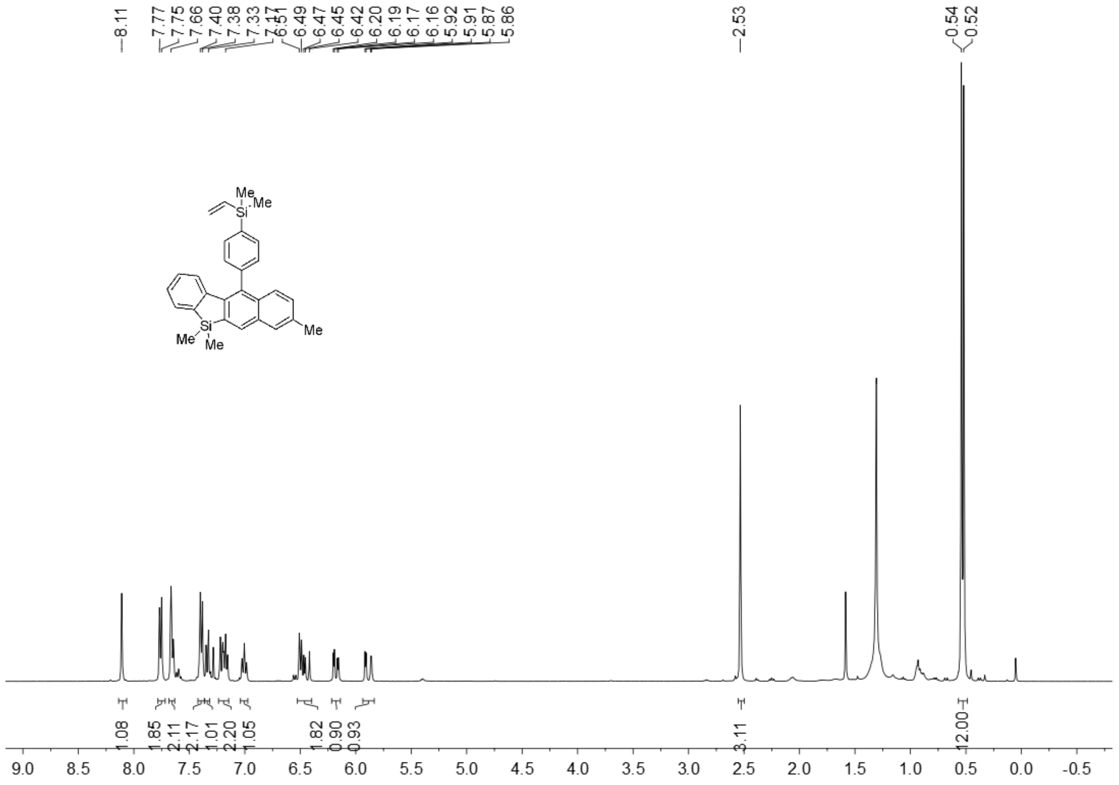

^{13}C NMR spectrum (101 MHz, $CDCl_3$) of **3k**

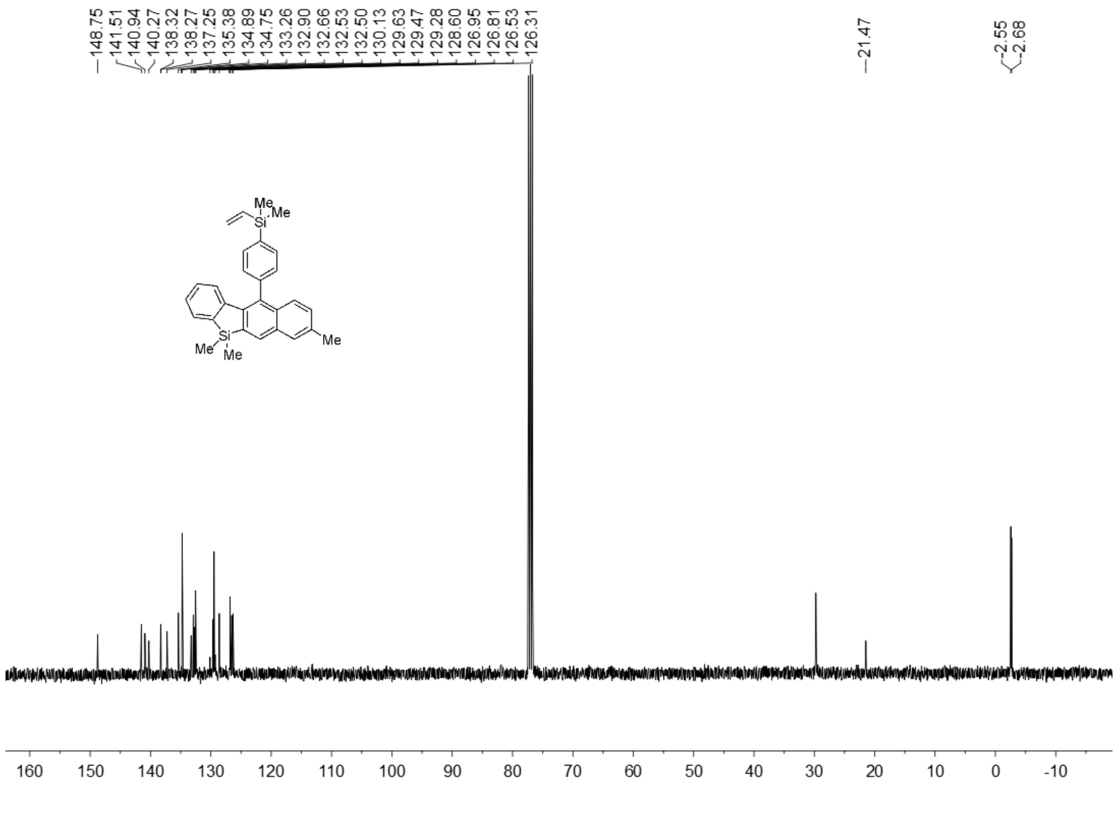

^{1}H NMR spectrum (500 MHz, $CDCl_3$) of **3l**

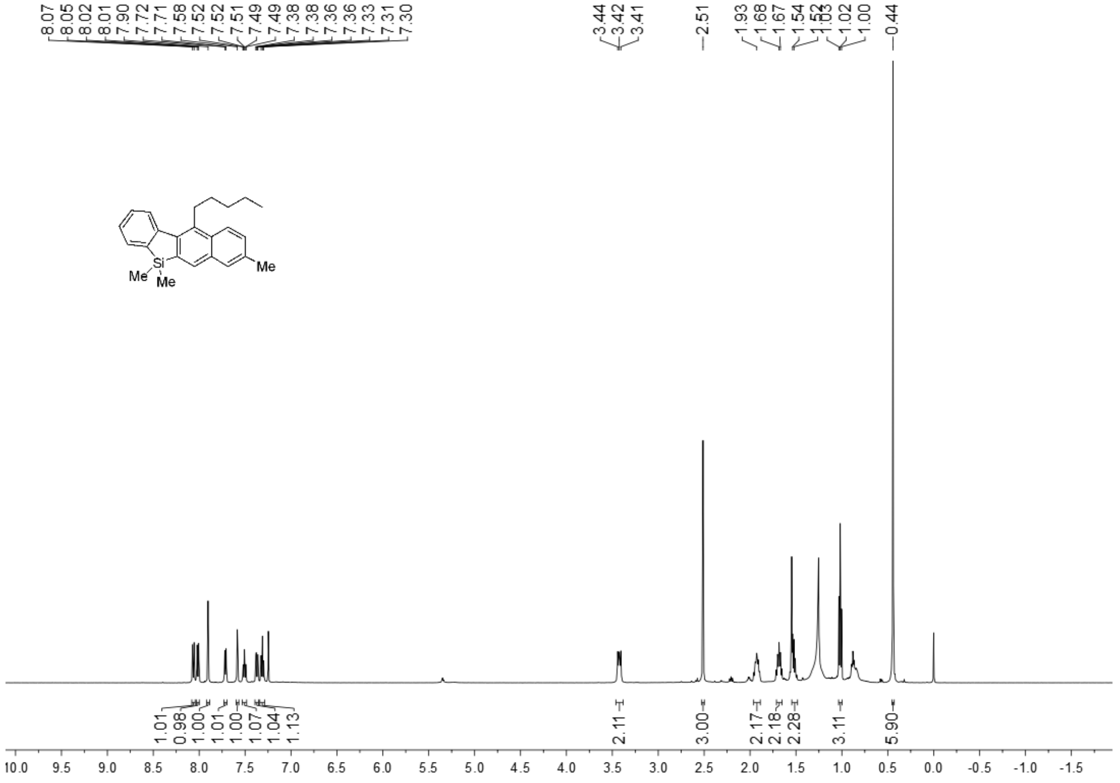

^{13}C NMR spectrum (126 MHz, $CDCl_3$) of **3l**

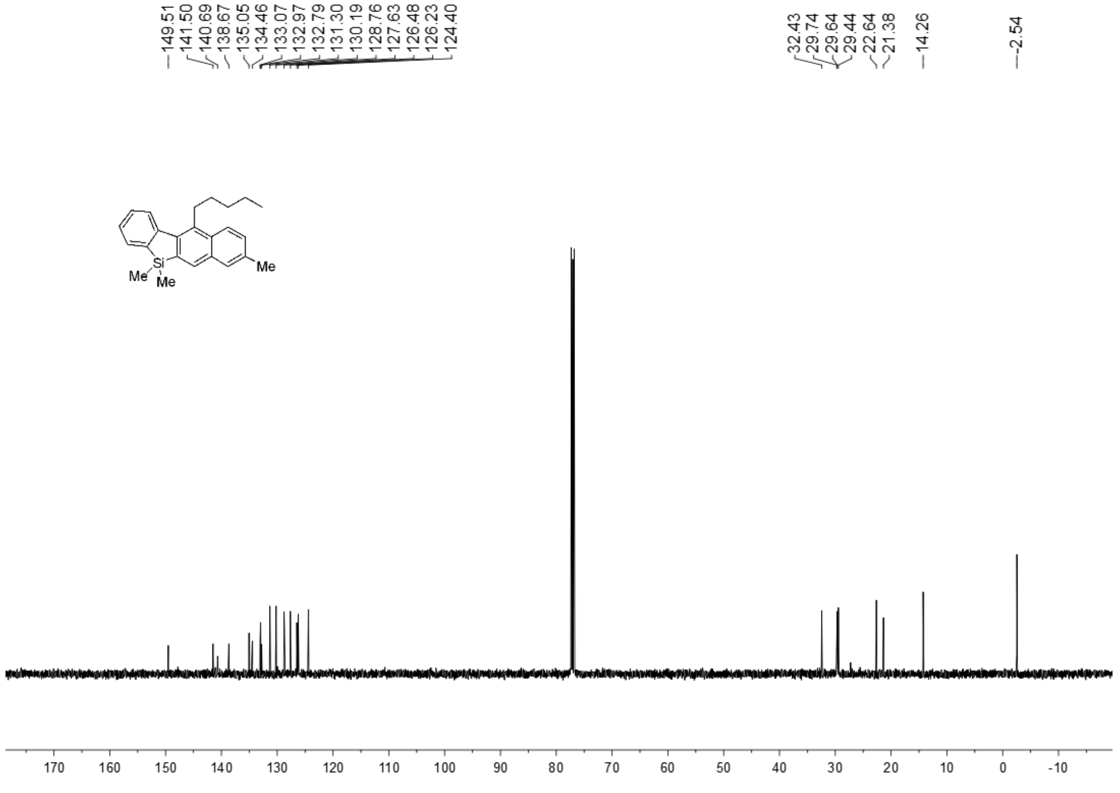

^{1}H NMR spectrum (400 MHz, $CDCl_3$) of **3m**

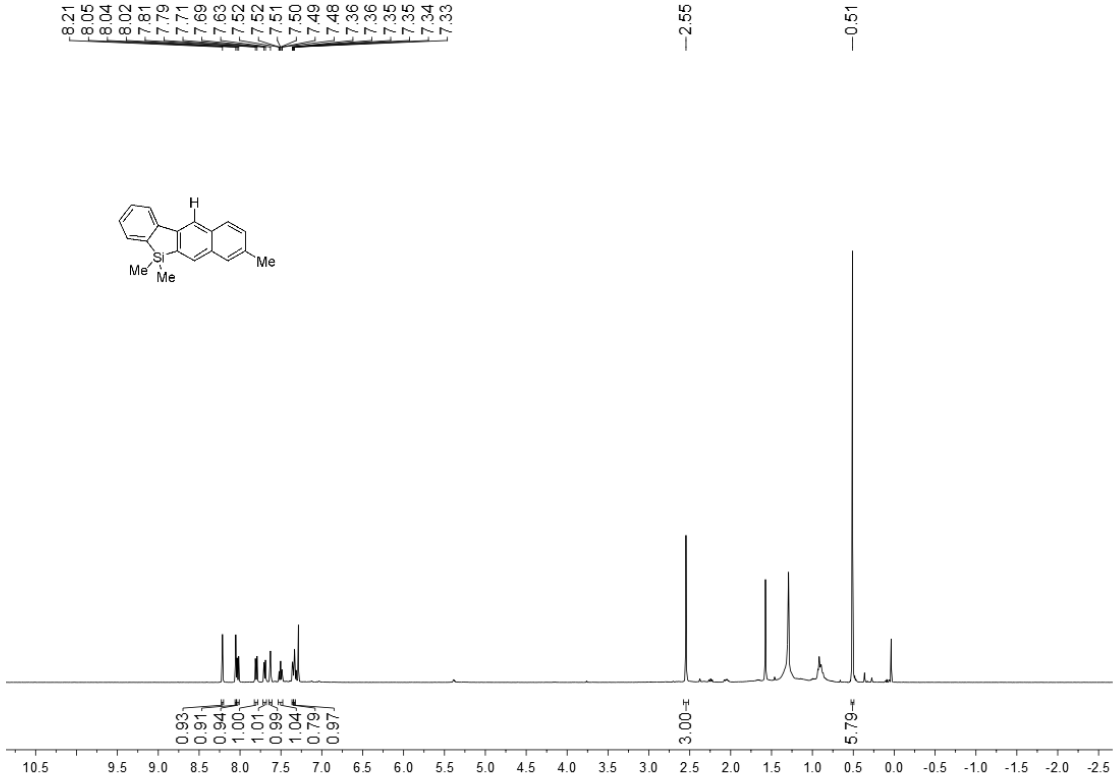

^{13}C NMR spectrum (126 MHz, $CDCl_3$) of **3m**

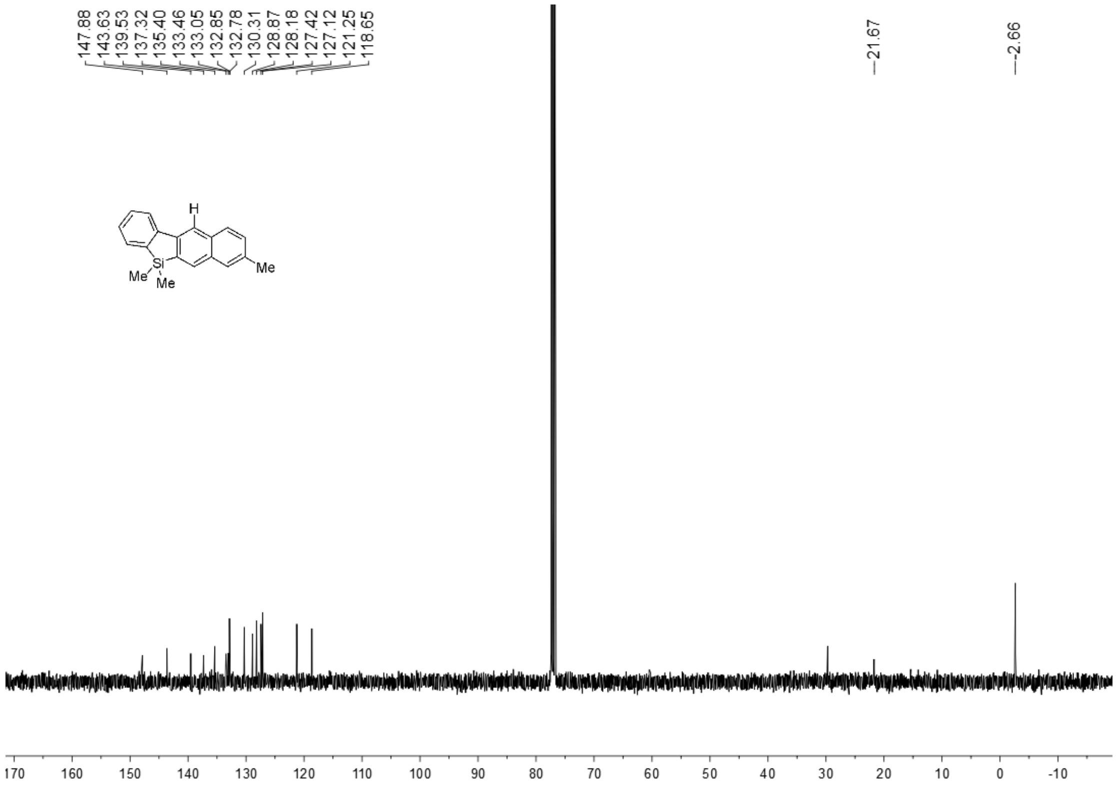

^{1}H NMR spectrum (400 MHz, $CDCl_3$) of **3n**

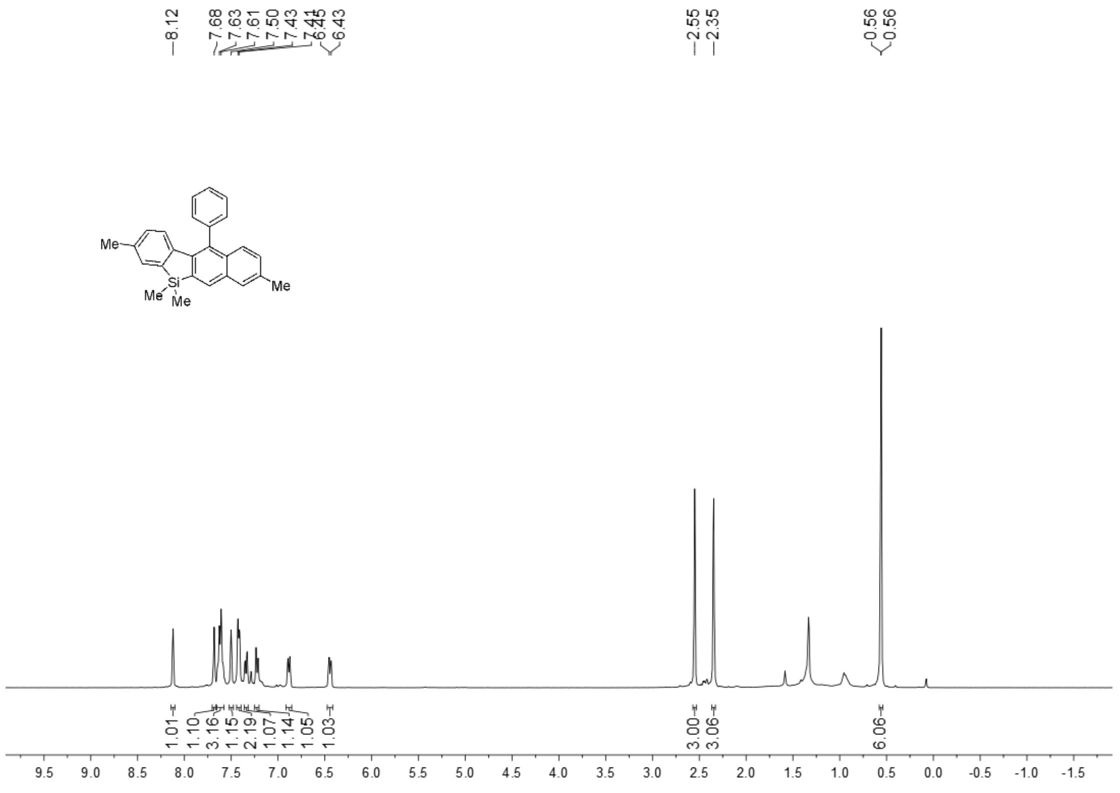

^{13}C NMR spectrum (101 MHz, $CDCl_3$) of **3n**

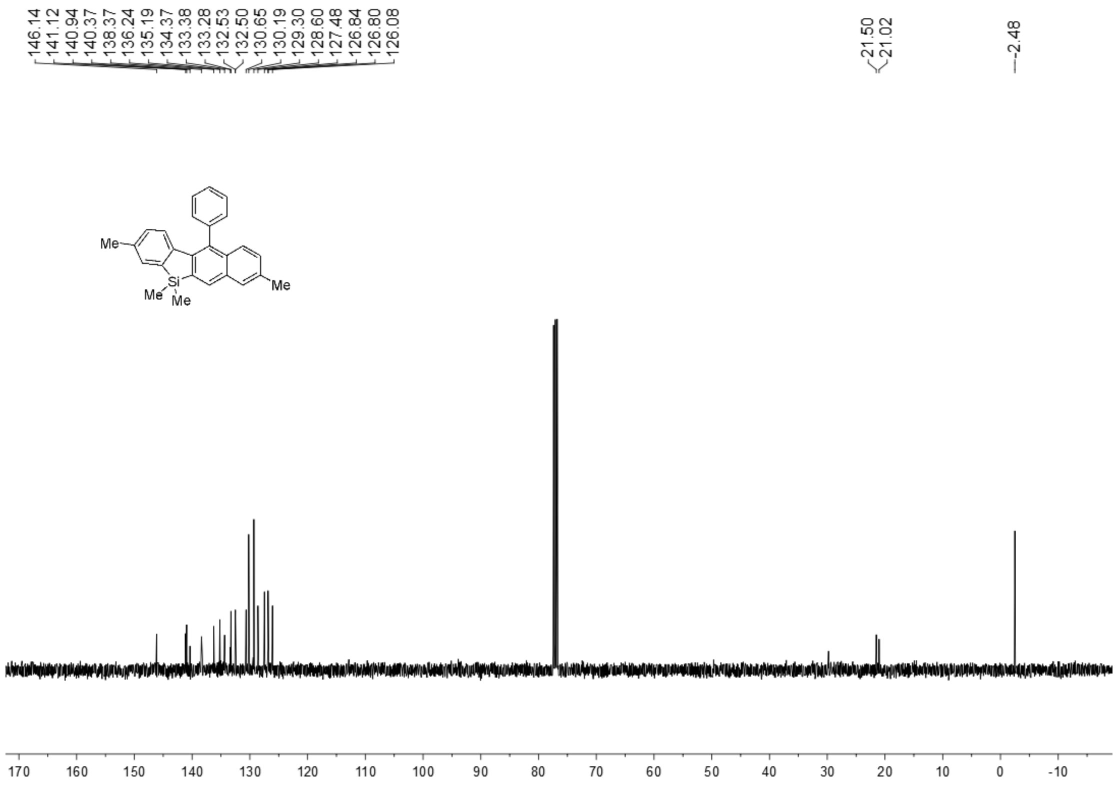

^{1}H NMR spectrum (400 MHz, $CDCl_3$) of **3o**

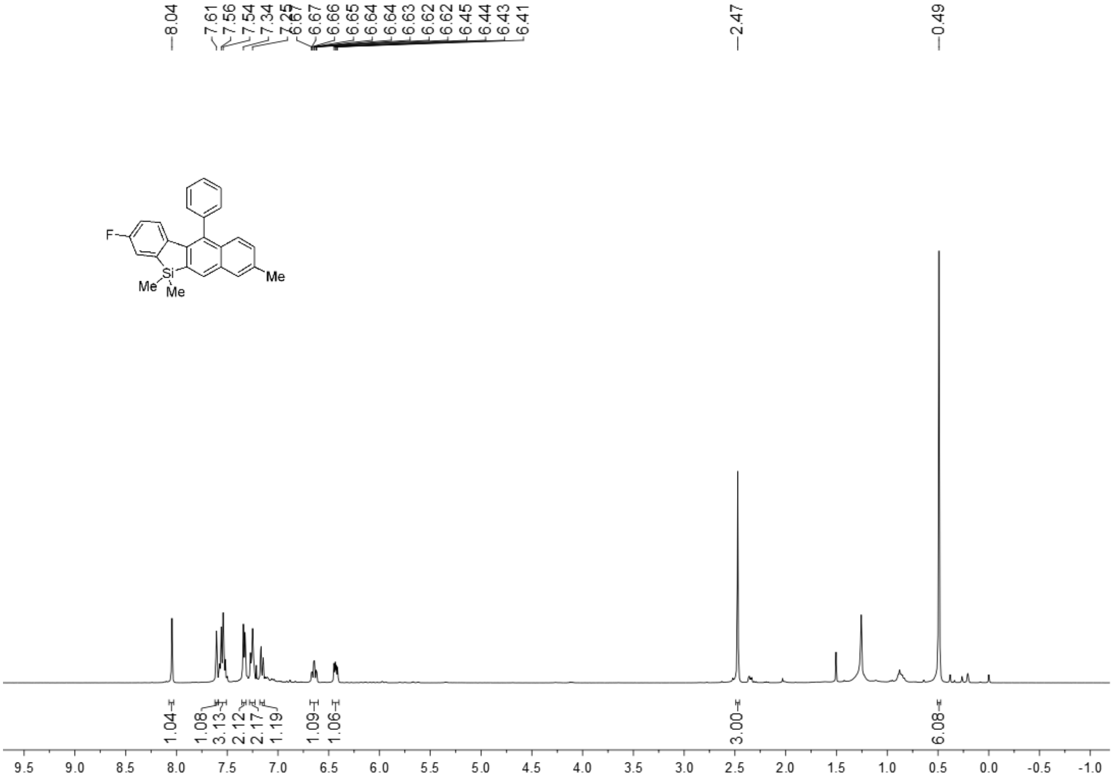

^{13}C NMR spectrum (101 MHz, $CDCl_3$) of **3o**

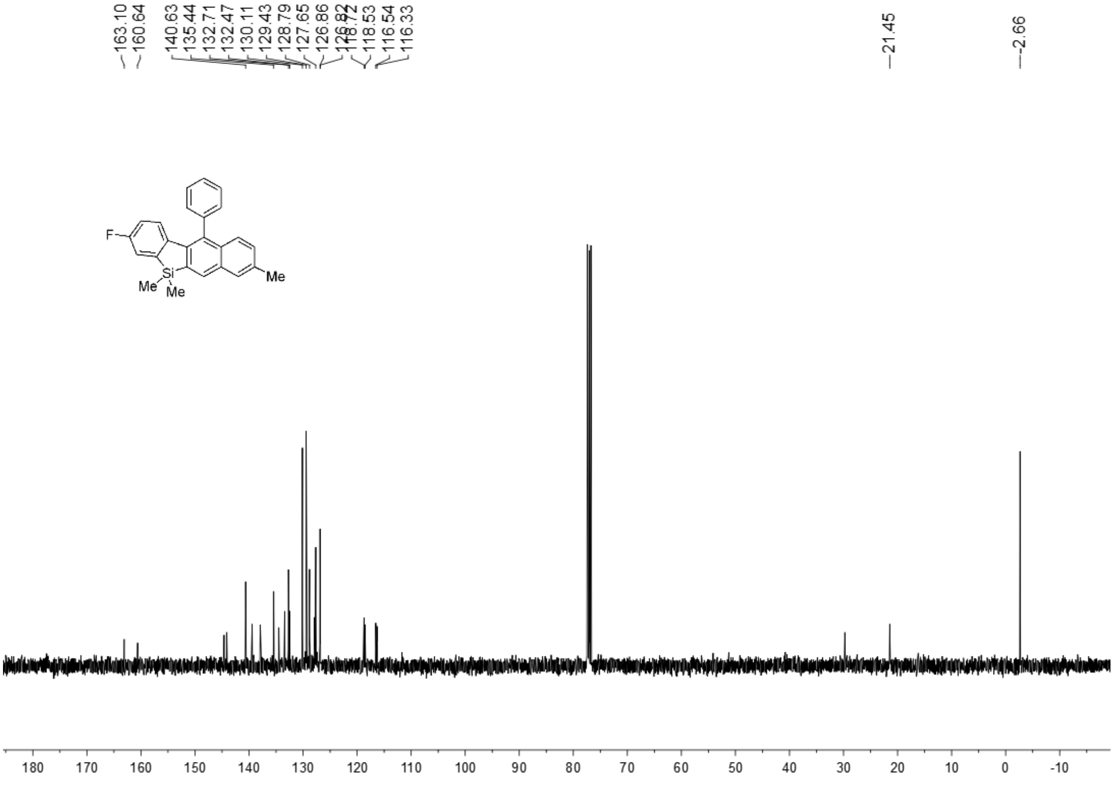

^{1}H NMR spectrum (400 MHz, $CDCl_3$) of **3p**

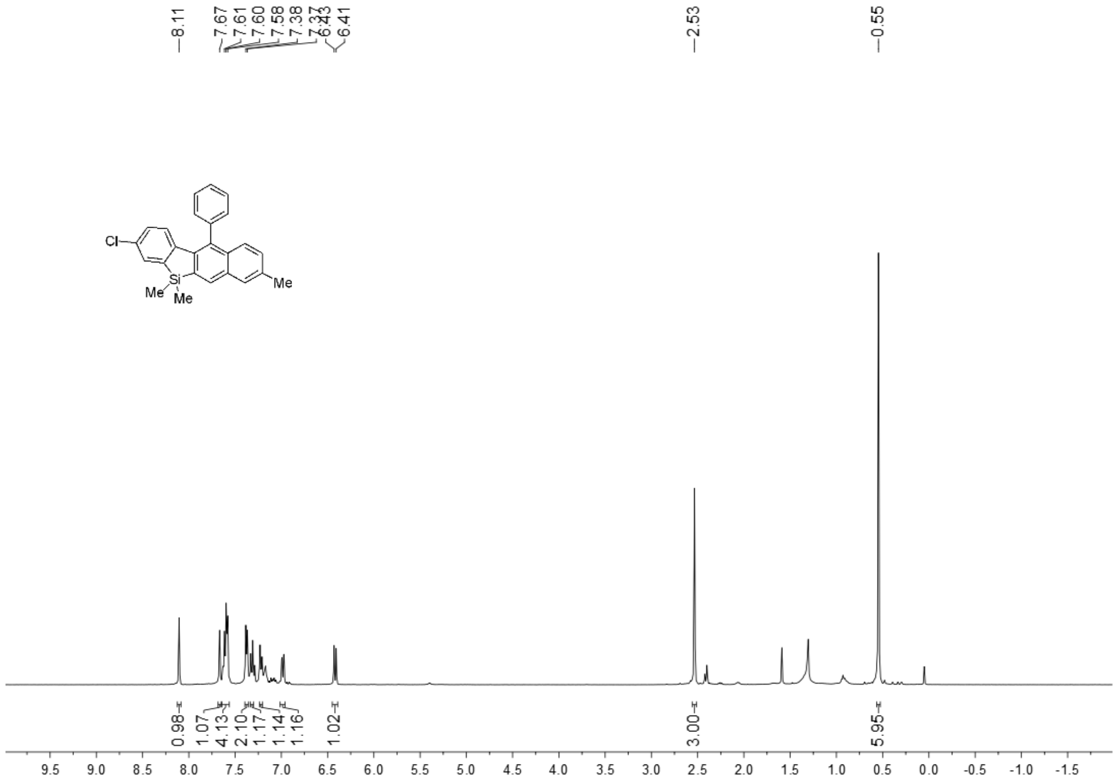

^{13}C NMR spectrum (101 MHz, $CDCl_3$) of **3p**

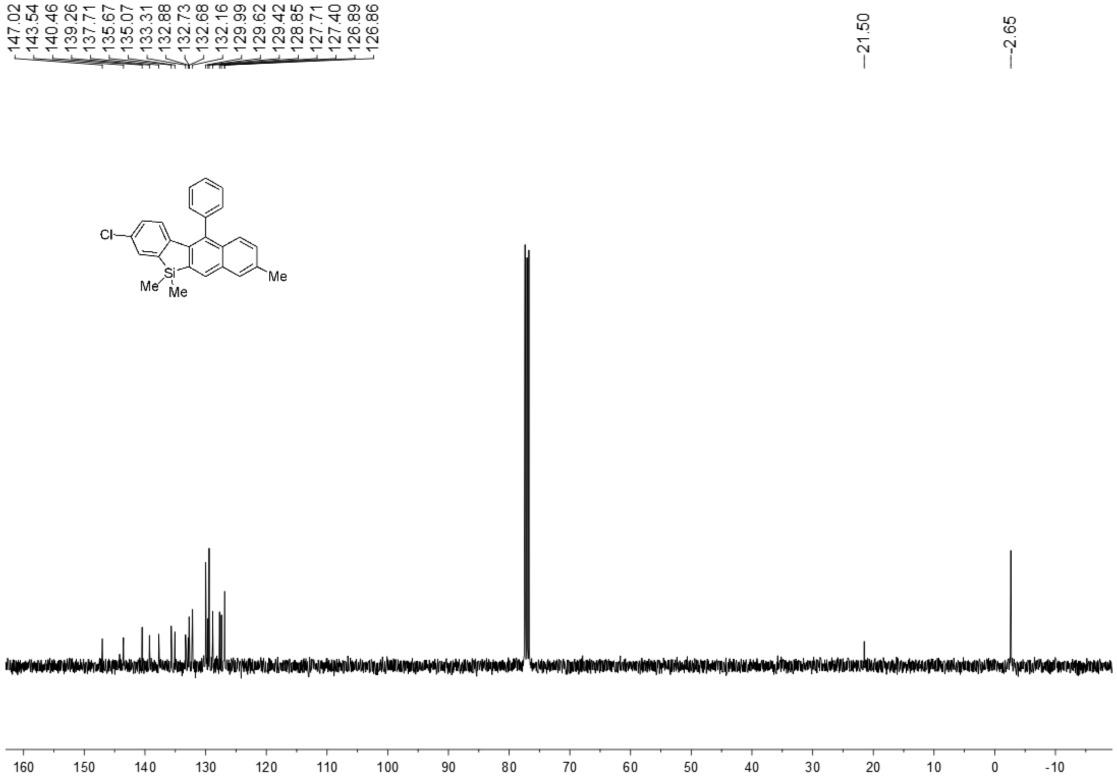

^{1}H NMR spectrum (400 MHz, $CDCl_3$) of **3q**

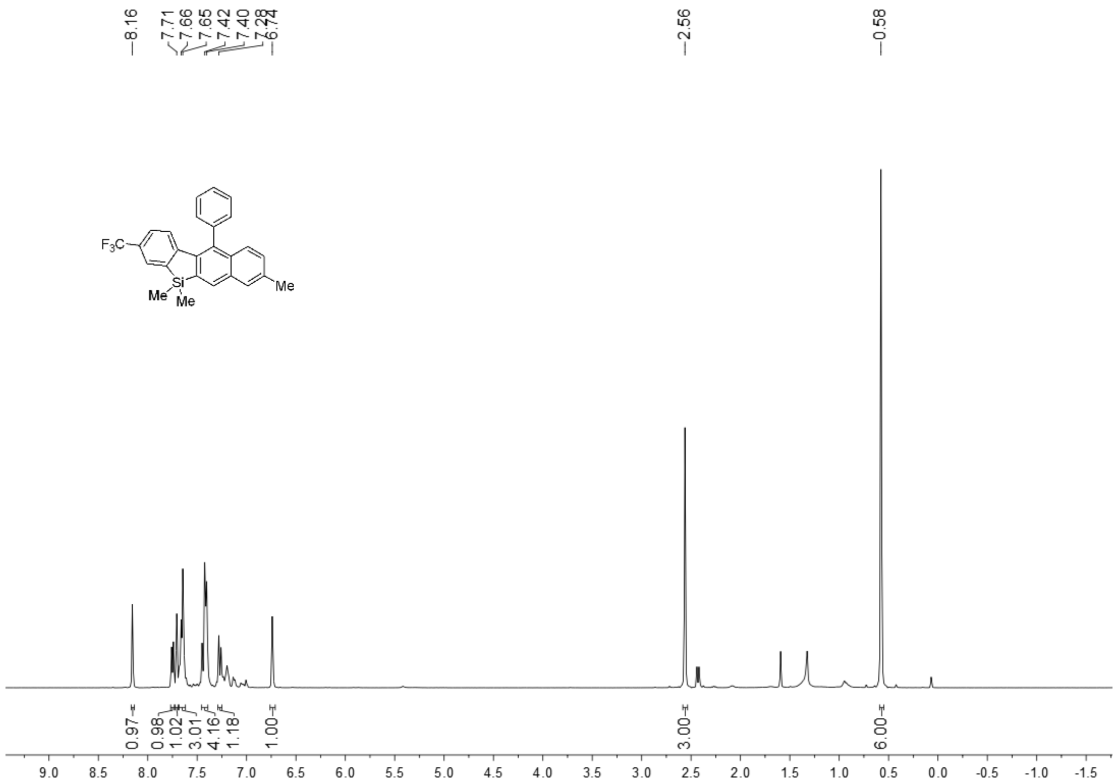

^{13}C NMR spectrum (101 MHz, $CDCl_3$) of **3q**

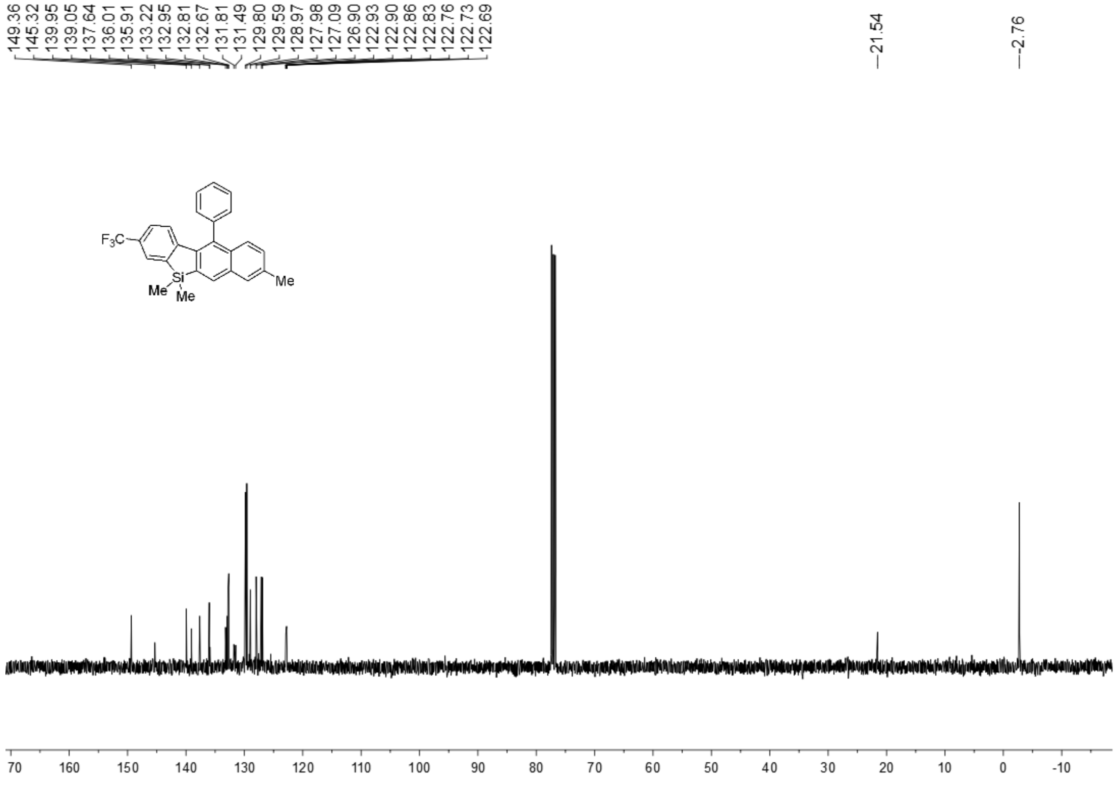

^{1}H NMR spectrum (400 MHz, $CDCl_3$) of **3r**

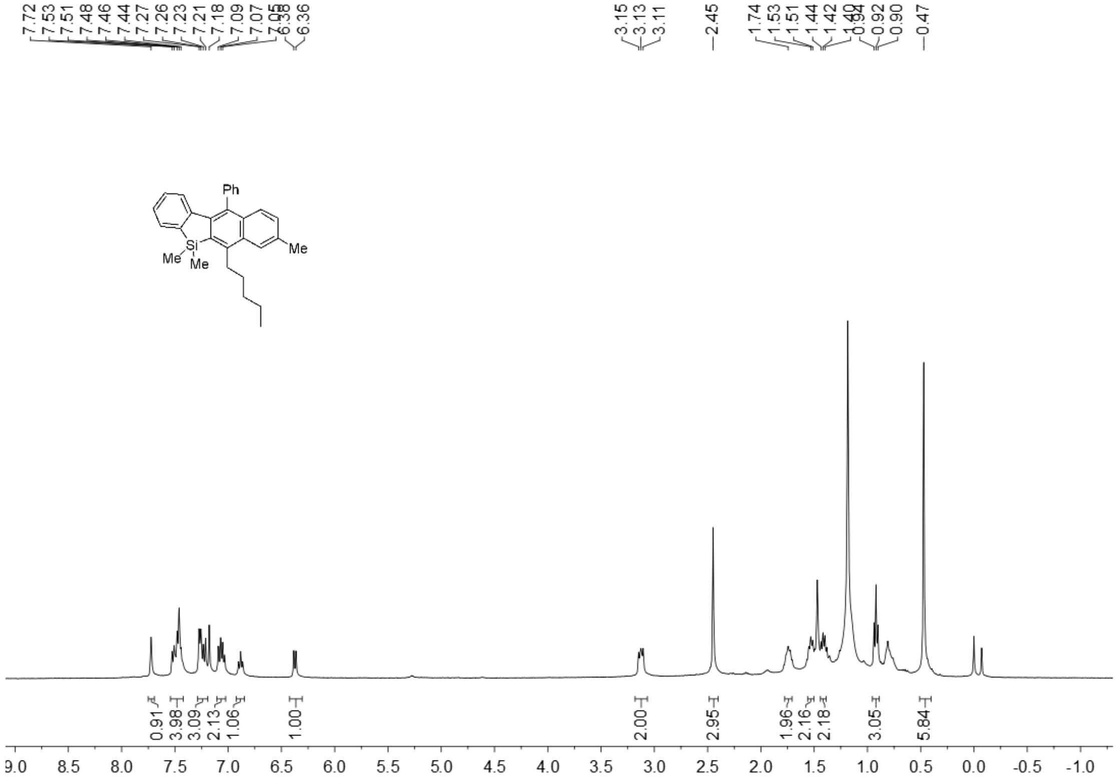

^{13}C NMR spectrum (101 MHz, $CDCl_3$) of **3r**

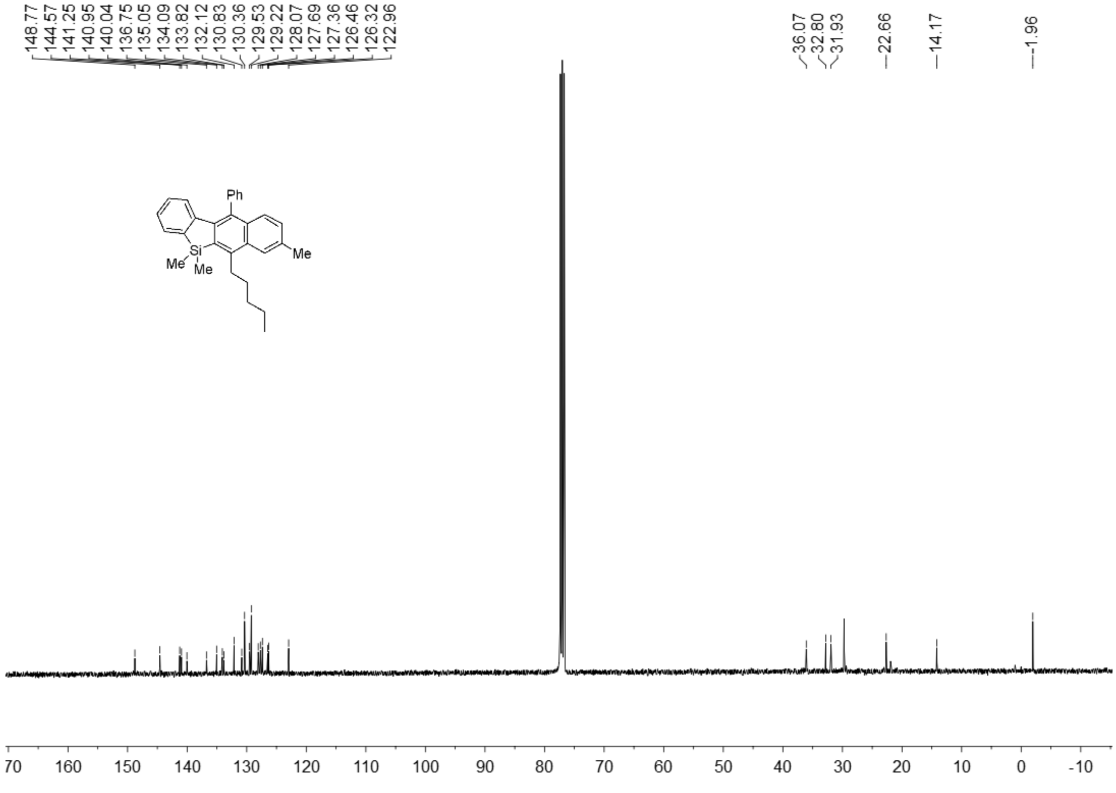

^{1}H NMR spectrum (500 MHz, $CDCl_3$) of **5a**

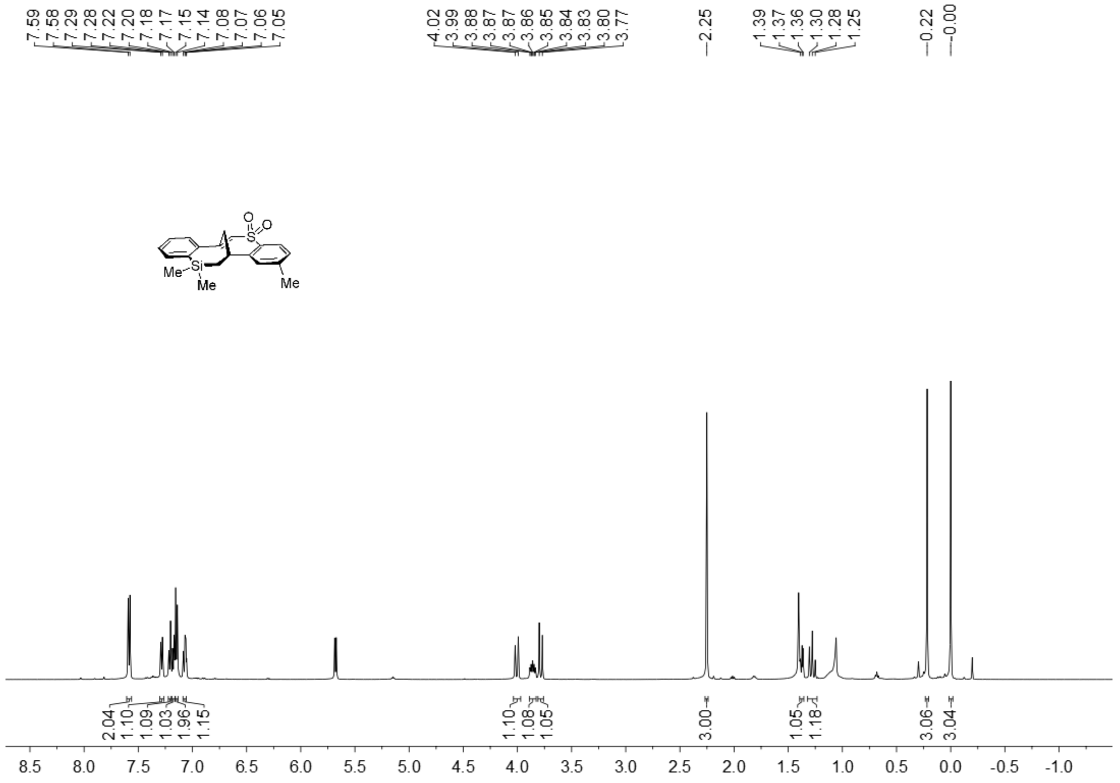

^{13}C NMR spectrum (126 MHz, $CDCl_3$) of **5a**

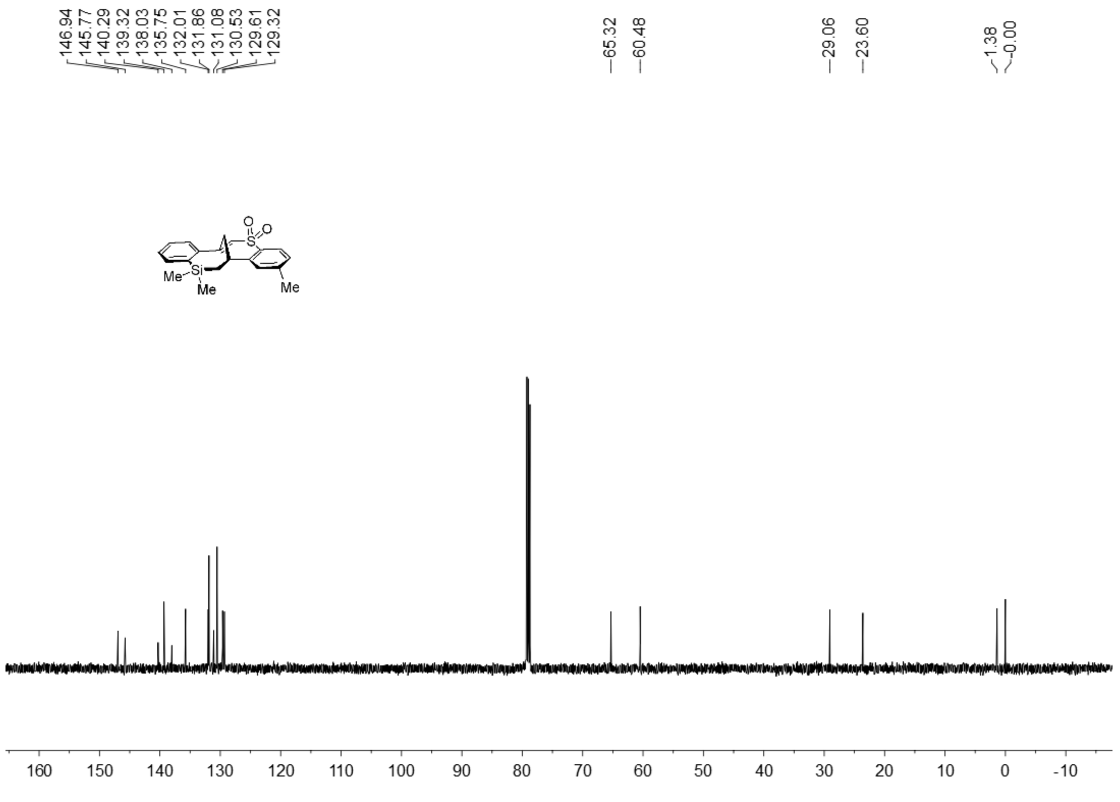

^{1}H NMR spectrum (400 MHz, $CDCl_3$) of **5b**

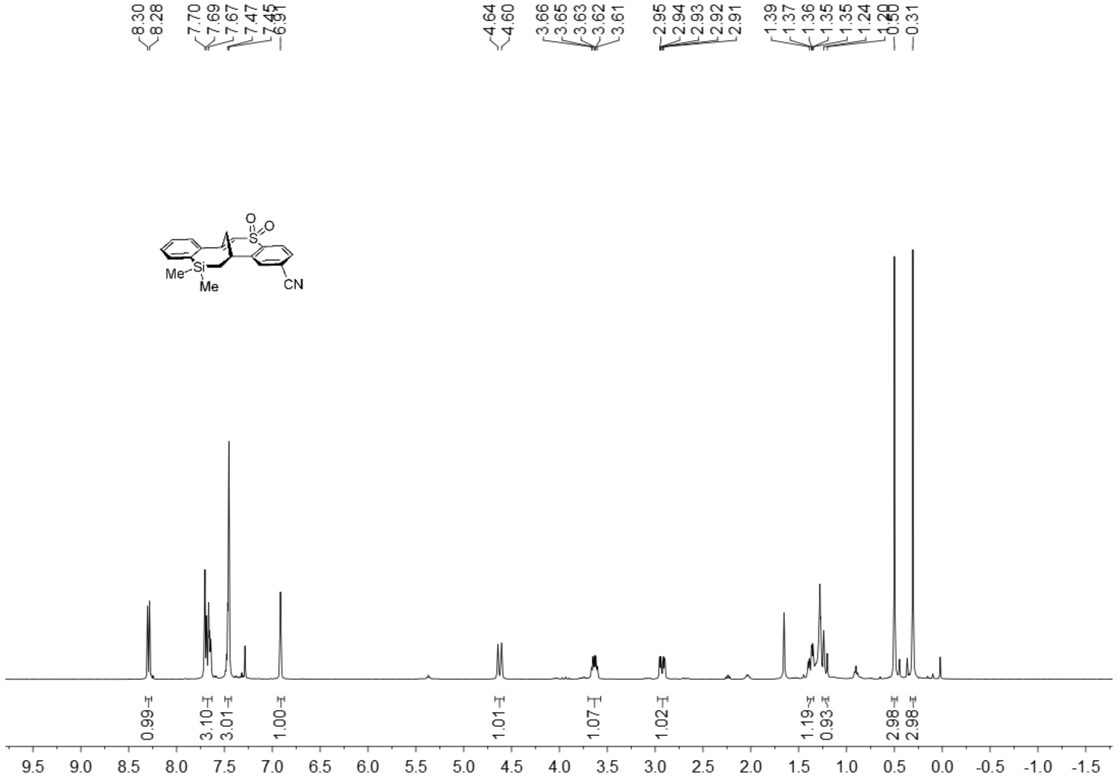

^{13}C NMR spectrum (101 MHz, $CDCl_3$) of **5b**

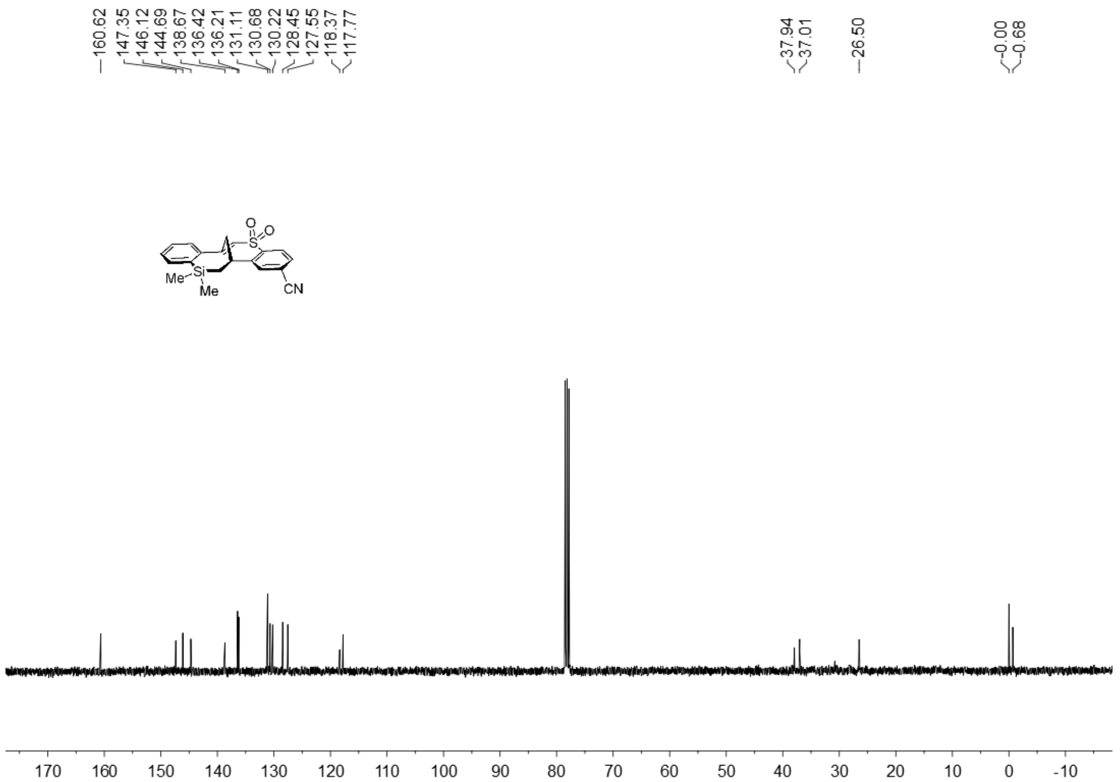

^{1}H NMR spectrum (400 MHz, $CDCl_3$) of **5c**

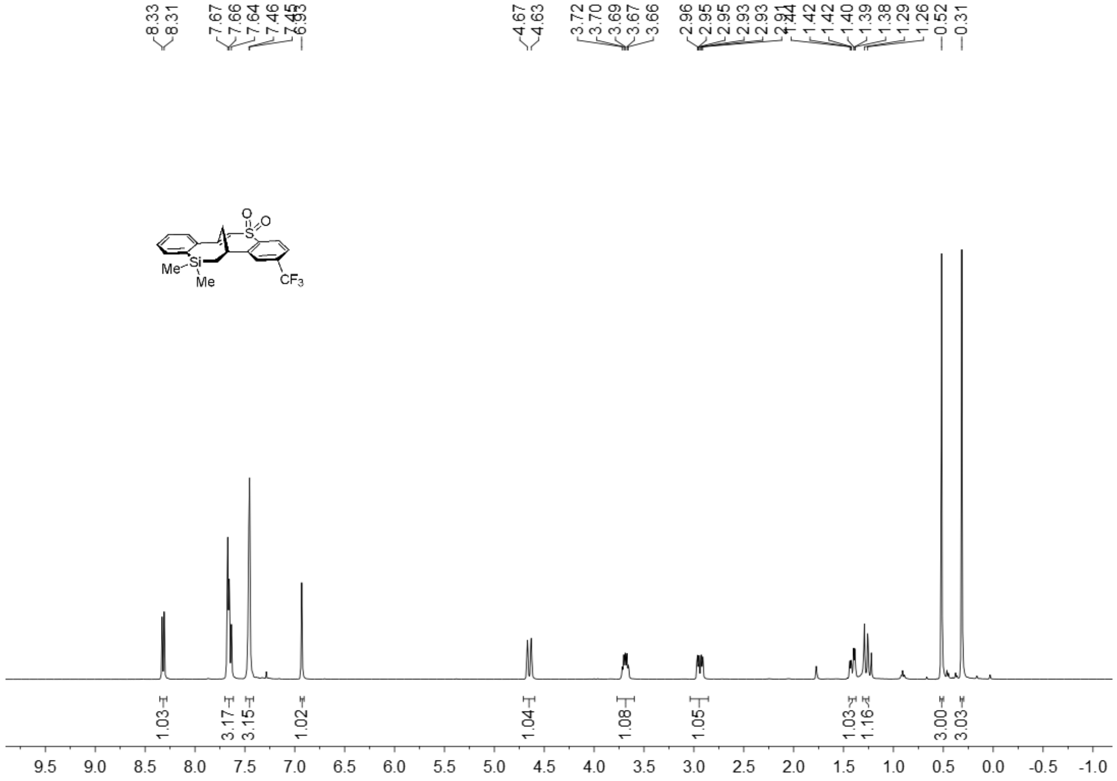

^{13}C NMR spectrum (101 MHz, $CDCl_3$) of **5c**

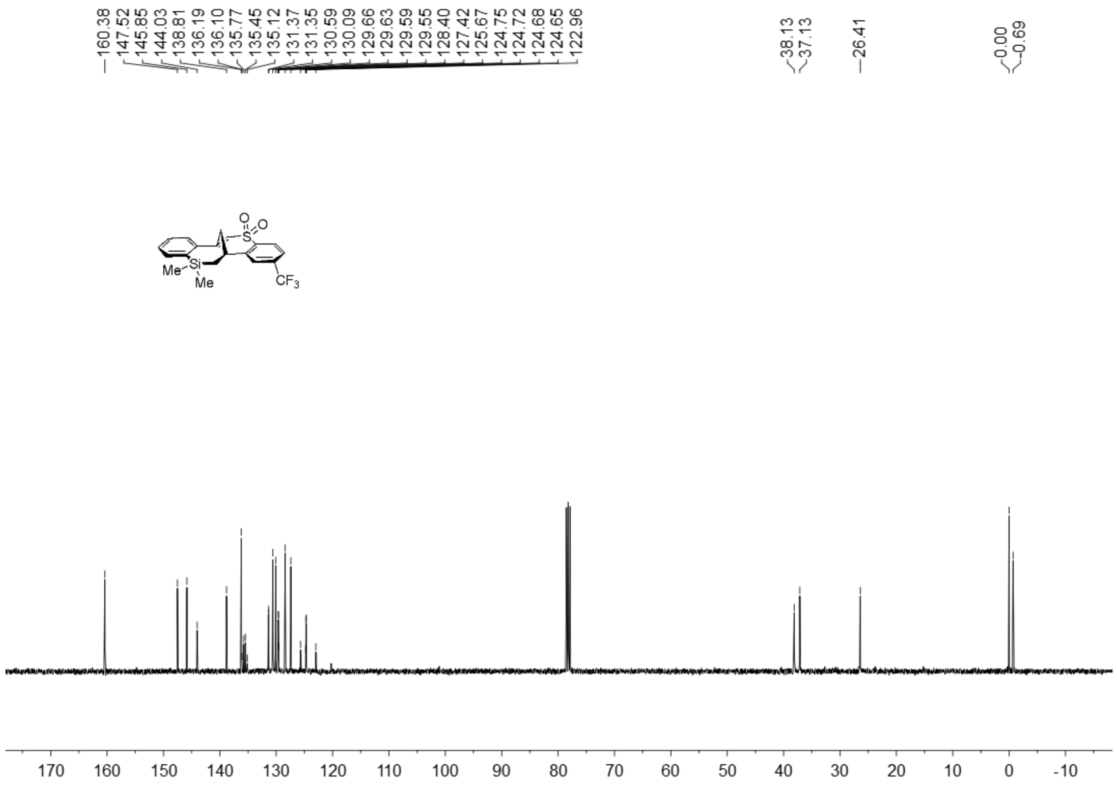

^{1}H NMR spectrum (500 MHz, $CDCl_3$) of **6a**

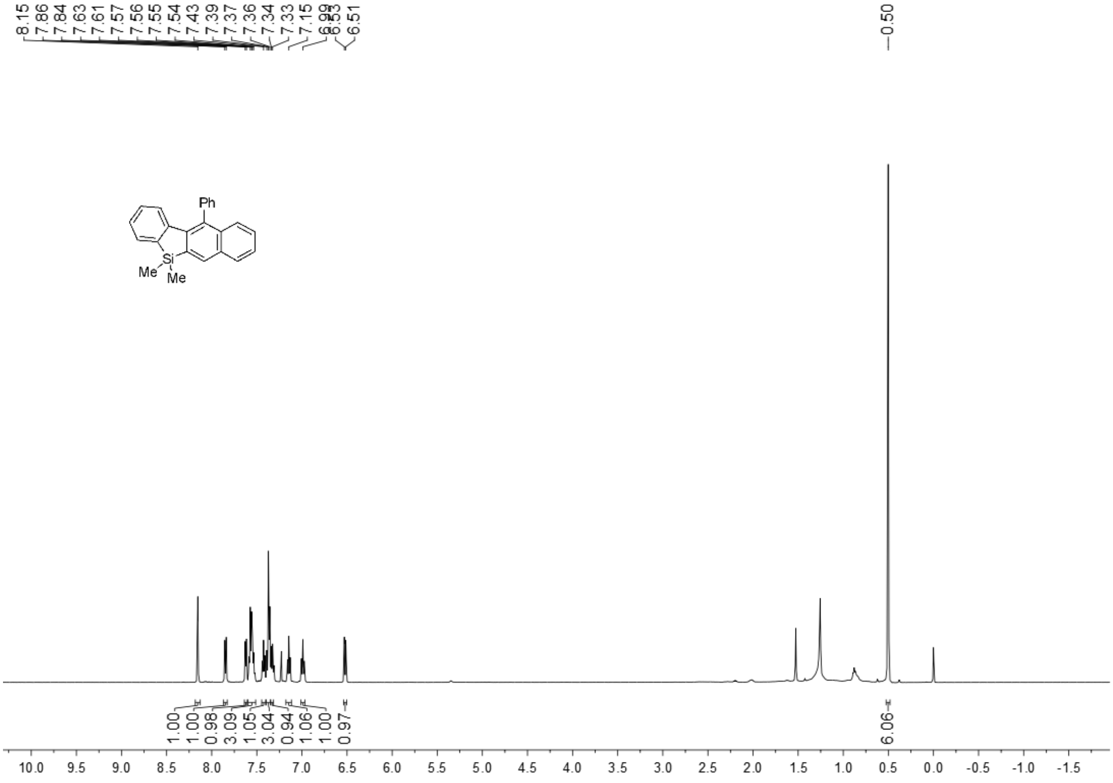

^{13}C NMR spectrum (126 MHz, $CDCl_3$) of **6a**

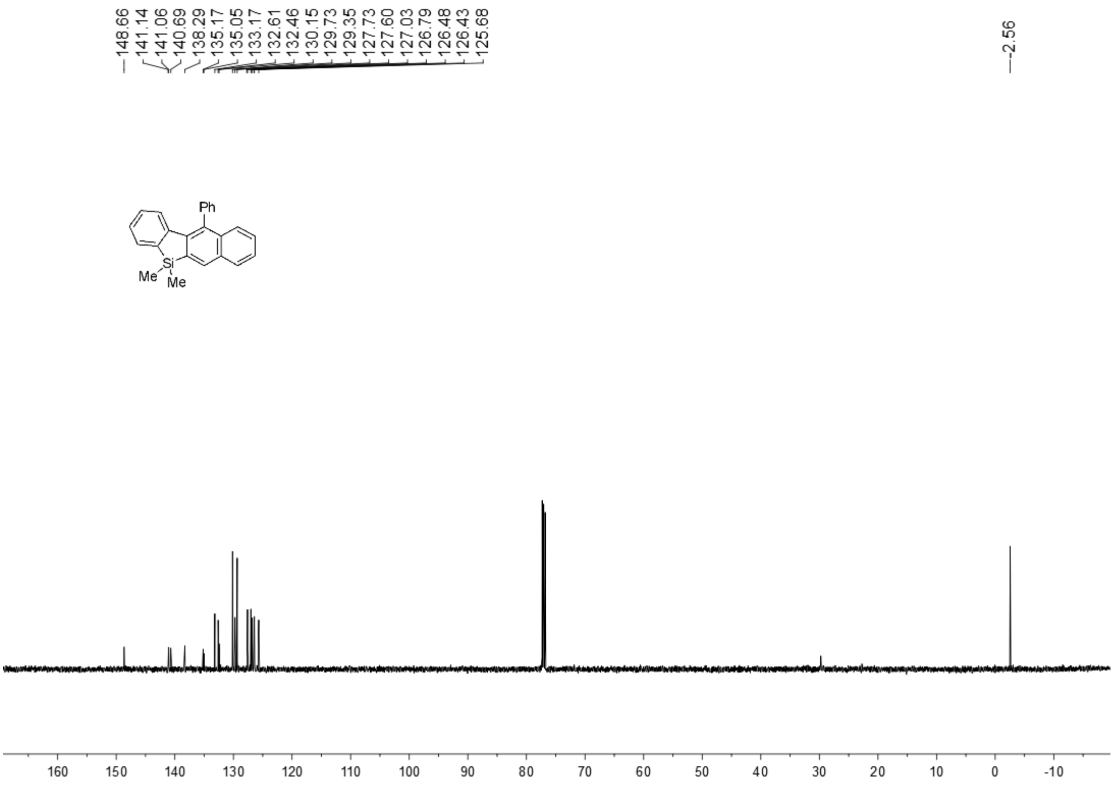

^{1}H NMR spectrum (400 MHz, $CDCl_3$) of **6b** and **6c**

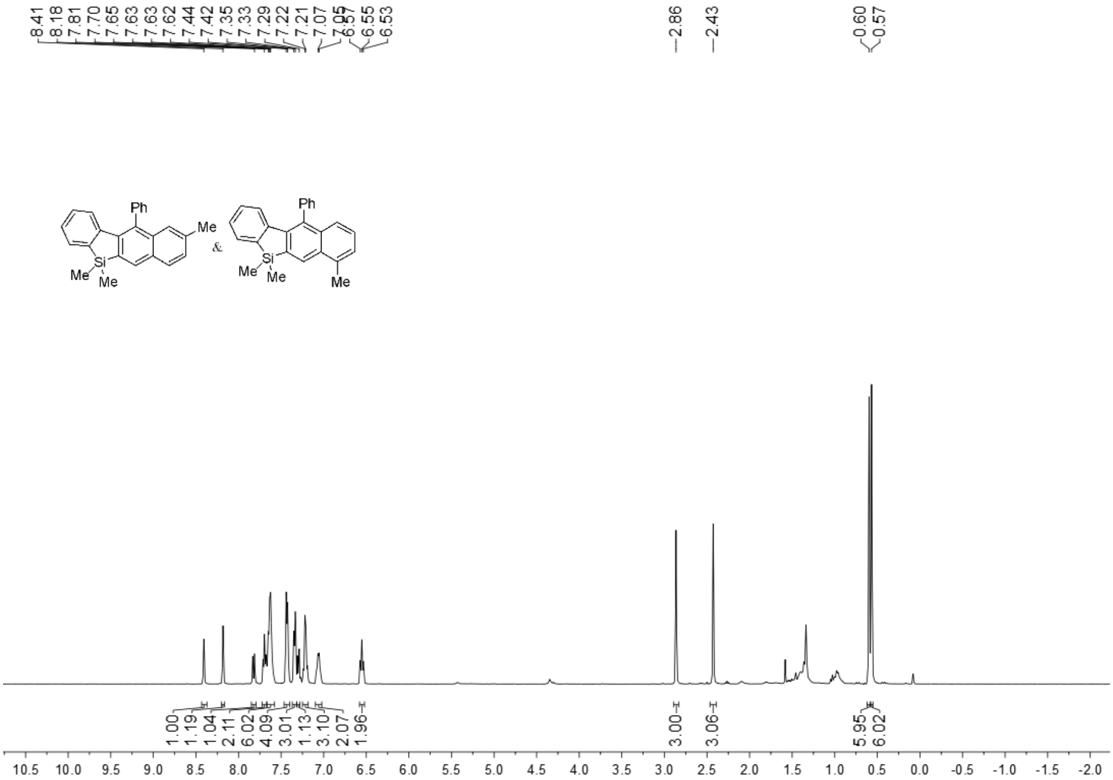

^{13}C NMR spectrum (101 MHz, $CDCl_3$) of **6b** and **6c**

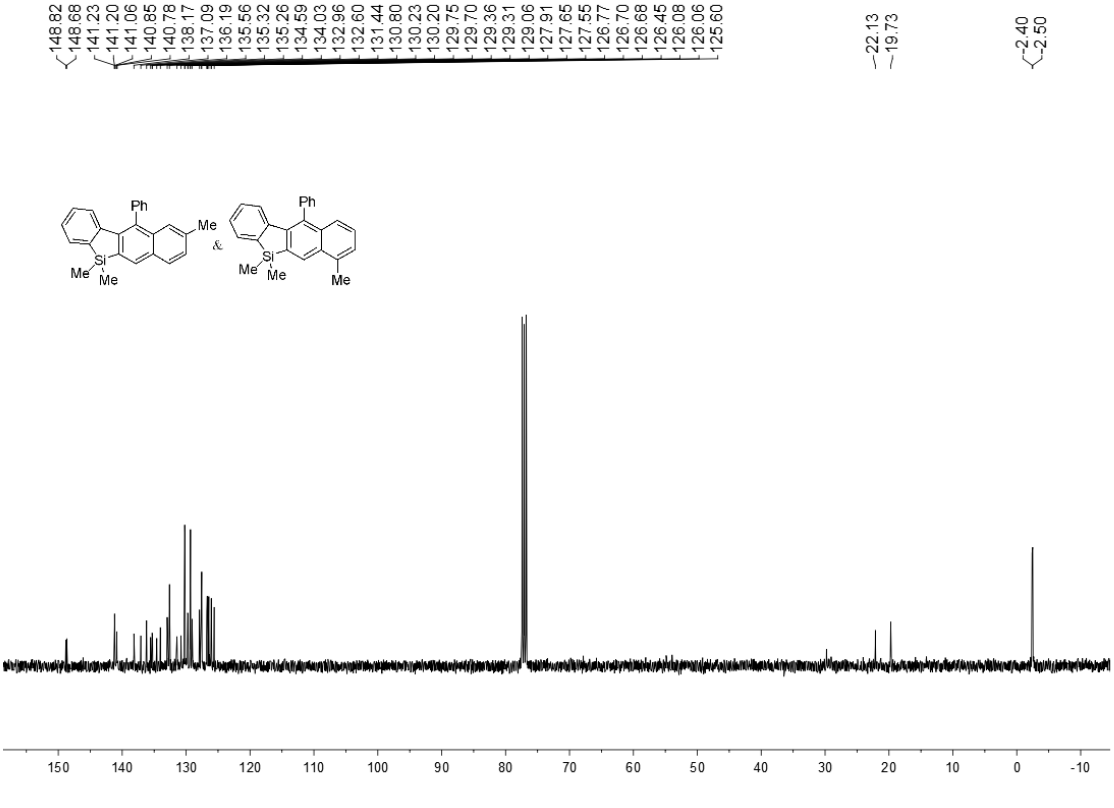

^{1}H NMR spectrum (500 MHz, $CDCl_3$) of **6d**

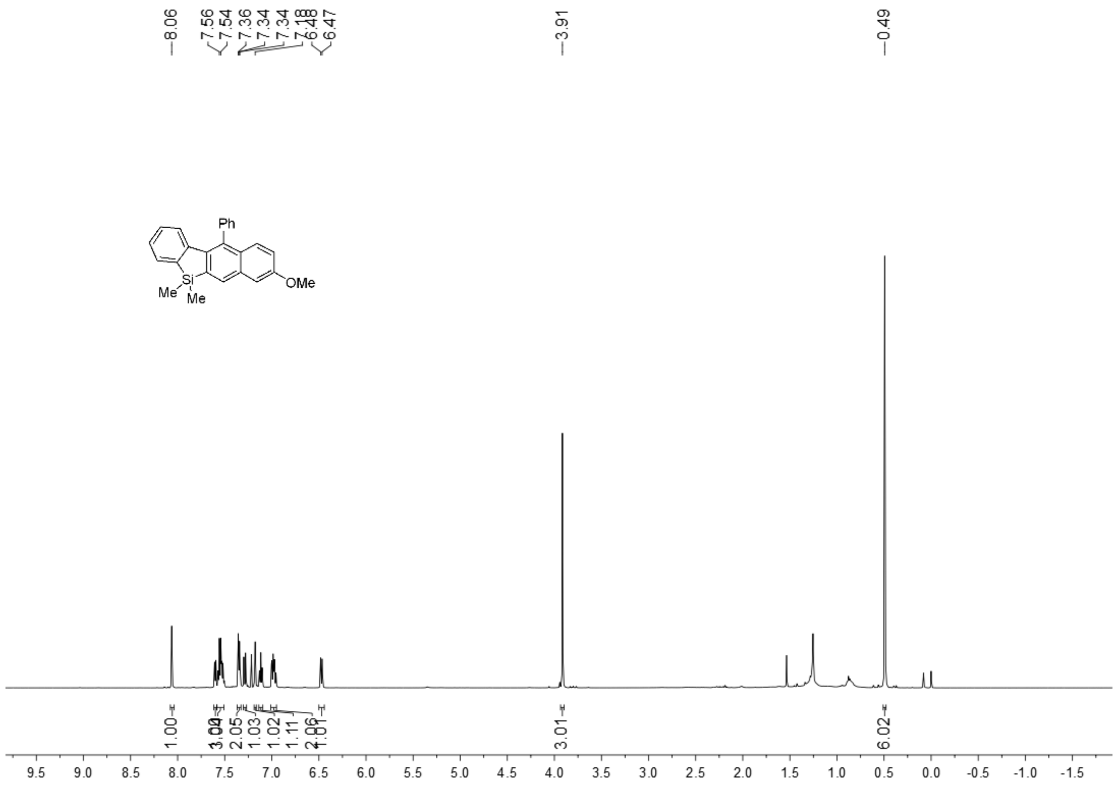

^{13}C NMR spectrum (126 MHz, $CDCl_3$) of **6d**

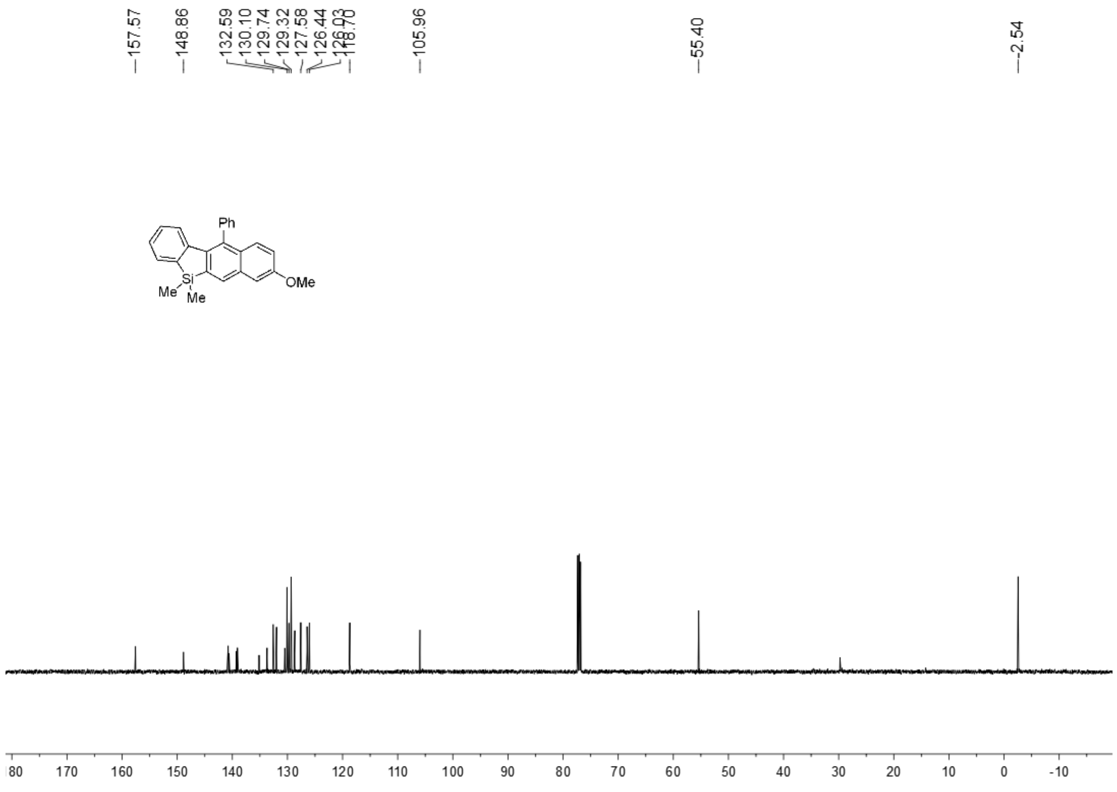

^{1}H NMR spectrum (500 MHz, $CDCl_3$) of **6e**

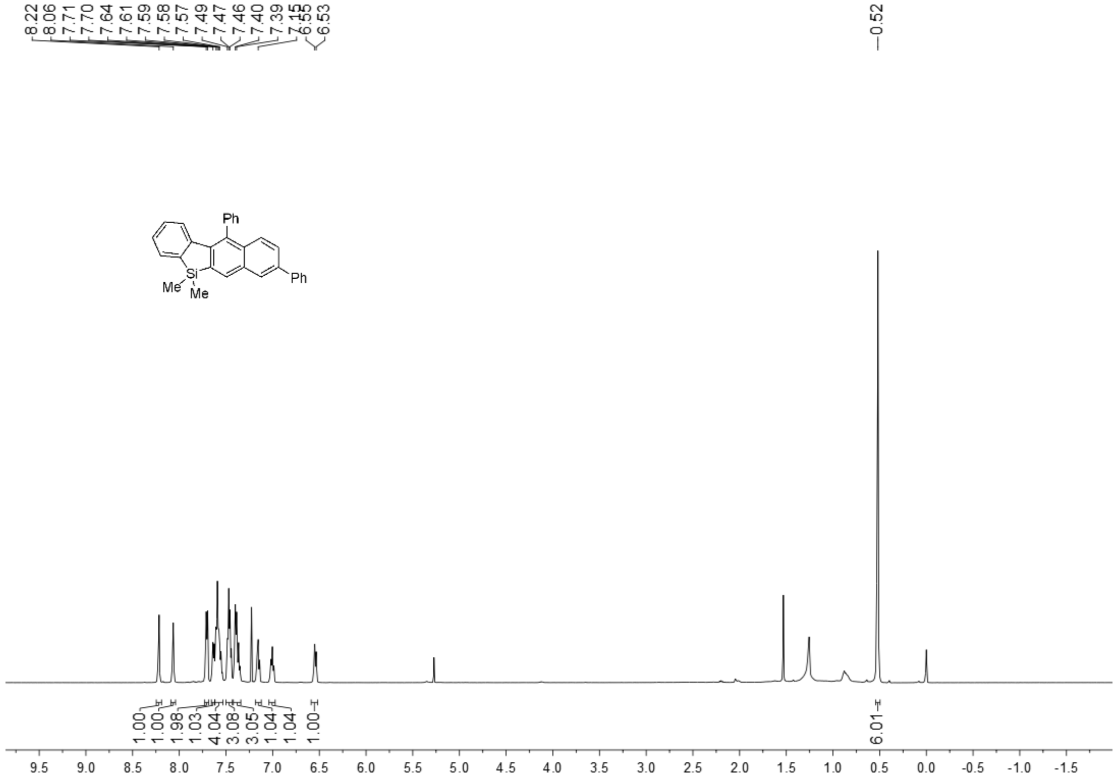

^{13}C NMR spectrum (126 MHz, $CDCl_3$) of **6e**

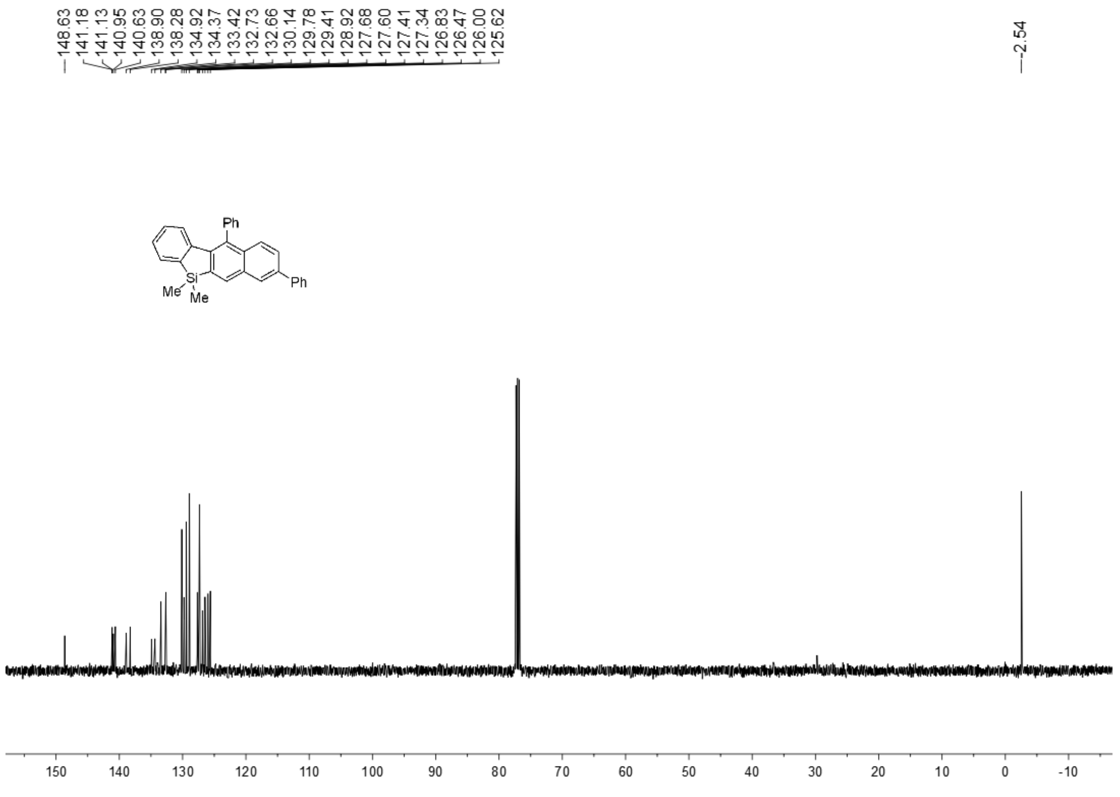

^{1}H NMR spectrum (500 MHz, $CDCl_3$) of **6f** and **6g**

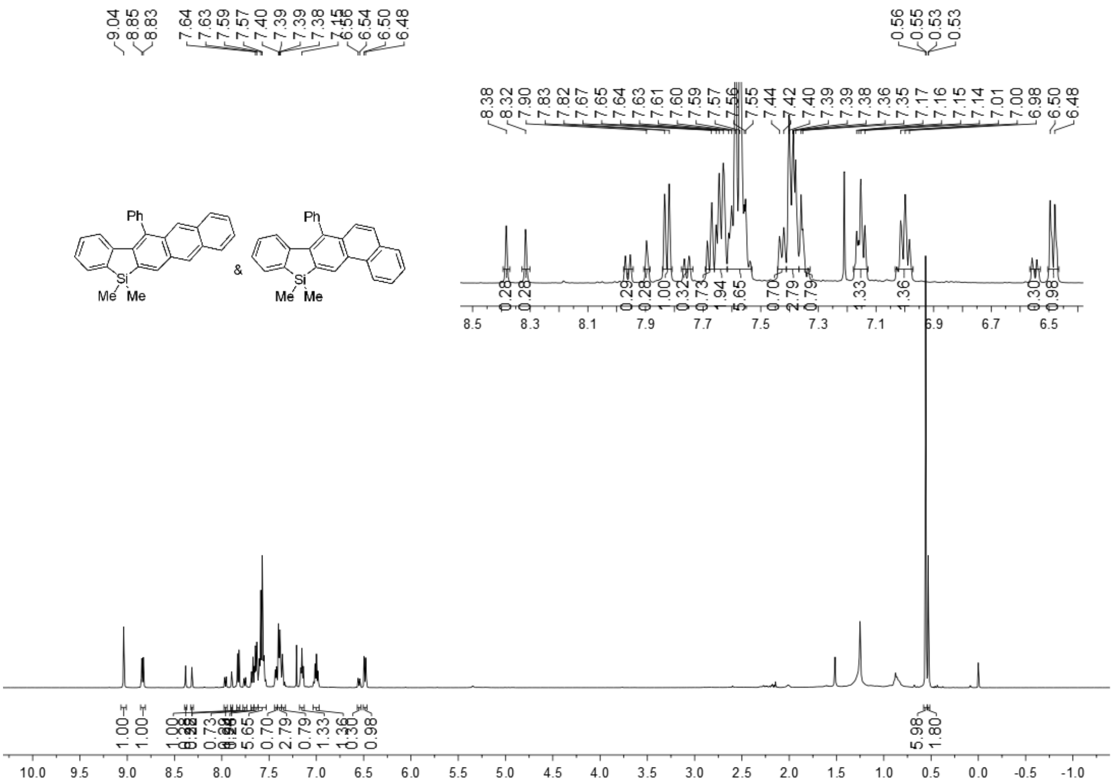

^{13}C NMR spectrum (126 MHz, $CDCl_3$) of **6f** and **6g**

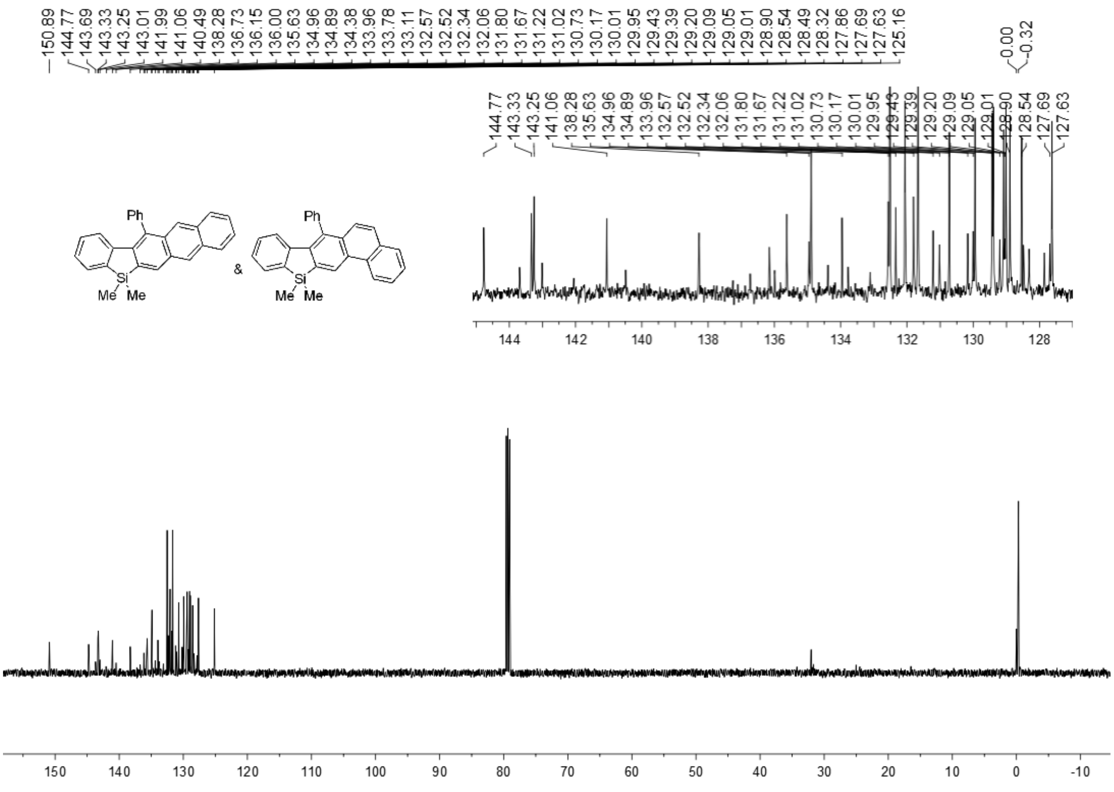

^{1}H NMR spectrum (400 MHz, $CDCl_3$) of **6h**

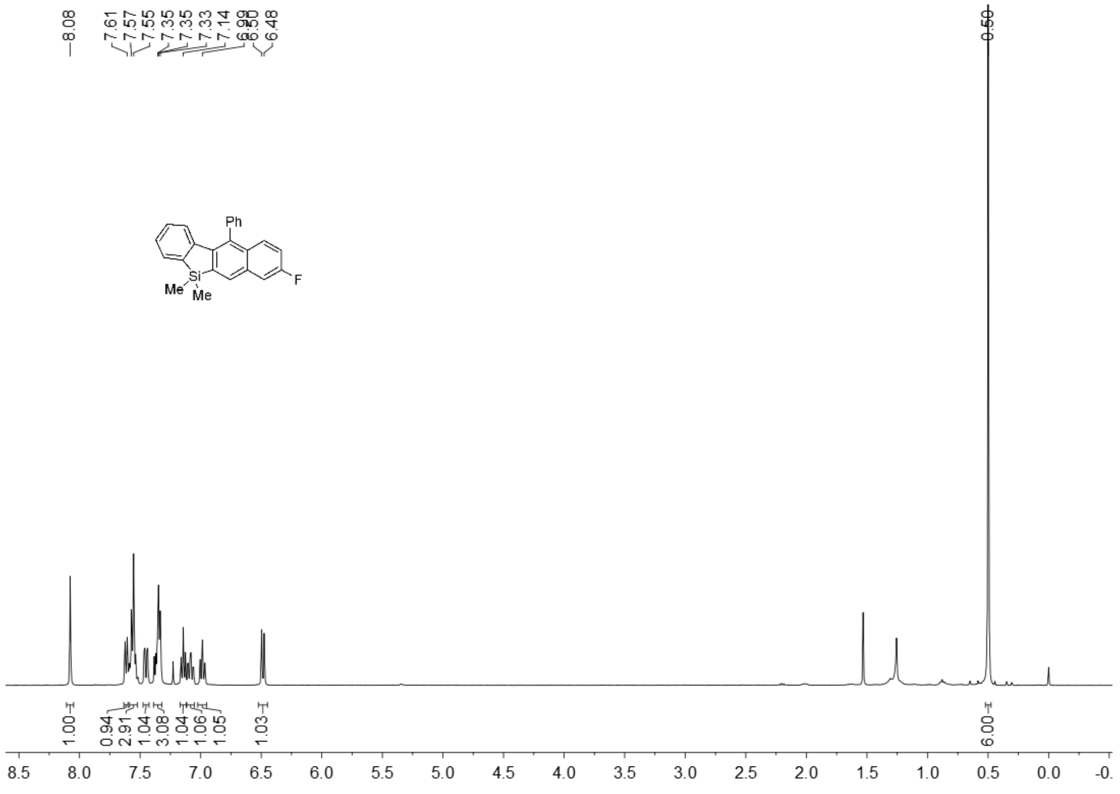

^{13}C NMR spectrum (101 MHz, $CDCl_3$) of **6h**

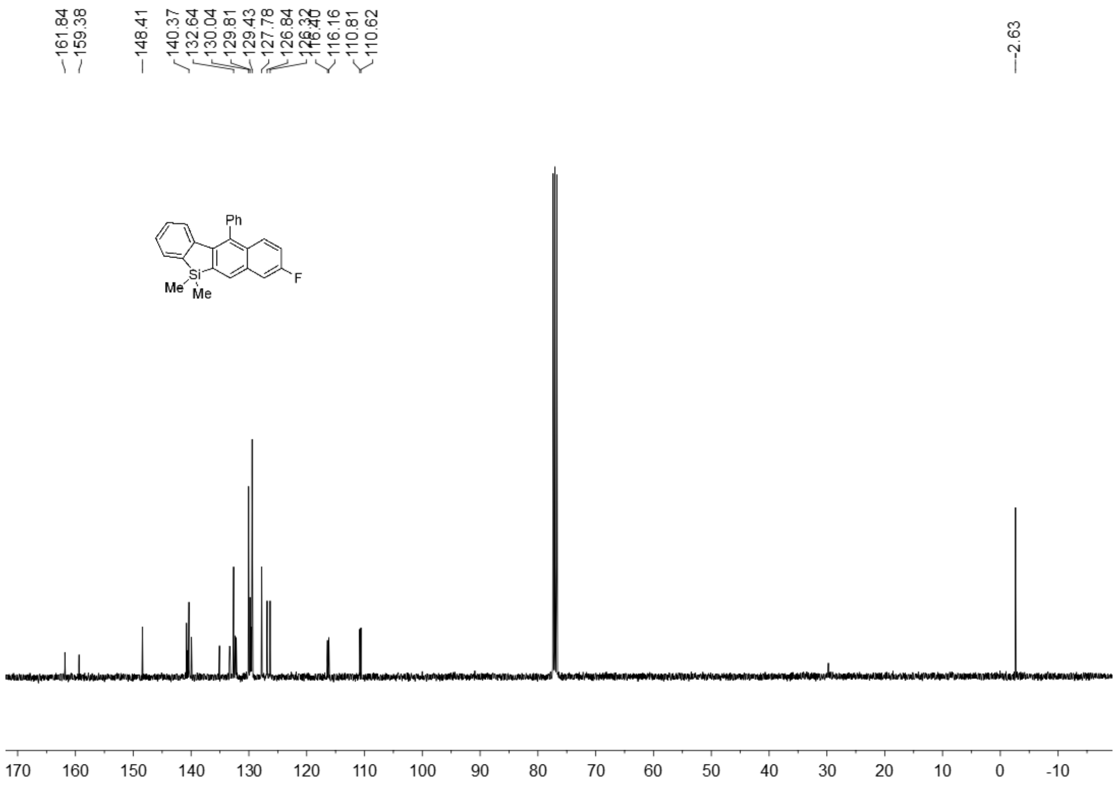

^{1}H NMR spectrum (400 MHz, $CDCl_3$) of **6i**

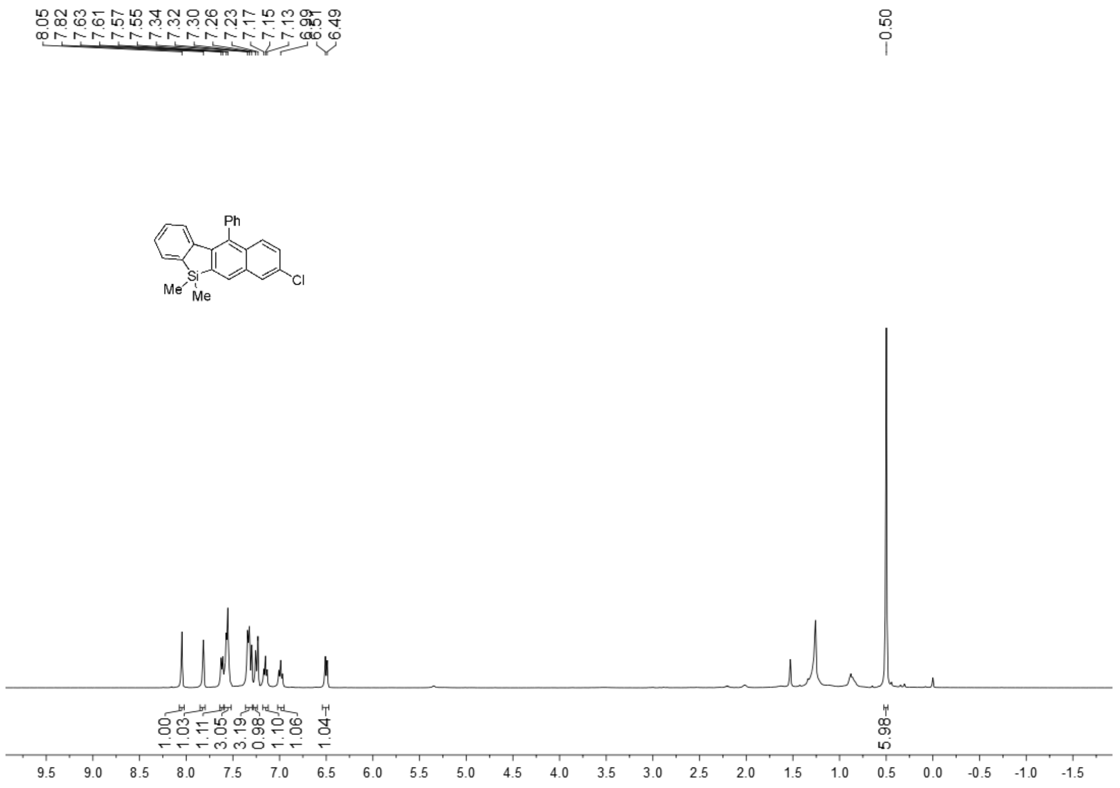

^{13}C NMR spectrum (101 MHz, $CDCl_3$) of **6i**

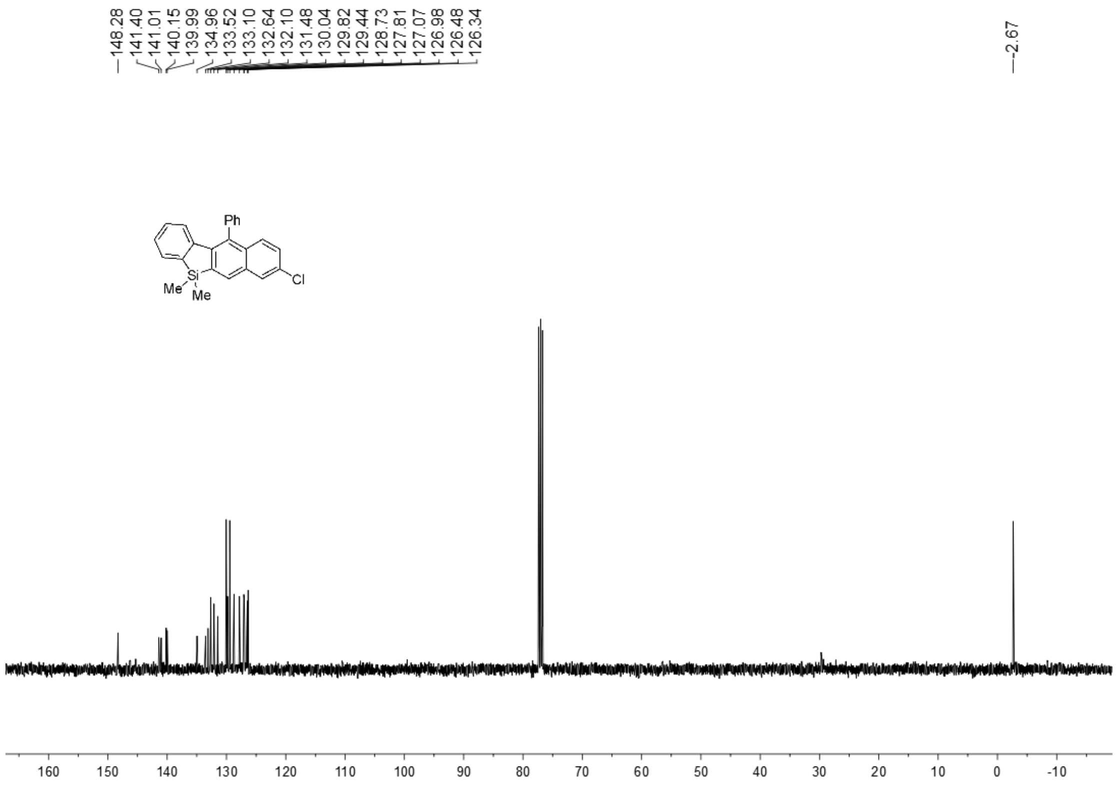

^{1}H NMR spectrum (400 MHz, $CDCl_3$) of **6j** and **6k**

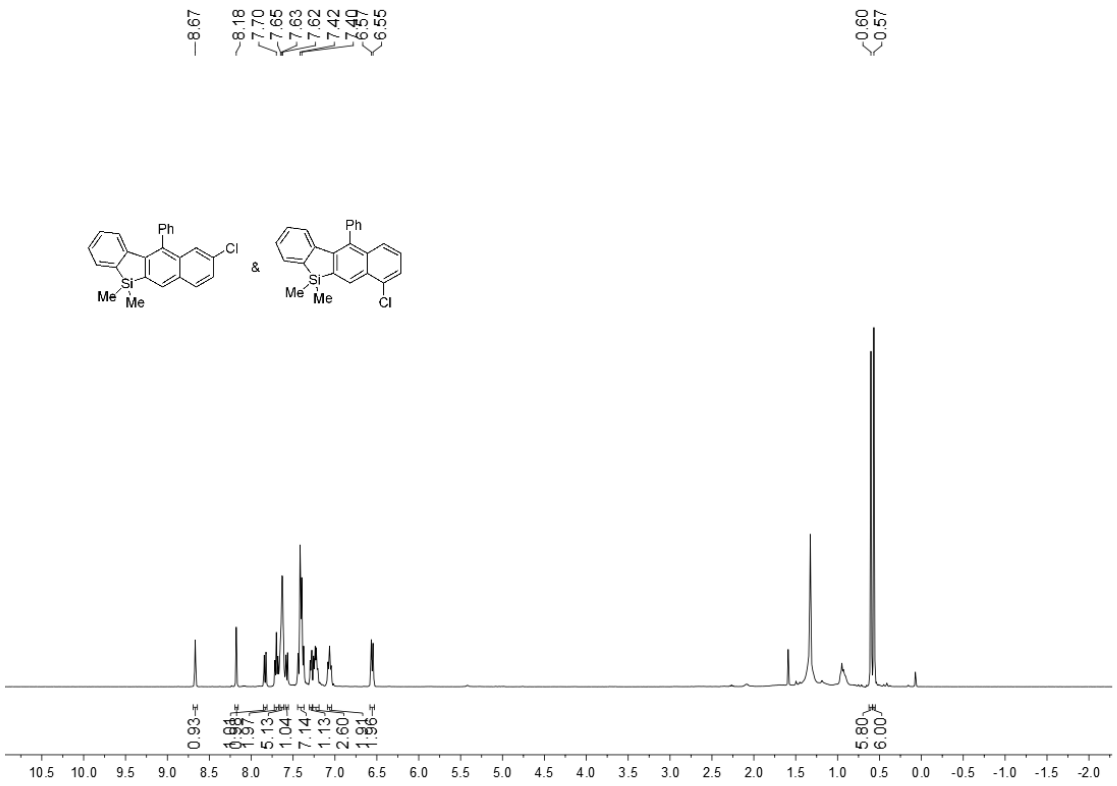

^{13}C NMR spectrum (101 MHz, $CDCl_3$) of **6j** and **6k**

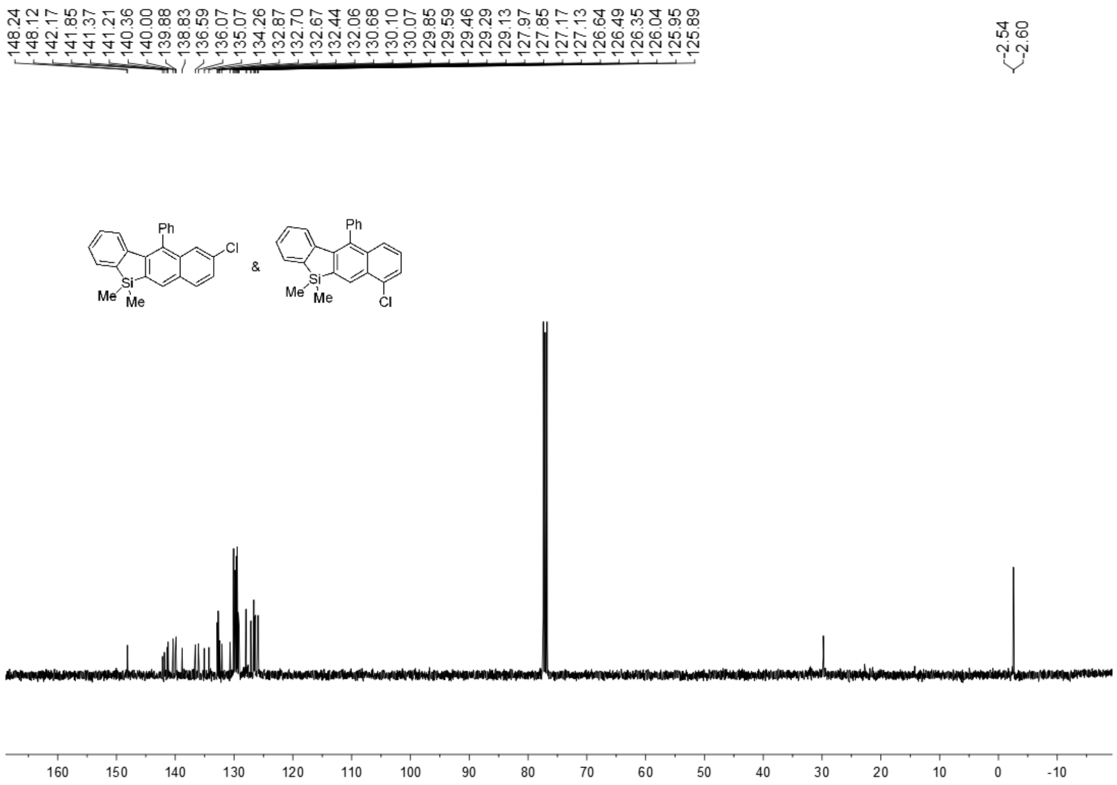

^{1}H NMR spectrum (500 MHz, $CDCl_3$) of **6l**

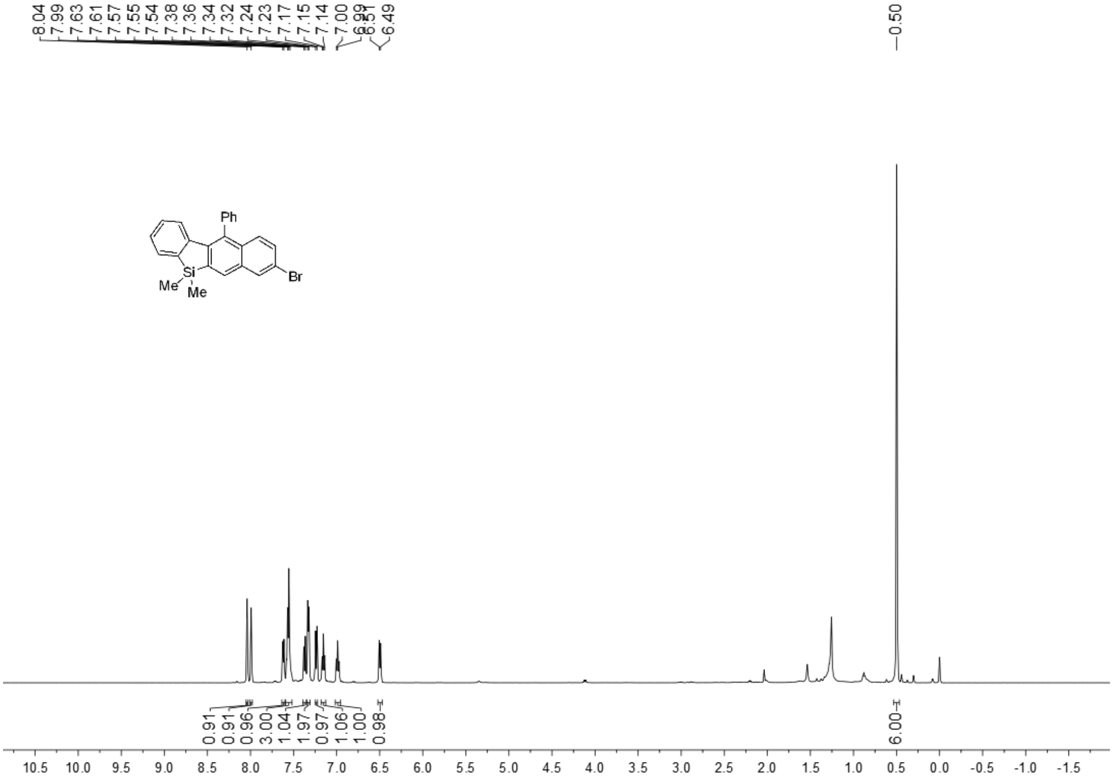

^{13}C NMR spectrum (126 MHz, $CDCl_3$) of **6l**

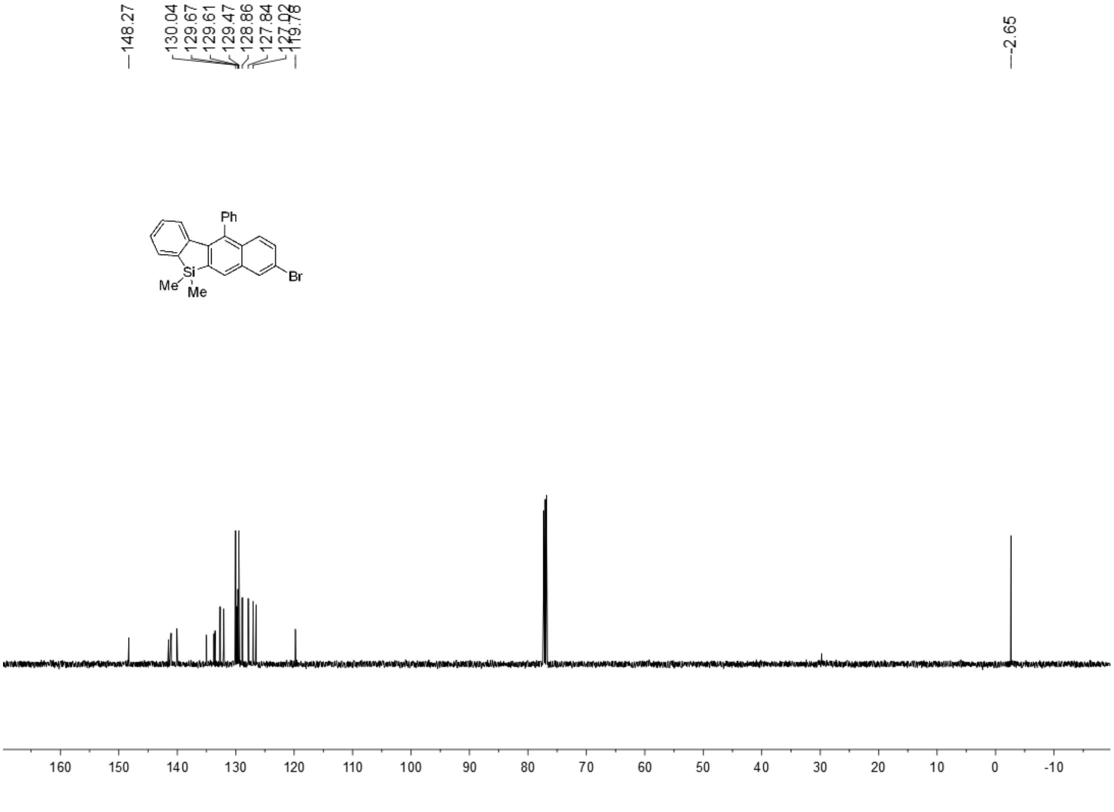

^{1}H NMR spectrum (400 MHz, $CDCl_3$) of **6m**

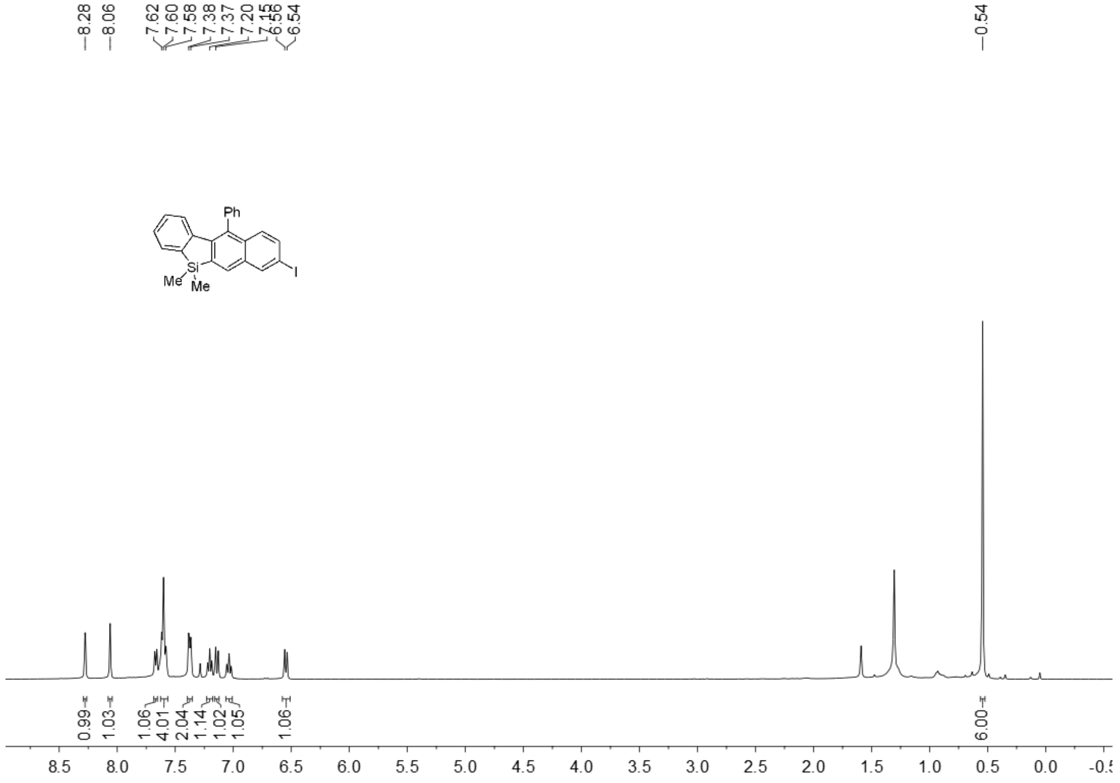

^{13}C NMR spectrum (101 MHz, $CDCl_3$) of **6m**

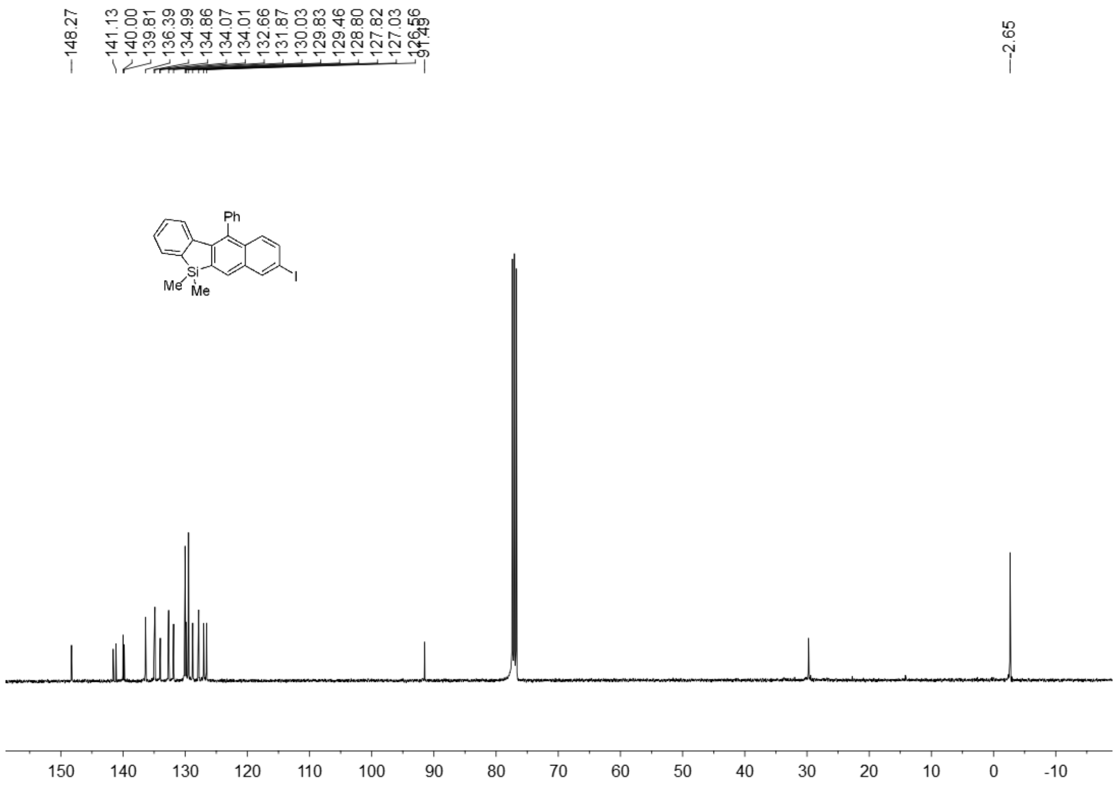

^{1}H NMR spectrum (400 MHz, DMSO) of **6n**

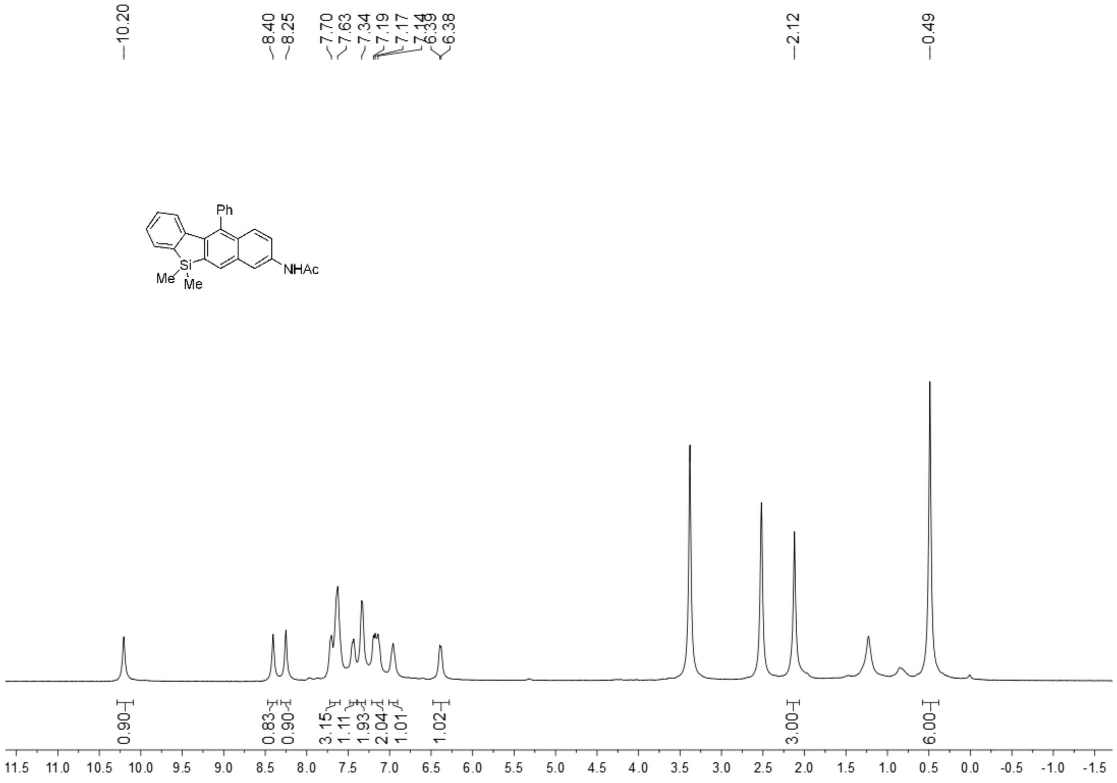

^{13}C NMR spectrum (101 MHz, DMSO) of **6n**

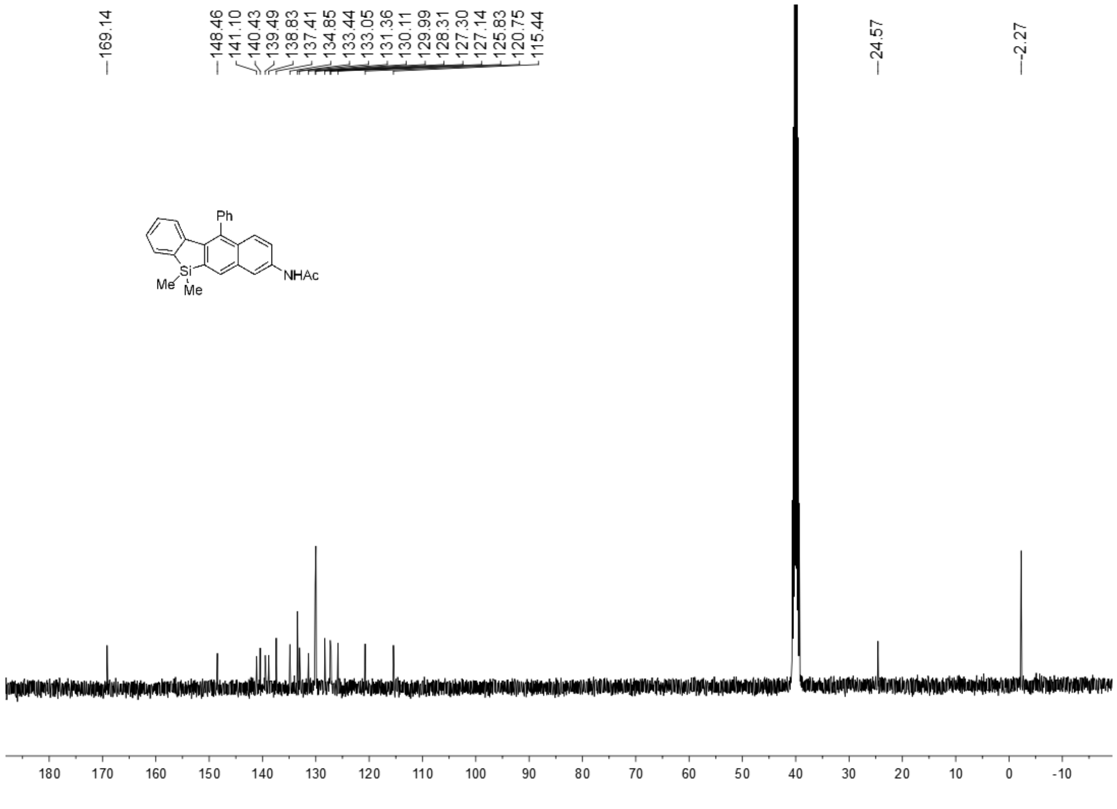

^{1}H NMR spectrum (500 MHz, $CDCl_3$) of **6o**

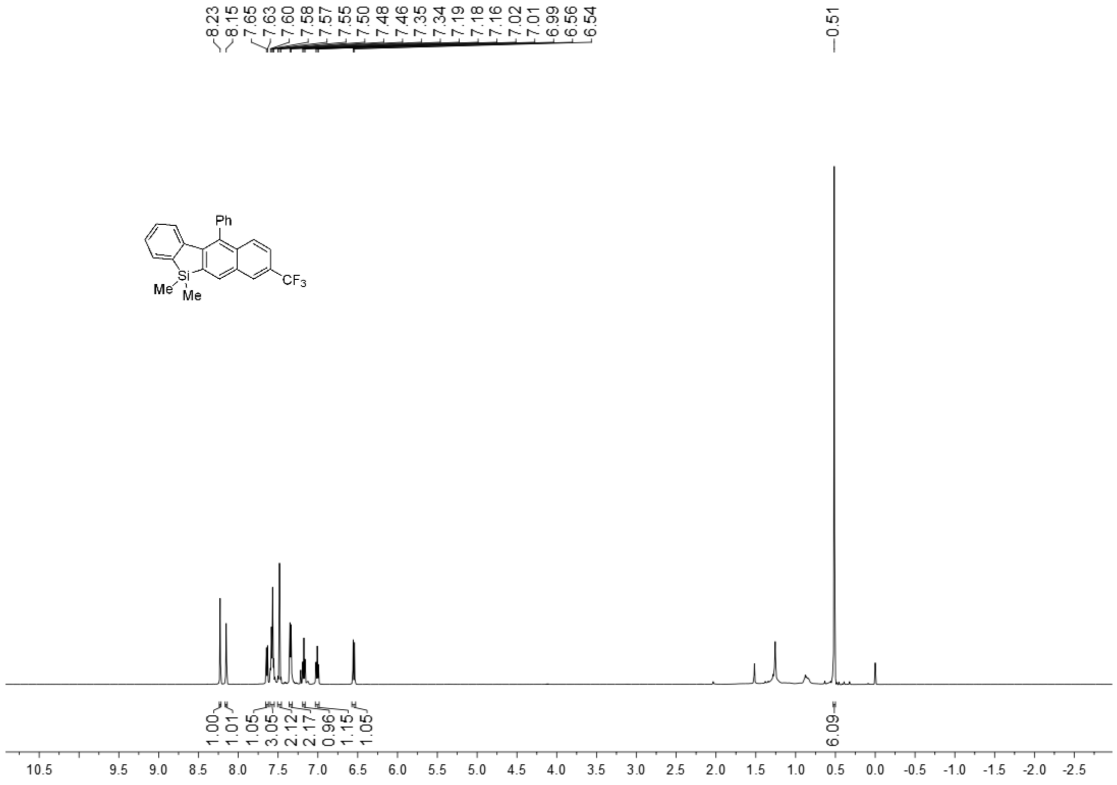

^{13}C NMR spectrum (126 MHz, $CDCl_3$) of **6o**

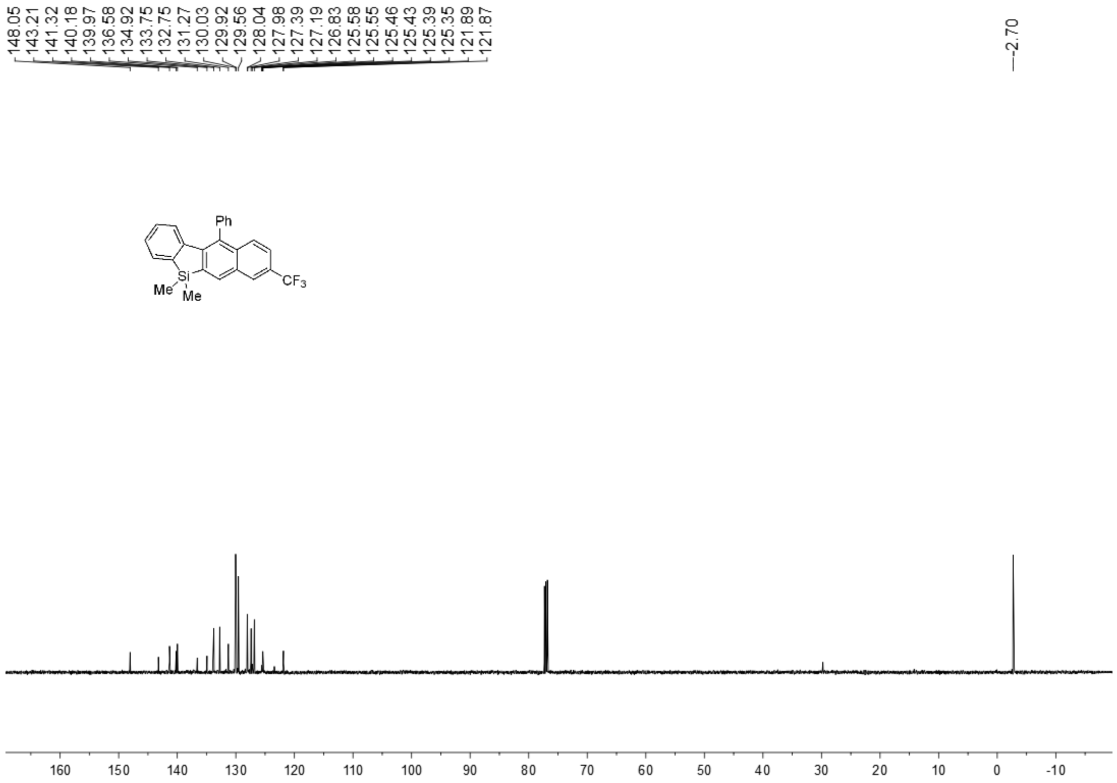

^{1}H NMR spectrum (400 MHz, $CDCl_3$) of **6p**

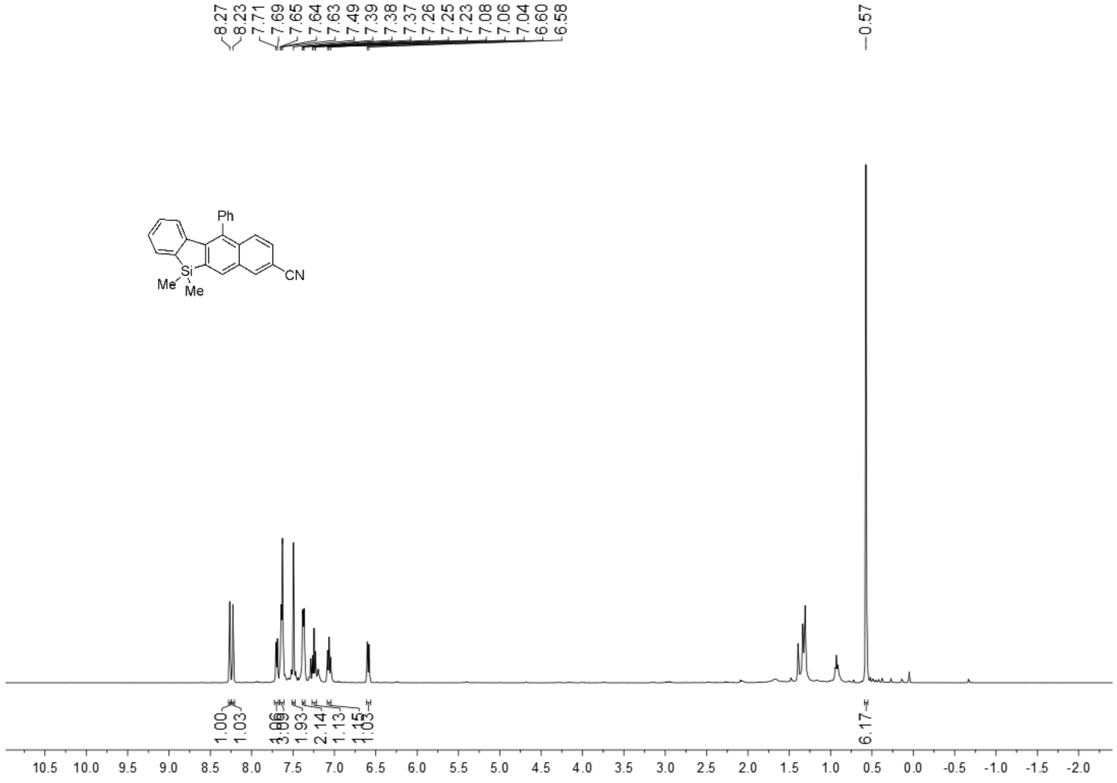

^{13}C NMR spectrum (101 MHz, $CDCl_3$) of **6p**

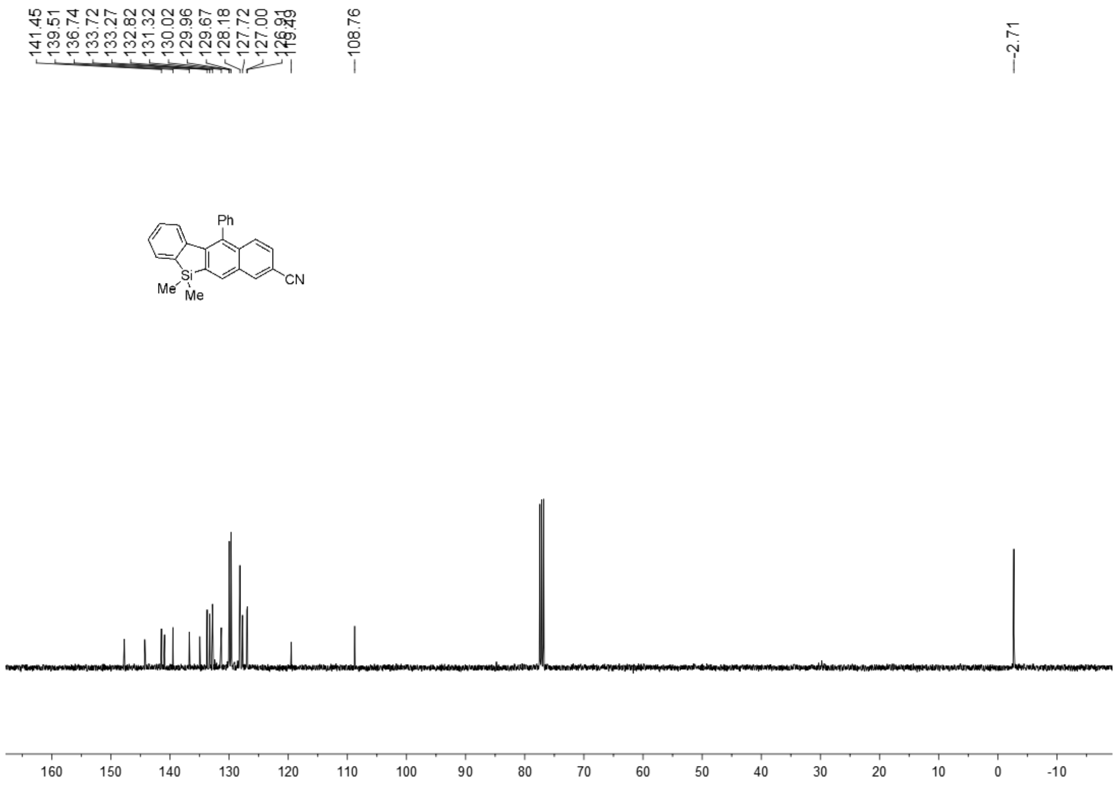

^{1}H NMR spectrum (500 MHz, $CDCl_3$) of **6q**

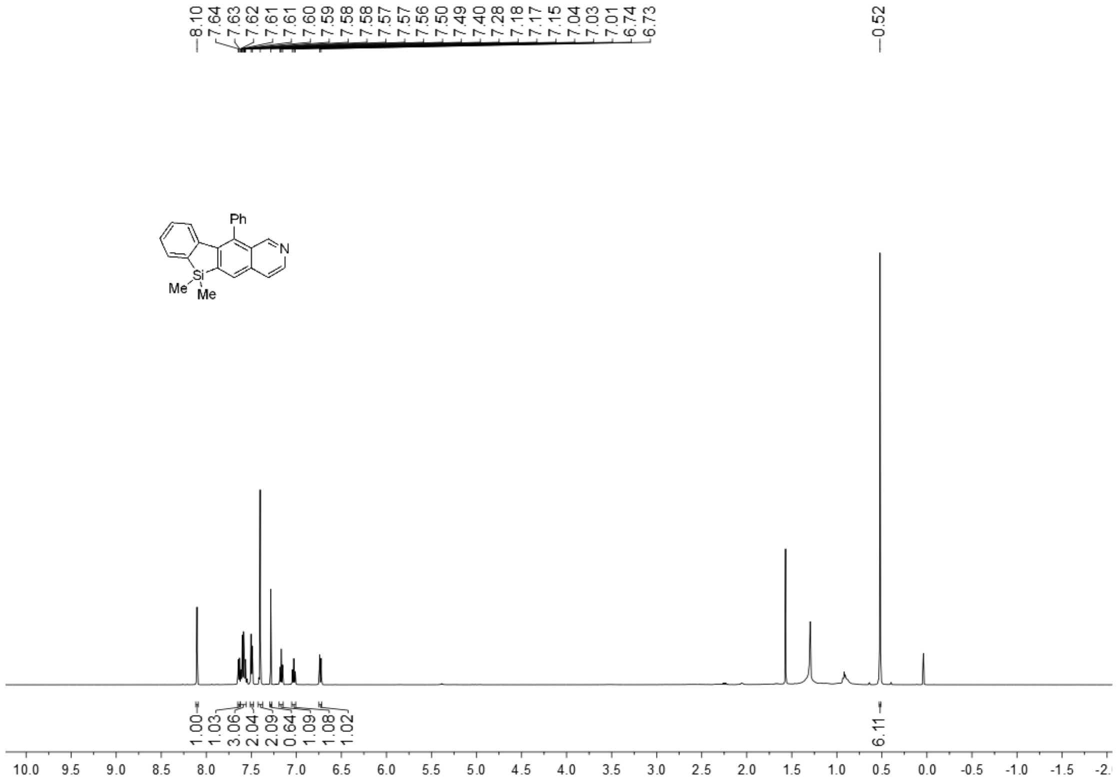

^{13}C NMR spectrum (126 MHz, $CDCl_3$) of **6q**

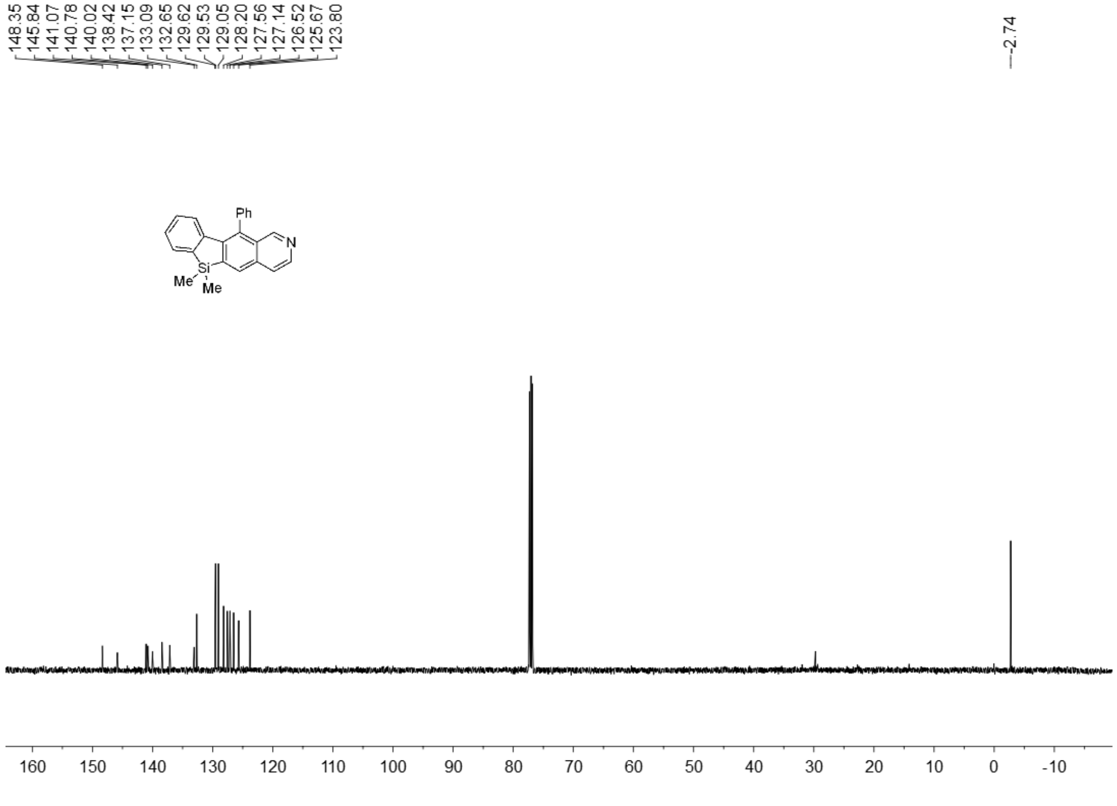

^{1}H NMR spectrum (400 MHz, $CDCl_3$) of **6r**

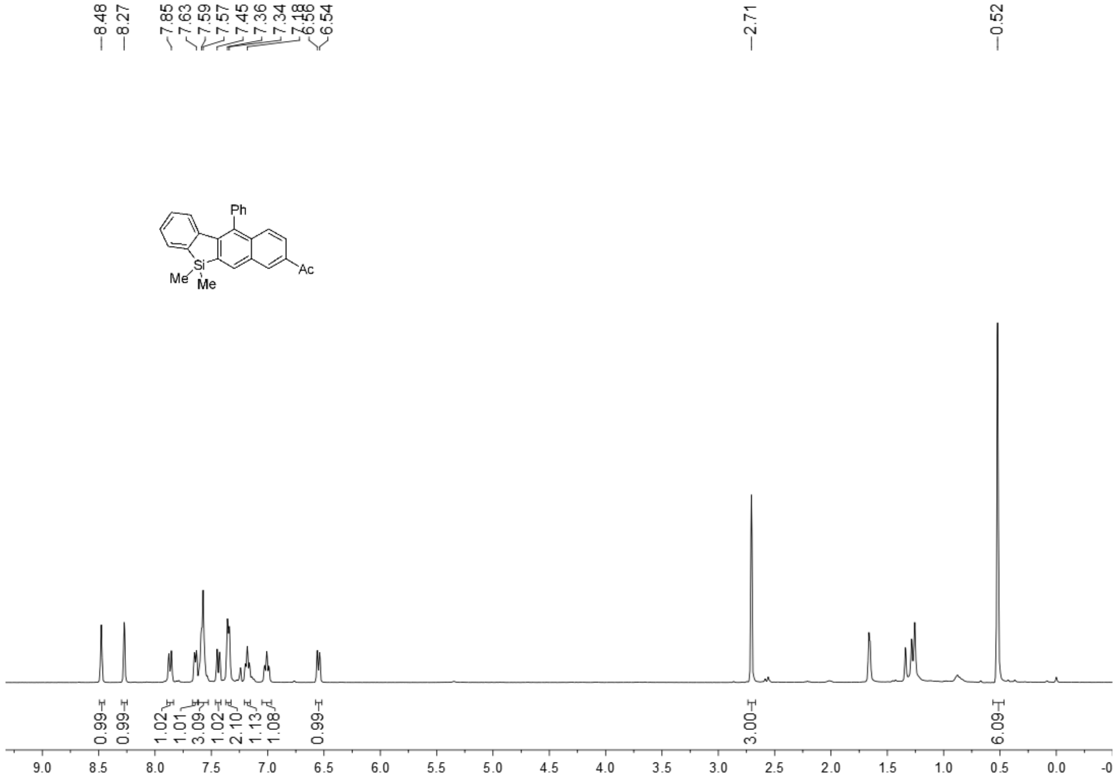

^{13}C NMR spectrum (101 MHz, $CDCl_3$) of **6r**

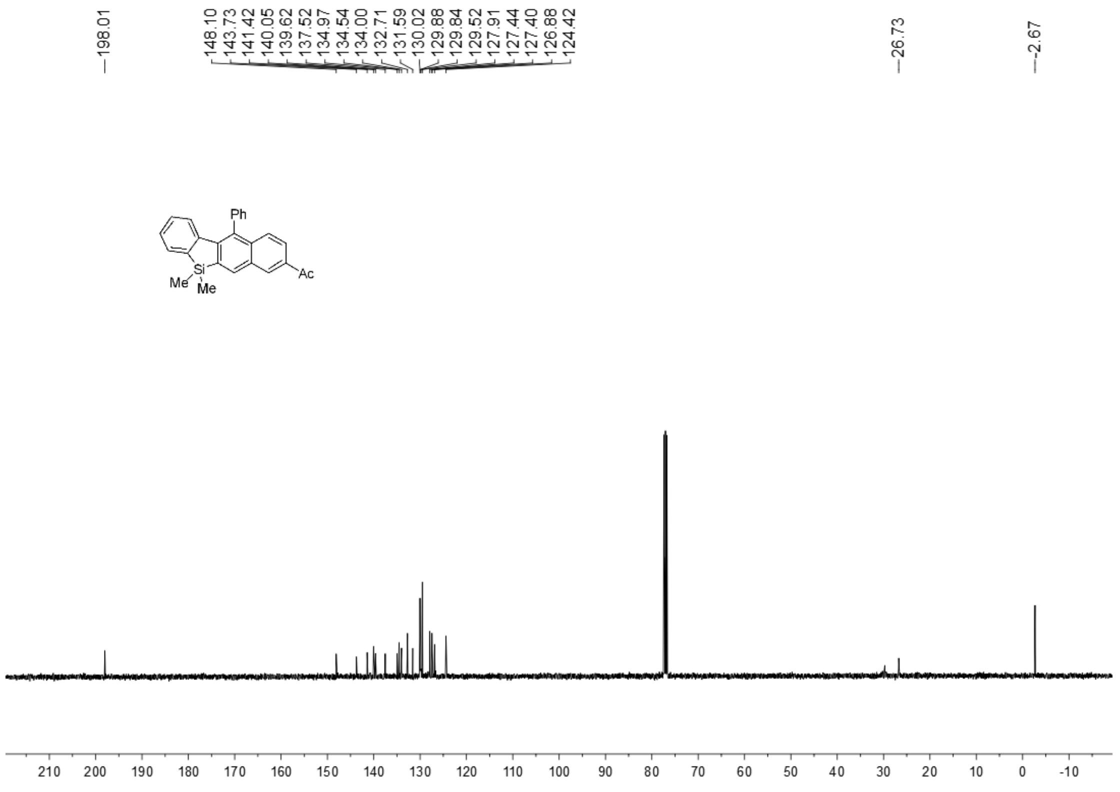

^{1}H NMR spectrum (500 MHz, $CDCl_3$) of **6s**

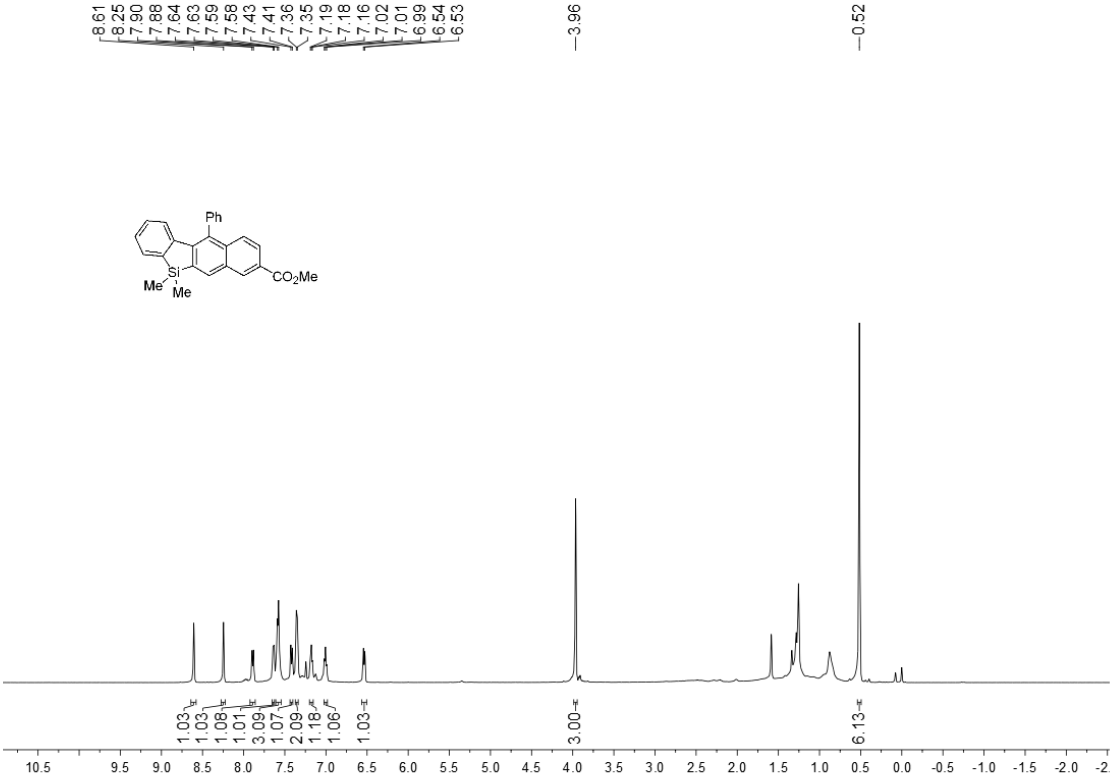

^{13}C NMR spectrum (126 MHz, $CDCl_3$) of **6s**

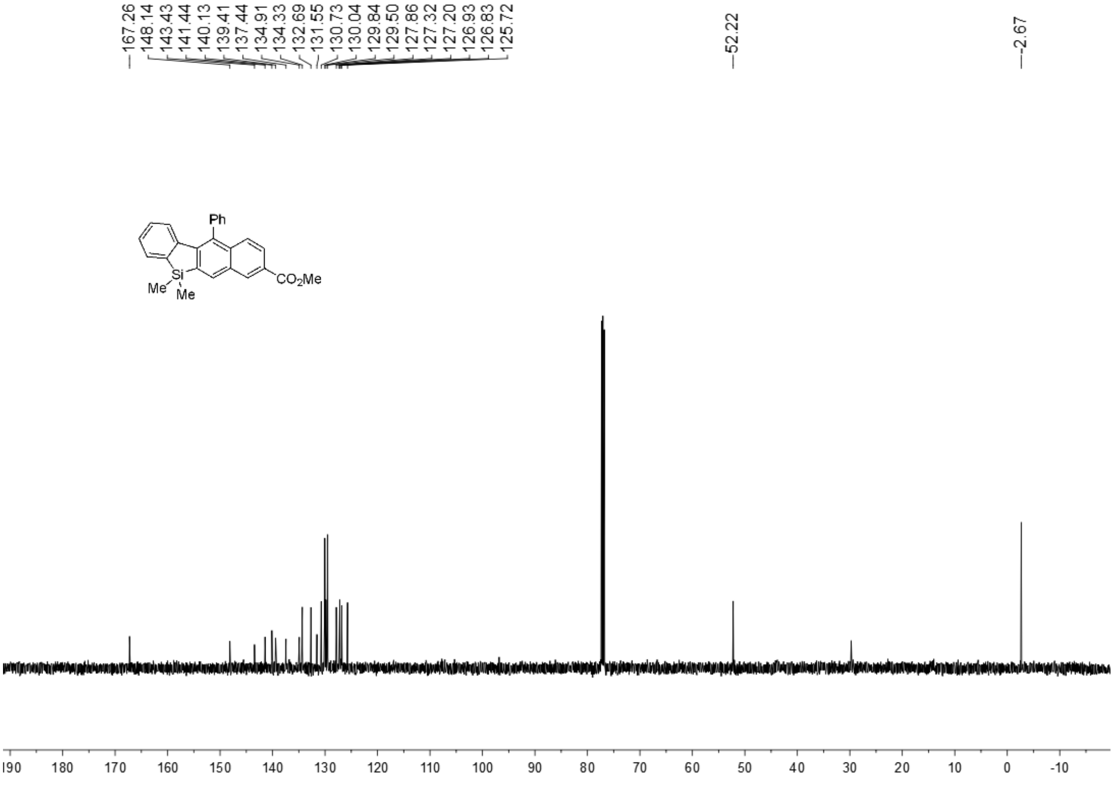

^{1}H NMR spectrum (400 MHz, DMSO) of **6t**

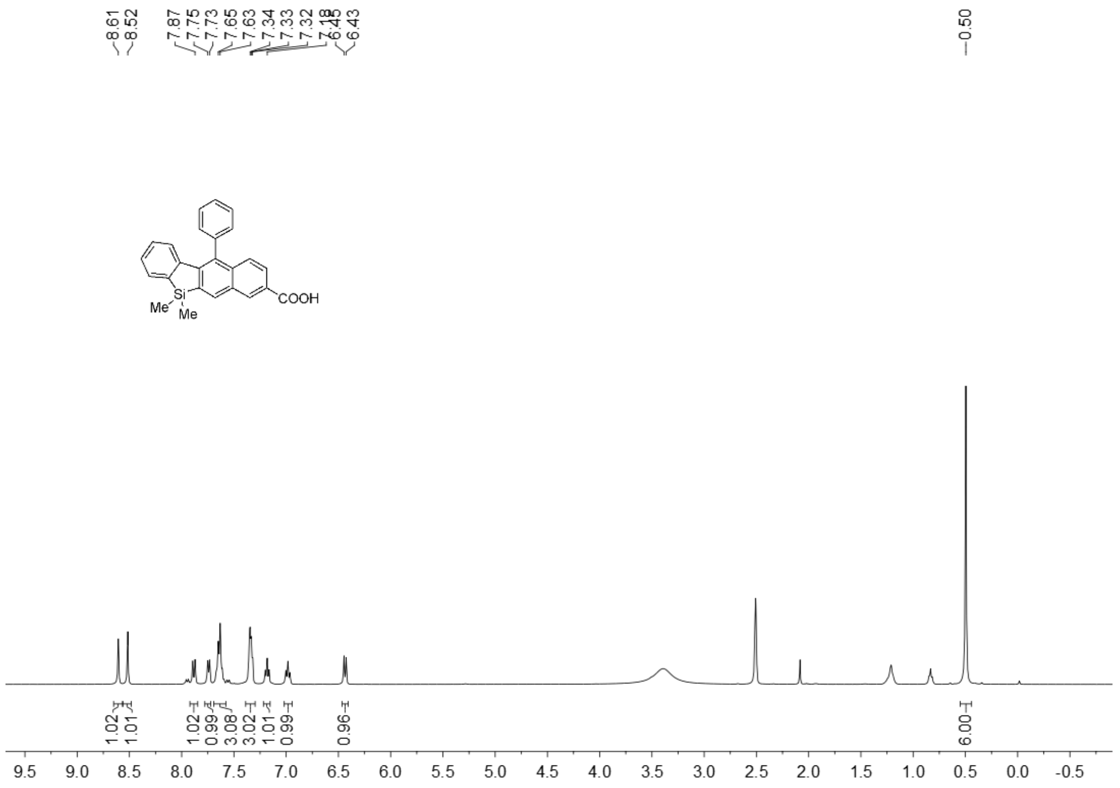

^{13}C NMR spectrum (101 MHz, DMSO) of **6t**

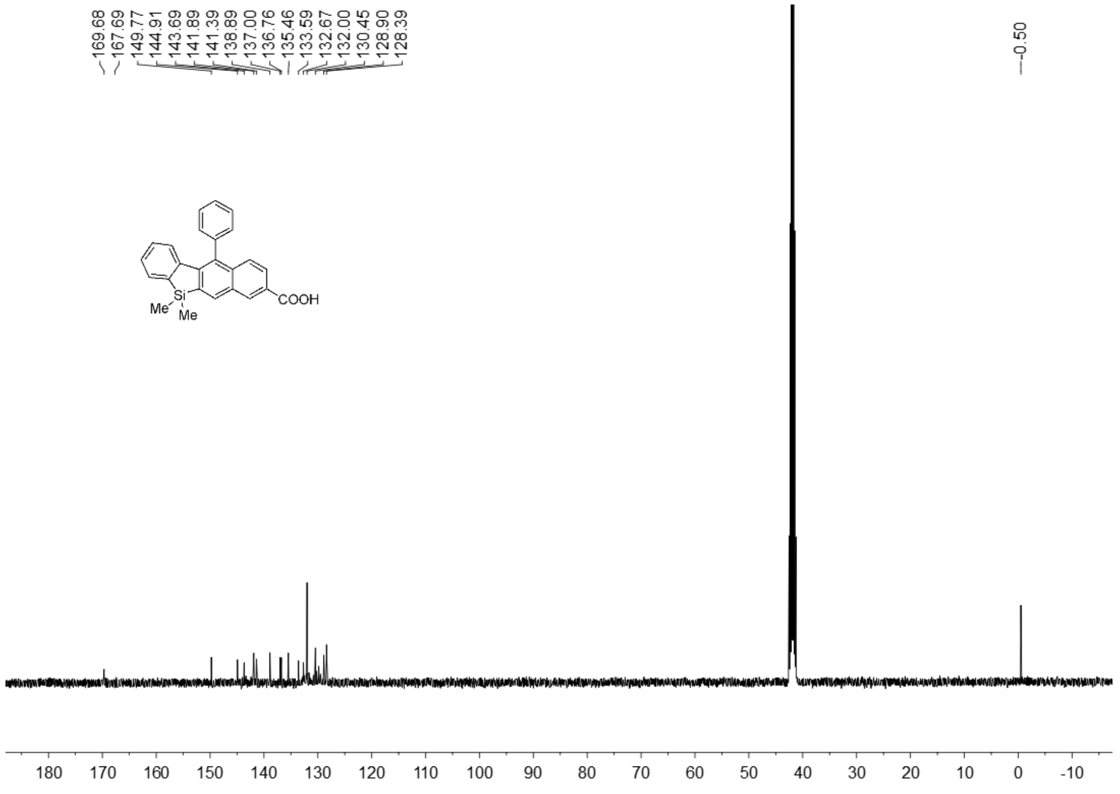

^{1}H NMR spectrum (400 MHz, $CDCl_3$) of **8**

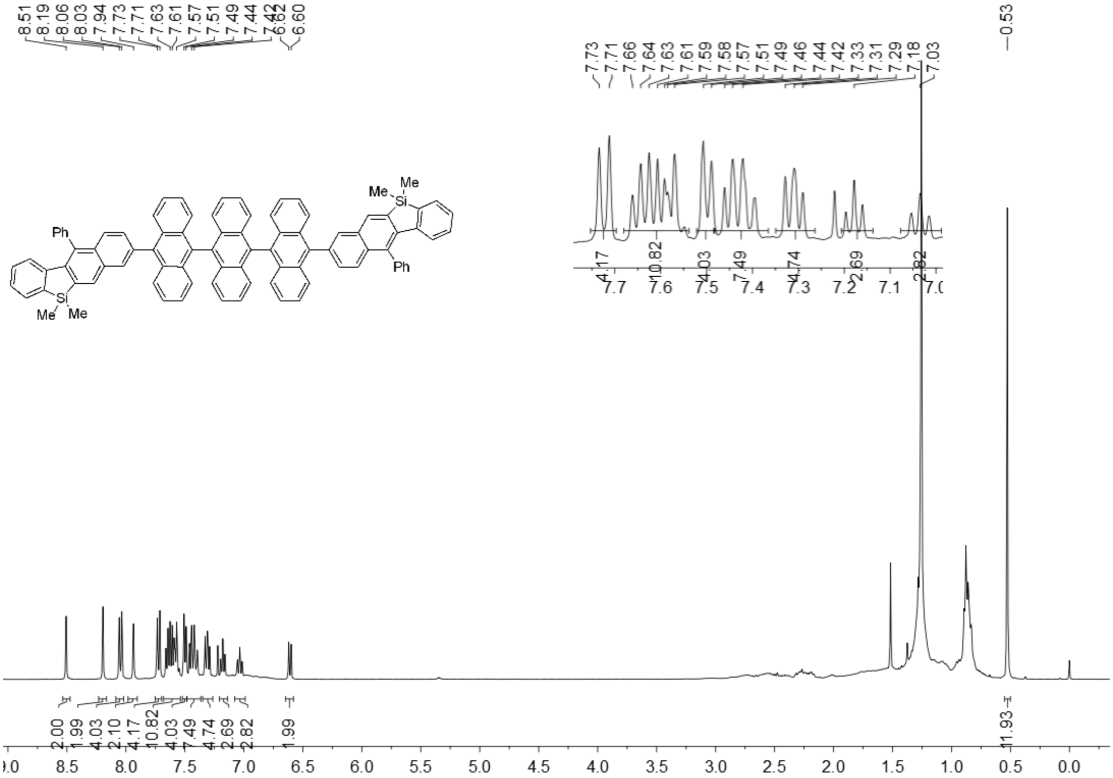

^{13}C NMR spectrum (101 MHz, $CDCl_3$) of **8**

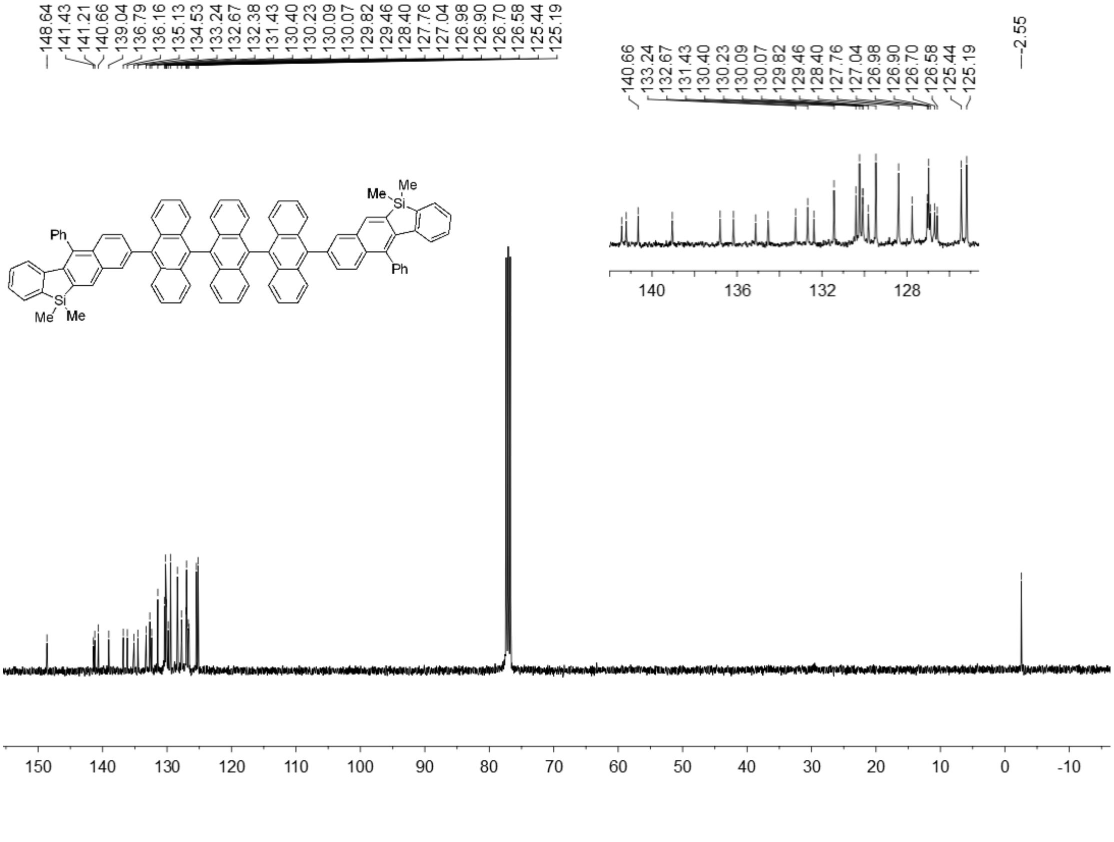

^{1}H NMR spectrum (500 MHz, $CDCl_3$) of **9**

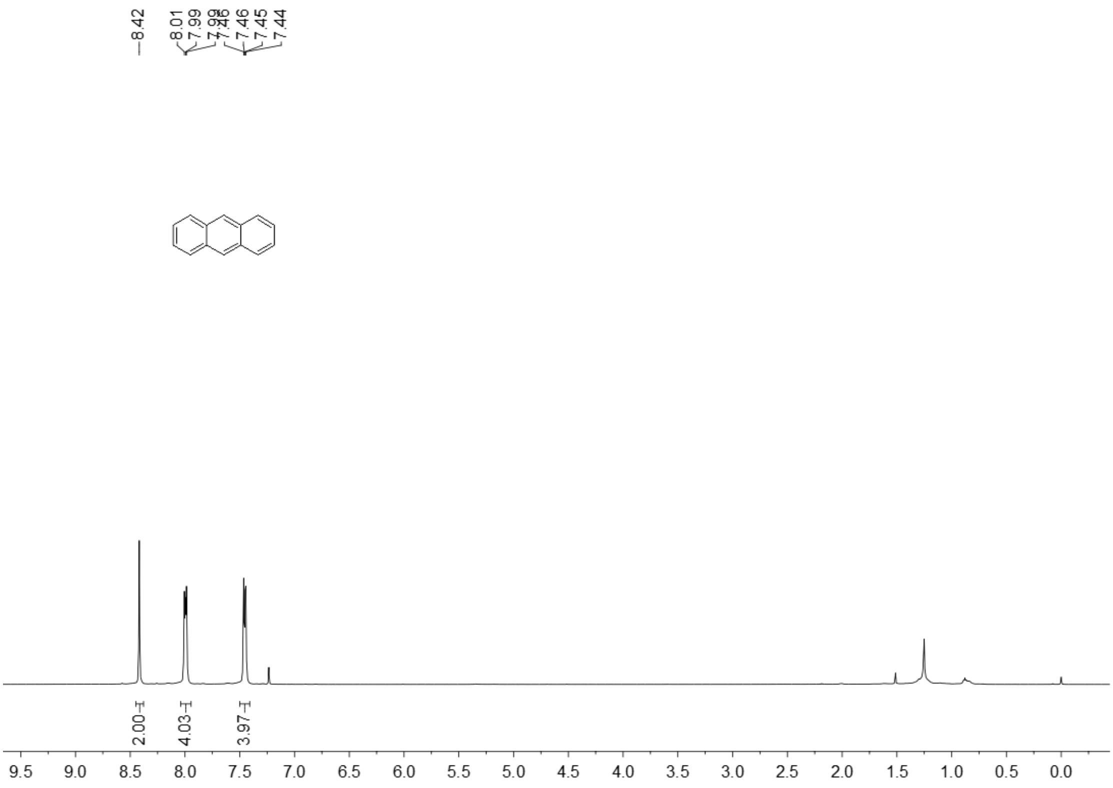

^{13}C NMR spectrum (126 MHz, DMSO) of **9**

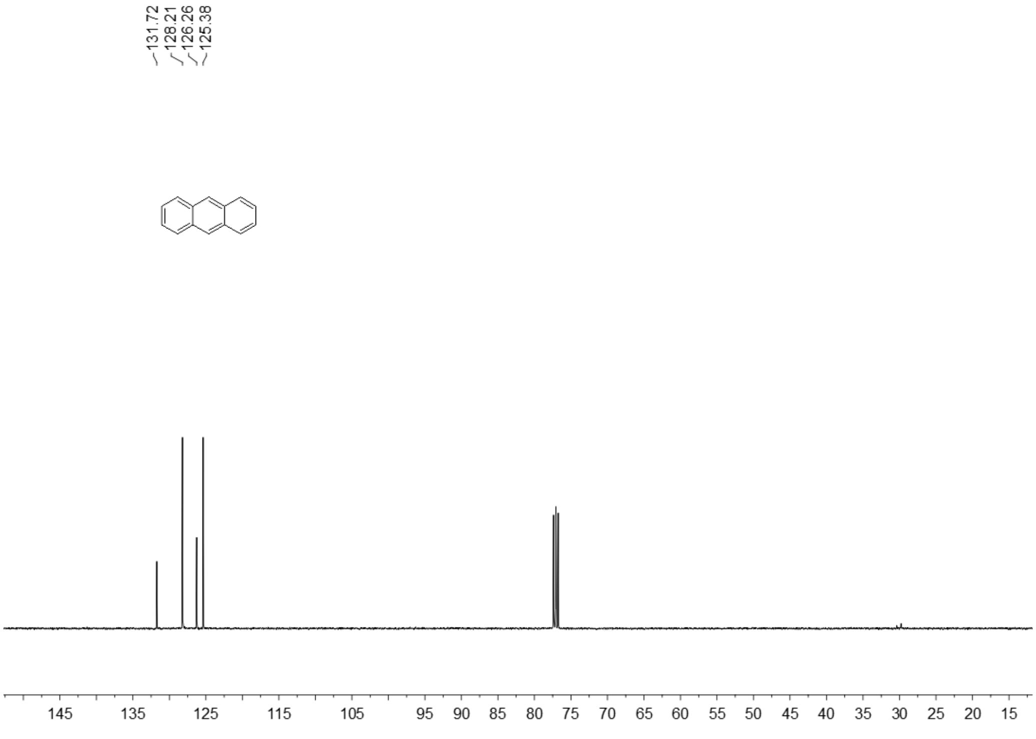

^{1}H NMR spectrum (400 MHz, DMSO) of **10**

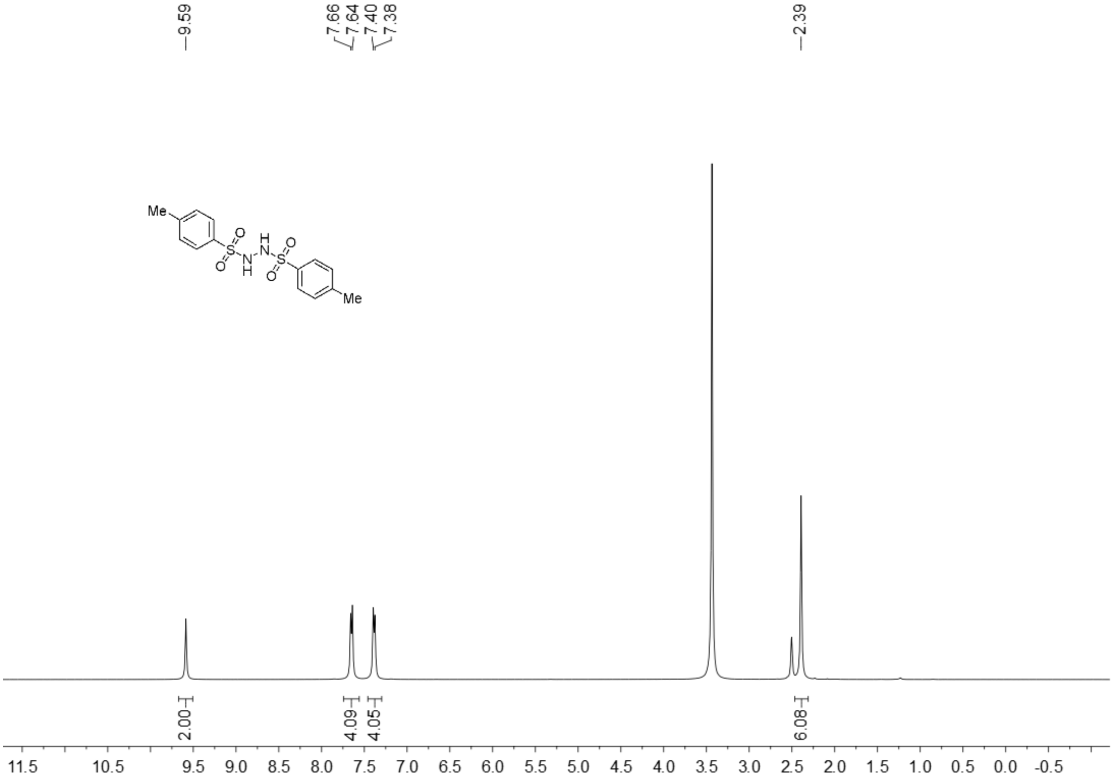

^{13}C NMR spectrum (126 MHz, DMSO) of **10**

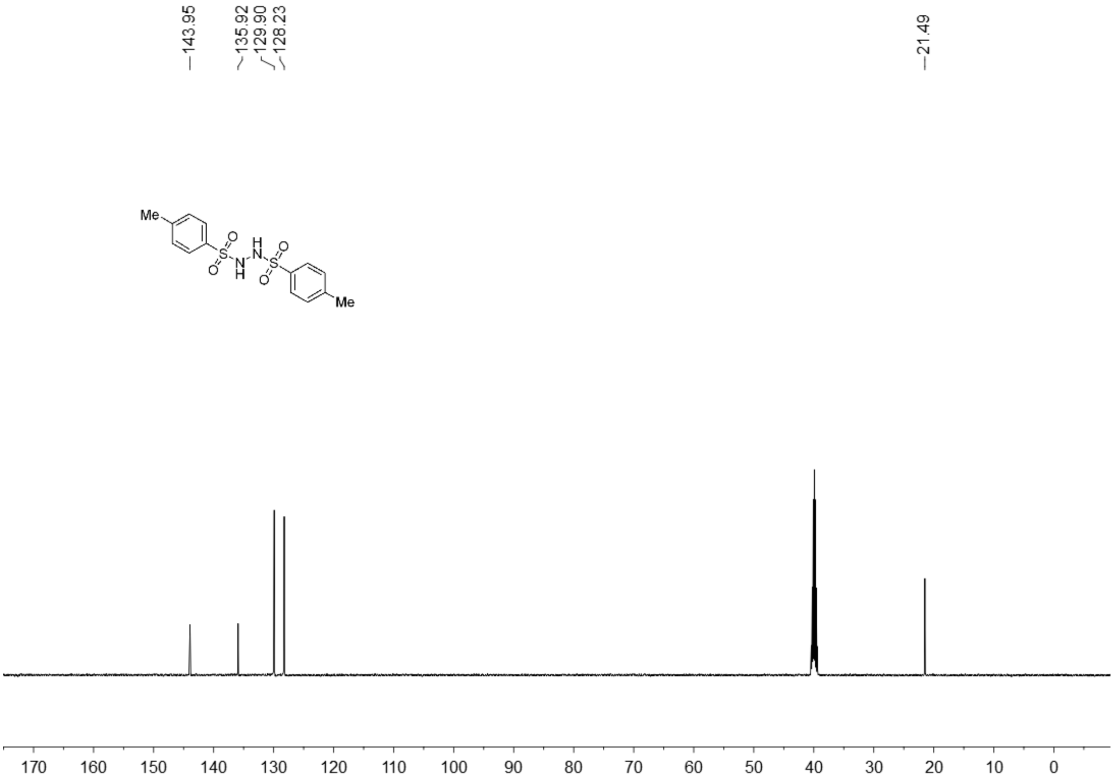

^{1}H NMR spectrum (400 MHz, $CDCl_3$) of **12**

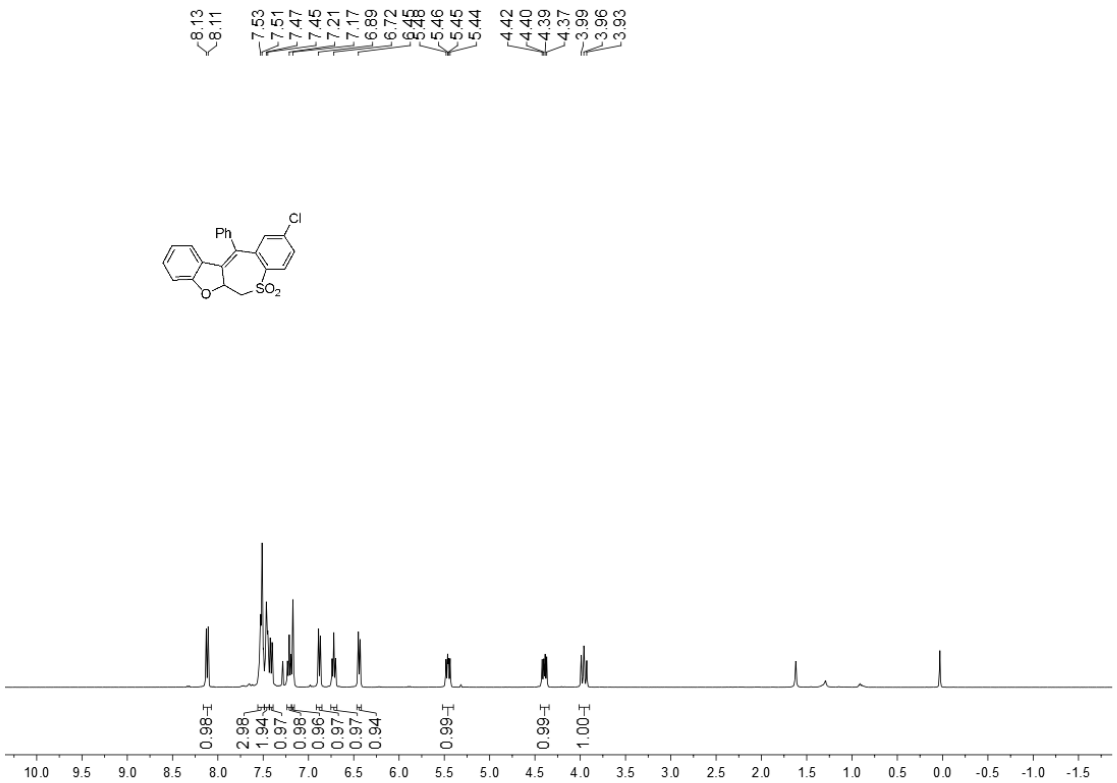

^{13}C NMR spectrum (101 MHz, $CDCl_3$) of **12**

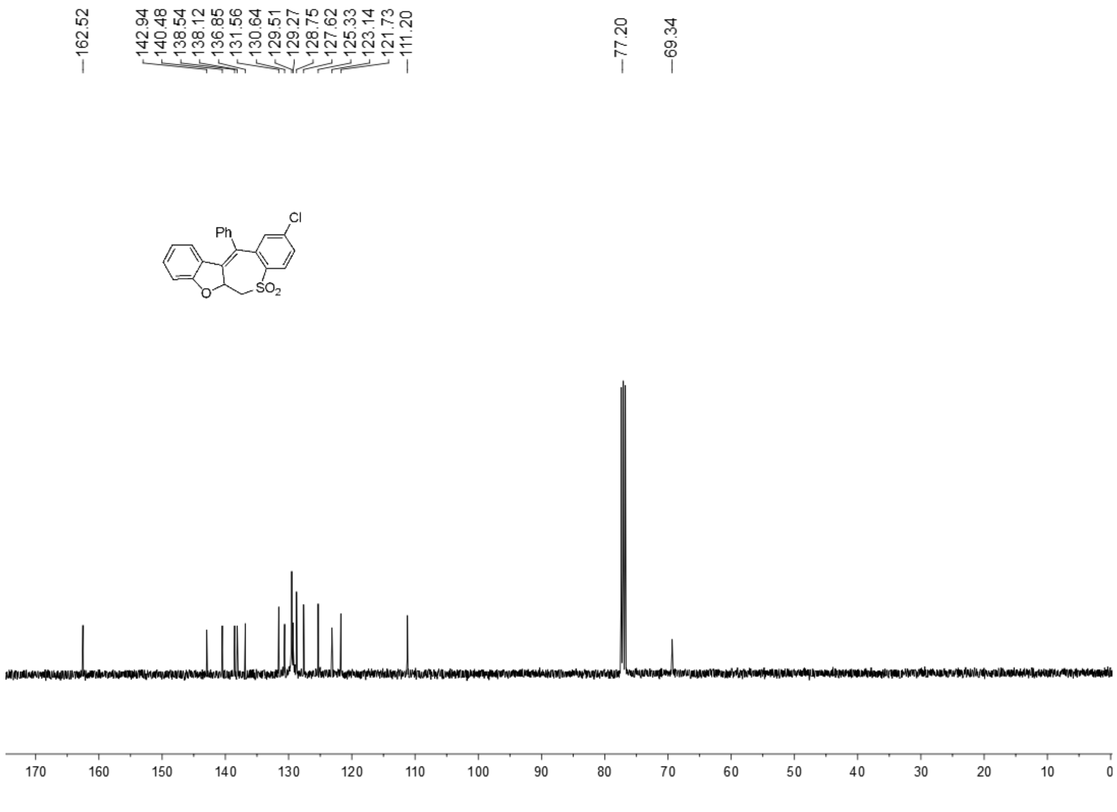

^{1}H NMR spectrum (500 MHz, $CDCl_3$) of **14**

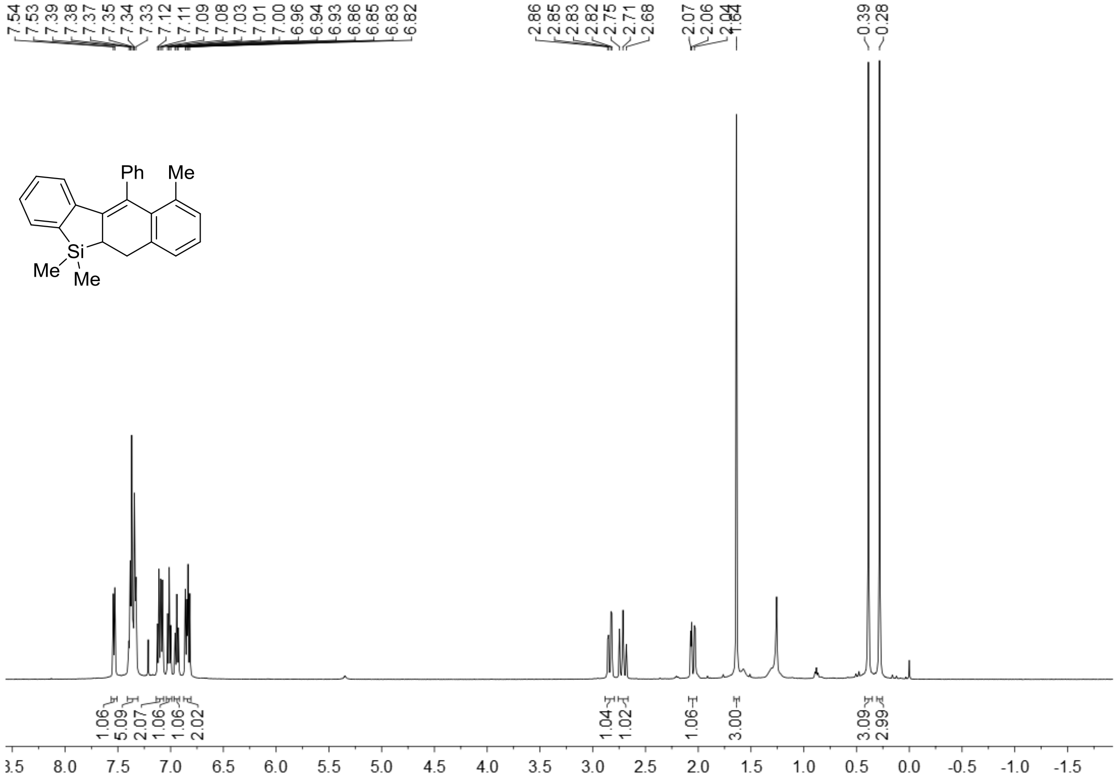

^{13}C NMR spectrum (101 MHz, $CDCl_3$) of **14**

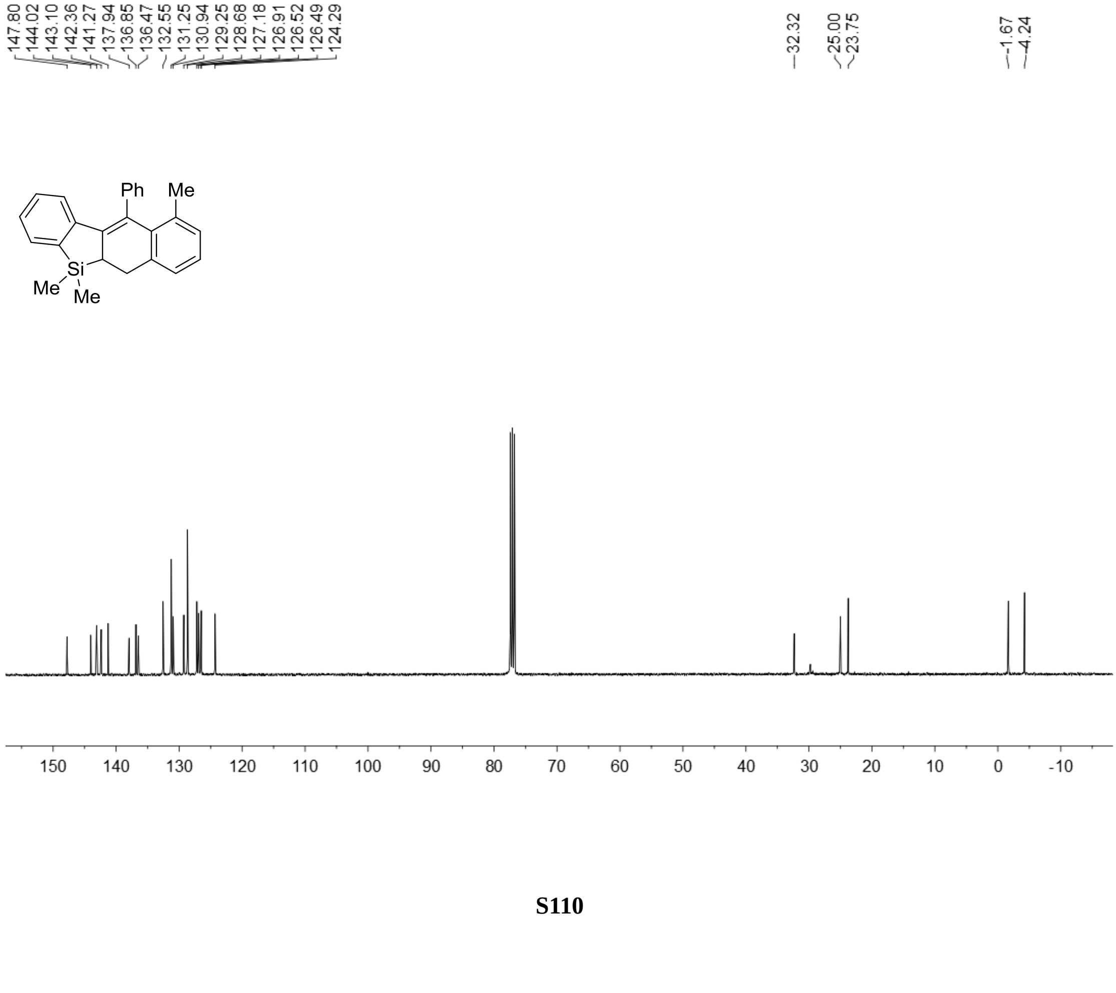

Appendix-II: Spectral Copies of HR-MS Spectrum for All the Isolated Products

1a

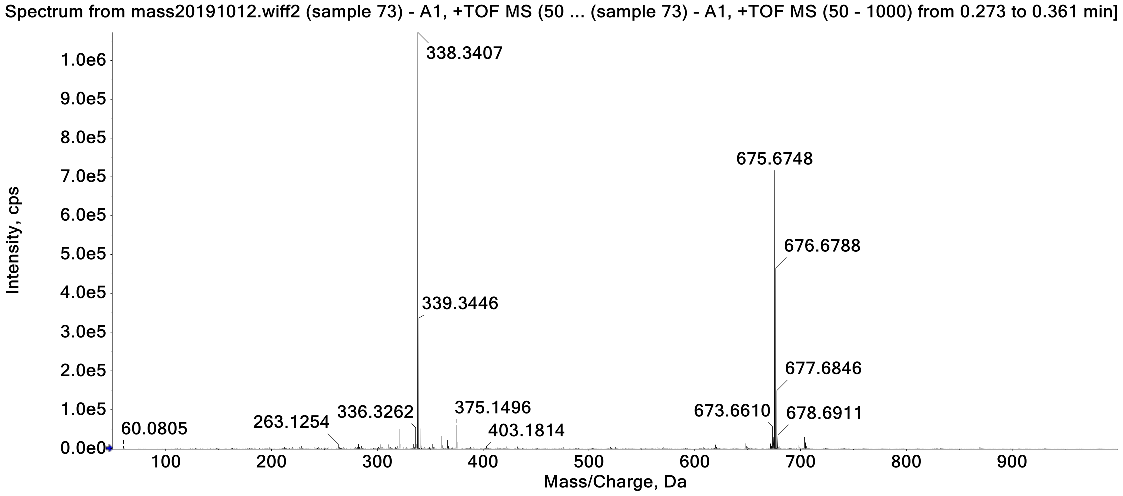

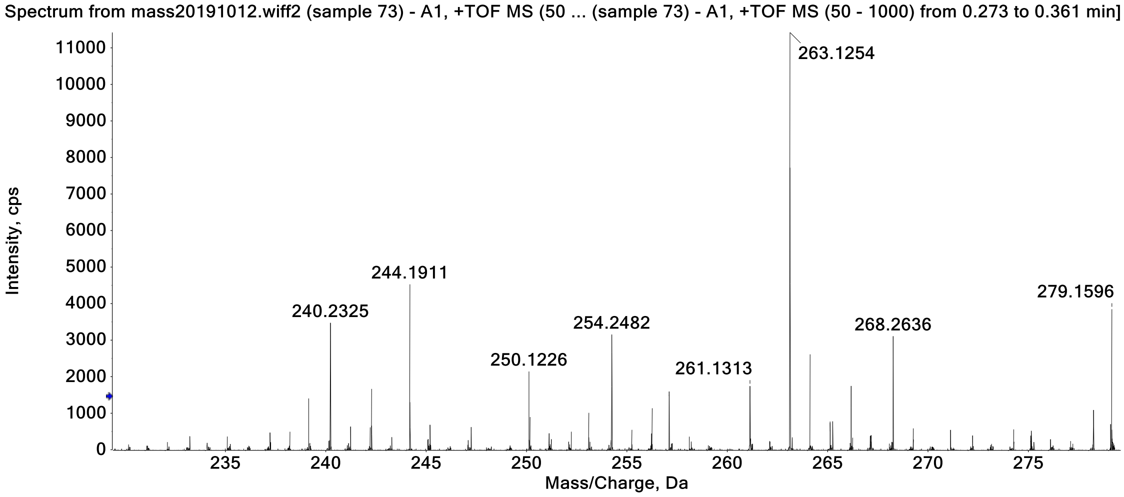

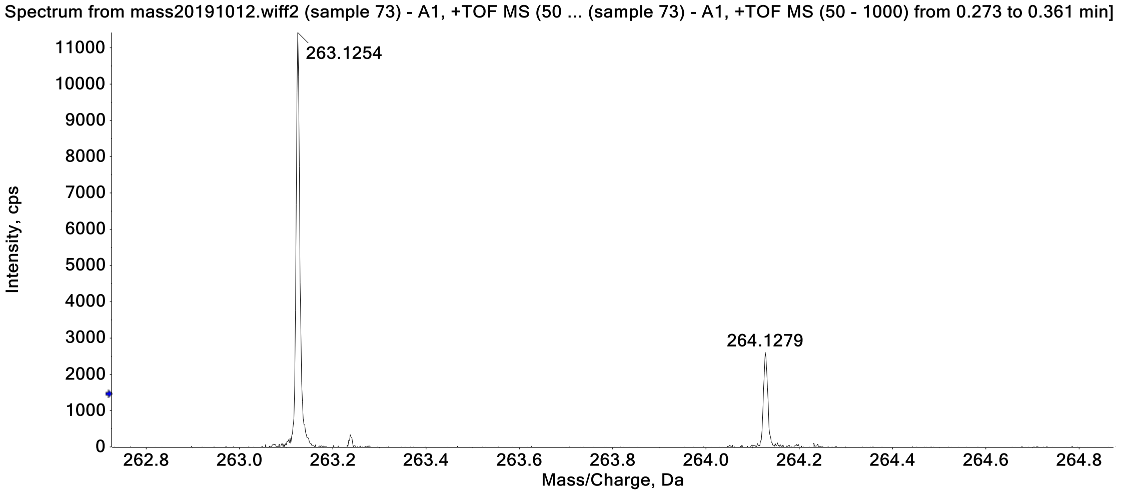

Hit	Formula	m/z	RDB	ppm	MS Rank	MSMS ppm	MSMS Rank	Found
1	C18H18Si	263.1251	11.0	1.3	1			NA/NA

1b

Spectrum from mass20191012.wiff2 (sample 81) - A9, +TOF MS (50 - 1000) from 0.044 to 0.070 min

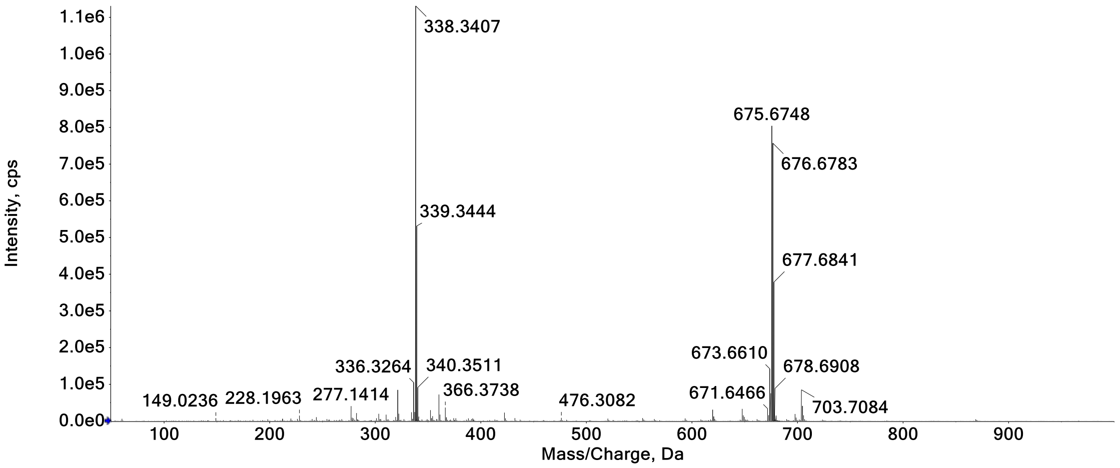

Spectrum from mass20191012.wiff2 (sample 81) - A9, +TOF MS (50 - 1000) from 0.044 to 0.070 min

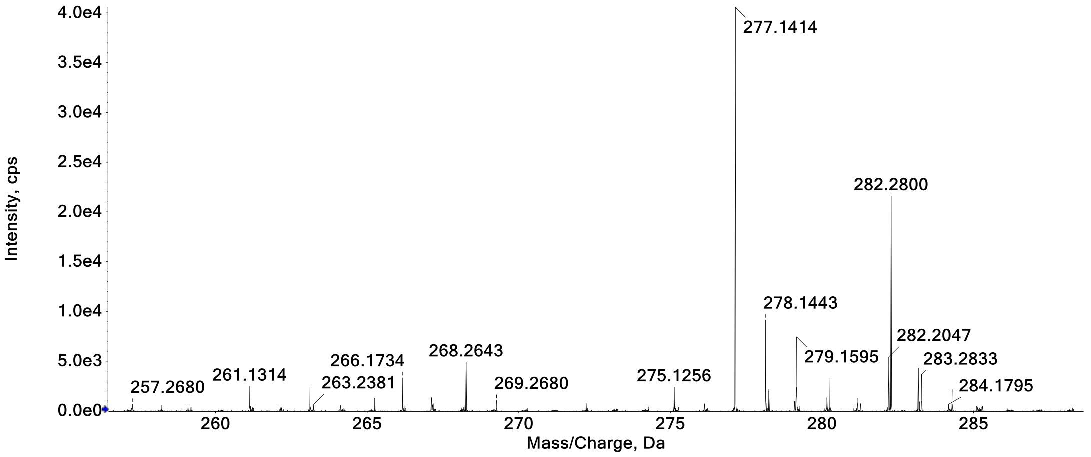

Spectrum from mass20191012.wiff2 (sample 81) - A9, +TOF MS (50 - 1000) from 0.044 to 0.070 min

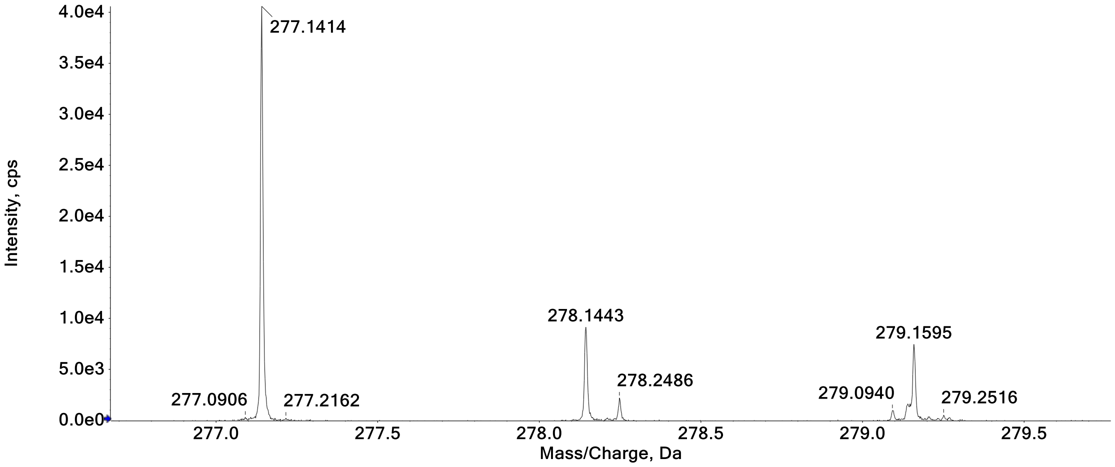

Hit	Formula	m/z	RDB	ppm	MS Rank	MSMS ppm	MSMS Rank	Found
1	C19H20Si	277.1407	11.0	2.5	1			NA/NA

1c

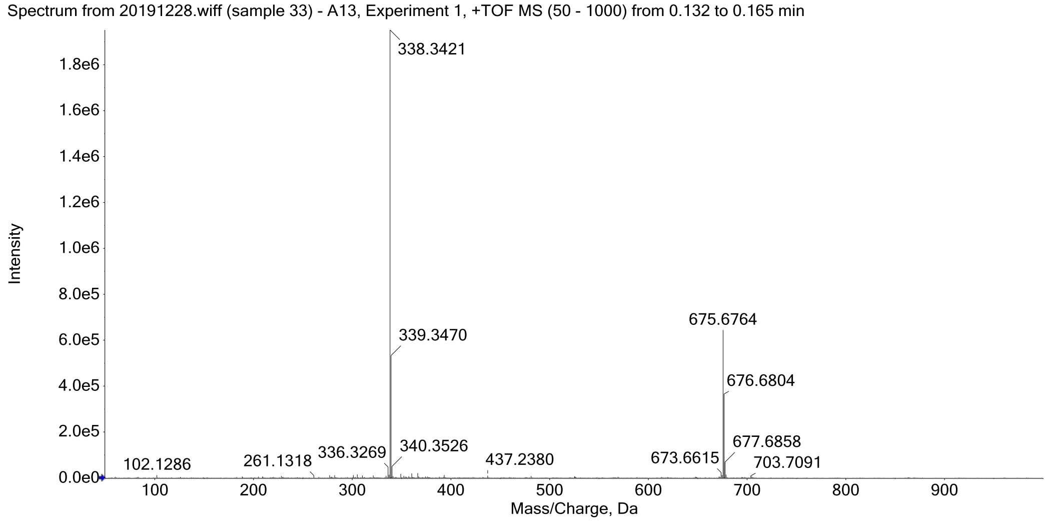

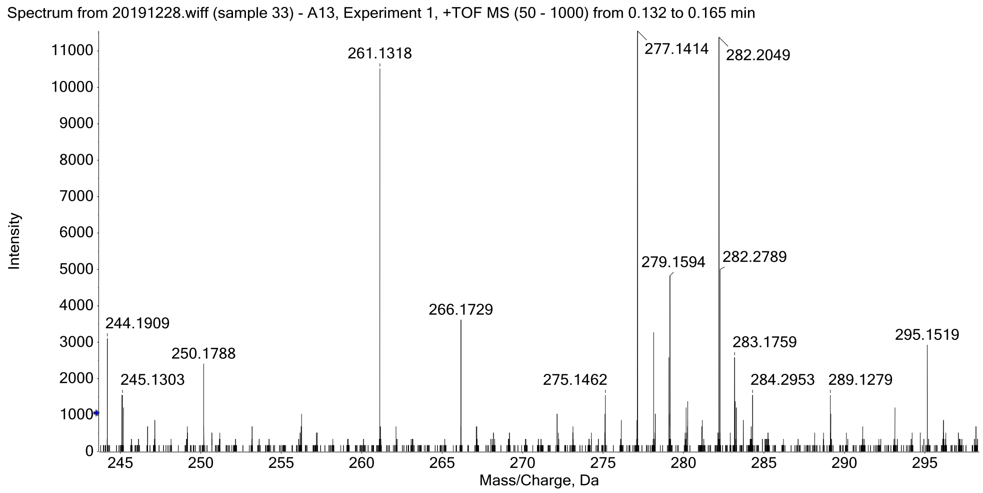

Hit	Formula	m/z	RDB	ppm	MS Rank	MSMS ppm	MSMS Rank	Found
1	SiC19H20	277.1407	11.0	2.5	1			NA/NA

1d

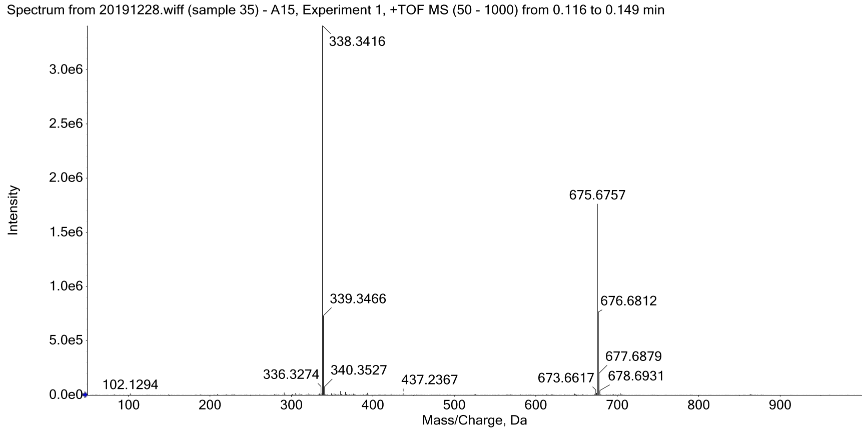

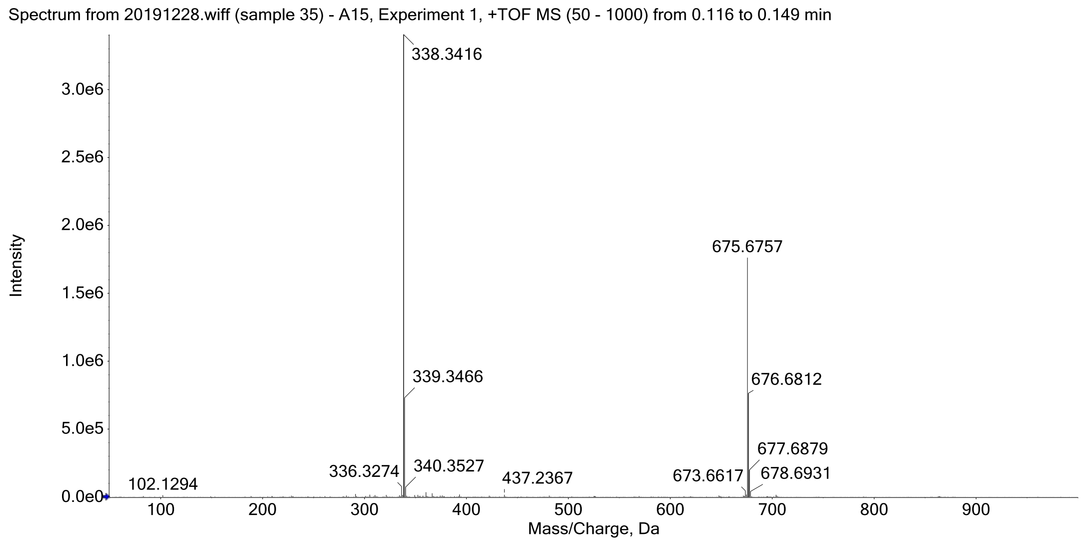

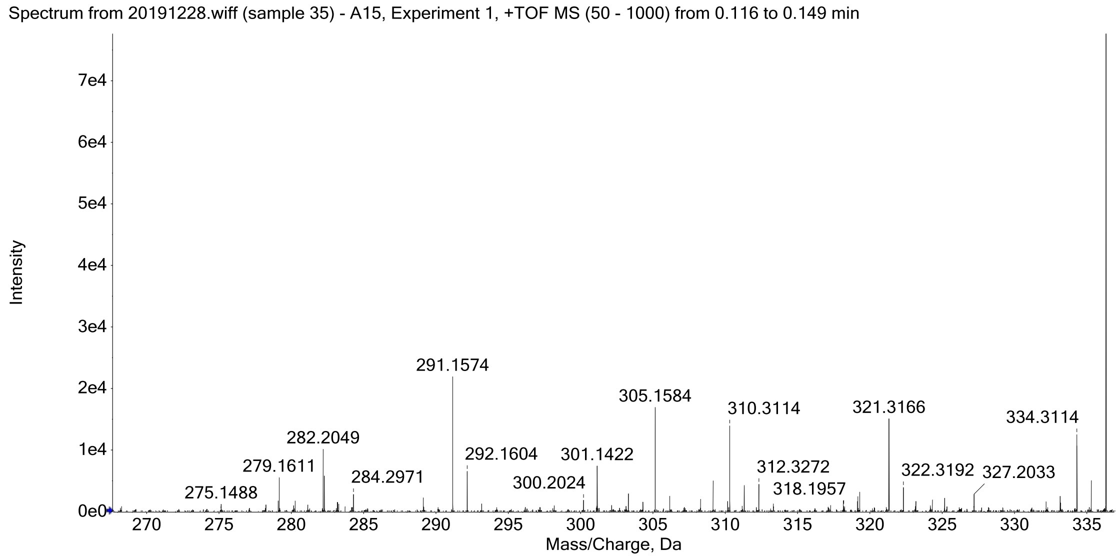

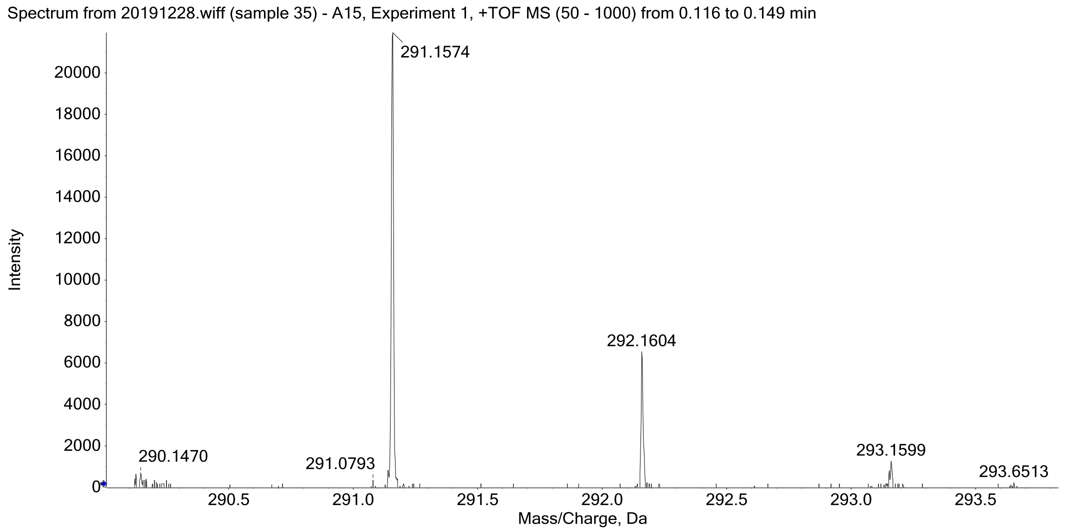

Hit	Formula	m/z	RDB	ppm	MS Rank	MSMS ppm	MSMS Rank	Found
1	SiC20H22	291.1564	11.0	3.6	1			NA/NA

1e

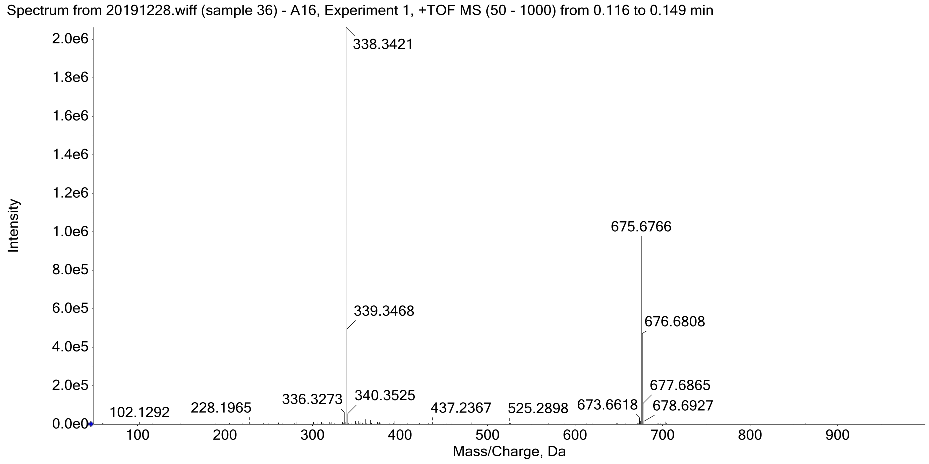

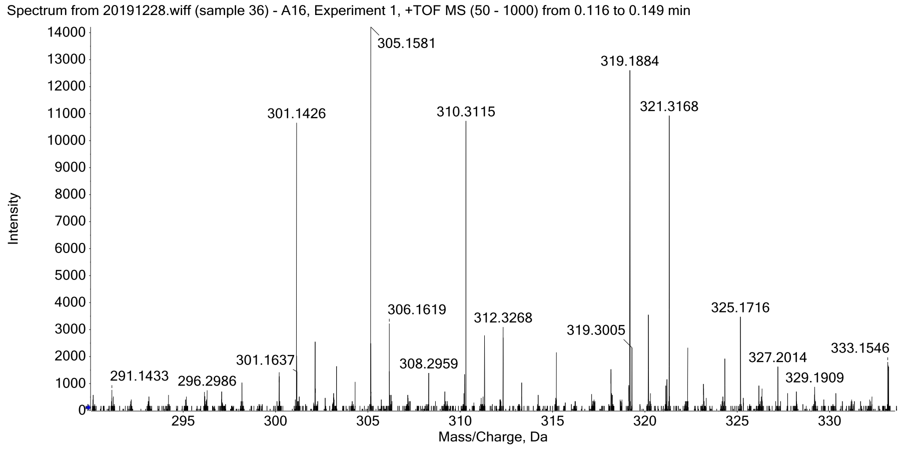

Hit	Formula	m/z	RDB	ppm	MS Rank	MSMS ppm	MSMS Rank	Found
1	SiC22H26	319.1877	11.0	2.3	1			NA/NA

1f

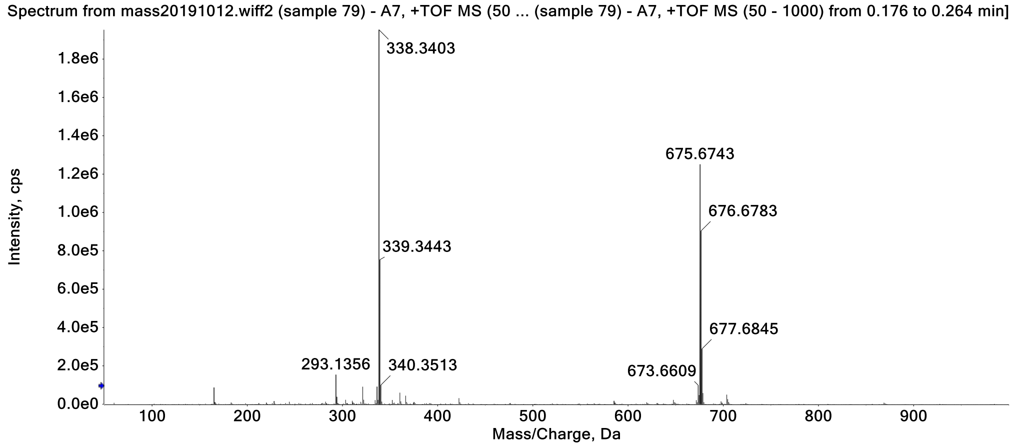

Spectrum from mass20191012.wiff2 (sample 79) - A7, +TOF MS (50 ... (sample 79) - A7, +TOF MS (50 - 1000) from 0.176 to 0.264 min]

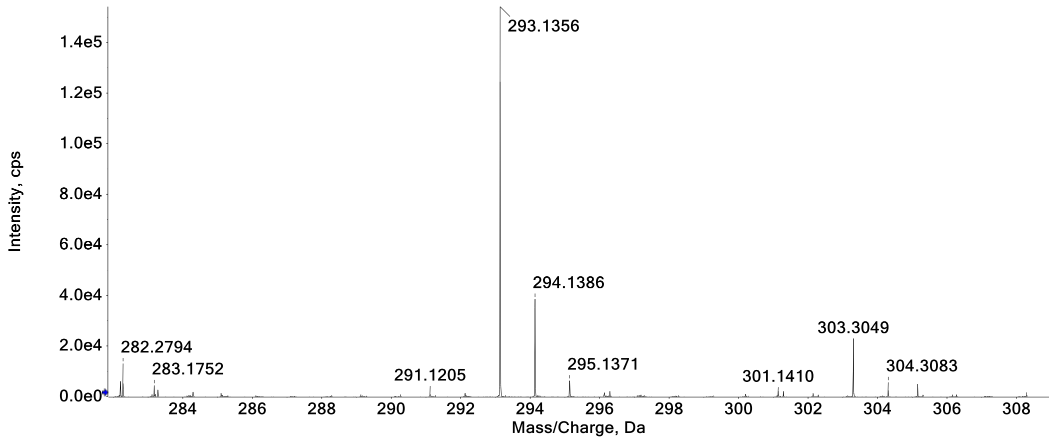

Hit	Formula	m/z	RDB	ppm	MS Rank	MSMS ppm	MSMS Rank	Found
1	C19H20OSi	293.1356	11.0	-0.1	1			NA/NA

1g

Spectrum from mass20191012.wiff2 (sample 77) - A5, +TOF MS (50 - 1000) from 0.079 to 0.140 min

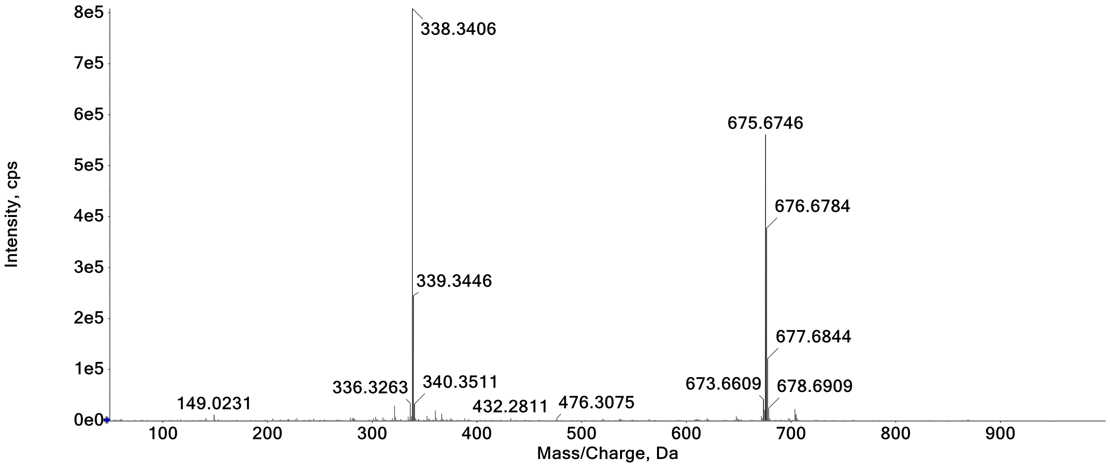

Spectrum from mass20191012.wiff2 (sample 77) - A5, +TOF MS (50 - 1000) from 0.079 to 0.140 min

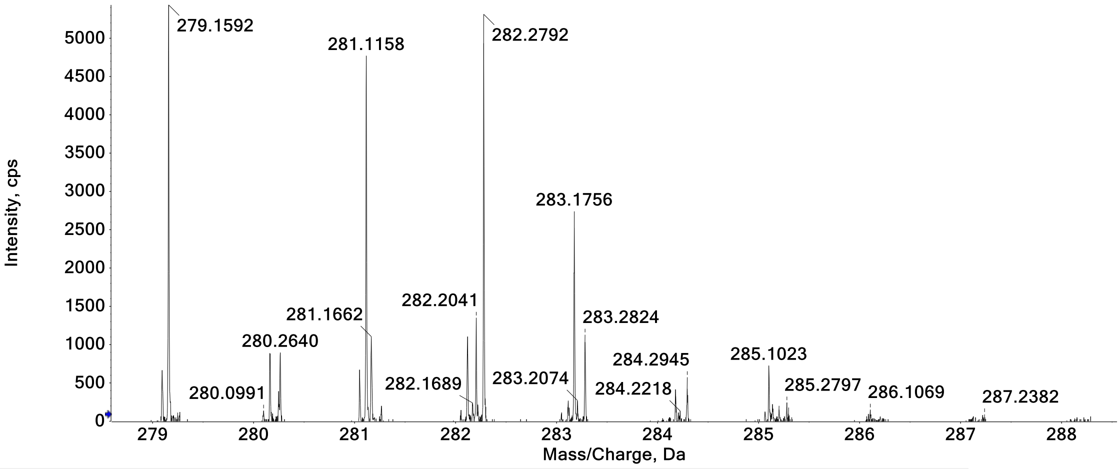

Hit	Formula	m/z	RDB	ppm	MS Rank	MSMS ppm	MSMS Rank	Found
1	C18H17FSi	281.1156	11.0	0.6	1			NA/NA

1h

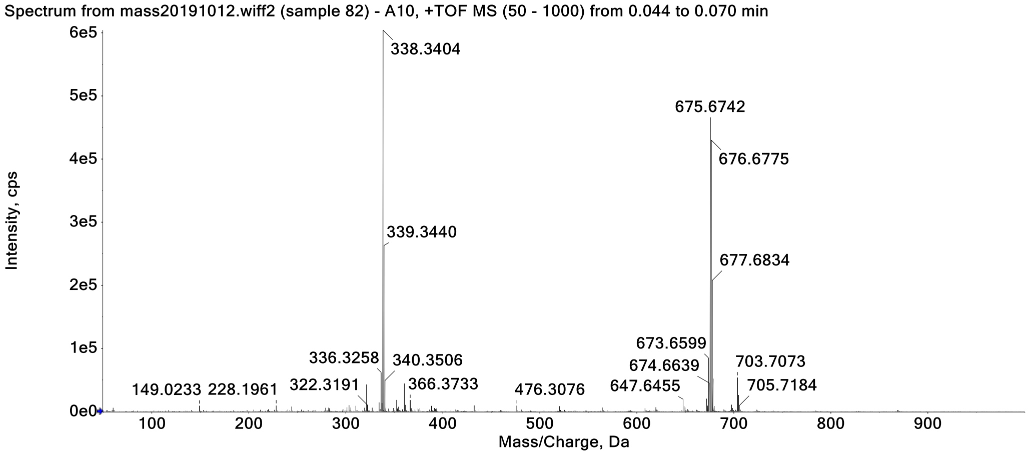

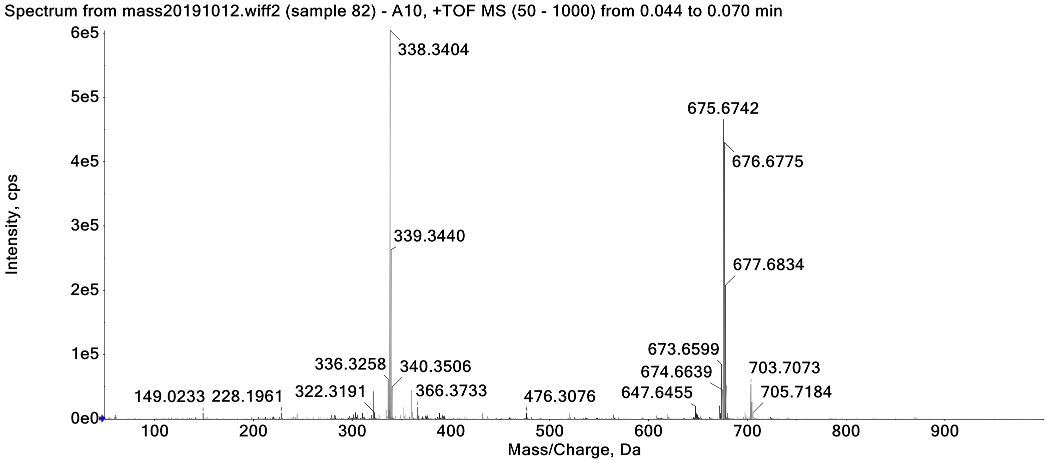

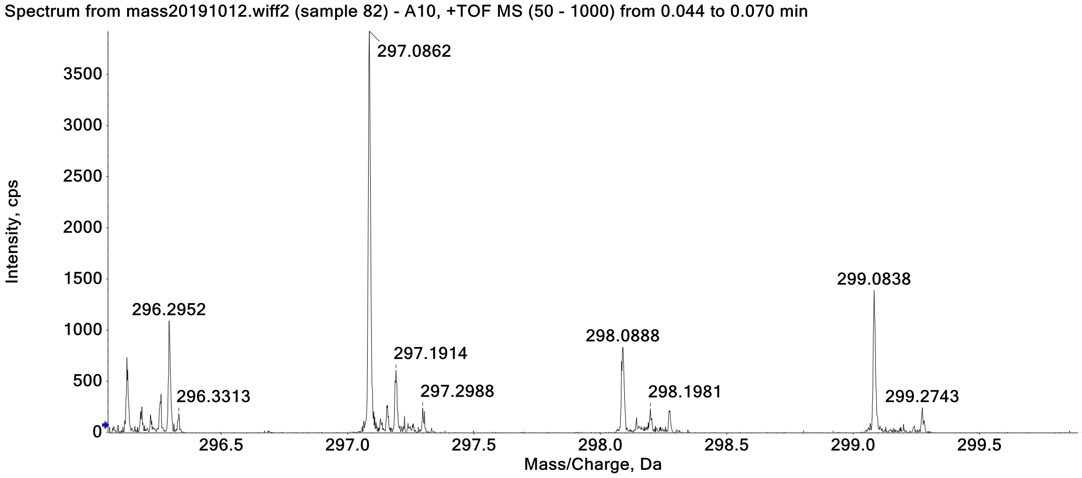

Hit	Formula	m/z	RDB	ppm	MS Rank	MSMS ppm	MSMS Rank	Found
1	C18H17ClSi	297.0861	11.0	0.4	1			NA/NA

1i

Spectrum from mass20191012.wiff2 (sample 83) - A11, +TOF MS (50 - 1000) from 0.044 to 0.070 min

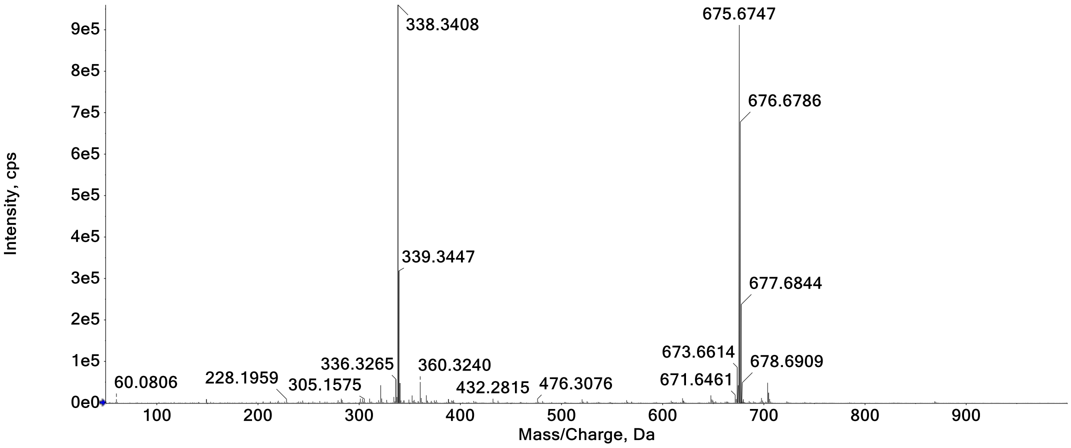

Spectrum from mass20191012.wiff2 (sample 83) - A11, +TOF MS (5...sample 83) - A11, +TOF MS (50 - 1000) from 0.176 to 0.264 min]

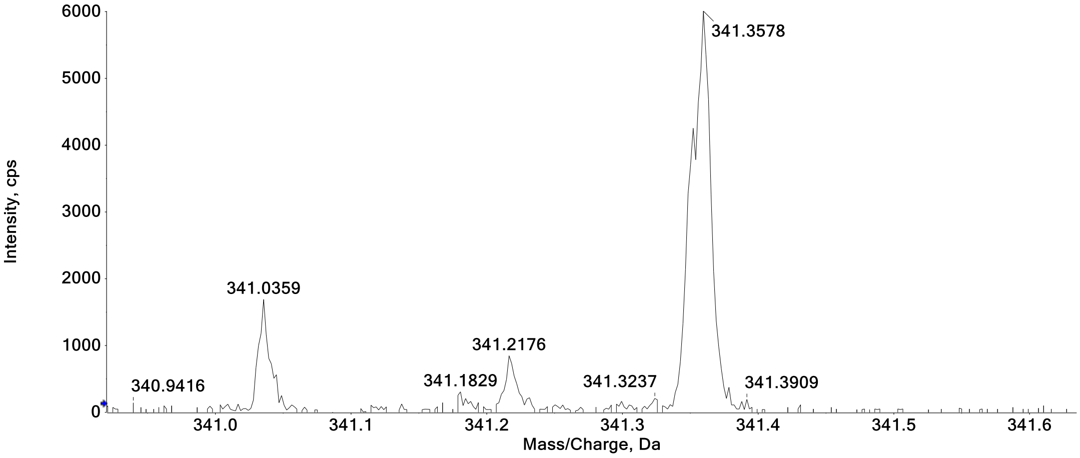

Hit	Formula	m/z	RDB	ppm	MS Rank	MSMS ppm	MSMS Rank	Found
1	C18H17BrSi	341.0356	11.0	1.0	1			NA/NA

1j

Spectrum from 20191228.wiff (sample 34) - A14, Experiment 1, +TOF MS (50 - 1000) from 0.132 to 0.165 min

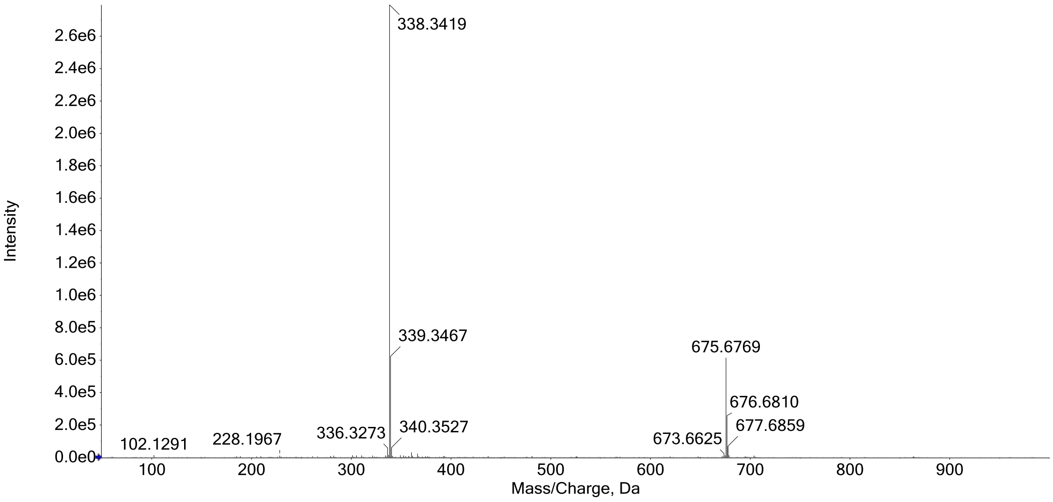

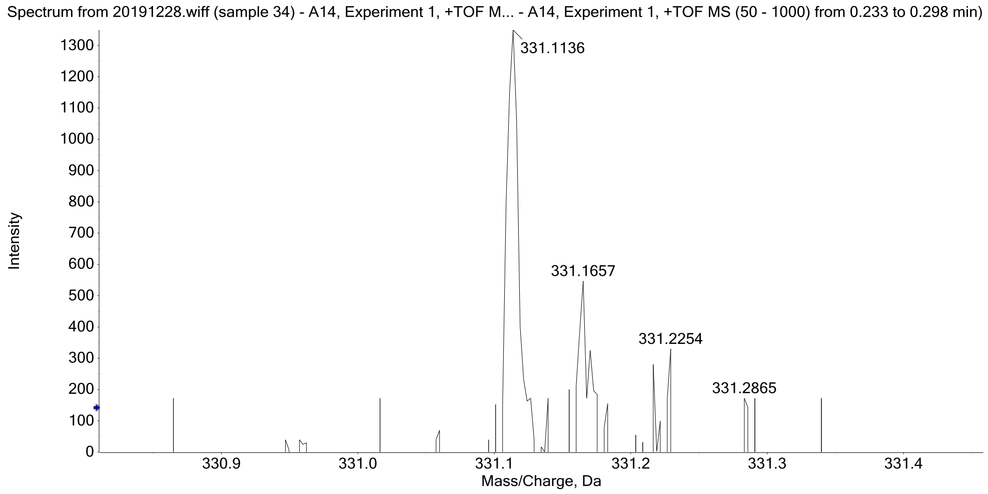

Hit	Formula	m/z	RDB	ppm	MS Rank	MSMS ppm	MSMS Rank	Found
1	SiC19H17F3	331.1124	11.0	3.5	1			NA/NA

1k

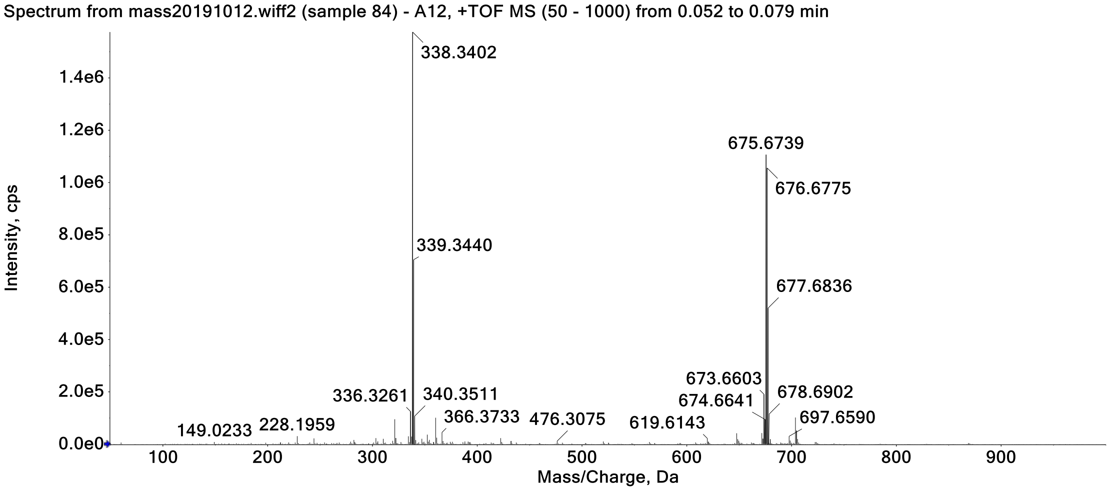

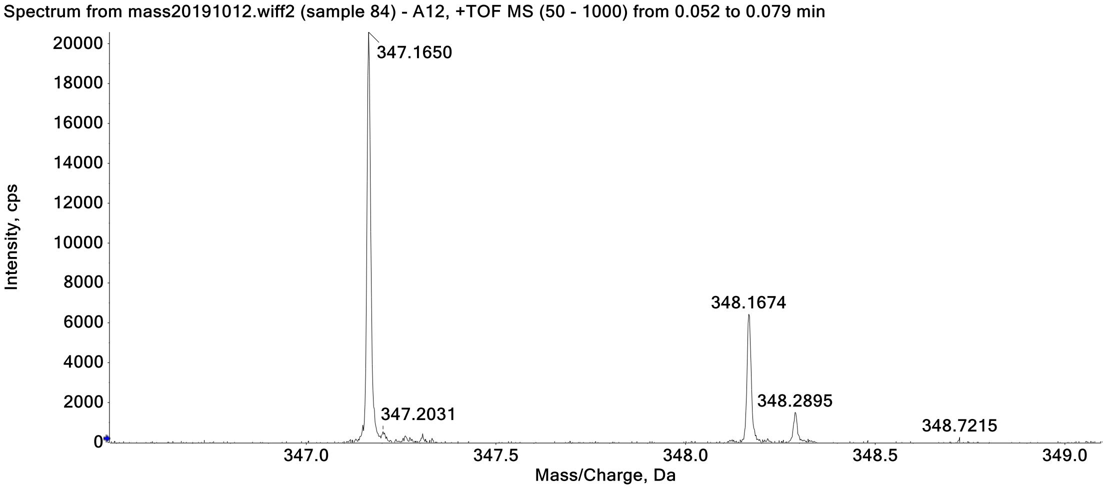

Hit	Formula	m/z	RDB	ppm	MS Rank	MSMS ppm	MSMS Rank	Found
1	C22H26Si2	347.1646	12.0	1.2	1			NA/NA

1l

Spectrum from 20191228.wiff (sample 37) - A17, Experiment 1, +TOF MS (50 - 1000) from 0.116 to 0.149 min

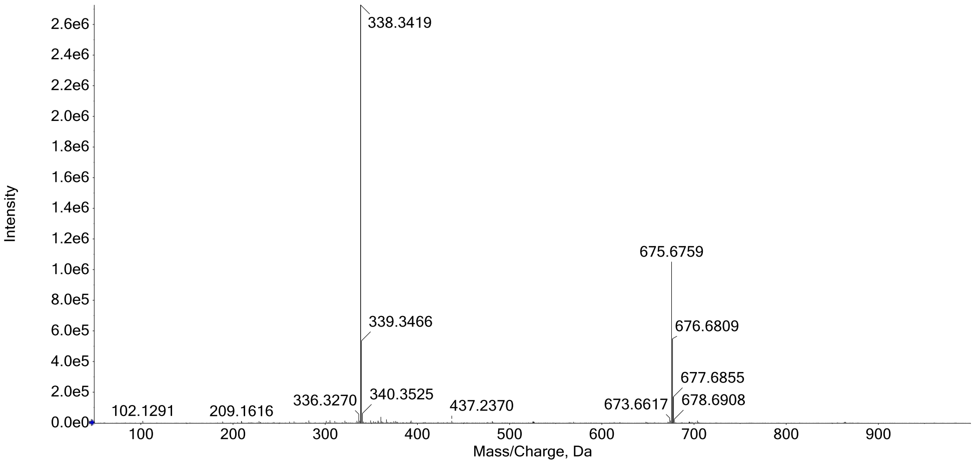

Spectrum from 20191228.wiff (sample 37) - A17, Experiment 1, +TOF MS (50 - 1000) from 0.116 to 0.149 min

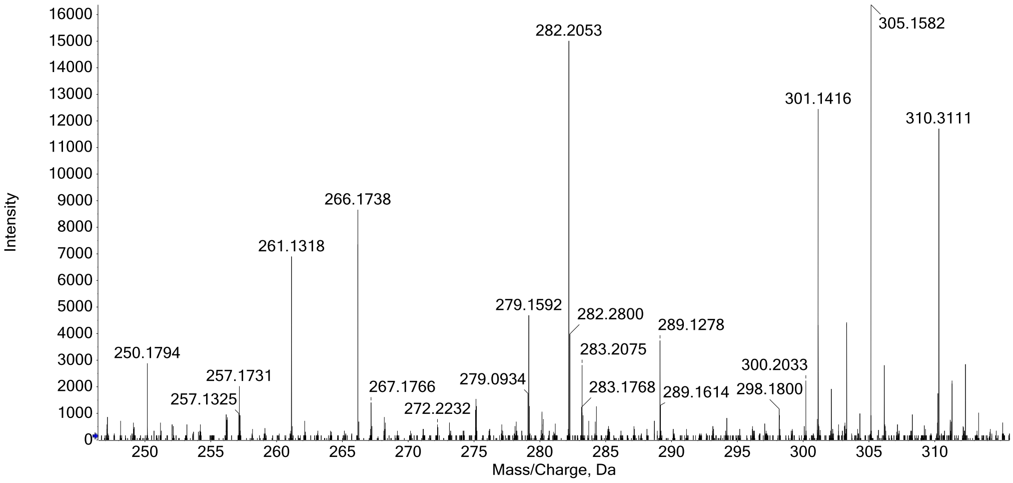

Spectrum from 20191228.wiff (sample 37) - A17, Experiment 1, +TOF MS (50 - 1000) from 0.116 to 0.149 min

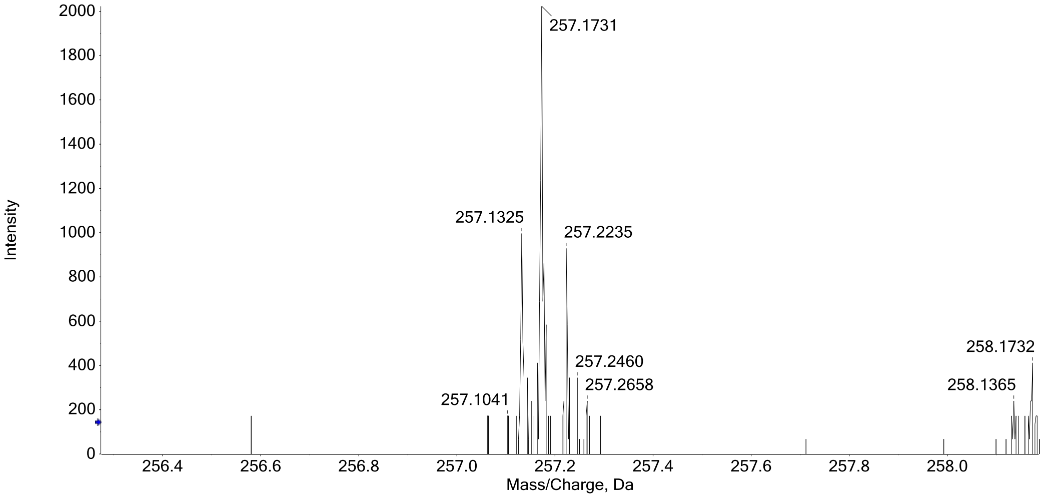

Hit	Formula	m/z	RDB	ppm	MS Rank	MSMS ppm	MSMS Rank	Found
1	SiC17H24	257.1720	7.0	4.3	1			NA/NA

1m

Spectrum from mass20191012.wiff2 (sample 78) - A6, +TOF MS (50 - 1000) from 0.079 to 0.140 min

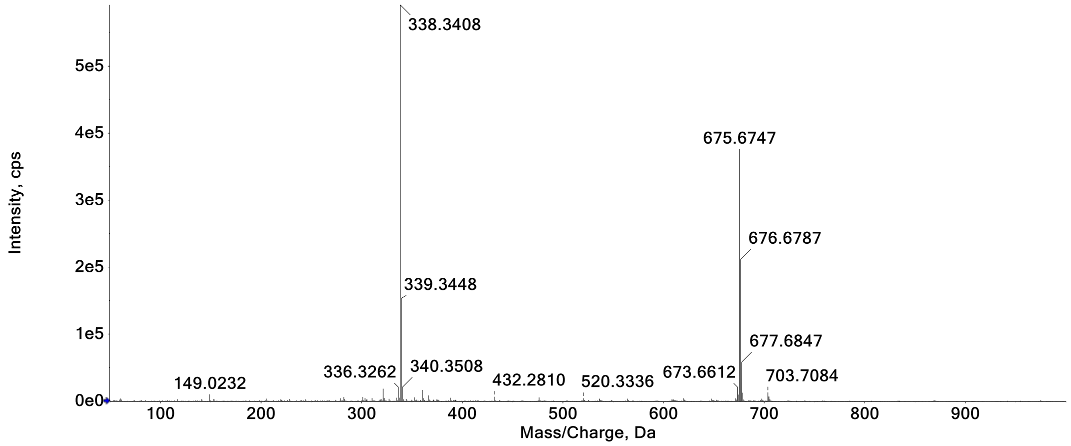

Spectrum from mass20191012.wiff2 (sample 78) - A6, +TOF MS (50 ... (sample 78) - A6, +TOF MS (50 - 1000) from 0.273 to 0.361 min]

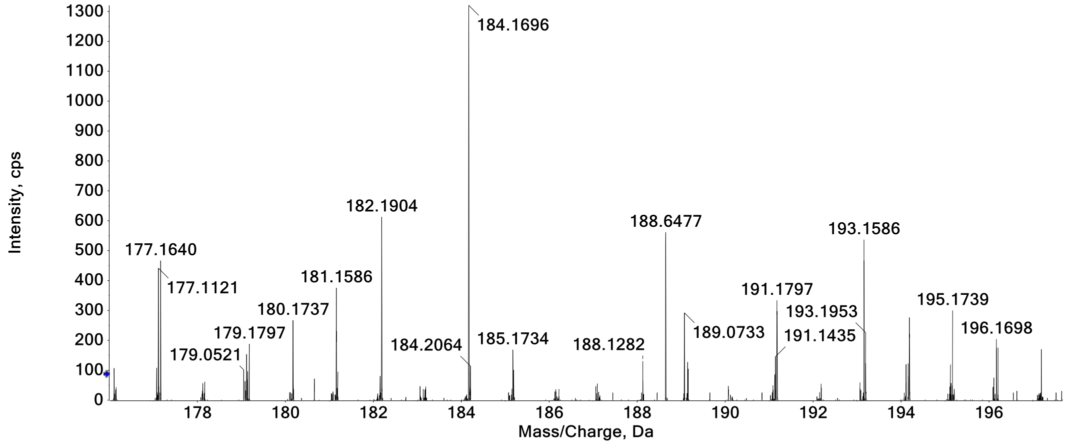

Spectrum from 20191228.wiff (sample 32) - A6, Experiment 1, +TOF MS...) - A6, Experiment 1, +TOF MS (50 - 1000) from 0.233 to 0.298 min)

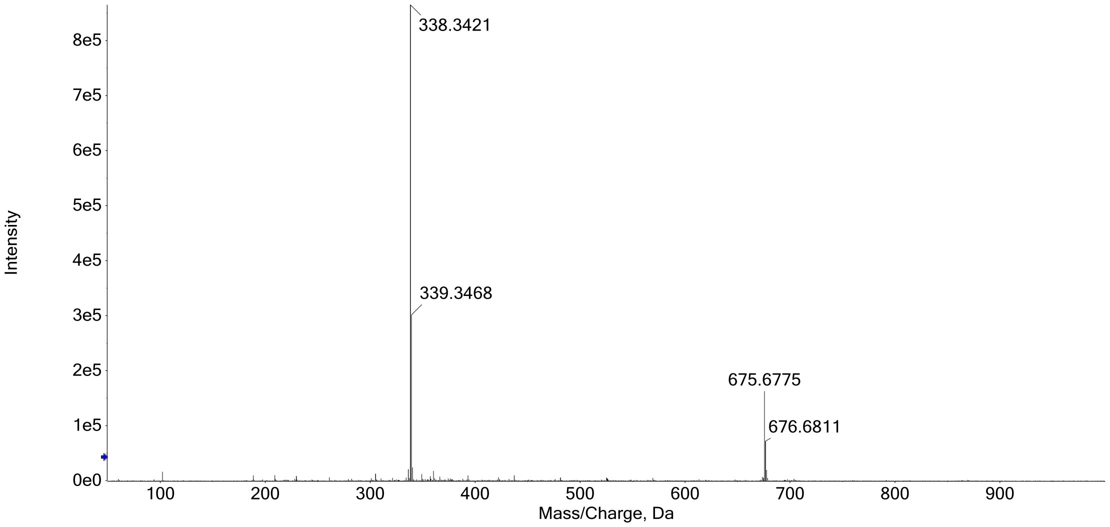

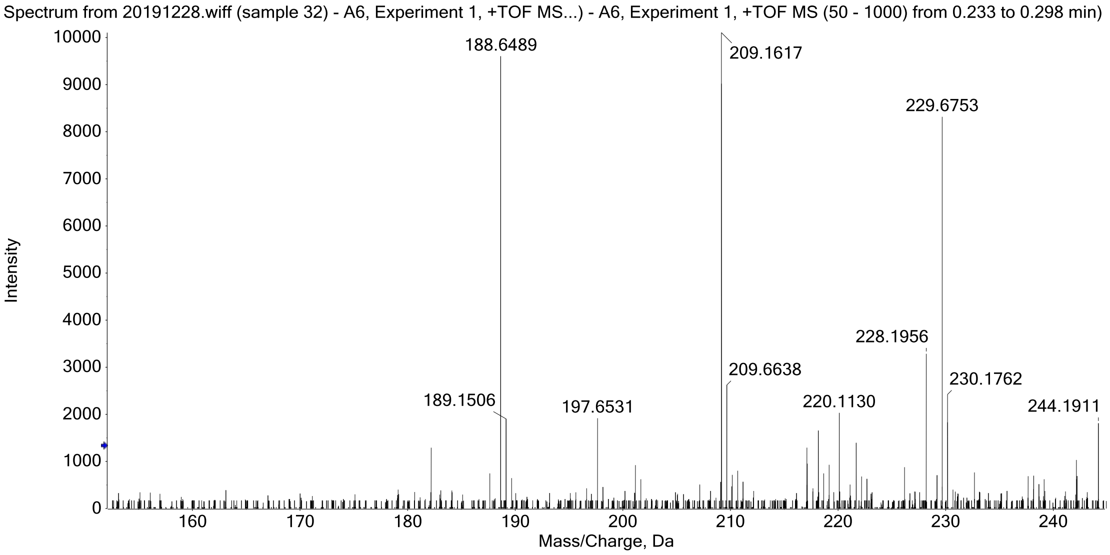
Spectrum from 20191228.wiff (sample 32) - A6, Experiment 1, +TOF MS...) - A6, Experiment 1, +TOF MS (50 - 1000) from 0.233 to 0.298 min)
Intensity
Mass/Charge, Da
188.6489
209.1617
229.6753
228.1956
209.6638
230.1762
189.1506
197.6531
220.1130
244.1911

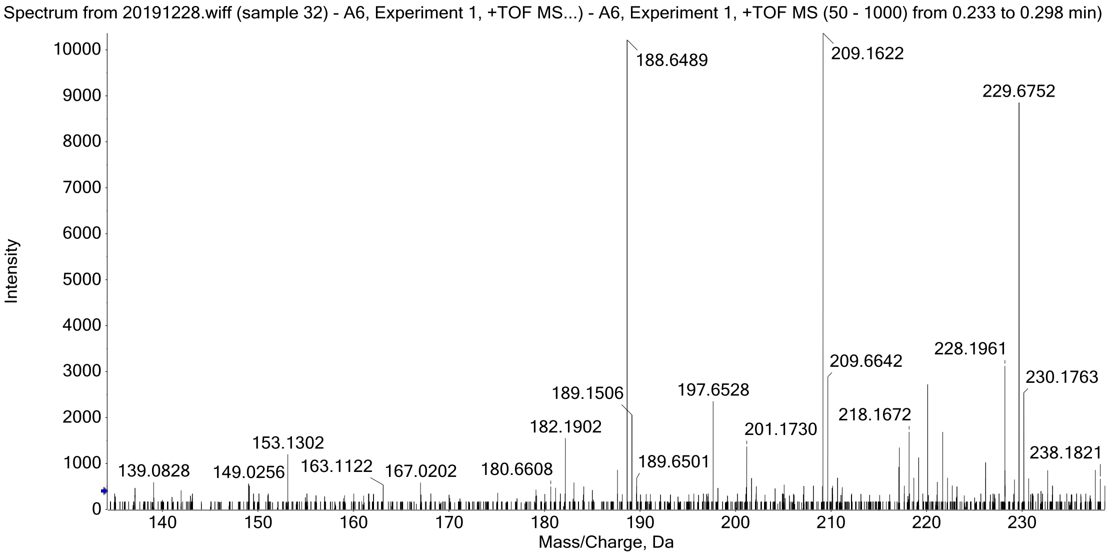
Spectrum from 20191228.wiff (sample 32) - A6, Experiment 1, +TOF MS...) - A6, Experiment 1, +TOF MS (50 - 1000) from 0.233 to 0.298 min)
Intensity
Mass/Charge, Da
188.6489
209.1622
229.6752
228.1961
209.6642
230.1763
189.1506
197.6528
218.1672
182.1902
201.1730
153.1302
238.1821
139.0828
149.0256
163.1122
167.0202
180.6608
189.6501

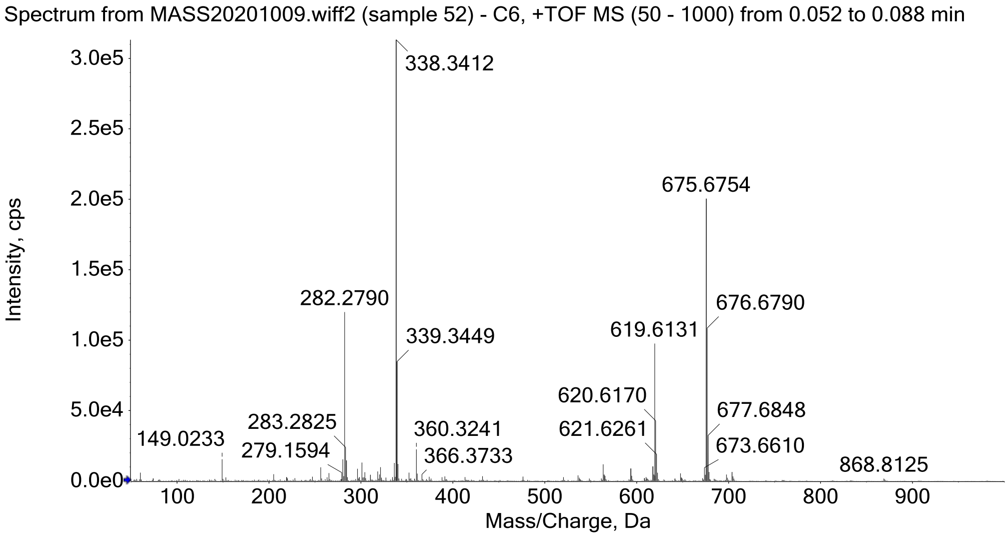
Spectrum from MASS20201009.wiff2 (sample 52) - C6, +TOF MS (50 - 1000) from 0.052 to 0.088 min
Intensity, cps
Mass/Charge, Da
338.3412
675.6754
282.2790
676.6790
619.6131
339.3449
620.6170
677.6848
283.2825
149.0233
360.3241
621.6261
673.6610
279.1594
366.3733
868.8125

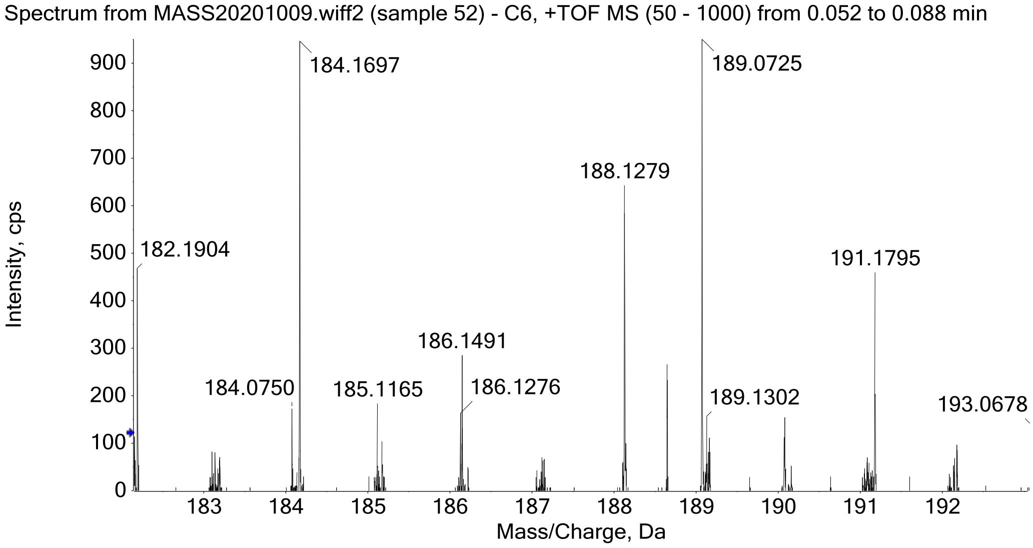

Spectrum from MASS20201009.wiff2 (sample 52) - C6, +TOF MS (50 - 1000) from 0.052 to 0.088 min
Intensity, cps
Mass/Charge, Da
182.1904
184.0750
184.1697
185.1165
186.1491
186.1276
188.1279
189.0725
189.1302
191.1795
193.0678

1n

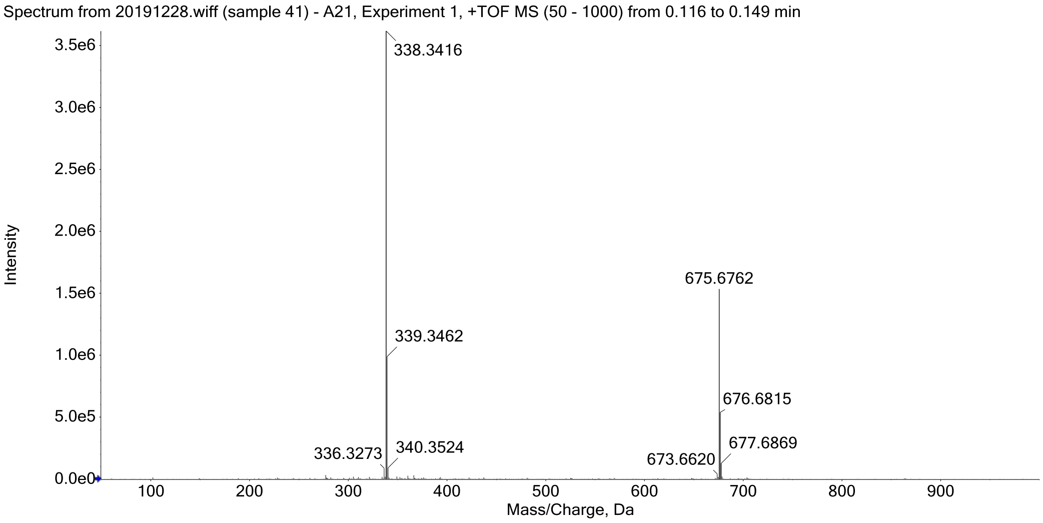

Spectrum from 20191228.wiff (sample 41) - A21, Experiment 1, +TOF MS (50 - 1000) from 0.116 to 0.149 min
Intensity
Mass/Charge, Da
338.3416
339.3462
336.3273
340.3524
675.6762
676.6815
673.6620
677.6869

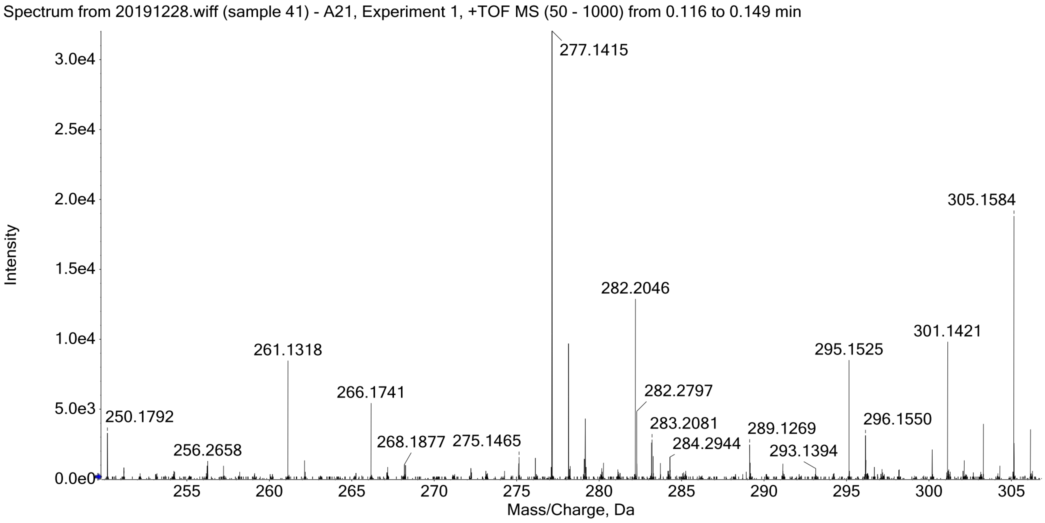

Spectrum from 20191228.wiff (sample 41) - A21, Experiment 1, +TOF MS (50 - 1000) from 0.116 to 0.149 min
Intensity
Mass/Charge, Da
277.1415
305.1584
282.2046
261.1318
295.1525
301.1421
266.1741
282.2797
250.1792
283.2081
289.1269
296.1550
284.2944
256.2658
268.1877
275.1465
293.1394

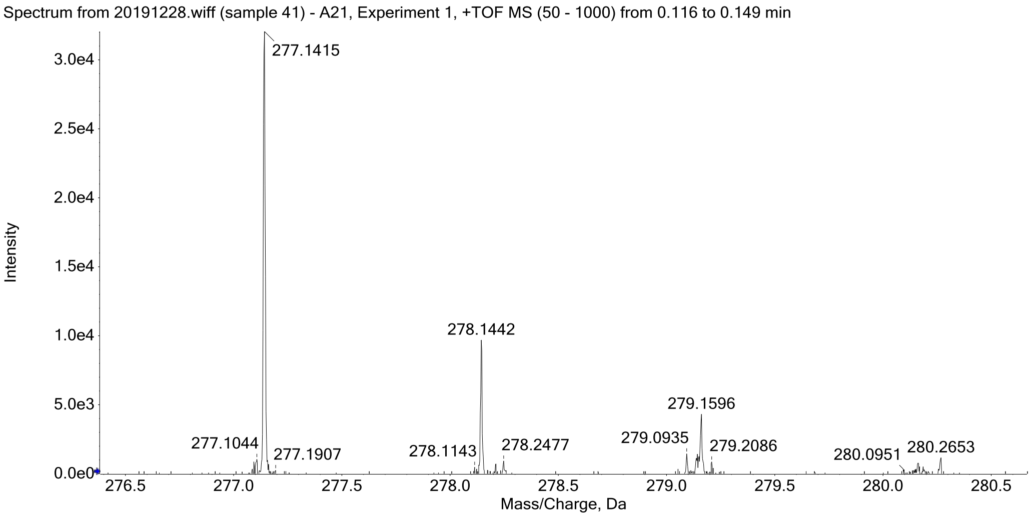

Hit	Formula	m/z	RDB	ppm	MS Rank	MSMS ppm	MSMS Rank	Found
1	SiC19H20	277.1407	11.0	2.9	1			NA/NA

1o

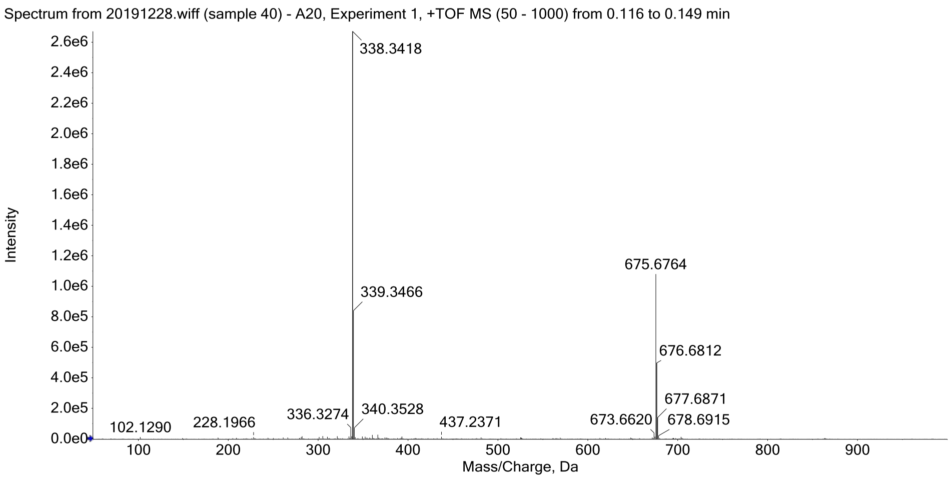

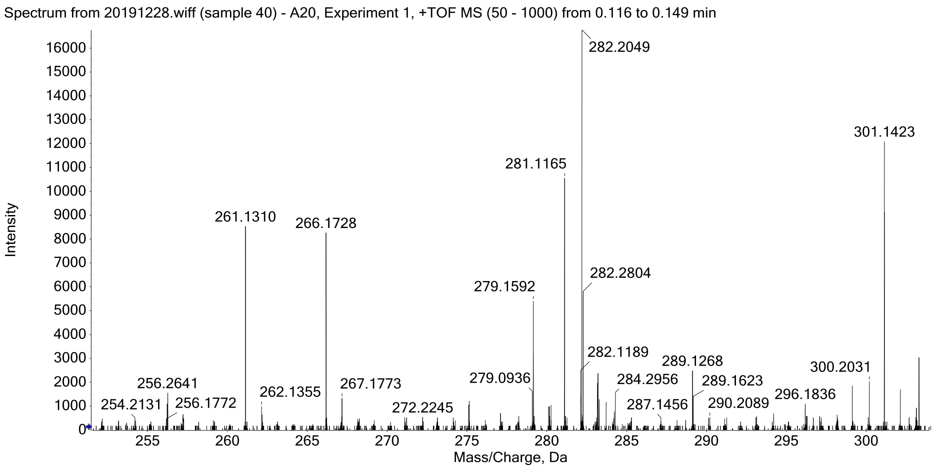

Hit	Formula	m/z	RDB	ppm	MS Rank	MSMS ppm	MSMS Rank	Found
1	SiC18H17F	281.1156	11.0	3.1	1			NA/NA

1p

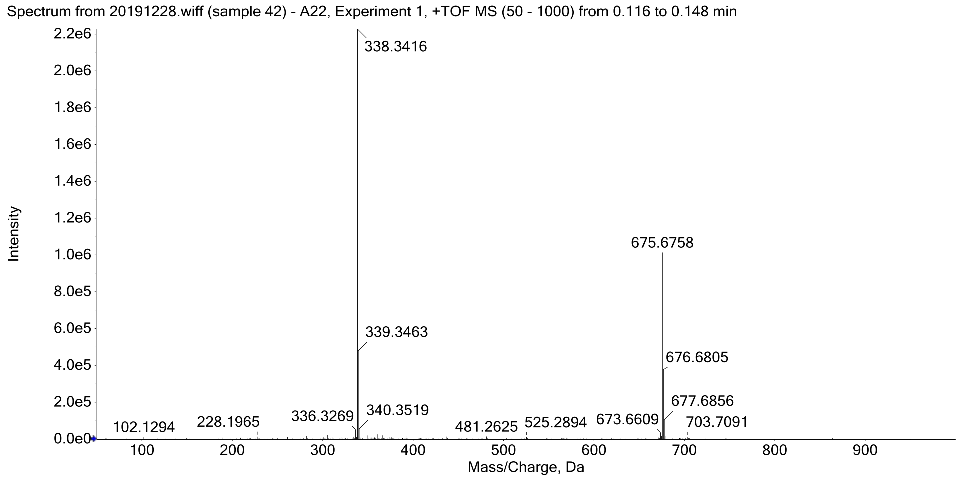

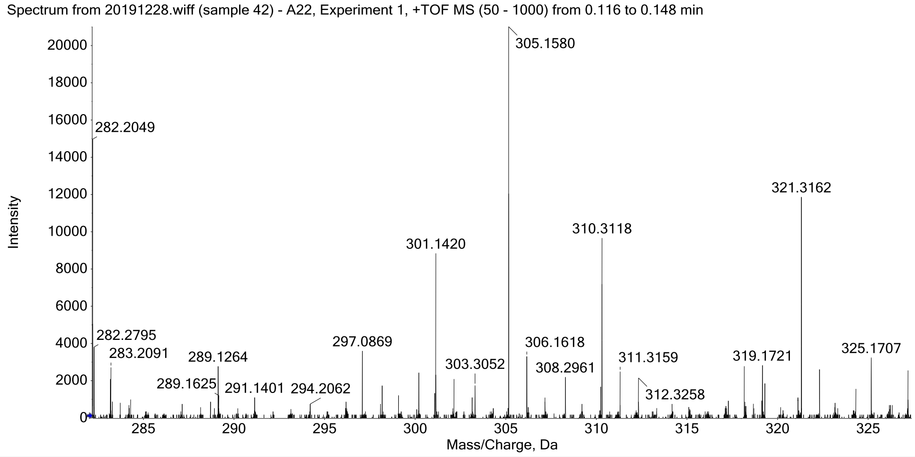

Hit	Formula	m/z	RDB	ppm	MS Rank	MSMS ppm	MSMS Rank	Found
1	SiC18H17Cl	297.0861	11.0	2.7	1			NA/NA

1q

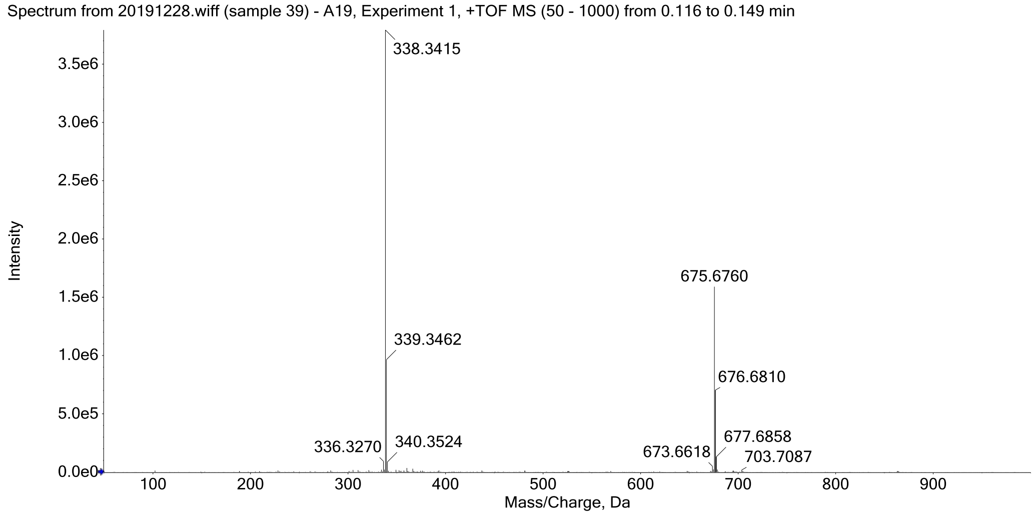

Spectrum from 20191228.wiff (sample 39) - A19, Experiment 1, +TOF MS (50 - 1000) from 0.116 to 0.149 min

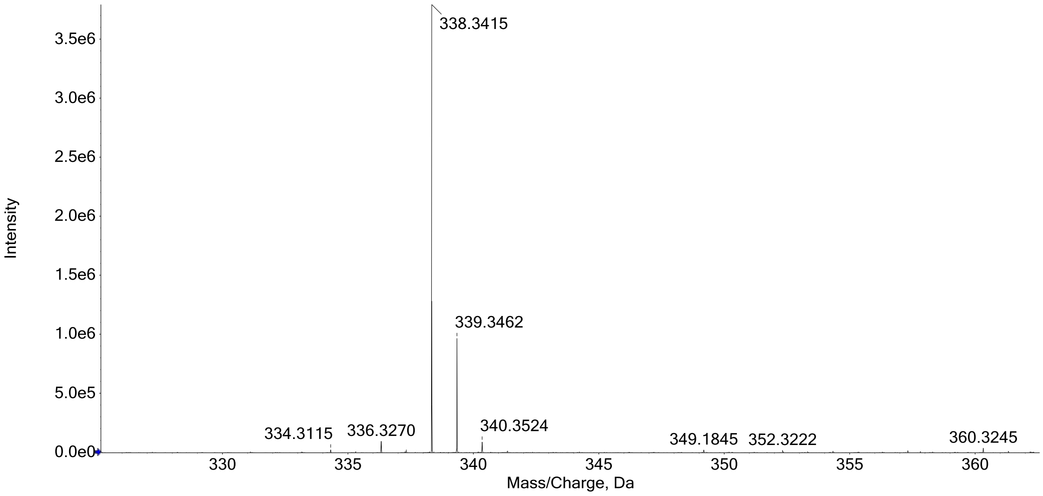

Spectrum from 20191228.wiff (sample 39) - A19, Experiment 1, +TOF MS (50 - 1000) from 0.116 to 0.149 min

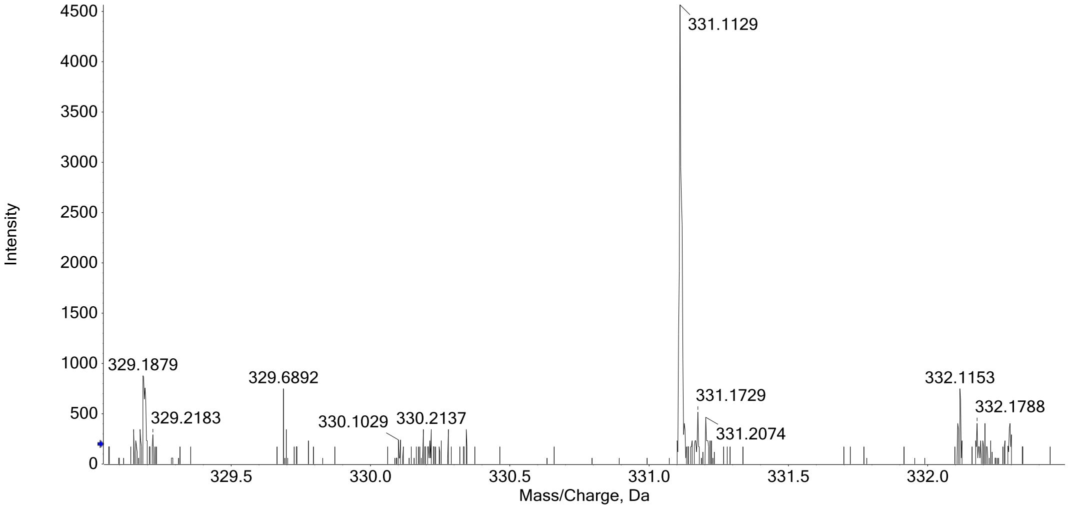

Hit	Formula	m/z	RDB	ppm	MS Rank	MSMS ppm	MSMS Rank	Found
1	SiC19H17F3	331.1124	11.0	1.4	1			NA/NA

4

Spectrum from mass20191012.wiff2 (sample 80) - A8, +TOF MS (50 - 1000) from 0.052 to 0.079 min

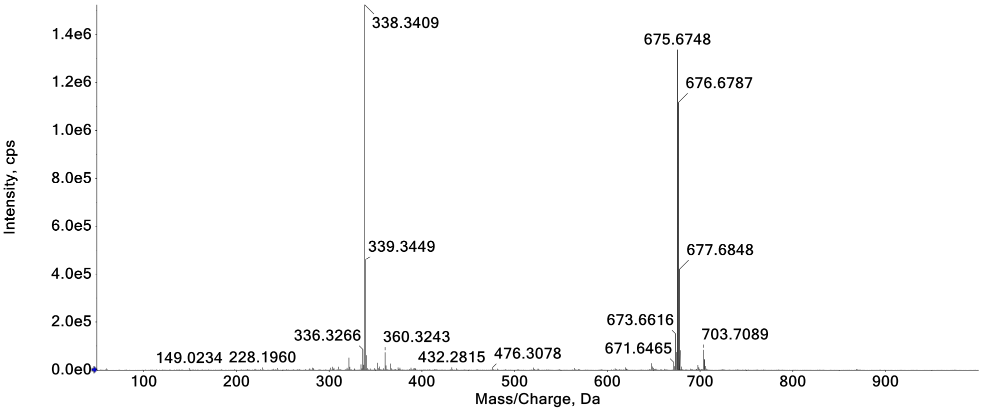

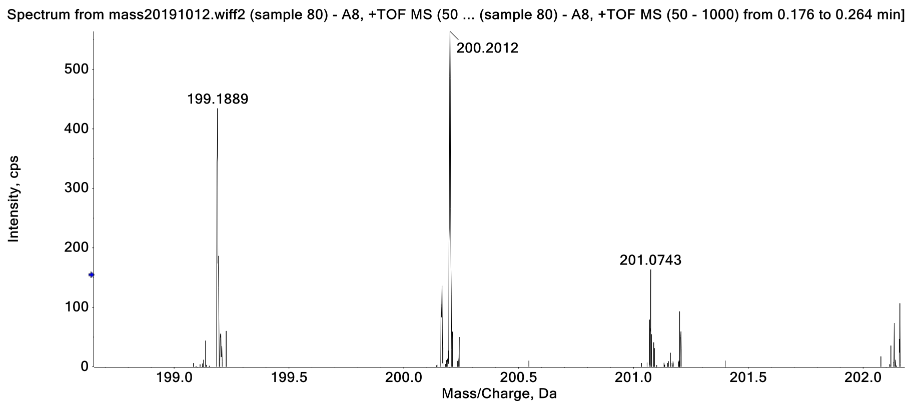
Spectrum from mass20191012.wiff2 (sample 80) - A8, +TOF MS (50 ... (sample 80) - A8, +TOF MS (50 - 1000) from 0.176 to 0.264 min]
200.2012
199.1889
201.0743
Intensity, cps
Mass/Charge, Da

1r

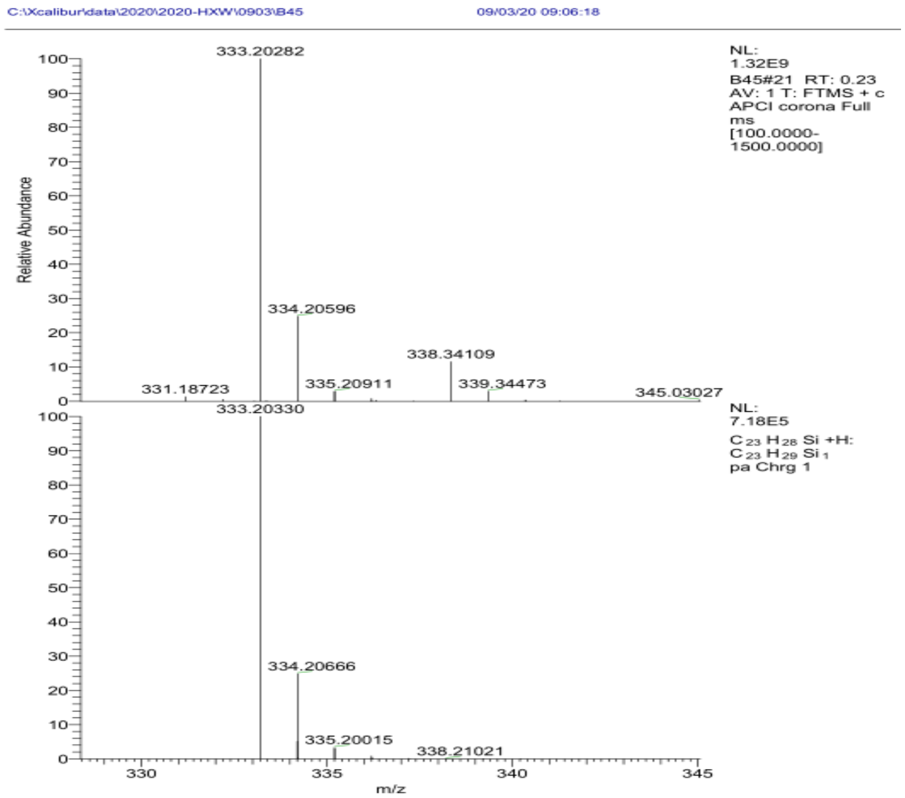
C:\Xcalibur\data\2020\2020-HXW\0903\B45
09/03/20 09:06:18
333.20282
NL: 1.32E9
B45#21 RT: 0.23
AV: 1 T: FTMS + c APCI corona Full ms [100.0000-1500.0000]
334.20596
338.34109
331.18723
335.20911
339.34473
345.03027
333.20330
NL: 7.18E5
C 23 H 28 Si +H: C 23 H 29 Si 1 pa Chrg 1
334.20666
335.20015
338.21021
Relative Abundance
m/z

3a

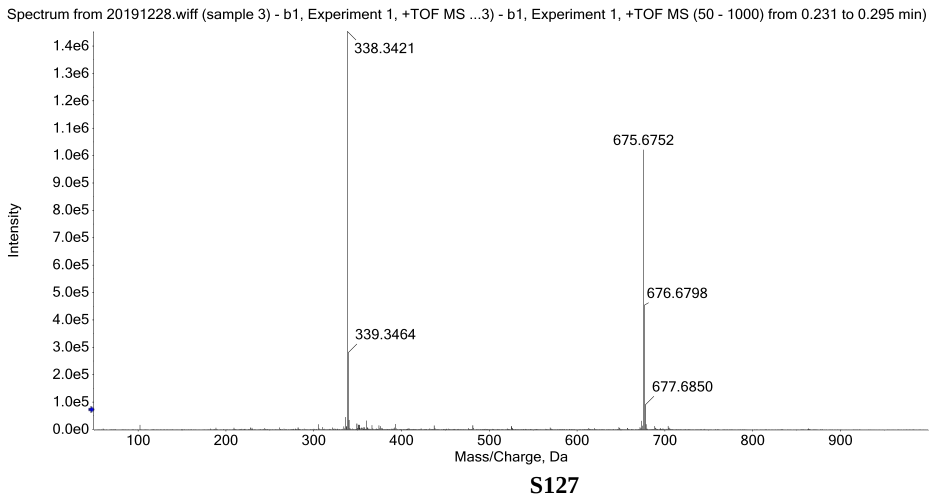
Spectrum from 20191228.wiff (sample 3) - b1, Experiment 1, +TOF MS ...3) - b1, Experiment 1, +TOF MS (50 - 1000) from 0.231 to 0.295 min)
338.3421
675.6752
676.6798
339.3464
677.6850
Intensity
Mass/Charge, Da

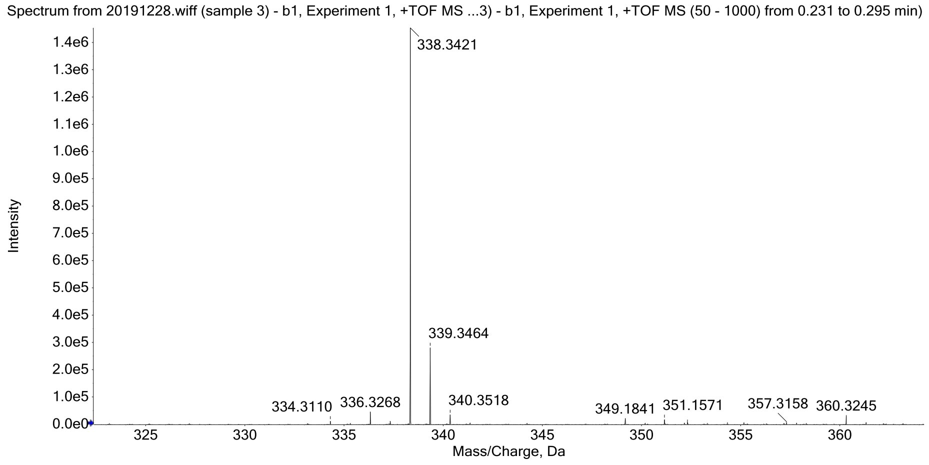

Hit	Formula	m/z	RDB	ppm	MS Rank	MSMS ppm	MSMS Rank	Found
1	SiC25H22	351.1564	16.0	2.1	1			NA/NA

3b

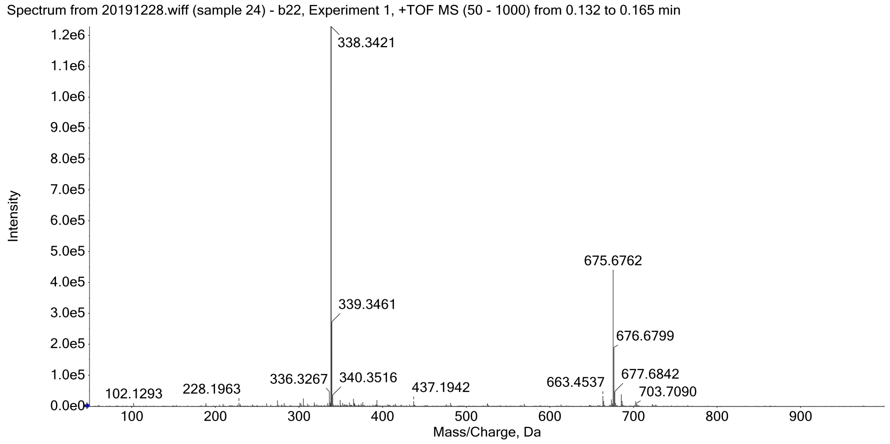

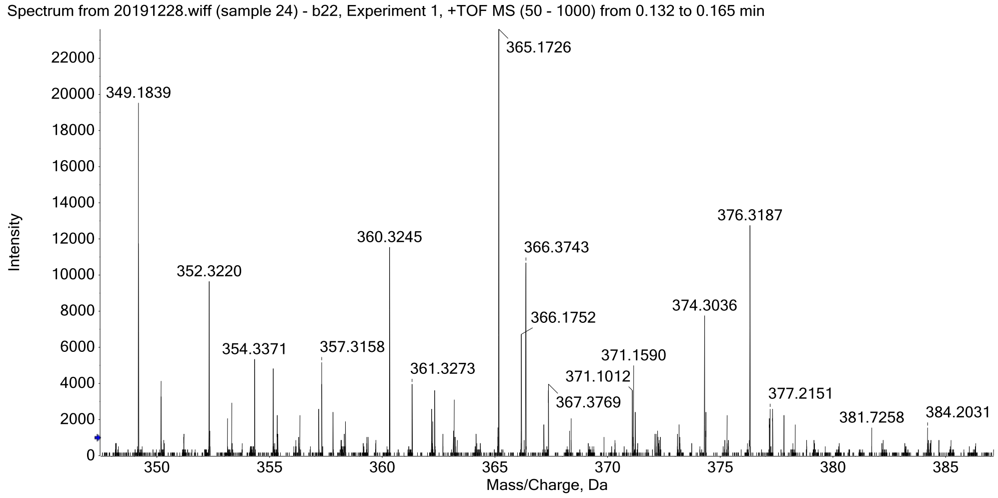

Hit	Formula	m/z	RDB	ppm	MS Rank	MSMS ppm	MSMS Rank	Found
1	SiC26H24	365.1720	16.0	1.6	1			NA/NA

3c

Spectrum from 20191228.wiff (sample 25) - b23, Experiment 1, +TOF MS (50 - 1000) from 0.133 to 0.165 min

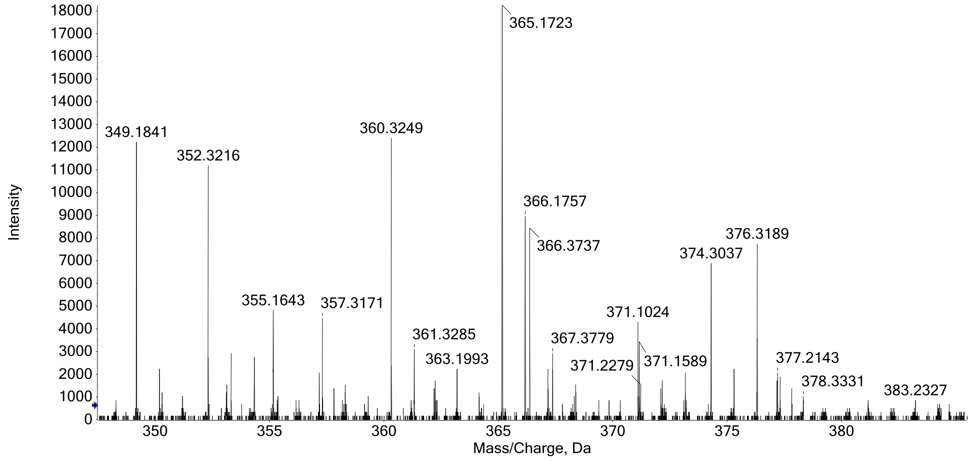

Spectrum from 20191228.wiff (sample 25) - b23, Experiment 1, +TOF MS (50 - 1000) from 0.133 to 0.165 min

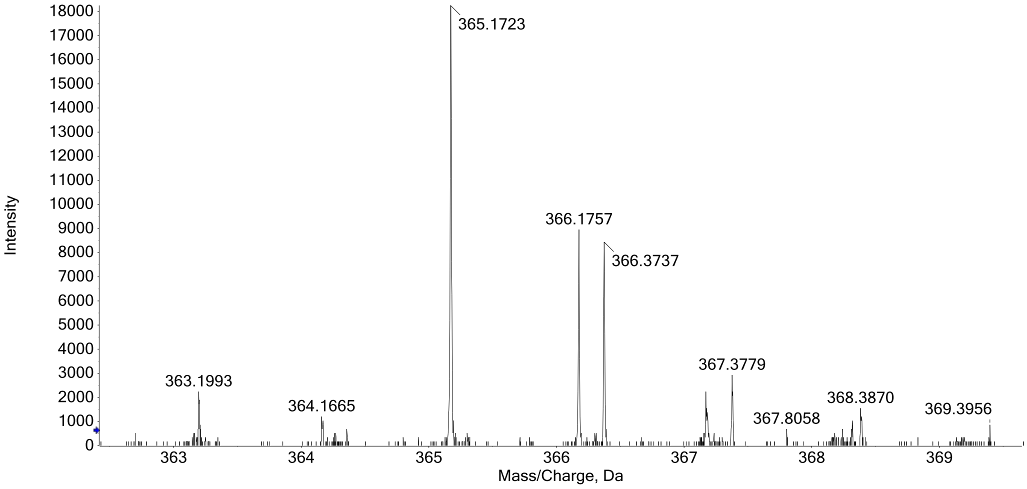

Spectrum from 20191228.wiff (sample 25) - b23, Experiment 1, +TOF MS (50 - 1000) from 0.133 to 0.165 min

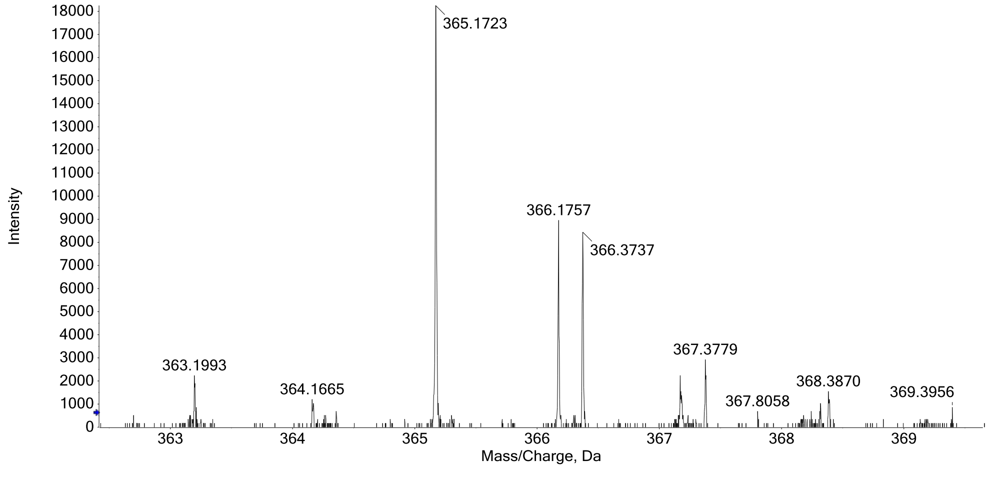

Spectrum from 20191228.wiff (sample 25) - b23, Experiment 1, +TOF MS (50 - 1000) from 0.133 to 0.165 min

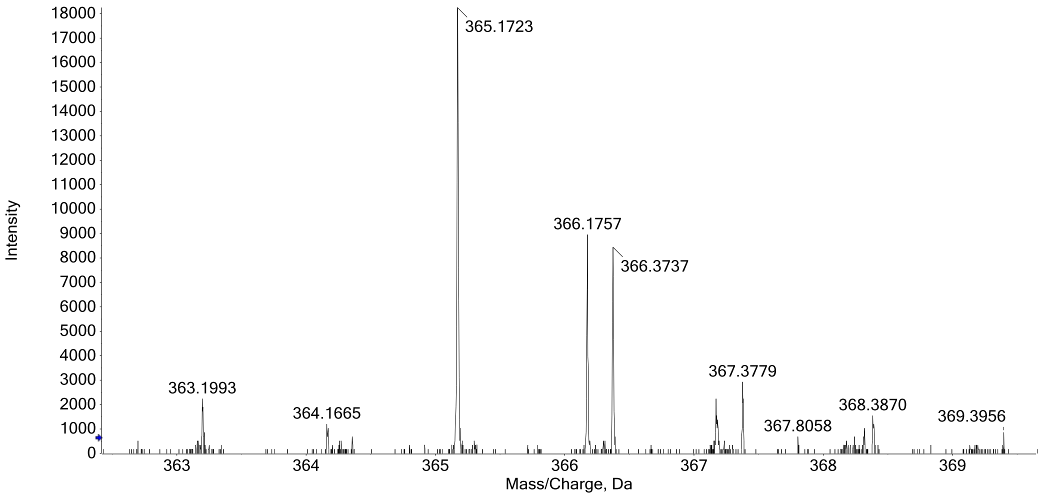

Spectrum from 20191228.wiff (sample 25) - b23, Experiment 1, +TOF MS (50 - 1000) from 0.133 to 0.165 min

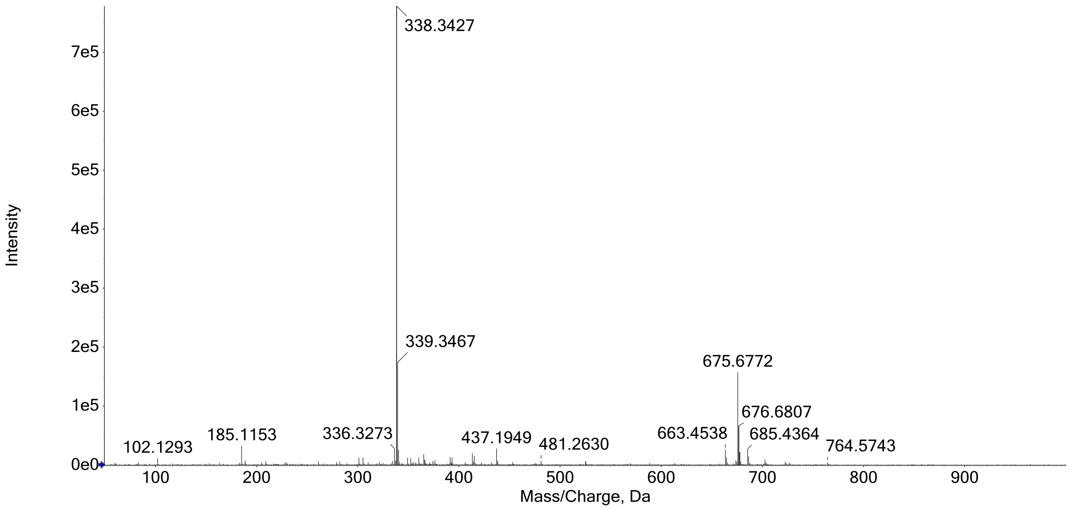

Spectrum from 20191228.wiff (sample 25) - b23, Experiment 1, +TOF MS (50 - 1000) from 0.133 to 0.165 min

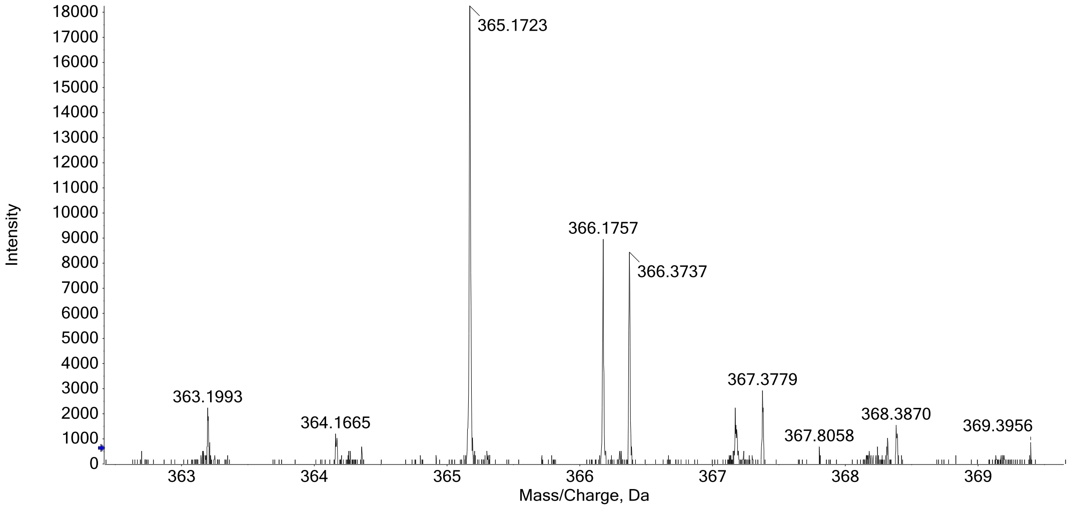

Hit	Formula	m/z	RDB	ppm	MS Rank	MSMS ppm	MSMS Rank	Found
1	SiC26H24	365.1720	16.0	0.8	1			NA/NA

3d

Spectrum from 20191228.wiff (sample 17) - b15, Experiment 1, +TOF MS (50 - 1000) from 0.132 to 0.165 min

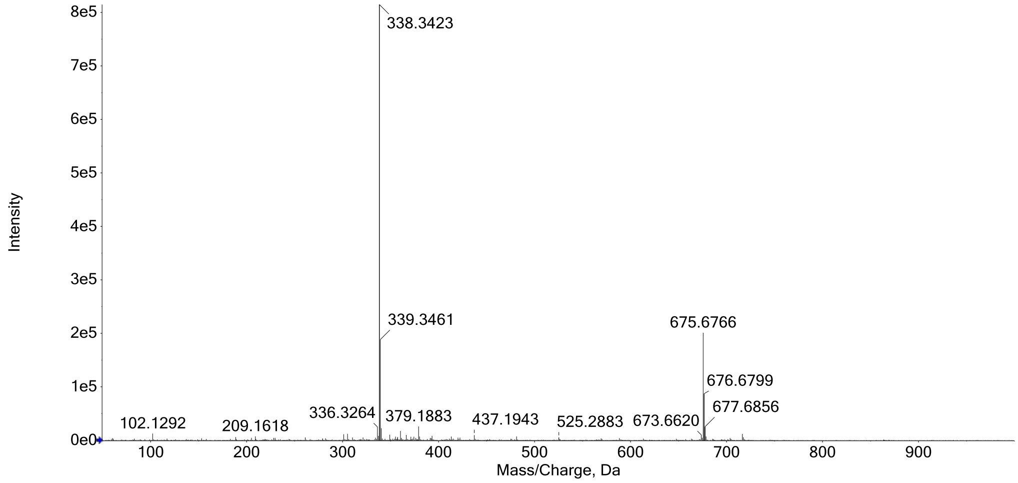

Spectrum from 20191228.wiff (sample 17) - b15, Experiment 1, +TOF MS (50 - 1000) from 0.132 to 0.165 min

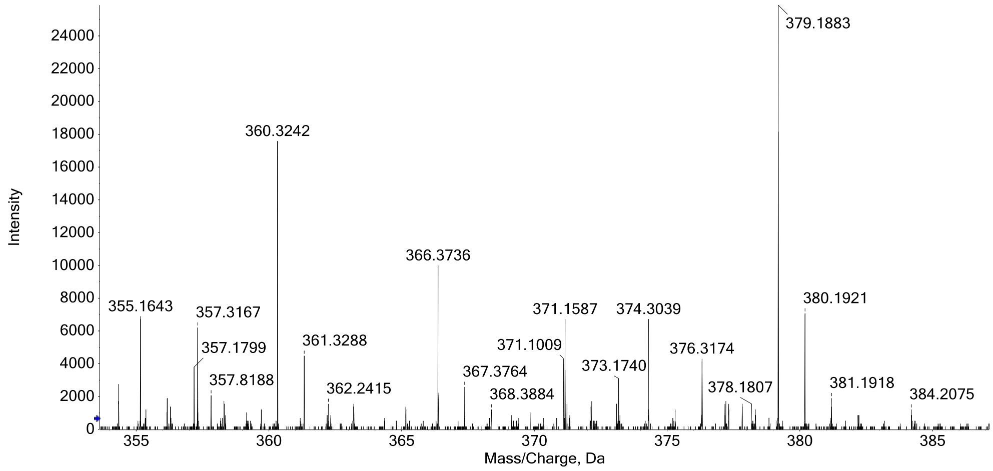

Hit	Formula	m/z	RDB	ppm	MS Rank	MSMS ppm	MSMS Rank	Found
1	SiC27H26	379.1877	16.0	1.7	1			NA/NA

3e

Spectrum from 20191228.wiff (sample 16) - b14, Experiment 1, +TOF M... - b14, Experiment 1, +TOF MS (50 - 1000) from 0.231 to 0.295 min)

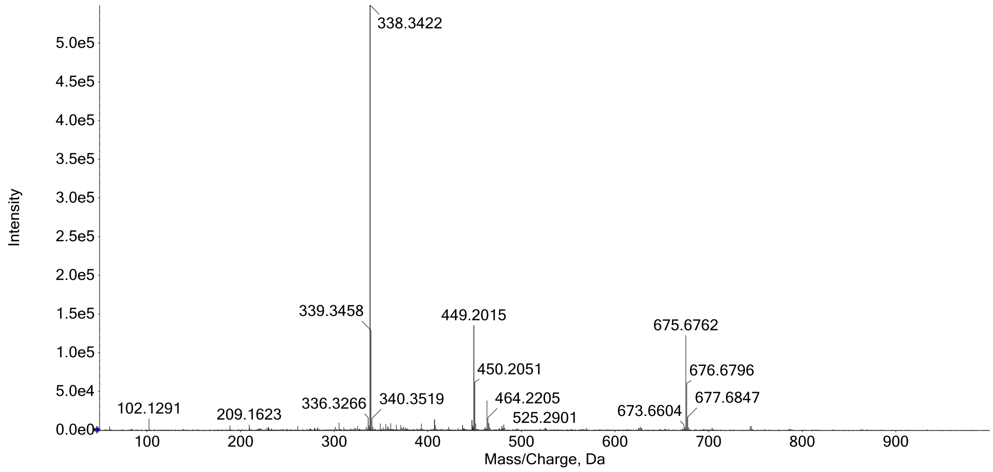

Spectrum from 20191228.wiff (sample 16) - b14, Experiment 1, +TOF M... - b14, Experiment 1, +TOF MS (50 - 1000) from 0.231 to 0.295 min)

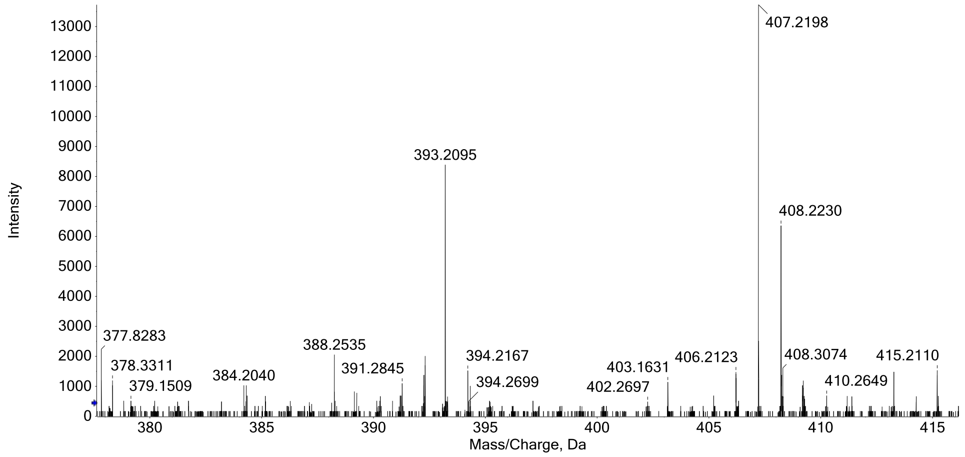

Hit	Formula	m/z	RDB	ppm	MS Rank	MSMS ppm	MSMS Rank	Found
1	SiC29H30	407.2190	16.0	2.1	1			NA/NA

3f

Spectrum from 20191228.wiff (sample 18) - b16, Experiment 1, +TOF MS (50 - 1000) from 0.132 to 0.165 min

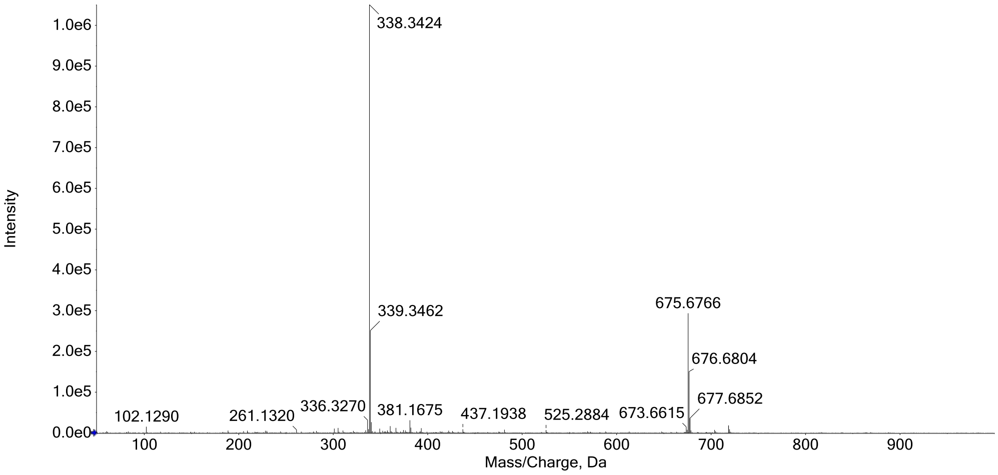

Spectrum from 20191228.wiff (sample 18) - b16, Experiment 1, +TOF MS (50 - 1000) from 0.132 to 0.165 min

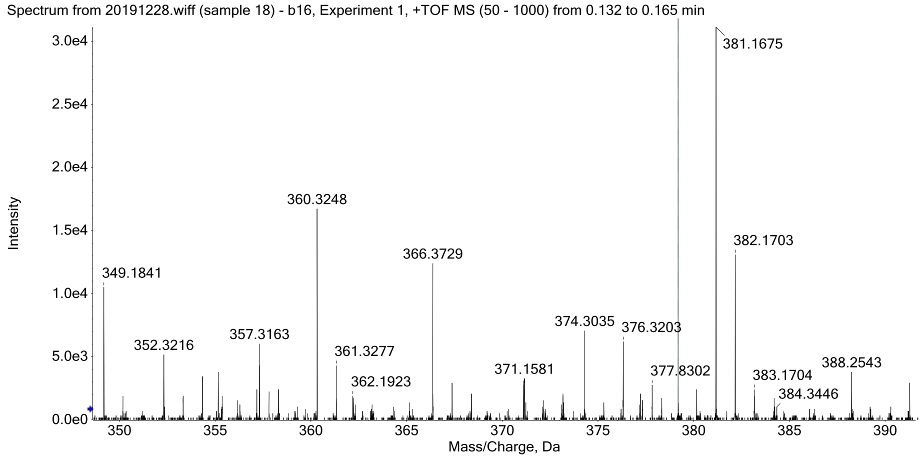

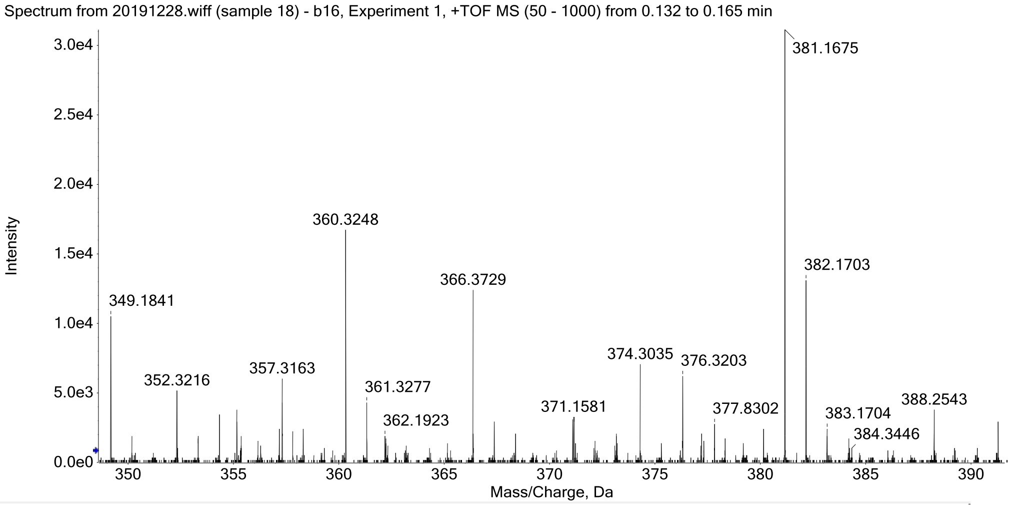

Hit	Formula	m/z	RDB	ppm	MS Rank	MSMS ppm	MSMS Rank	Found
1	SiC26H24O	381.1669	16.0	1.5	1			NA/NA

3g

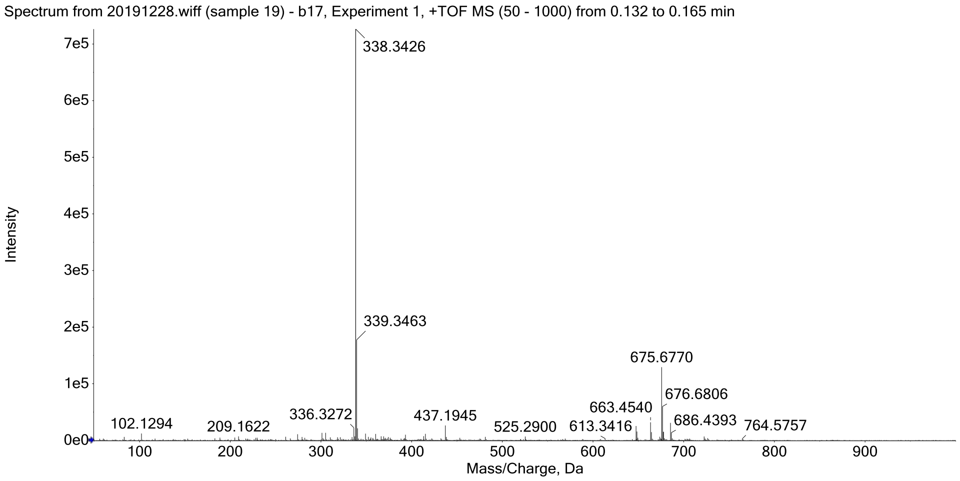

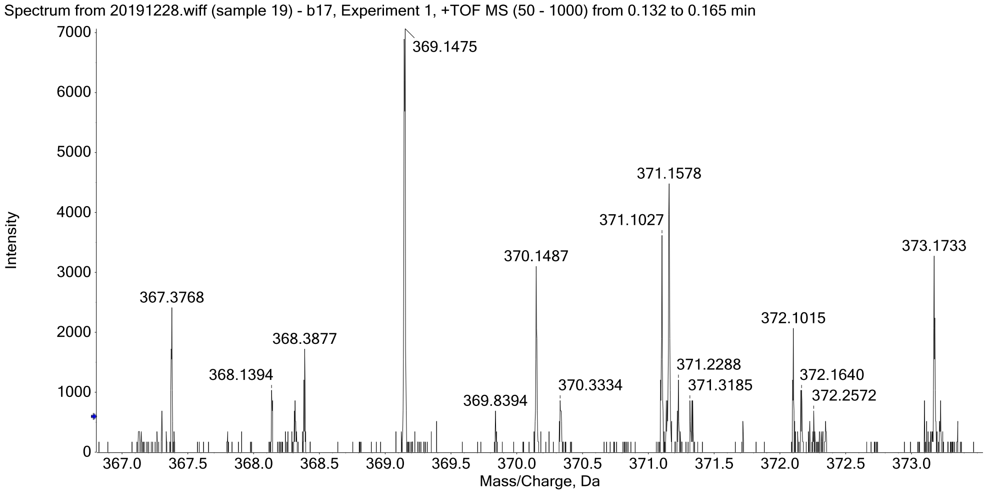

Hit	Formula	m/z	RDB	ppm	MS Rank	MSMS ppm	MSMS Rank	Found
1	SiC25H21F	369.1469	16.0	1.5	1			NA/NA

3h

Spectrum from 20191228.wiff (sample 20) - b18, Experiment 1, +TOF MS (50 - 1000) from 0.132 to 0.164 min

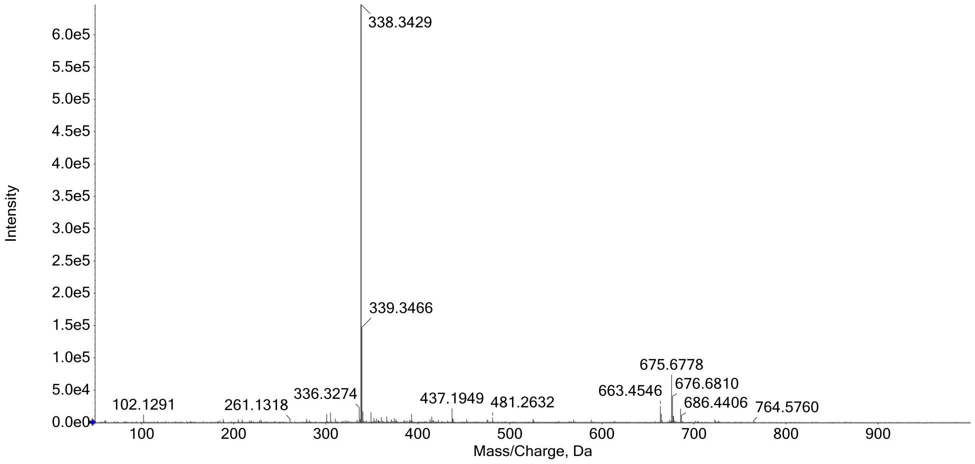

Spectrum from 20191228.wiff (sample 20) - b18, Experiment 1, +TOF MS (50 - 1000) from 0.132 to 0.164 min

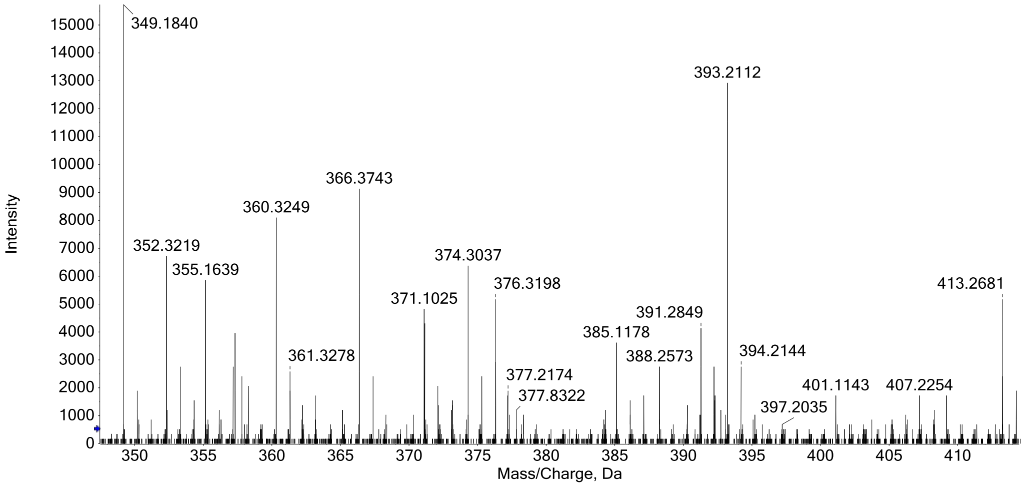

Hit	Formula	m/z	RDB	ppm	MS Rank	MSMS ppm	MSMS Rank	Found
1	SiC25H21Cl	385.1174	16.0	1.1	1			NA/NA

3i

Spectrum from 20191228.wiff (sample 21) - b19, Experiment 1, +TOF MS (50 - 1000) from 0.132 to 0.164 min

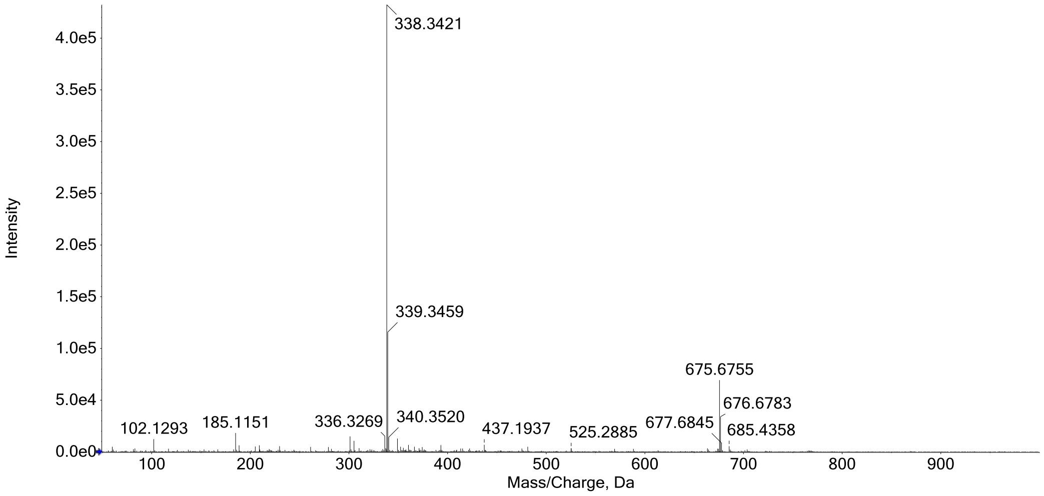

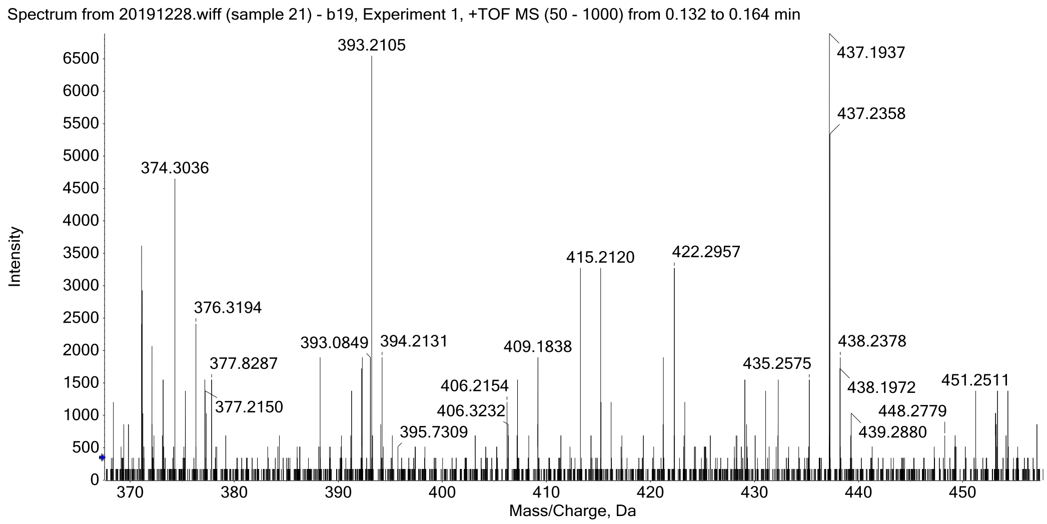

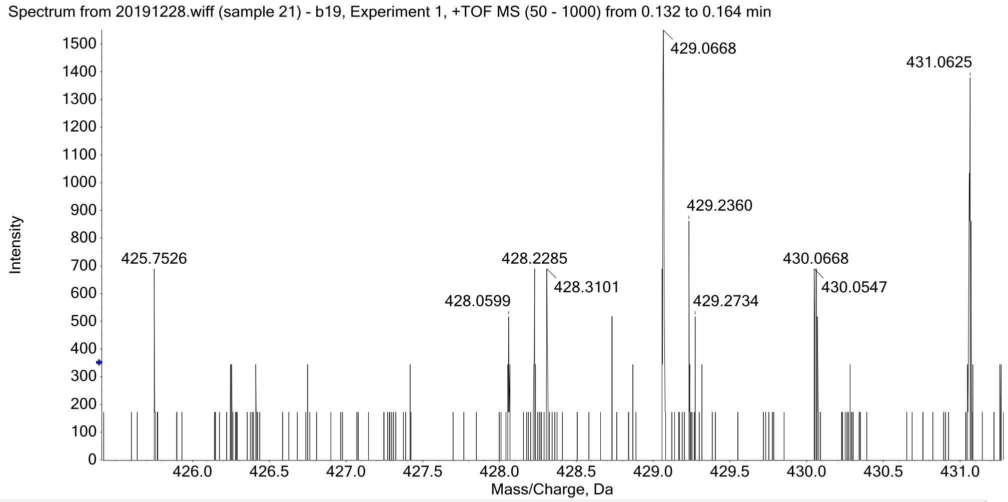

Hit	Formula	m/z	RDB	ppm	MS Rank	MSMS ppm	MSMS Rank	Found
1	SiC25H21Br	429.0669	16.0	-0.2	1			NA/NA

3j

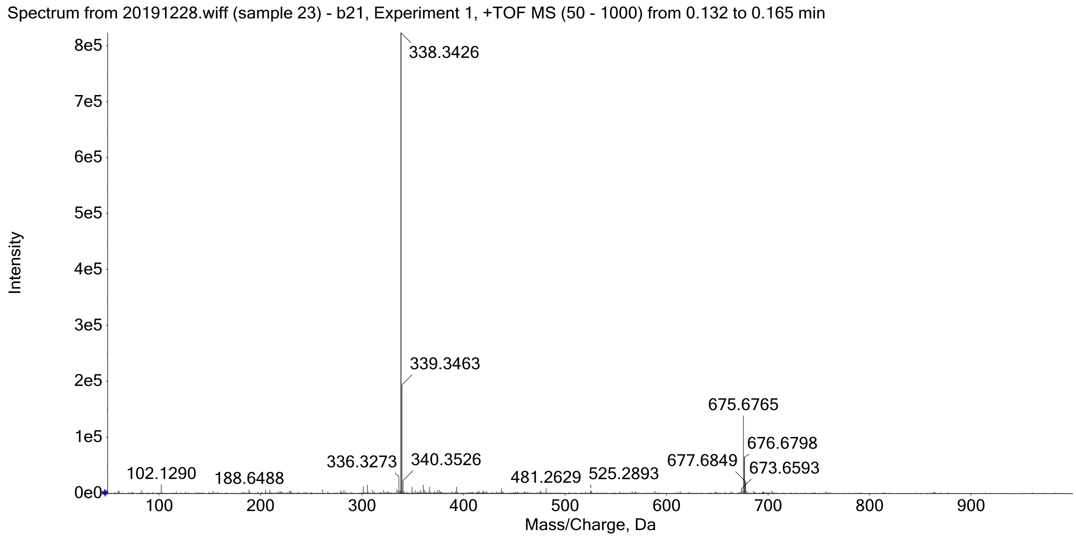

Spectrum from 20191228.wiff (sample 23) - b21, Experiment 1, +TOF MS (50 - 1000) from 0.132 to 0.165 min

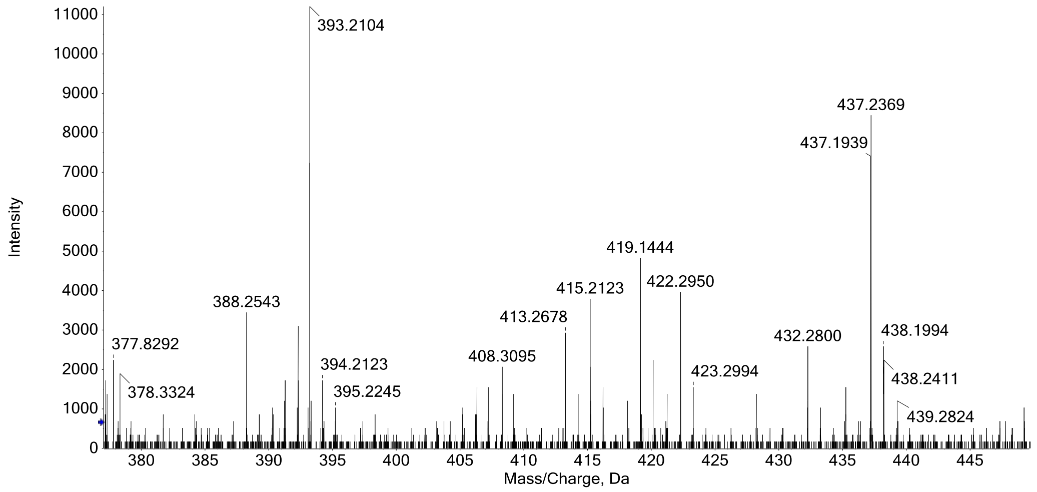

Spectrum from 20191228.wiff (sample 23) - b21, Experiment 1, +TOF MS (50 - 1000) from 0.132 to 0.165 min

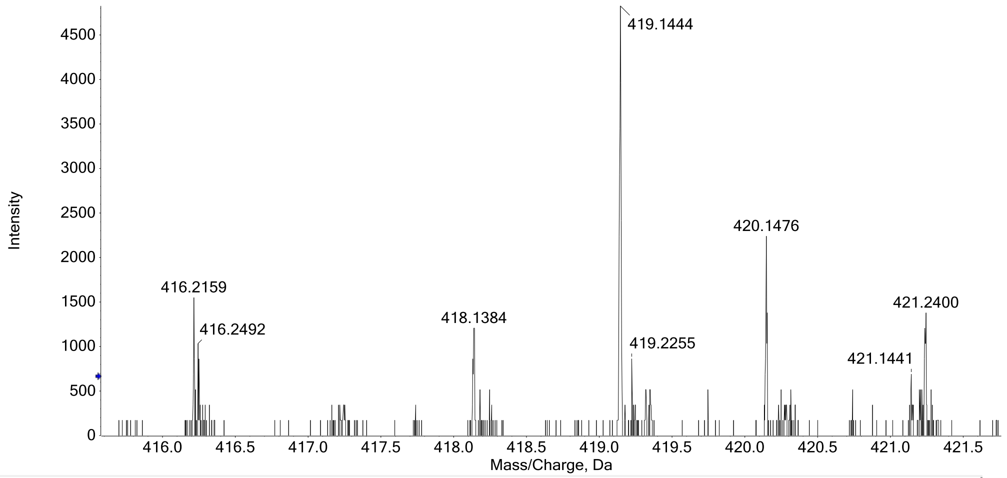

Hit	Formula	m/z	RDB	ppm	MS Rank	MSMS ppm	MSMS Rank	Found
1	SiC26H21F3	419.1437	16.0	1.6	1			NA/NA

3k

Spectrum from 20191228.wiff (sample 22) - b20, Experiment 1, +TOF MS (50 - 1000) from 0.132 to 0.164 min

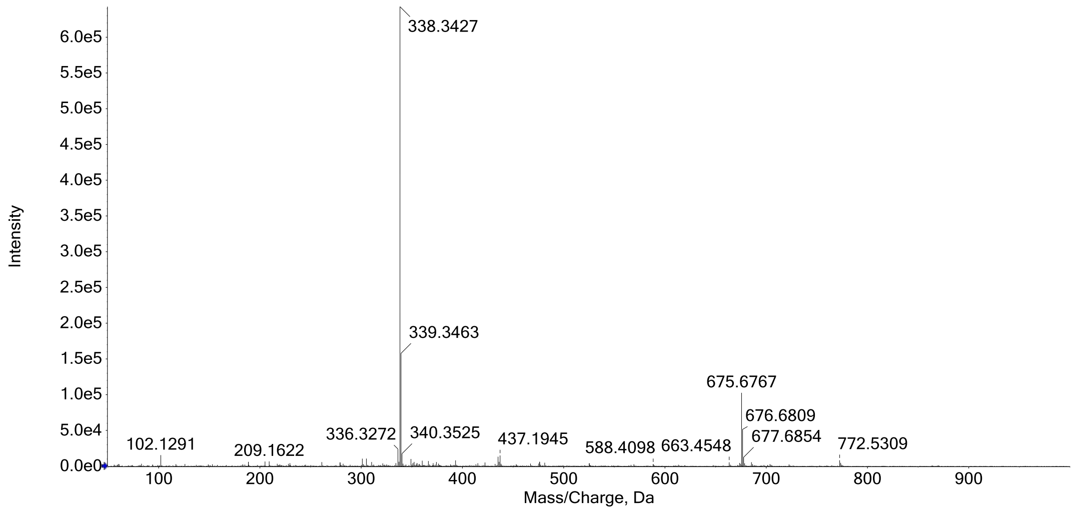

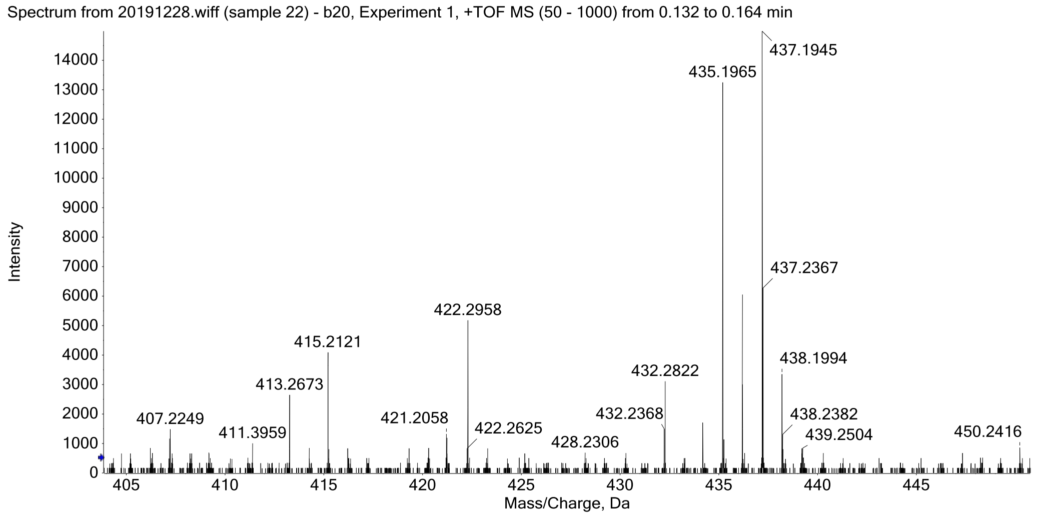

Hit	Formula	m/z	RDB	ppm	MS Rank	MSMS ppm	MSMS Rank	Found
1	Si2C29H30	435.1959	17.0	1.4	1			NA/NA

3l

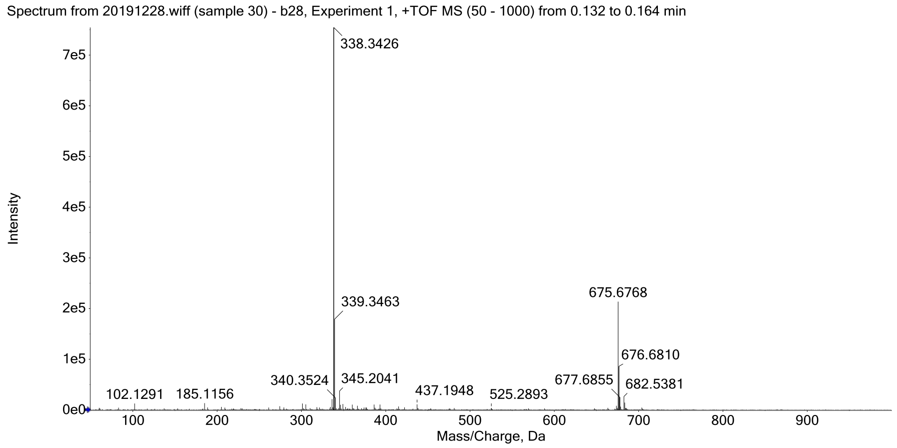

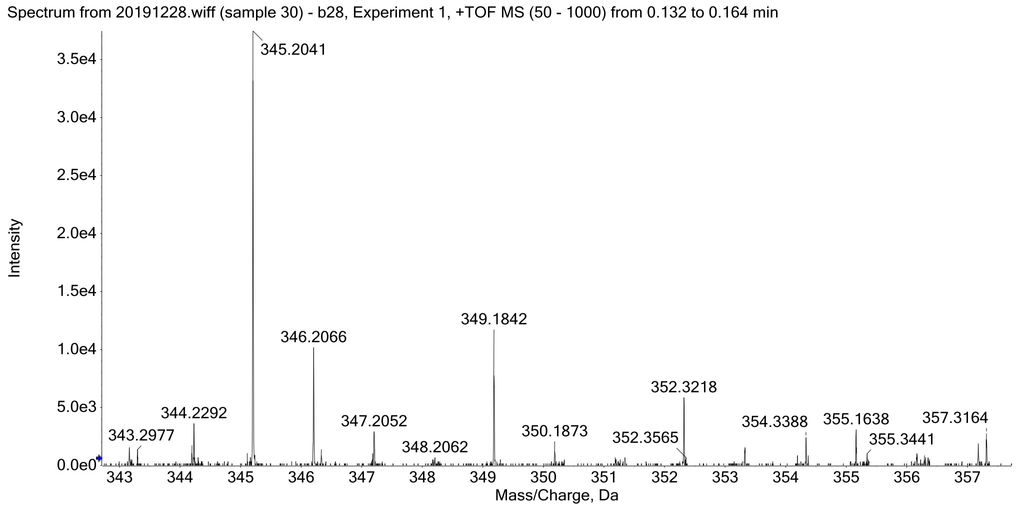

Hit	Formula	m/z	RDB	ppm	MS Rank	MSMS ppm	MSMS Rank	Found
1	SiC24H28	345.2033	12.0	2.3	1			NA/NA

3m

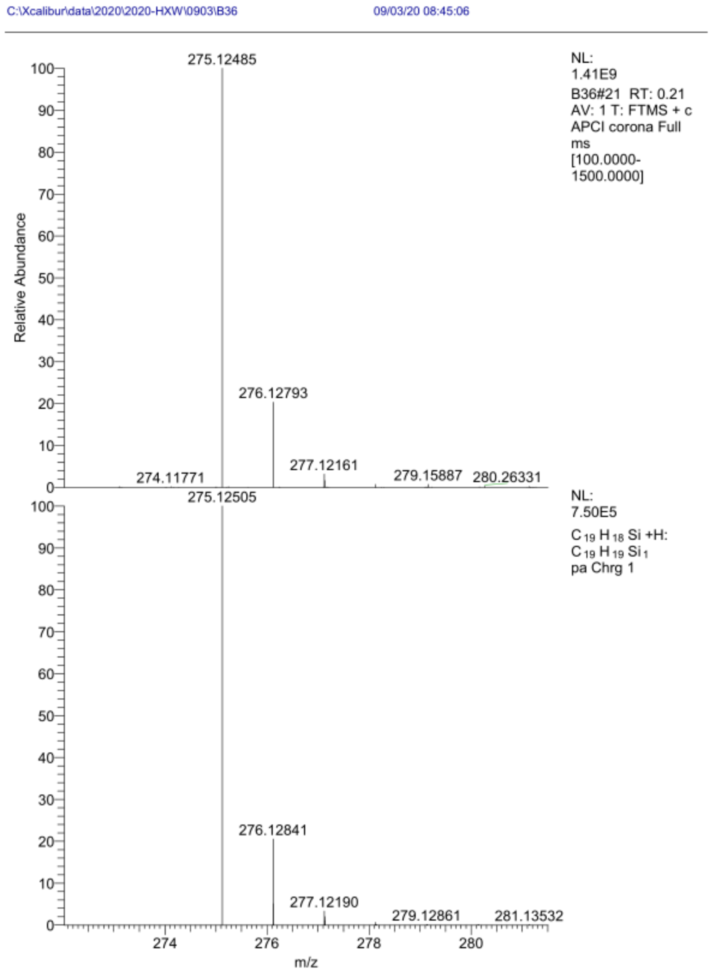

3n

Spectrum from 20191228.wiff (sample 28) - b26, Experiment 1, +TOF MS (50 - 1000) from 0.133 to 0.165 min

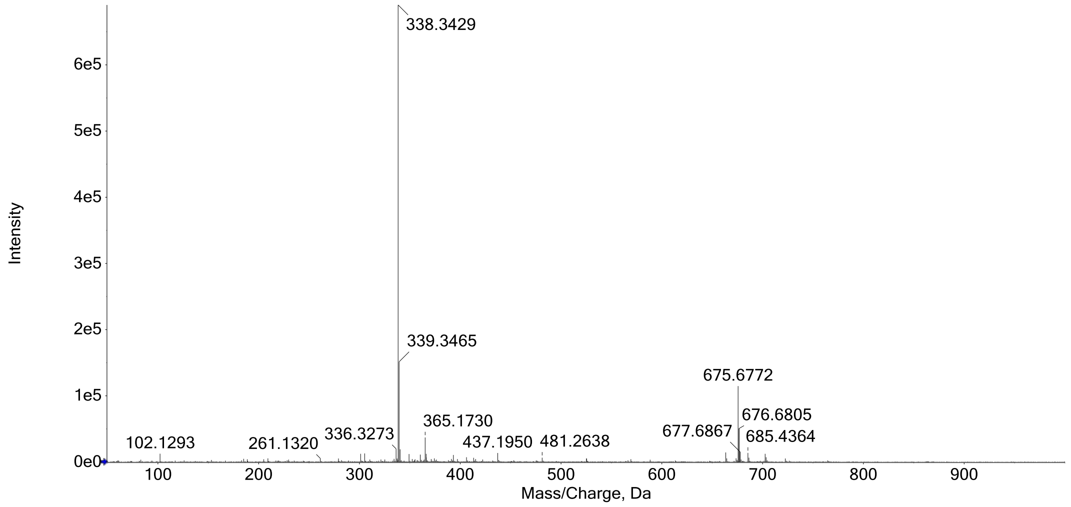

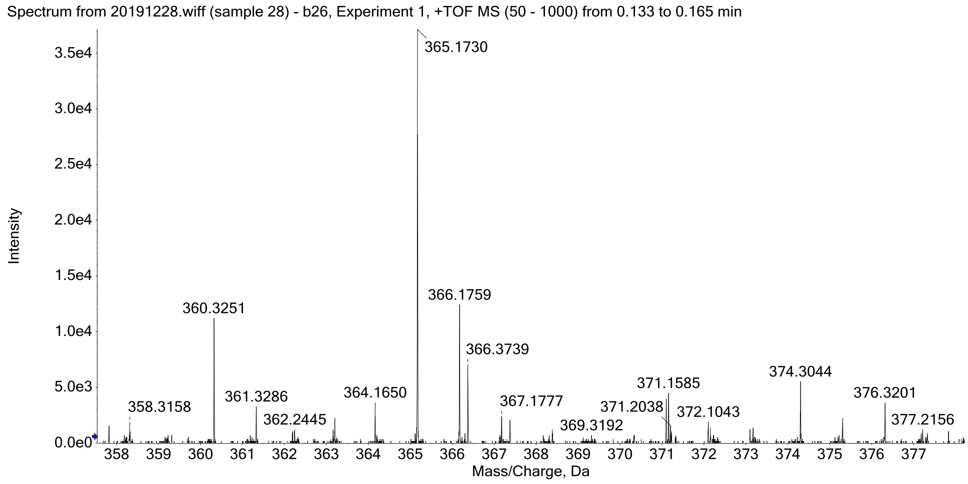

Hit	Formula	m/z	RDB	ppm	MS Rank	MSMS ppm	MSMS Rank	Found
1	SiC26H24	365.1720	16.0	2.7	1			NA/NA

3o

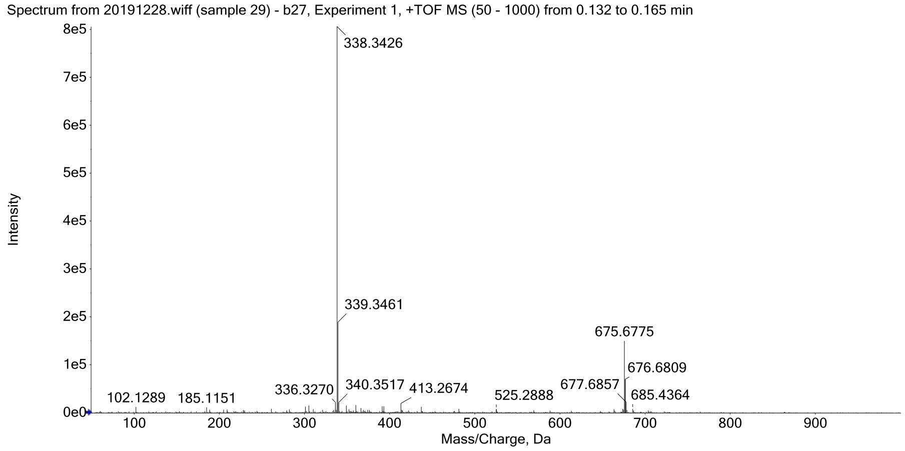

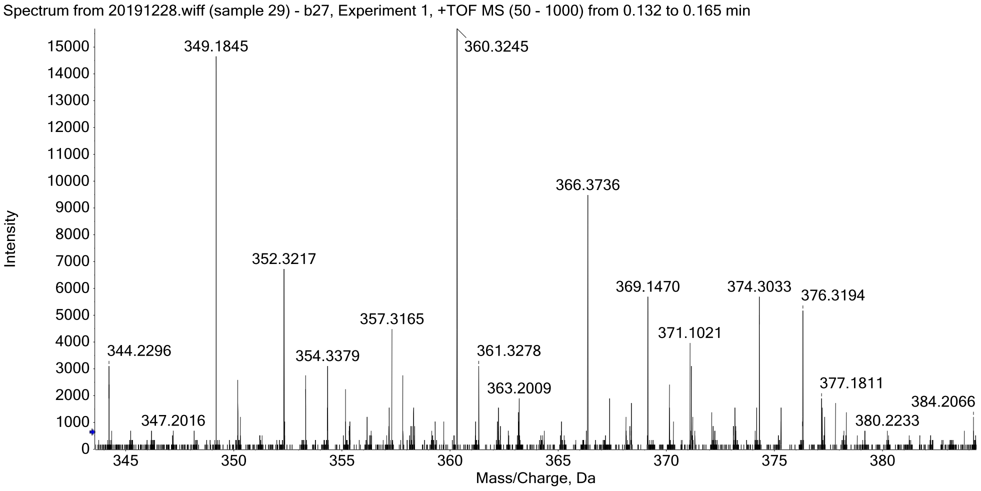

Spectrum from 20191228.wiff (sample 29) - b27, Experiment 1, +TOF MS (50 - 1000) from 0.132 to 0.165 min

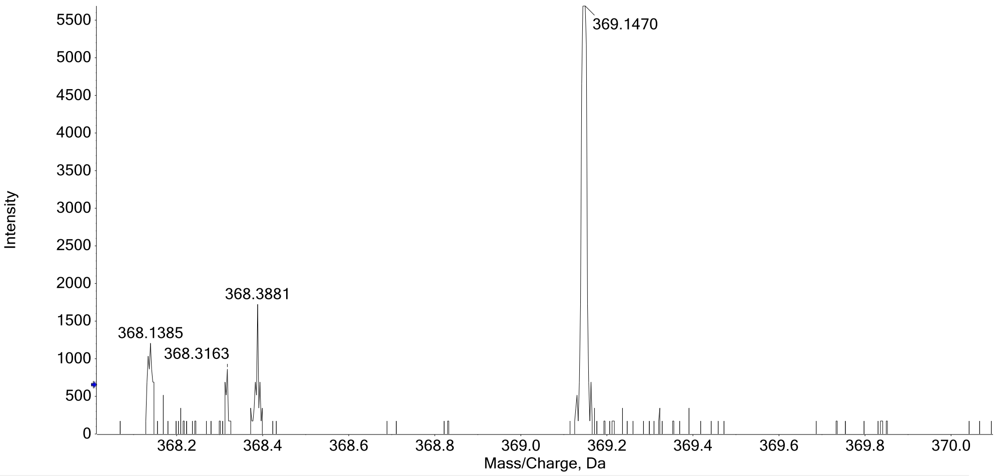

Hit	Formula	m/z	RDB	ppm	MS Rank	MSMS ppm	MSMS Rank	Found
1	SiC25H21F	369.1469	16.0	0.2	1			NA/NA

3p

Spectrum from 20191228.wiff (sample 26) - b24, Experiment 1, +TOF MS (50 - 1000) from 0.132 to 0.165 min

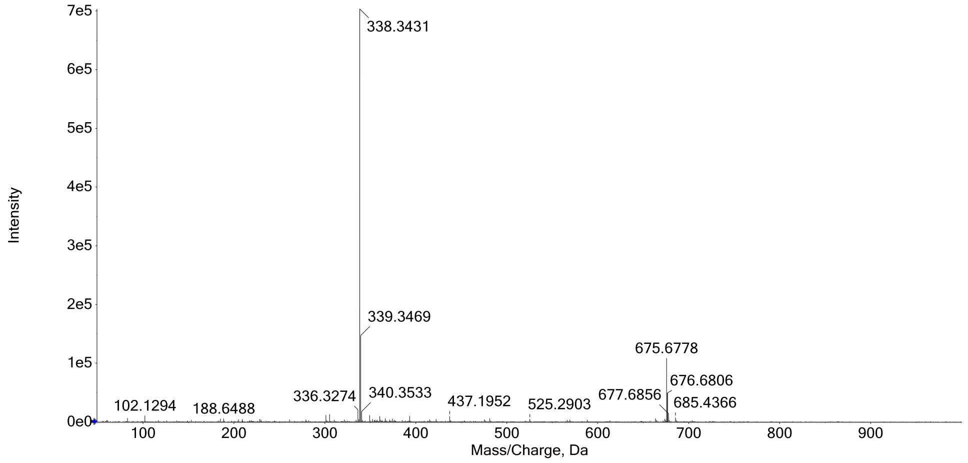

Spectrum from 20191228.wiff (sample 26) - b24, Experiment 1, +TOF MS (50 - 1000) from 0.132 to 0.165 min

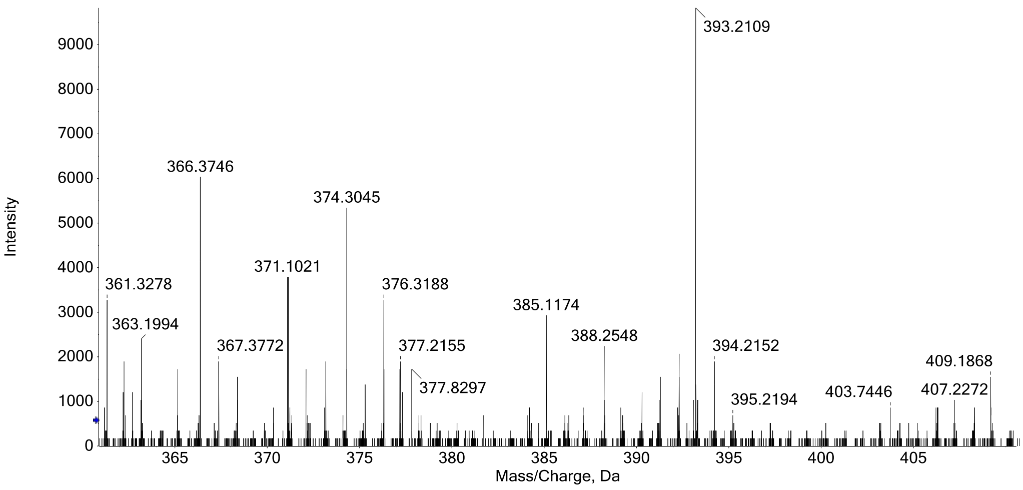

Spectrum from 20191228.wiff (sample 26) - b24, Experiment 1, +TOF MS (50 - 1000) from 0.132 to 0.165 min

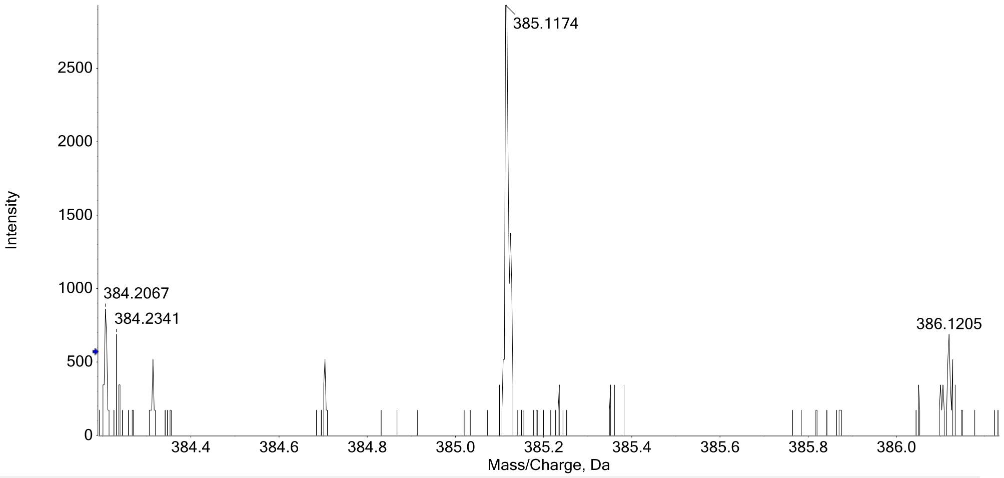

Hit	Formula	m/z	RDB	ppm	MS Rank	MSMS ppm	MSMS Rank	Found
1	SiC25H21Cl	385.1174	16.0	0.0	1			NA/NA

3q

Spectrum from 20191228.wiff (sample 27) - b25, Experiment 1, +TOF MS (50 - 1000) from 0.132 to 0.165 min

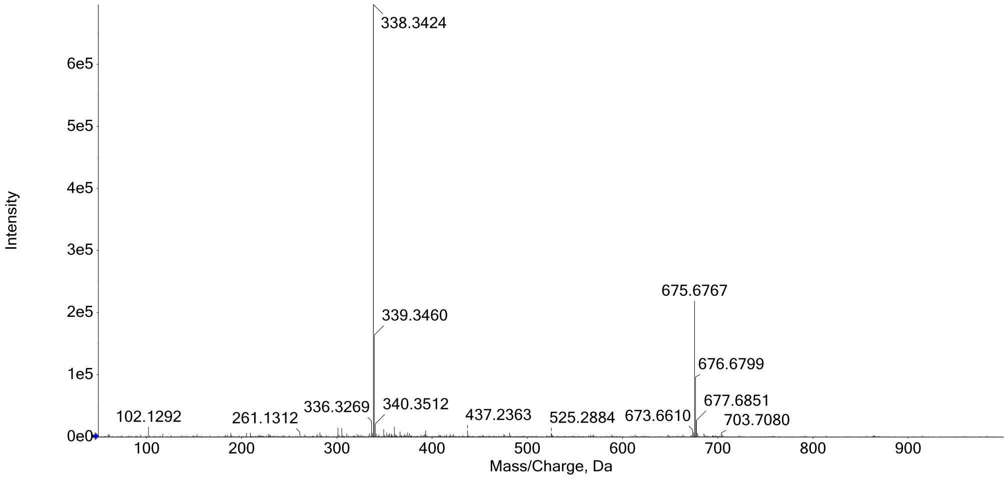

Spectrum from 20191228.wiff (sample 27) - b25, Experiment 1, +TOF MS (50 - 1000) from 0.132 to 0.165 min

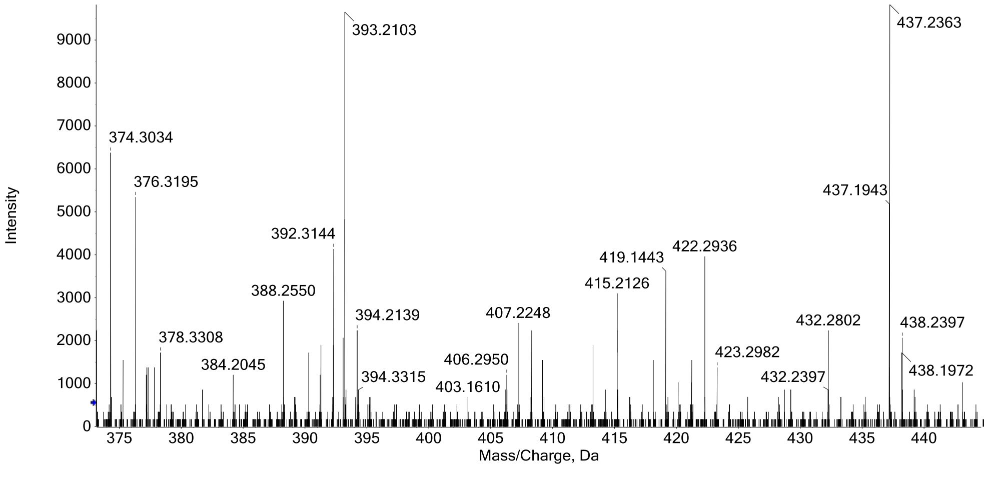

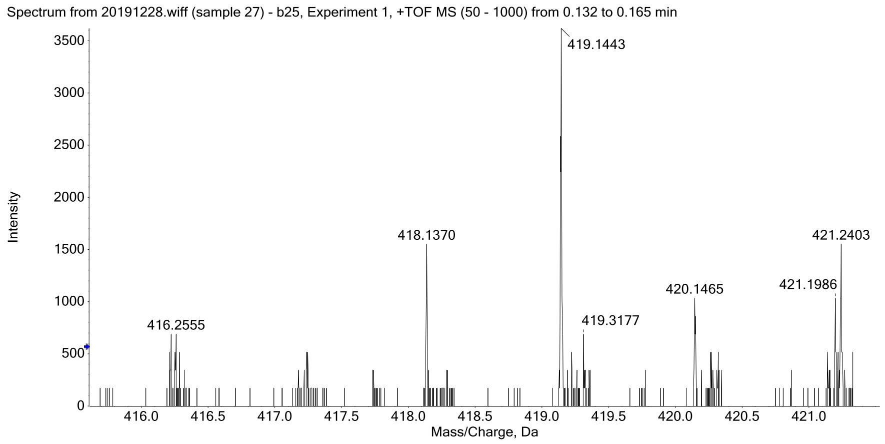

Hit	Formula	m/z	RDB	ppm	MS Rank	MSMS ppm	MSMS Rank	Found
1	SiC26H21F3	419.1437	16.0	1.3	1			NA/NA

3r

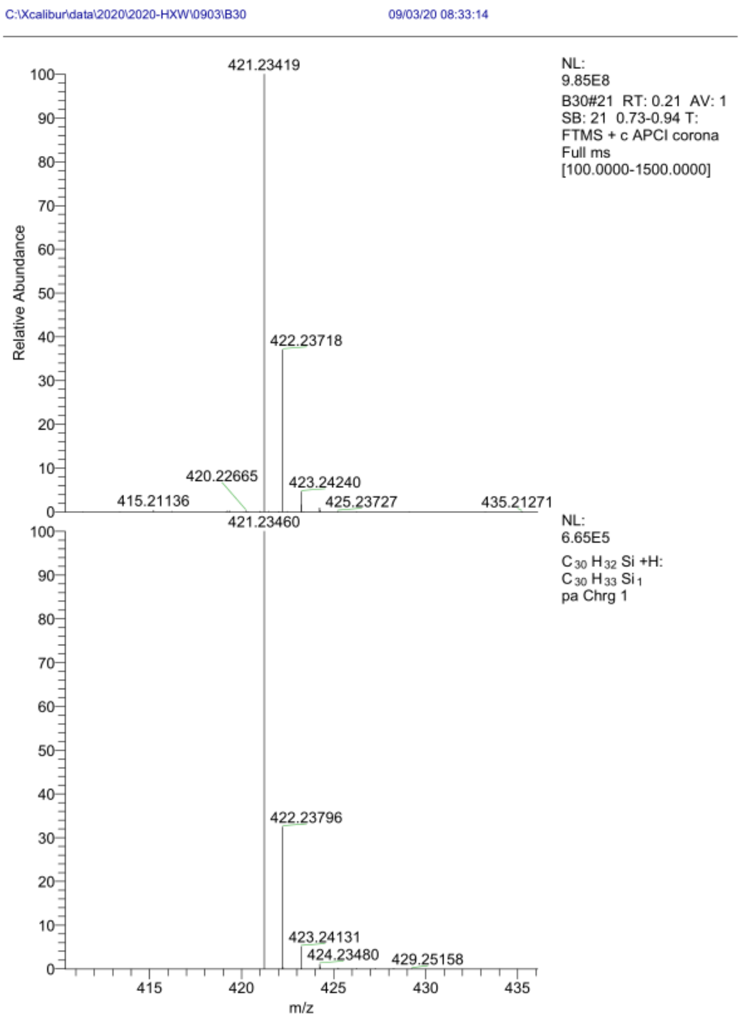

5a

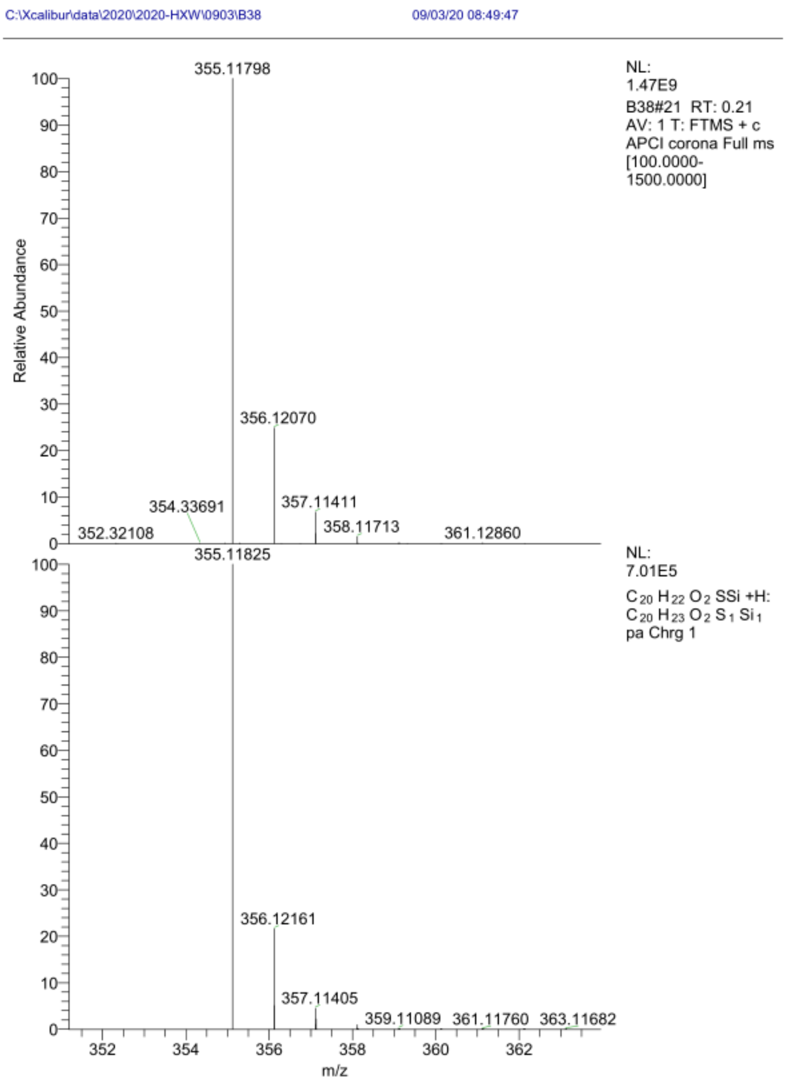

C:\Xcalibur\data\2020\2020-HXW\0903\B38
09/03/20 08:49:47
NL: 1.47E9
B38#21 RT: 0.21
AV: 1 T: FTMS + c APCI corona Full ms [100.0000-1500.0000]
355.11798
356.12070
357.11411
358.11713
354.33691
352.32108
361.12860
NL: 7.01E5
C 20 H 22 O 2 SSi +H: C 20 H 23 O 2 S 1 Si 1 pa Chrg 1
355.11825
356.12161
357.11405
359.11089
361.11760
363.11682
Relative Abundance
m/z

5b

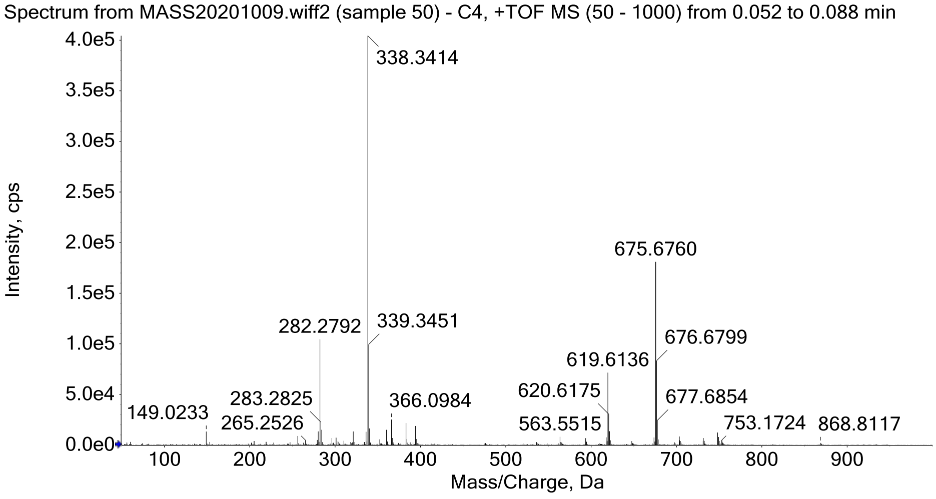

Spectrum from MASS20201009.wiff2 (sample 50) - C4, +TOF MS (50 - 1000) from 0.052 to 0.088 min
338.3414
675.6760
282.2792
339.3451
676.6799
619.6136
620.6175
677.6854
149.0233
283.2825
265.2526
366.0984
563.5515
753.1724
868.8117
Intensity, cps
Mass/Charge, Da

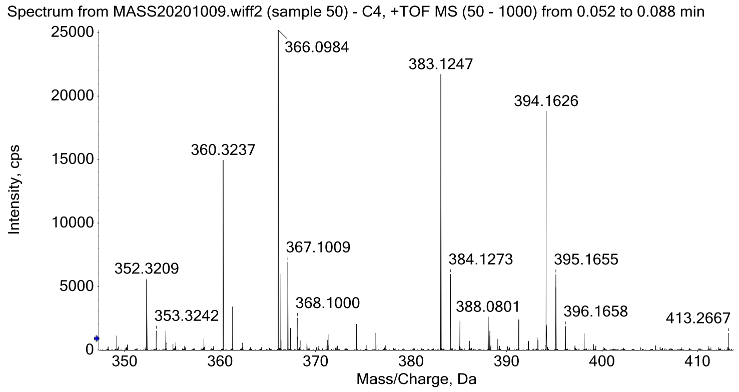

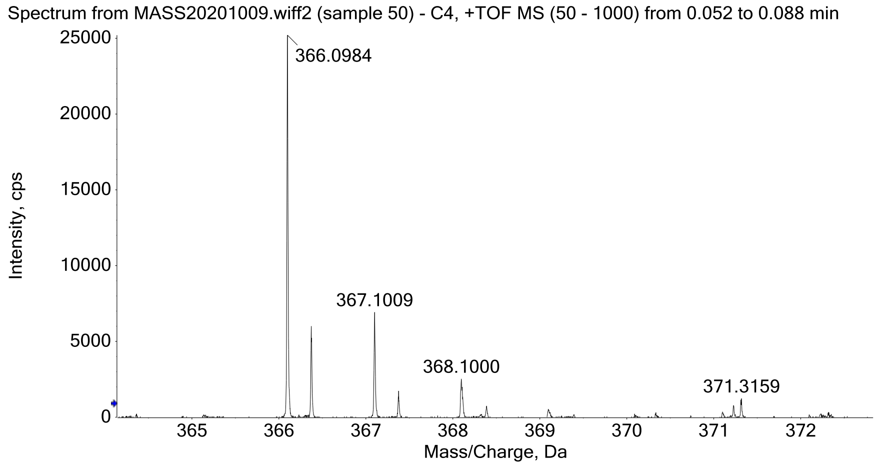

Hit	Formula	m/z	RDB	ppm	MS Rank	MSMS ppm	MSMS Rank	Found
1	C20H19NO2SSi	366.0979	13.0	1.5	1			NA/NA

5c

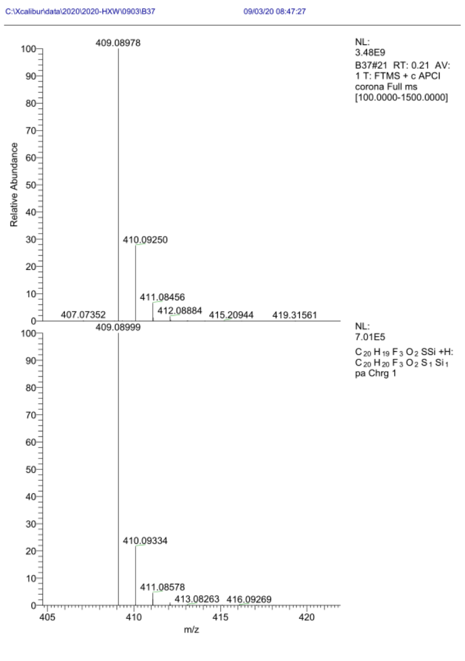

6a

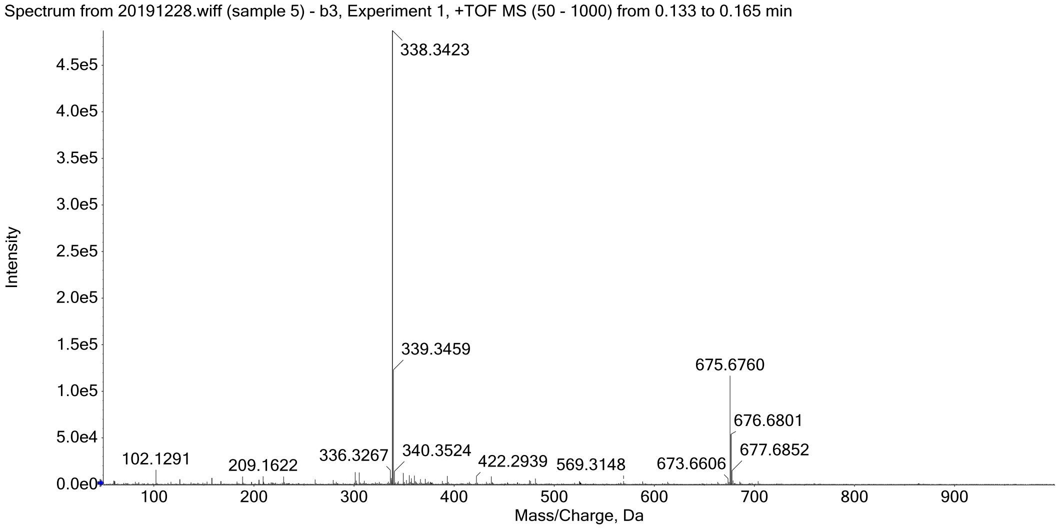

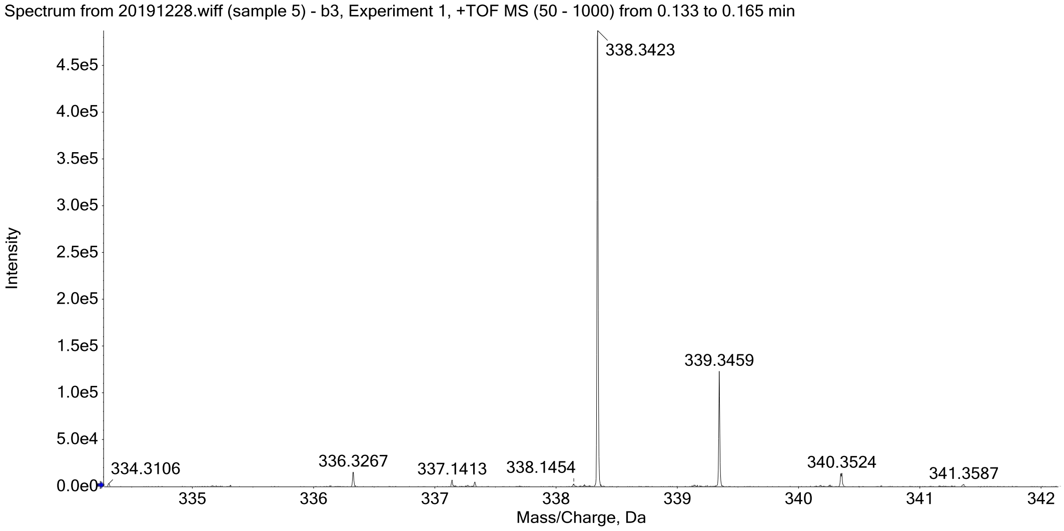

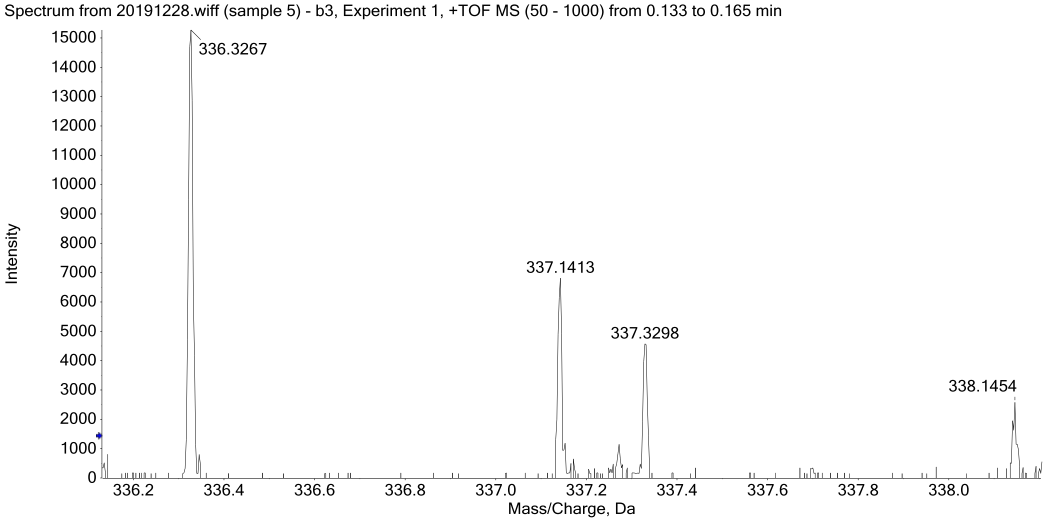

Hit	Formula	m/z	RDB	ppm	MS Rank	MSMS ppm	MSMS Rank	Found
1	SiC24H20	337.1407	16.0	1.8	1			NA/NA

6b and 6c

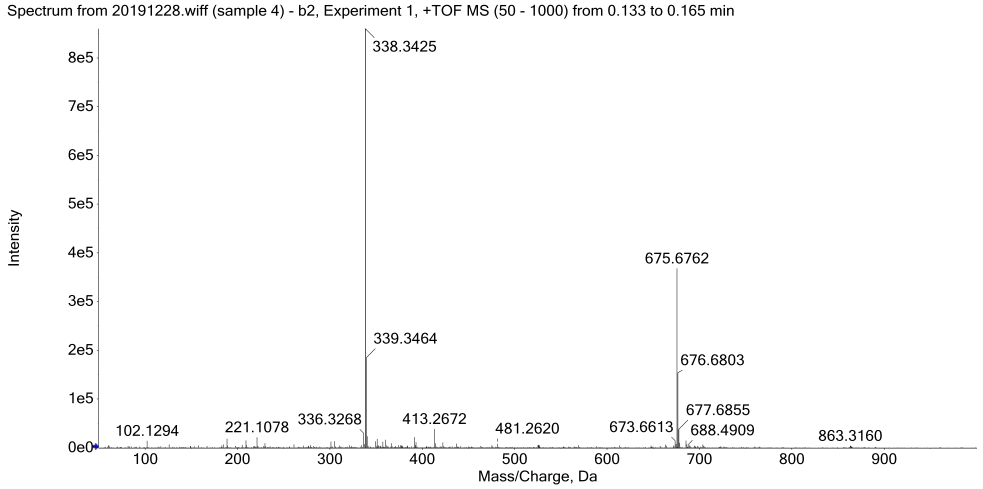

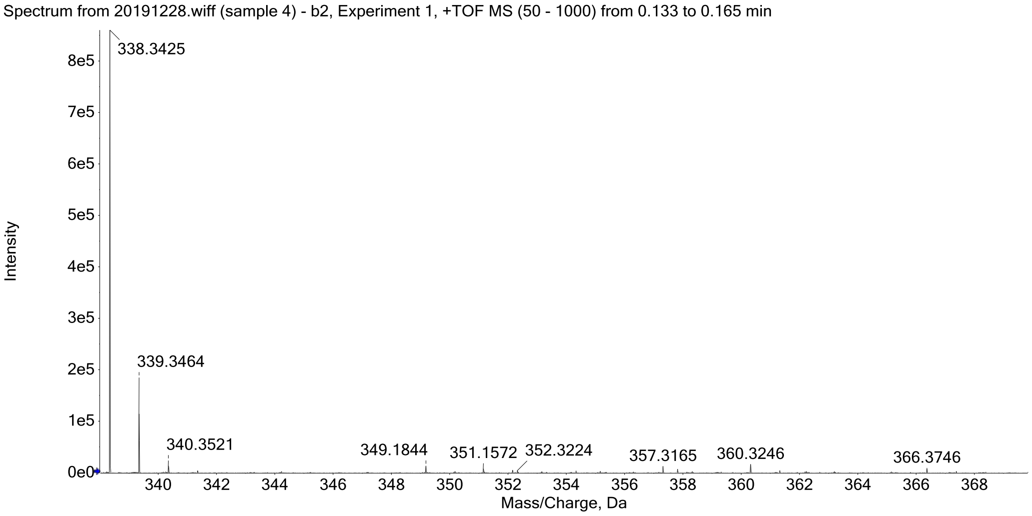

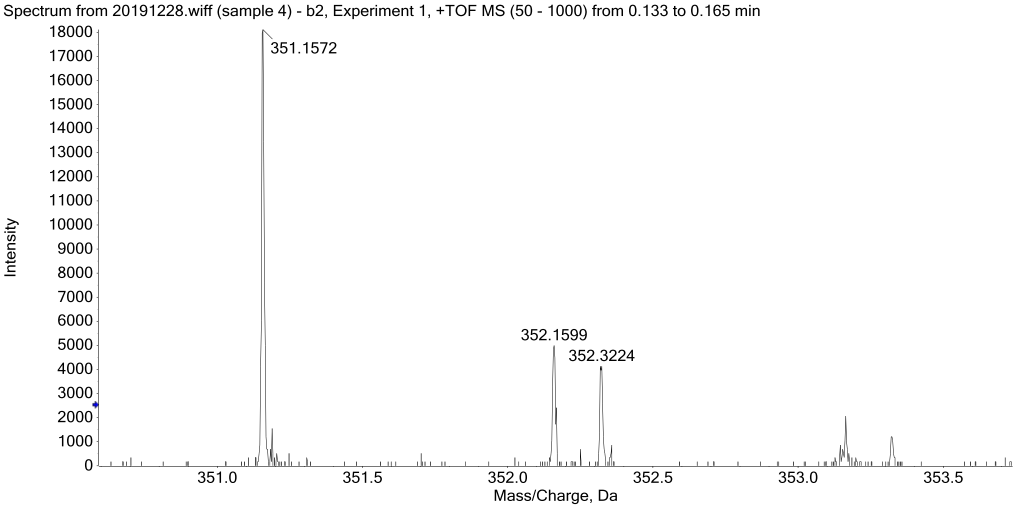

Hit	Formula	m/z	RDB	ppm	MS Rank	MSMS ppm	MSMS Rank	Found
1	SiC25H22	351.1564	16.0	2.4	1			NA/NA

6d

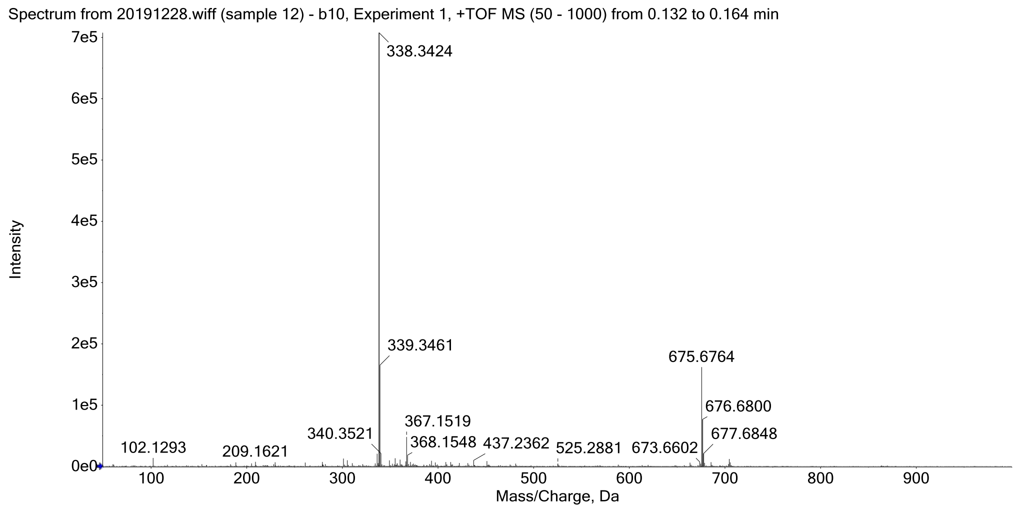

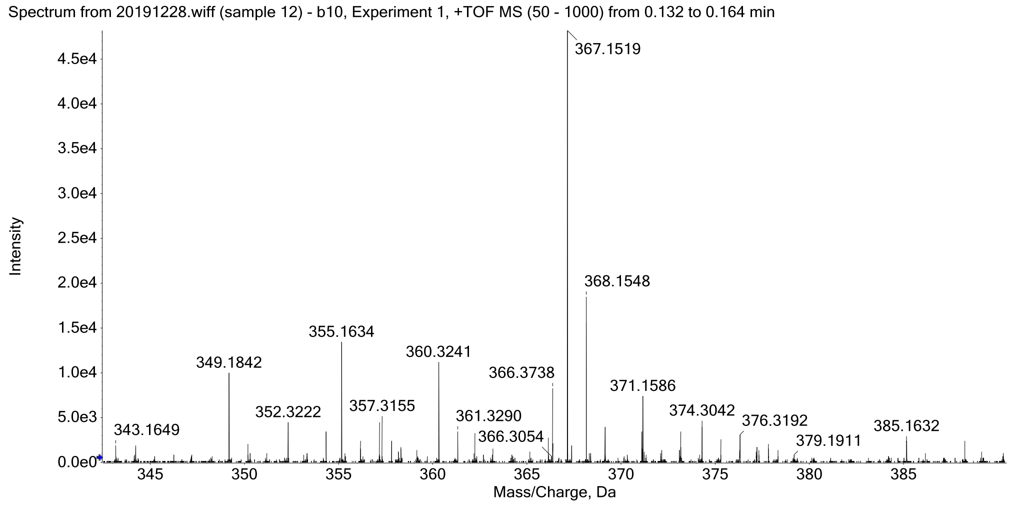

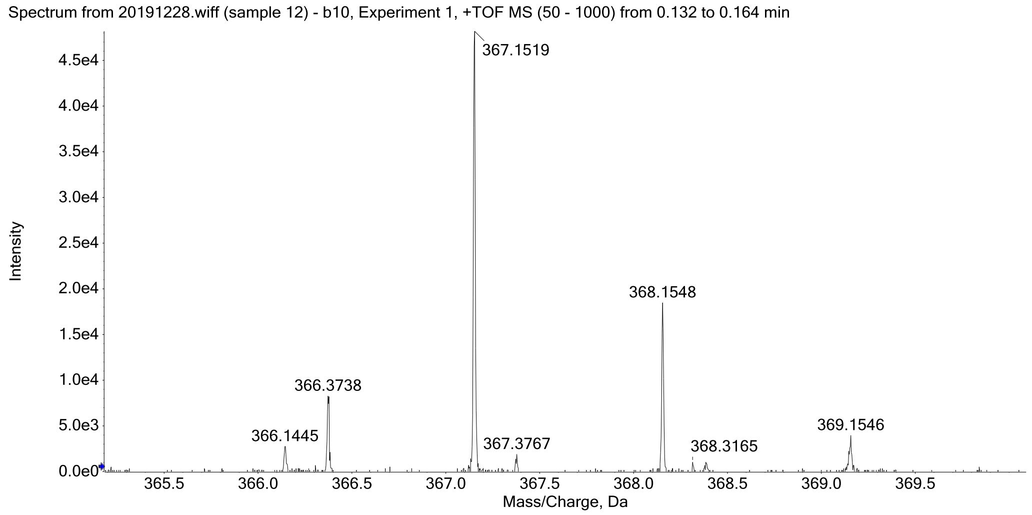

Hit	Formula	m/z	RDB	ppm	MS Rank	MSMS ppm	MSMS Rank	Found
1	SiC25H22O	367.1513	16.0	1.7	1			NA/NA

6e

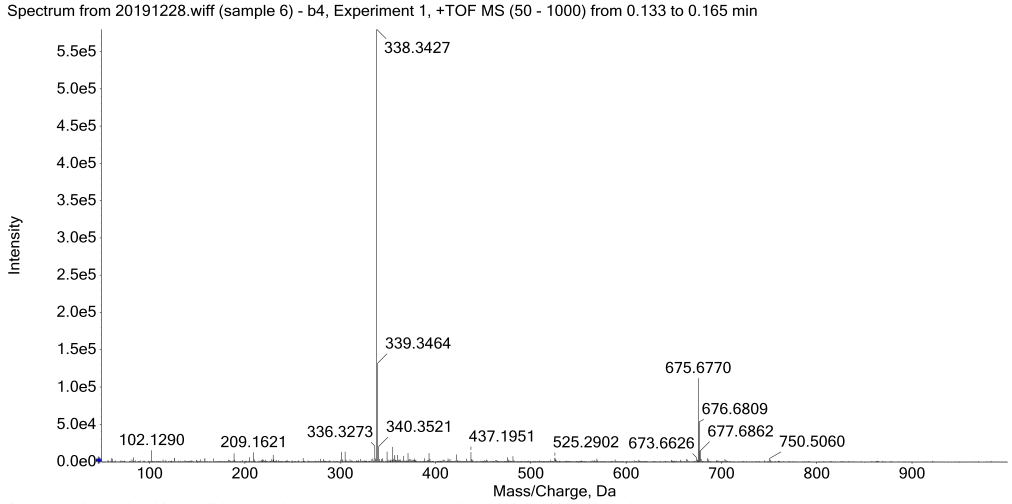

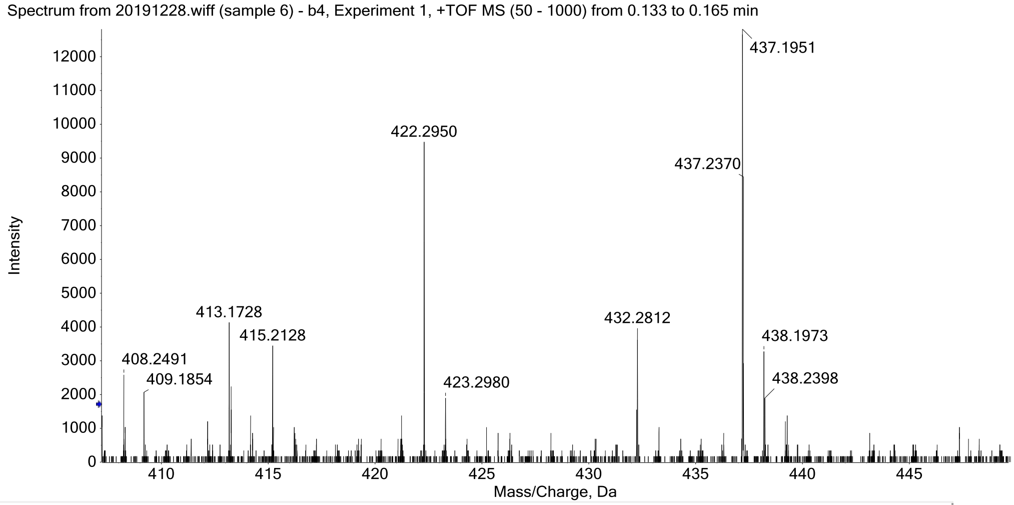

Hit	Formula	m/z	RDB	ppm	MS Rank	MSMS ppm	MSMS Rank	Found
1	SiC30H24	413.1720	20.0	1.9	1			NA/NA

6f and 6g

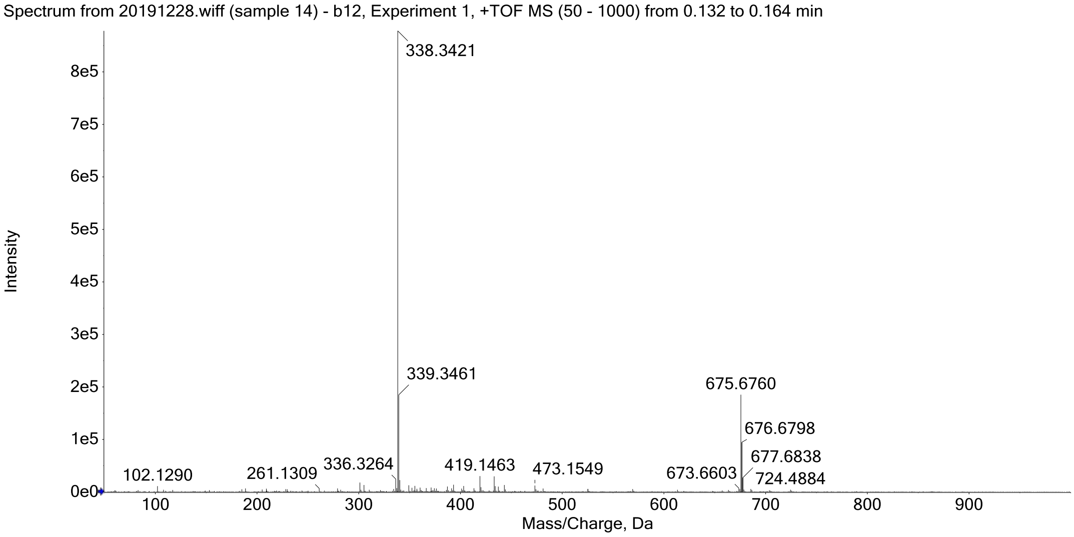

Spectrum from 20191228.wiff (sample 14) - b12, Experiment 1, +TOF MS (50 - 1000) from 0.132 to 0.164 min

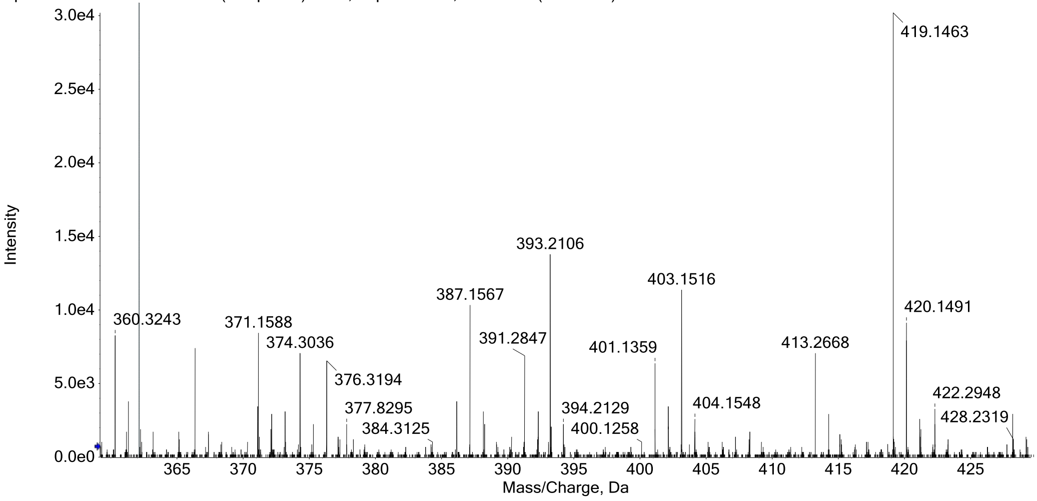

Spectrum from 20191228.wiff (sample 14) - b12, Experiment 1, +TOF MS (50 - 1000) from 0.132 to 0.164 min

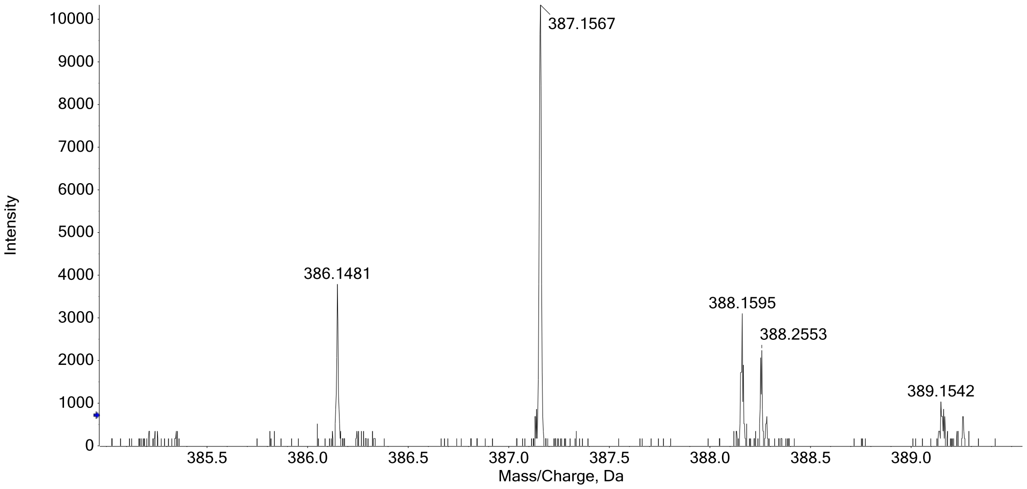

Hit	Formula	m/z	RDB	ppm	MS Rank	MSMS ppm	MSMS Rank	Found
1	SiC28H22	387.1564	19.0	0.9	1			NA/NA

6h

Spectrum from 20191228.wiff (sample 7) - b5, Experiment 1, +TOF MS (50 - 1000) from 0.133 to 0.165 min

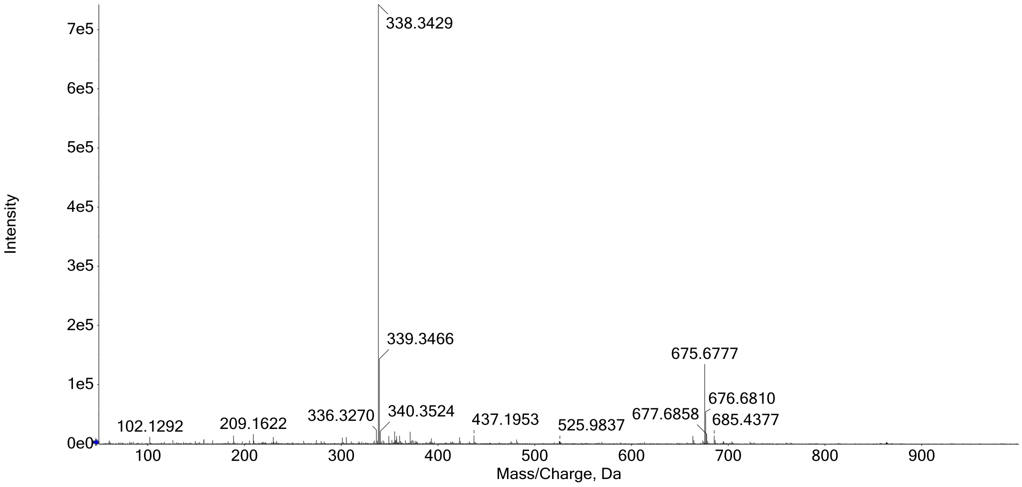

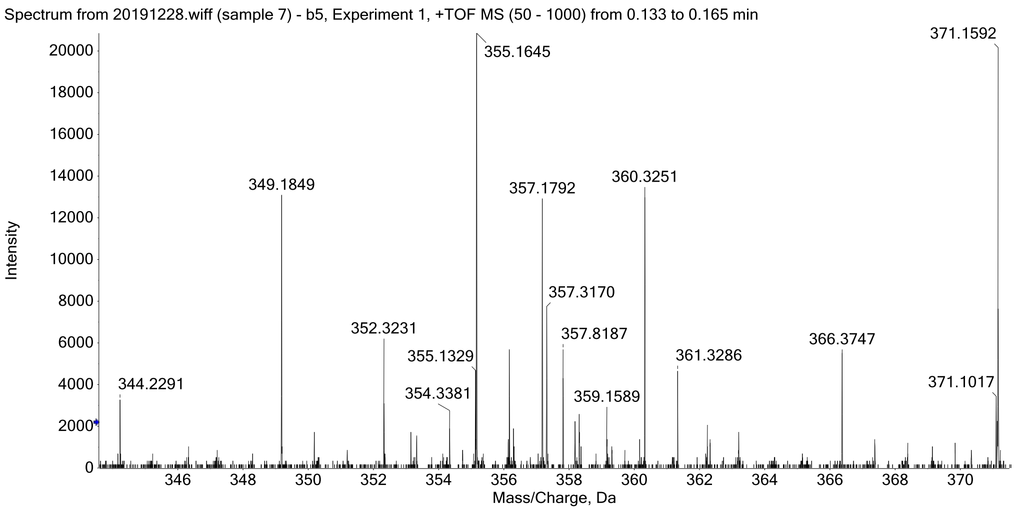

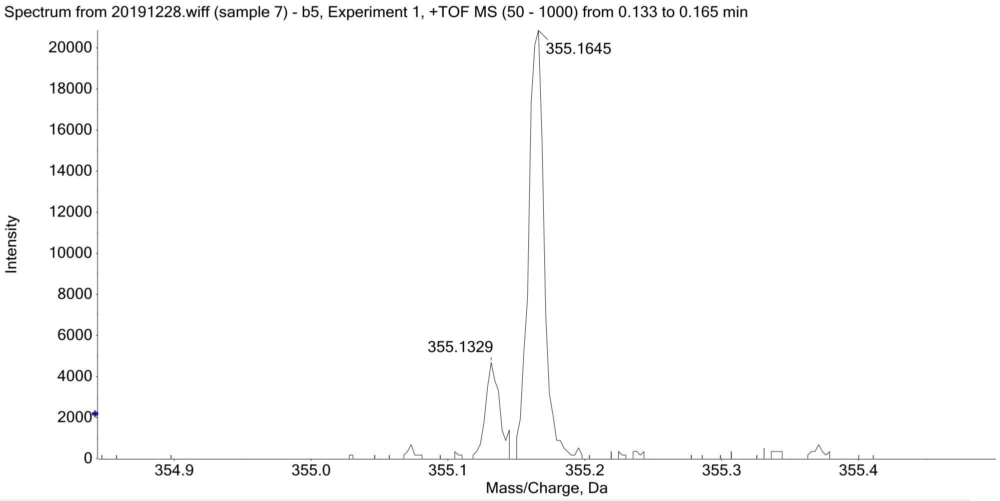

Hit	Formula	m/z	RDB	ppm	MS Rank	MSMS ppm	MSMS Rank	Found
1	SiC24H19F	355.1313	16.0	4.6	1			NA/NA

6i

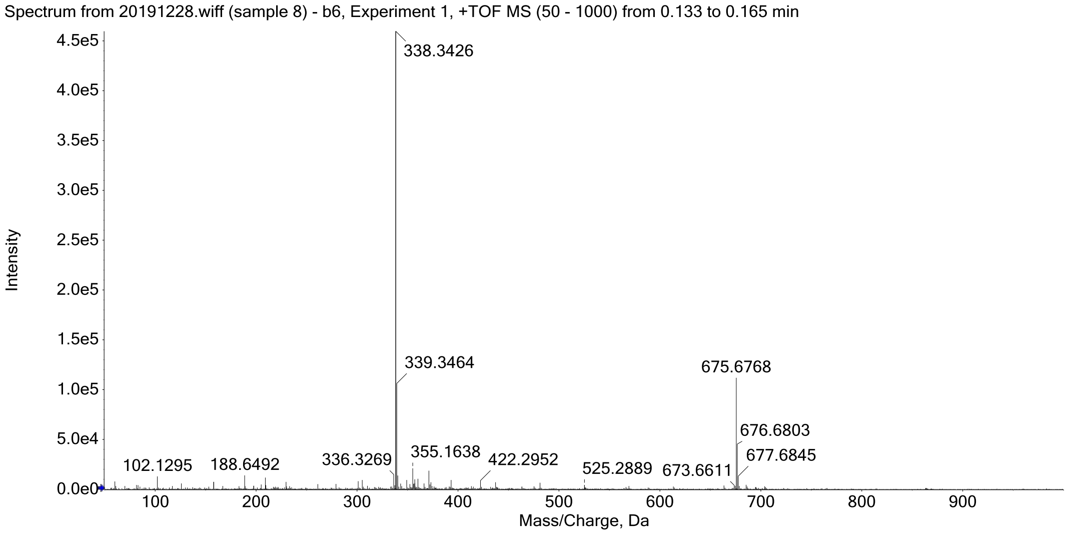

Spectrum from 20191228.wiff (sample 8) - b6, Experiment 1, +TOF MS (50 - 1000) from 0.133 to 0.165 min

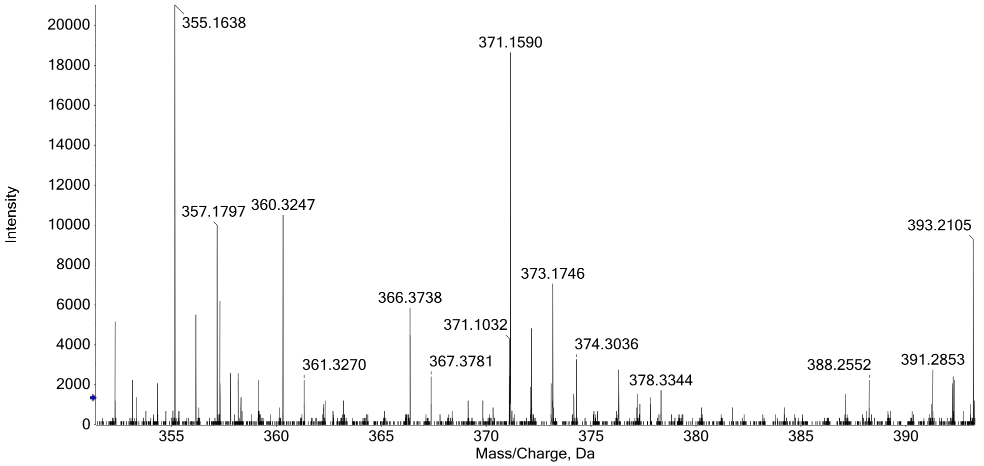

Spectrum from 20191228.wiff (sample 8) - b6, Experiment 1, +TOF MS (50 - 1000) from 0.133 to 0.165 min

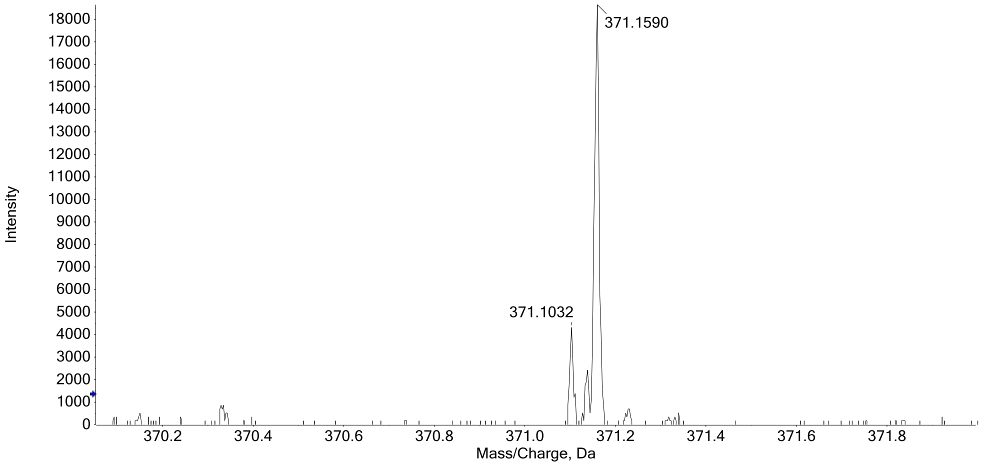

Hit	Formula	m/z	RDB	ppm	MS Rank	MSMS ppm	MSMS Rank	Found
1	SiC24H19Cl	371.1017	16.0	4.0	1			NA/NA

6j and 6k

Spectrum from 20191228.wiff (sample 9) - b7, Experiment 1, +TOF MS (50 - 1000) from 0.133 to 0.165 min

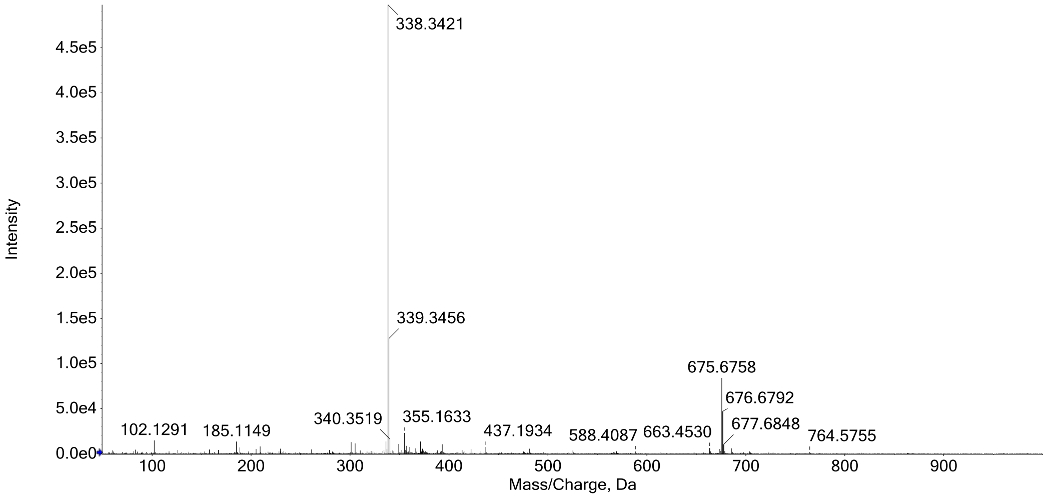

Spectrum from 20191228.wiff (sample 9) - b7, Experiment 1, +TOF MS (50 - 1000) from 0.133 to 0.165 min

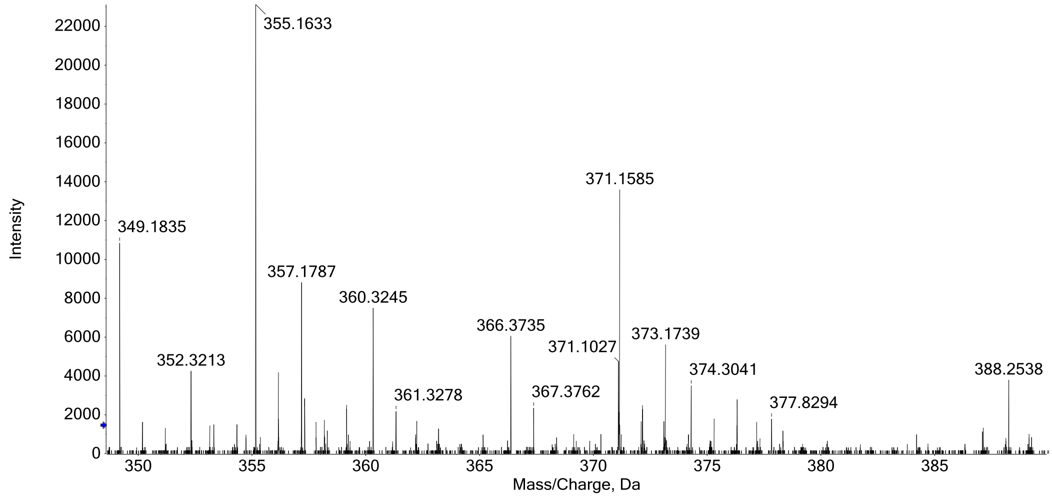

Spectrum from 20191228.wiff (sample 9) - b7, Experiment 1, +TOF MS (50 - 1000) from 0.133 to 0.165 min

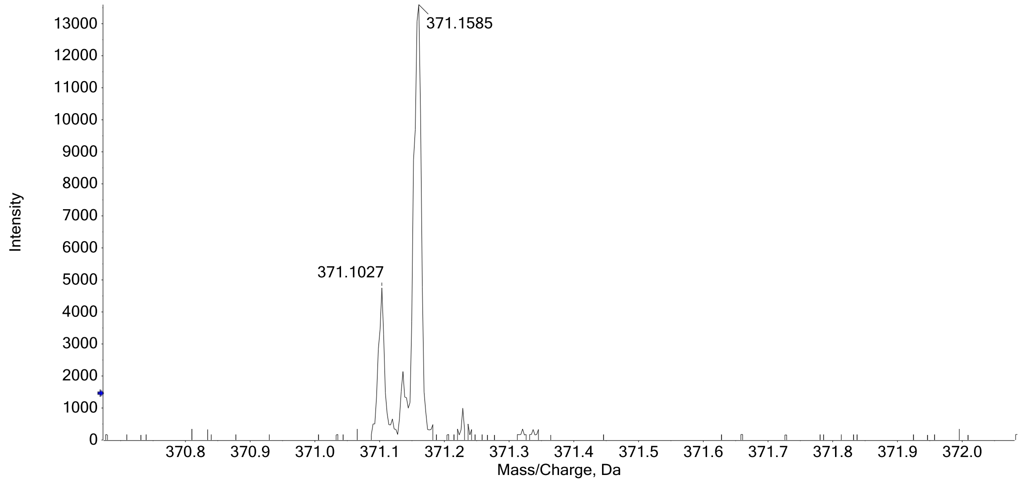

Hit	Formula	m/z	RDB	ppm	MS Rank	MSMS ppm	MSMS Rank	Found
1	SiC24H19Cl	371.1017	16.0	2.6	1			NA/NA

6l

Spectrum from 20191228.wiff (sample 10) - b8, Experiment 1, +TOF MS (50 - 1000) from 0.132 to 0.165 min

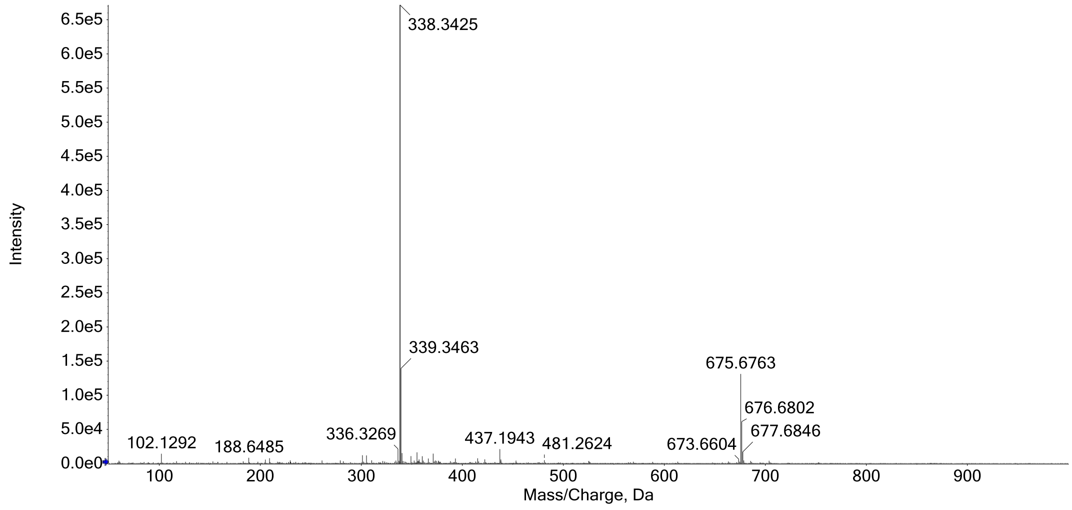

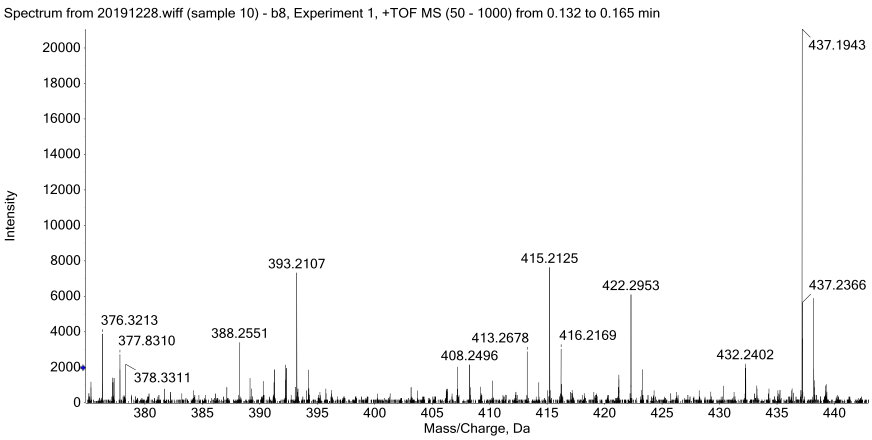

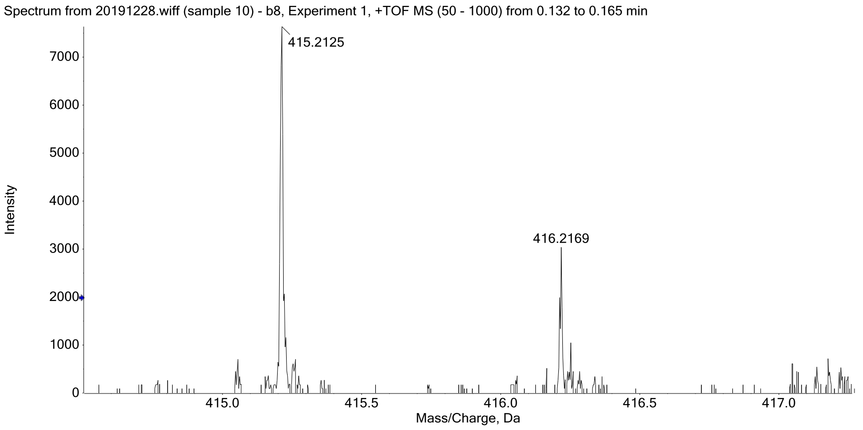

Hit	Formula	m/z	RDB	ppm	MS Rank	MSMS ppm	MSMS Rank	Found
1	SiC24H19Br	415.0512	16.0	4.5	1			NA/NA

6m

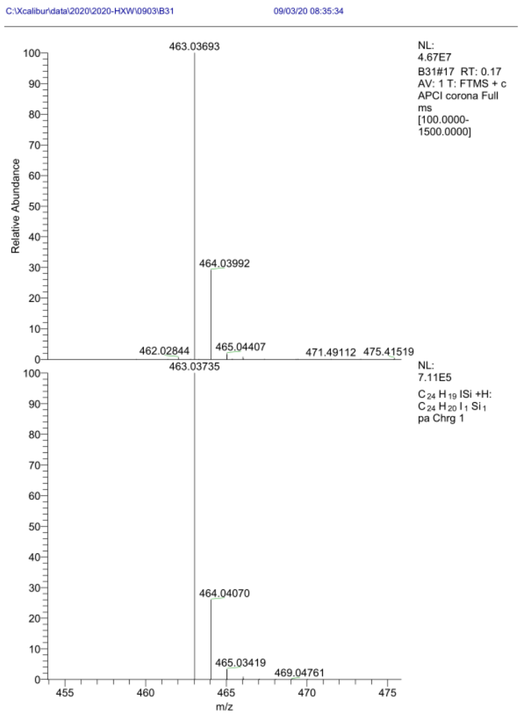

6n

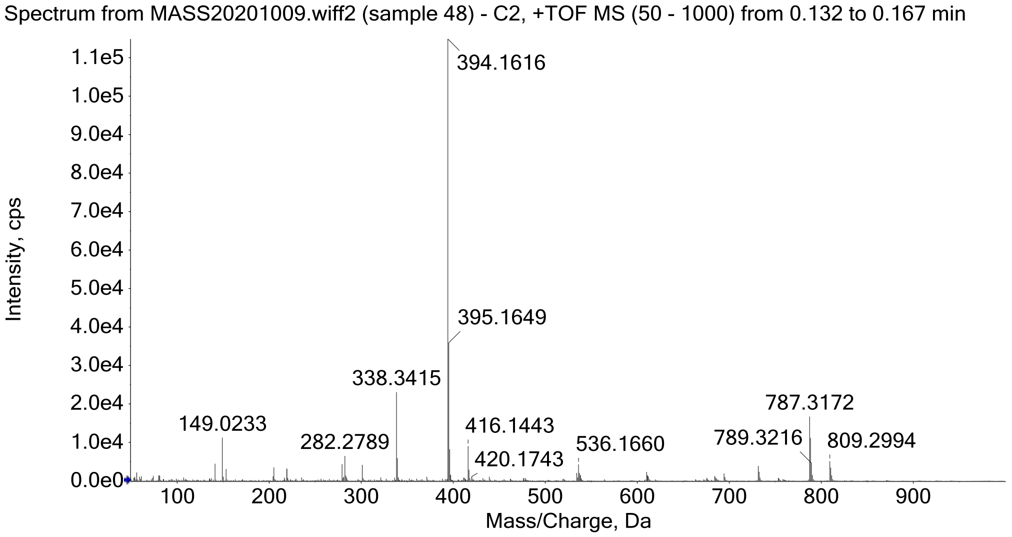

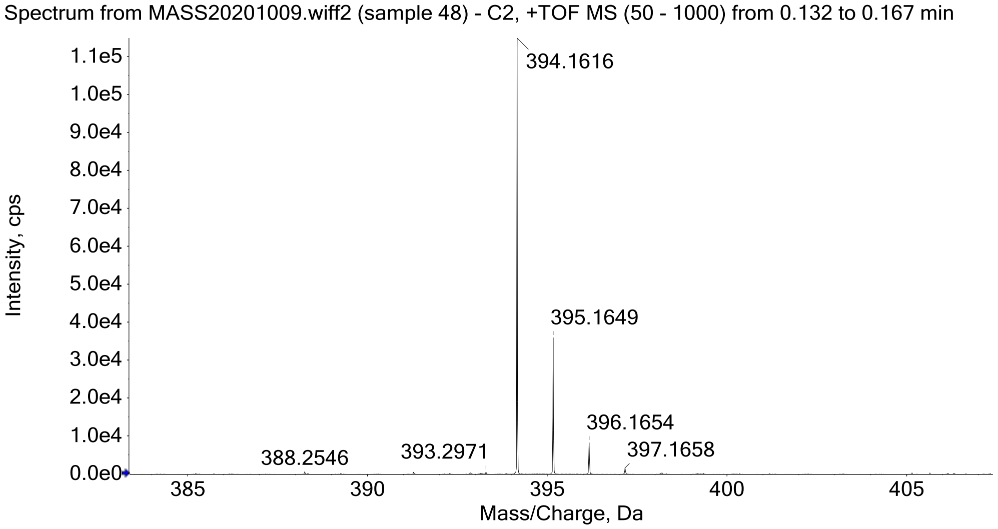

Hit	Formula	m/z	RDB	ppm	MS Rank	MSMS ppm	MSMS Rank	Found
1	C26H23NOSi	394.1622	17.0	-1.4	1			NA/NA

6o

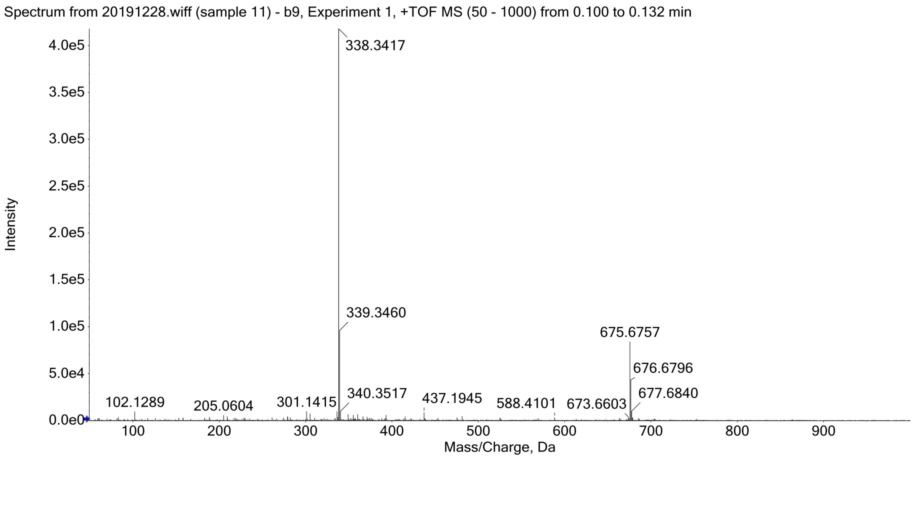

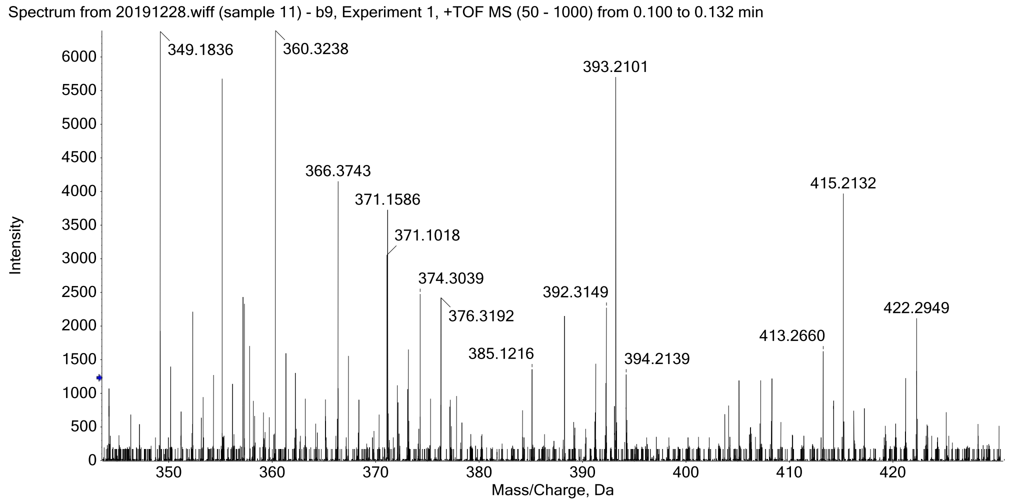

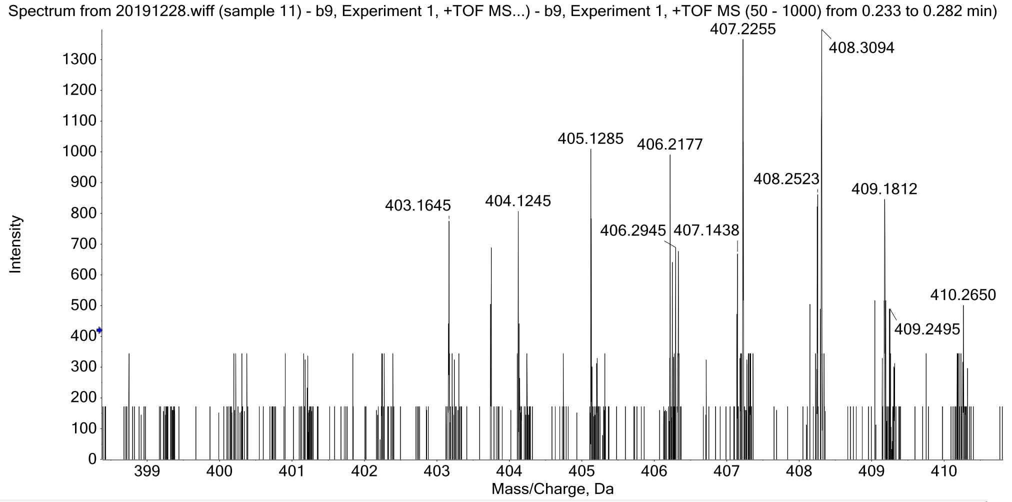

Hit	Formula	m/z	RDB	ppm	MS Rank	MSMS ppm	MSMS Rank	Found
1	SiC25H19F3	405.1281	16.0	1.0	1			NA/NA

6p

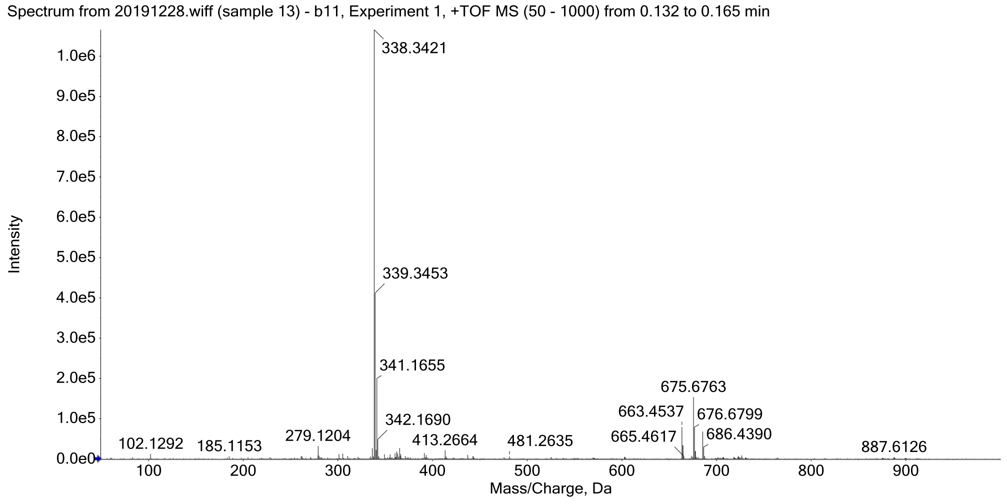

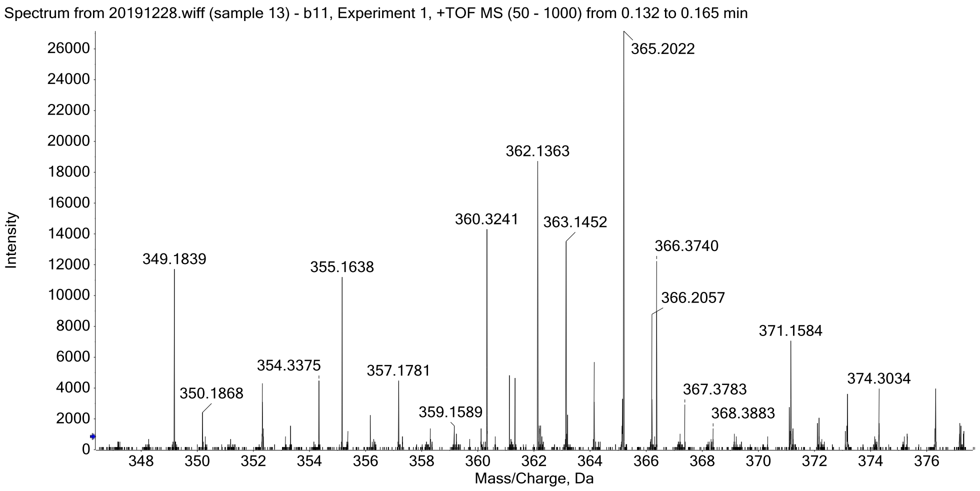

Hit	Formula	m/z	RDB	ppm	MS Rank	MSMS ppm	MSMS Rank	Found
1	SiC25H19N	362.1360	18.0	1.0	1			NA/NA

6q

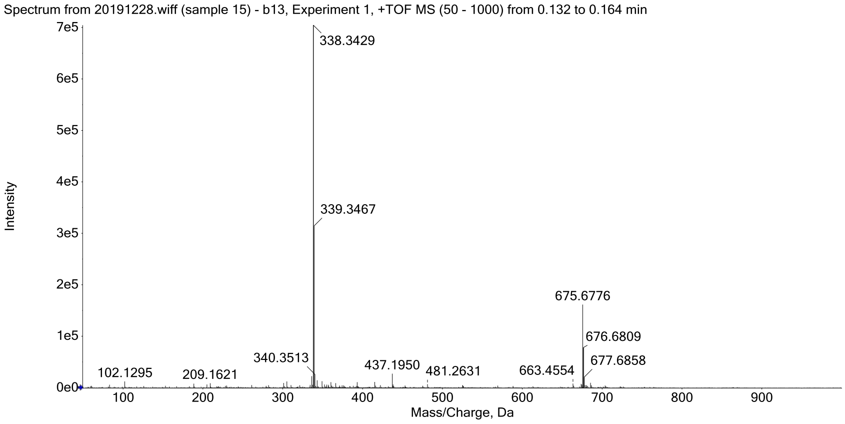

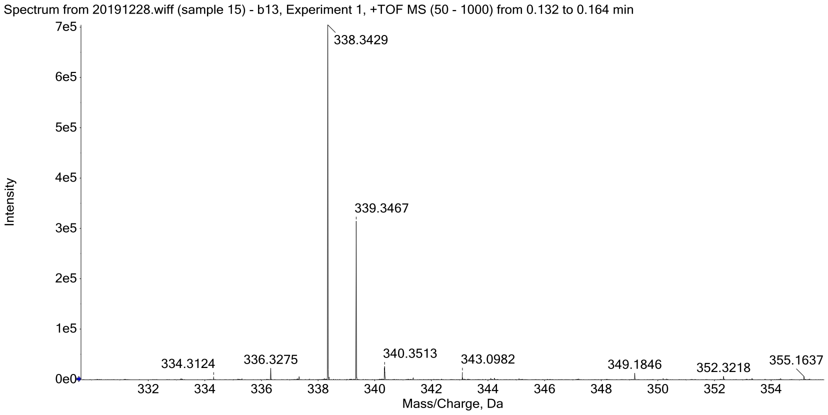

6r

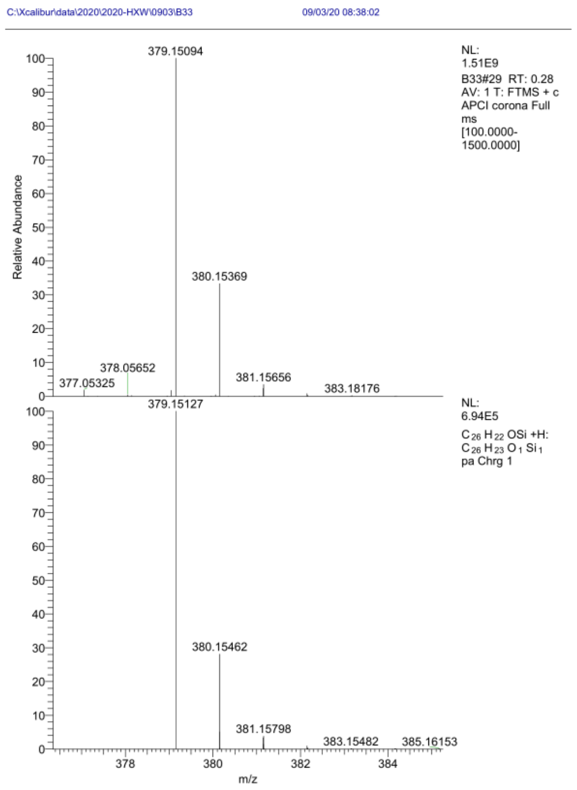
C:\Xcalibur\data\2020\2020-HXW\0903\B33
09/03/20 08:38:02
NL:
1.51E9
B33#29 RT: 0.28
AV: 1 T: FTMS + c
APCI corona Full
ms
[100.0000-
1500.0000]
379.15094
380.15369
378.05652
377.05325
381.15656
383.18176
Relative Abundance
NL:
6.94E5
C26 H22 OSi +H:
C26 H23 O1 Si1
pa Chrg 1
379.15127
380.15462
381.15798
383.15482
385.16153
m/z

6s

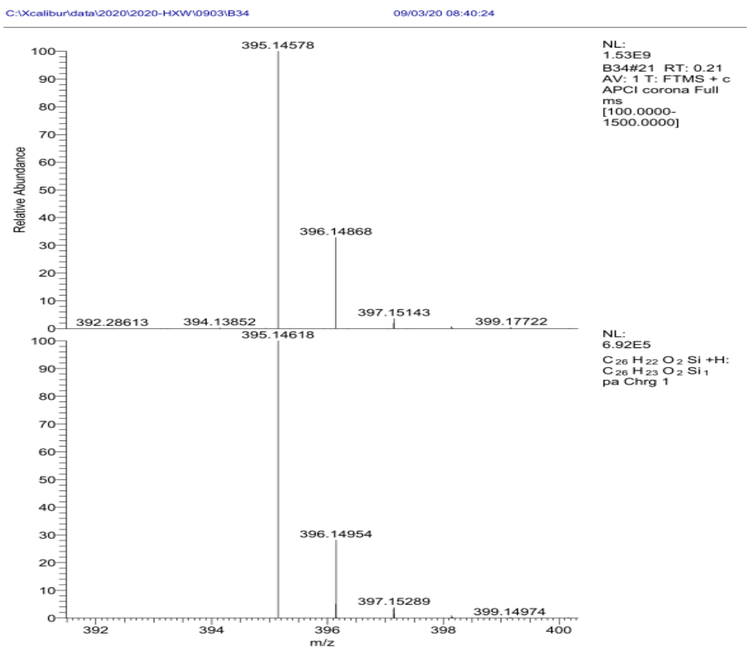
C:\Xcalibur\data\2020\2020-HXW\0903\B34
09/03/20 08:40:24
NL:
1.53E9
B34#21 RT: 0.21
AV: 1 T: FTMS + c
APCI corona Full
ms
[100.0000-
1500.0000]
395.14578
396.14868
392.28613
394.13852
397.15143
399.17722
Relative Abundance
NL:
6.92E5
C26 H22 O2 Si +H:
C26 H23 O2 Si1
pa Chrg 1
395.14618
396.14954
397.15289
399.14974
m/z

6t

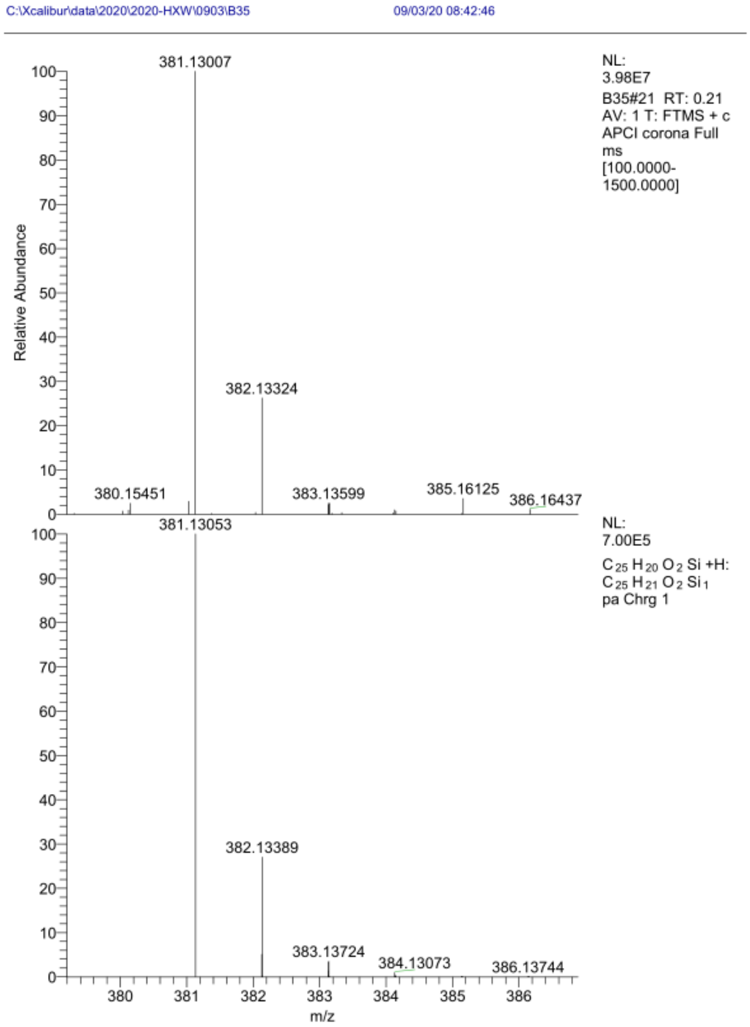

C:\Xcalibur\data\2020\2020-HXW\0903\B35
09/03/20 08:42:46
NL:
3.98E7
B35#21 RT: 0.21
AV: 1 T: FTMS + c
APCI corona Full
ms
[100.0000-
1500.0000]
381.13007
382.13324
380.15451
383.13599
385.16125
386.16437
Relative Abundance
NL:
7.00E5
C 25 H 20 O 2 Si +H:
C 25 H 21 O 2 Si 1
pa Chrg 1
381.13053
382.13389
383.13724
384.13073
386.13744
m/z

8

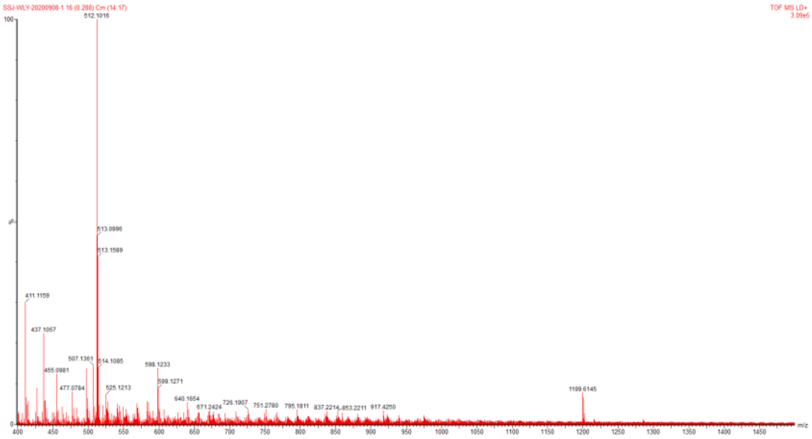

SSJ-WLY-20200908-1 16 (0.288) Cm (14:17)
TOF MS LD+
3.09e5
512.1016
513.0996
513.1589
411.1159
437.1057
507.1361
514.1085
455.0981
477.0784
525.1213
598.1233
599.1271
640.1654
671.2424
726.1907
751.2780
795.1811
837.2214
853.2211
917.4250
1199.6145
m/z

11

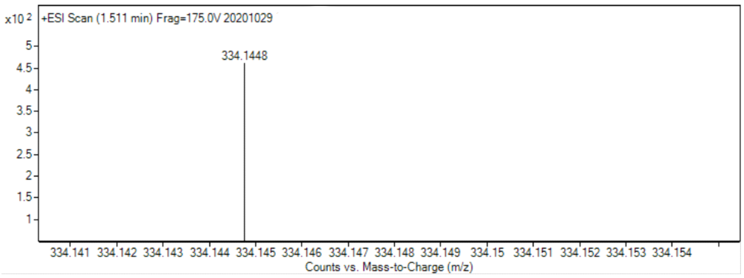
x10 2
+ESI Scan (1.511 min) Frag=175.0V 20201029
334.1448
Counts vs. Mass-to-Charge (m/z)

12

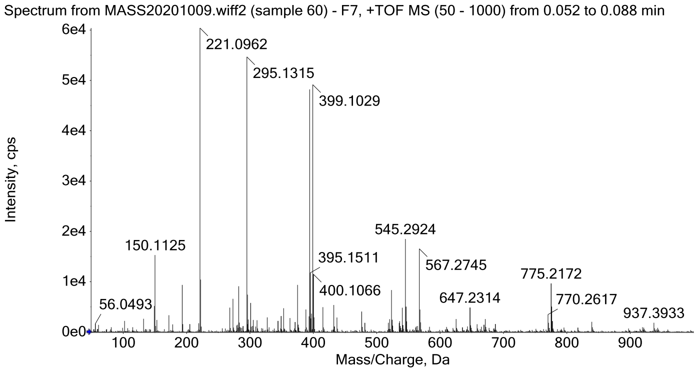
Spectrum from MASS20201009.wiff2 (sample 60) - F7, +TOF MS (50 - 1000) from 0.052 to 0.088 min
221.0962
295.1315
399.1029
150.1125
545.2924
395.1511
567.2745
400.1066
775.2172
647.2314
770.2617
56.0493
937.3933
Intensity, cps
Mass/Charge, Da

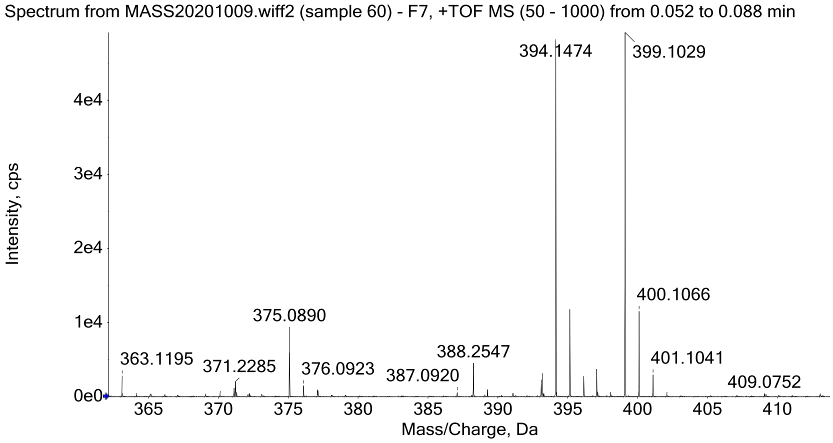
Spectrum from MASS20201009.wiff2 (sample 60) - F7, +TOF MS (50 - 1000) from 0.052 to 0.088 min
394.1474
399.1029
400.1066
375.0890
388.2547
401.1041
363.1195
371.2285
376.0923
387.0920
409.0752
Intensity, cps
Mass/Charge, Da

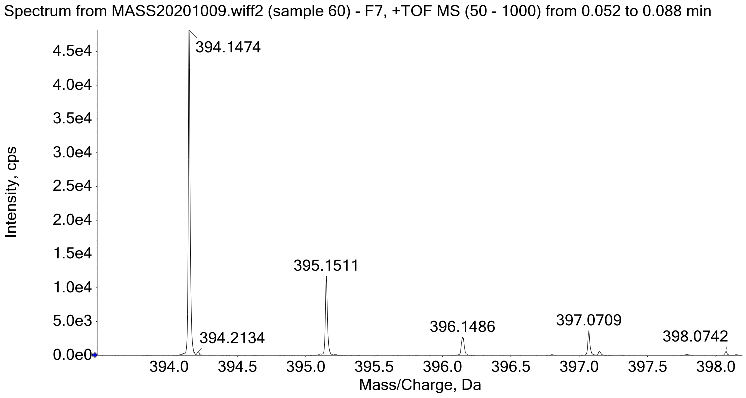
Spectrum from MASS20201009.wiff2 (sample 60) - F7, +TOF MS (50 - 1000) from 0.052 to 0.088 min
394.1474
395.1511
396.1486
397.0709
394.2134
398.0742
Intensity, cps
Mass/Charge, Da

14

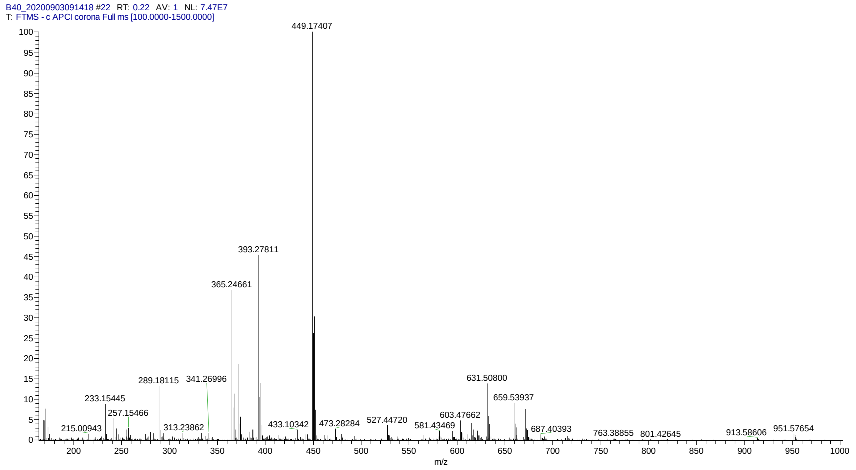
B40_20200903091418 #22 RT: 0.22 AV: 1 NL: 7.47E7
T: FTMS - c APCI corona Full ms [100.0000-1500.0000]
449.17407
393.27811
365.24661
289.18115
341.26996
233.15445
257.15466
215.00943
313.23862
433.10342
473.28284
527.44720
581.43469
603.47662
631.50800
659.53937
687.40393
763.38855
801.42645
913.58606
951.57654
m/z

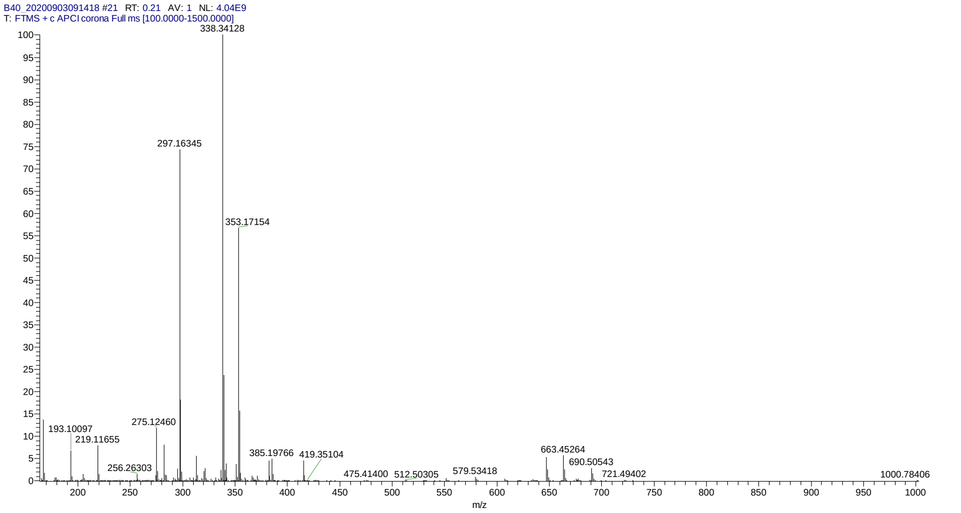
B40_20200903091418 #21 RT: 0.21 AV: 1 NL: 4.04E9
T: FTMS + c APCI corona Full ms [100.0000-1500.0000]
338.34128
297.16345
353.17154
275.12460
193.10097
219.11655
256.26303
385.19766
419.35104
475.41400
512.50305
579.53418
663.45264
690.50543
721.49402
1000.78406
m/z

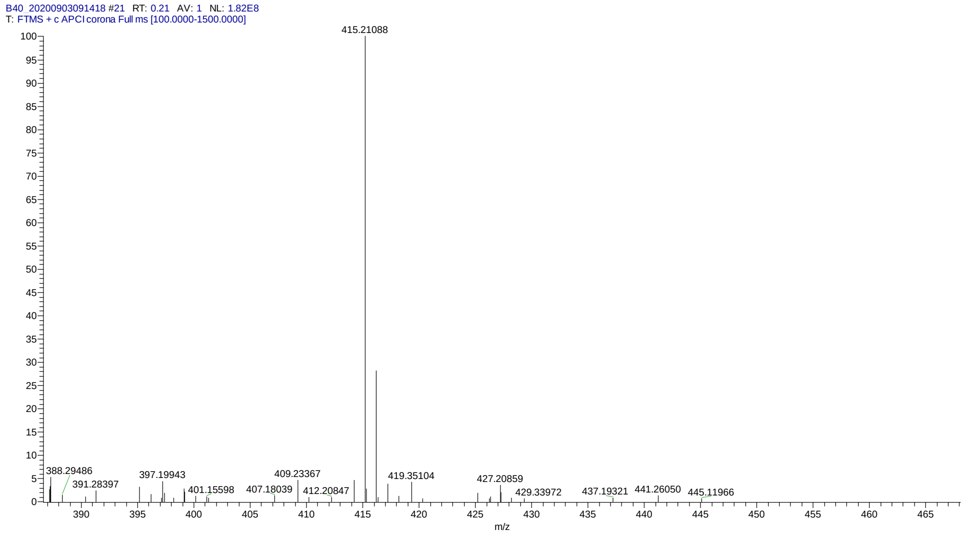
B40_20200903091418 #21 RT: 0.21 AV: 1 NL: 1.82E8
T: FTMS + c APCI corona Full ms [100.0000-1500.0000]
415.21088
388.29486
391.28397
397.19943
401.15598
407.18039
409.23367
412.20847
419.35104
427.20859
429.33972
437.19321
441.26050
445.11966
m/z
